# First insight into the proteome landscape of the porcine short posterior ciliary arteries: Key signalling pathways maintaining physiologic functions

**DOI:** 10.1038/srep38298

**Published:** 2016-12-06

**Authors:** Caroline Manicam, Natarajan Perumal, Norbert Pfeiffer, Franz H. Grus, Adrian Gericke

**Affiliations:** 1 Department of Ophthalmology, University Medical Centre of the Johannes Gutenberg University Mainz, Mainz, Germany

## Abstract

Short posterior ciliary arteries (sPCA) provide the major blood supply to the optic nerve head. Emerging evidence has linked structural and functional anomalies of sPCA to the pathogenesis of several ocular disorders that cause varying degrees of visual loss, particularly anterior ischaemic optic neuropathy and glaucoma. Although the functional relevance of this vascular bed is well-recognized, the proteome of sPCA remains uncharacterized. Since the porcine ocular system closely resembles that of the human’s and is increasingly employed in translational ophthalmic research, this study characterized the proteome of porcine sPCA employing the mass spectrometry-based proteomics strategy. A total of 1742 proteins and 10527 peptides were identified in the porcine sPCA. The major biological processes involved in the maintenance of physiological functions of the sPCA included redox and metabolic processes, and cytoskeleton organization. These proteins were further clustered into diverse signalling pathways that regulate vasoactivity of sPCA, namely the tight junction, α- and β-adrenoceptor, 14-3-3, nitric oxide synthase and endothelin-1 -mediated signalling pathways. This study provides the first insight into the complex mechanisms dictating the vast protein repertoire in normal vascular physiology of the porcine sPCA. It is envisioned that our findings will serve as important benchmarks for future studies of sPCA.

Short posterior ciliary arteries (sPCA) are the major blood suppliers to the optic nerve head (ONH). In humans, the sPCA arise from the medial branching of the ophthalmic artery, which emerges from the internal carotid artery^1^,^2^,^3^. Circulatory insufficiency in these retrobulbar blood vessels constitute one of the crucial contributing factors to the pathogenesis of several vision threatening ocular disorders, especially anterior ischaemic optic neuropathy (AION) and glaucomatous optic neuropathy (GON)^4^,^5^,^6^,^7^,^8^. The global burden of visual impairment due to glaucoma is projected to escalate by the year 2040, with almost 111.8 million of the world population affected by this second leading cause of blindness and 11.1 million of these are estimated to be bilaterally blind^9^,^10^,^11^,^12^ Likewise, up to 82 in 100 000 individuals are estimated to suffer from the most common type of AION, non-arteritic anterior ischemic optic neuropathy (NAION) annually^13^,^14^. Over the years, mounting evidence has associated these debilitating ocular diseases with hemodynamic alterations in the ONH^15^,^16^,^17^ Although elevated intraocular pressure (IOP) has been identified as the primary disease factor for glaucoma, its pathogenesis still remains a topic of much challenge in ophthalmic research owing to myriad other risk factors that may eventually cause optic nerve and retinal dysfunctions, regardless of the IOP^5^,^18^,^19^. It has been suggested that treatment options which increase ONH perfusion may facilitate clinical management of GON more effectively^20^.

The study of the pathogenesis of GON has made some progress in recent years with the use of animal models and genomic tools^21^,^22^. Although molecular technologies have profoundly facilitated the discovery of gene expression profiles in disease conditions, it is the effectors i.e. proteins that are the major players which regulate normal physiological functions. Therefore, proteomics, the protein cognate of genomics, has emerged as a powerful tool to characterize the protein expressions, post-translational modifications and to identify candidate disease biomarkers in pathological states compared to normal condition. Nevertheless, there is a paucity of information on the cellular signalling mechanisms underlying perturbed ocular microcirculation. A confounding challenge in ophthalmic research is the limited availability of human tissue samples for analysis. Therefore, it is important to employ samples from animal models that closely resemble those of human’s for highly translational results. In light of this, porcine ocular tissues were used in this study due to the high phylogenetic and morphological similarities to the human eye^23^. For decades, the pig has become an animal model of choice in biomedical research to study various human pathologies, including vascular functions in disease conditions compared to healthy controls^24^,^25^. Additionally, the pig eye is commonly used in vision research and is also a validated animal model to study glaucoma^26^.

Considering the functional relevance and importance of the sPCA in the perfusion of ONH and, the dearth of studies investigating the molecular regulators that maintain physiological functions in ocular blood vessels, this is the first study to characterize the fundamental cellular signalling mechanisms employing the mass-spectrometry-based proteomics approach. It is projected that the findings emerging from this study will provide an in-depth mechanistic insight into the complex cell signalling pathways that orchestrate circulatory functions in the sPCA and furnish vital information at the protein level. Finally, the methodology employed in this investigation, particularly catered for optimum protein extraction and proteome characterization of ocular blood vessels, is envisaged to be instrumental for future studies utilizing other ocular vascular beds.

## Results

### Identification of porcine sPCA proteins

**Figure 1:**
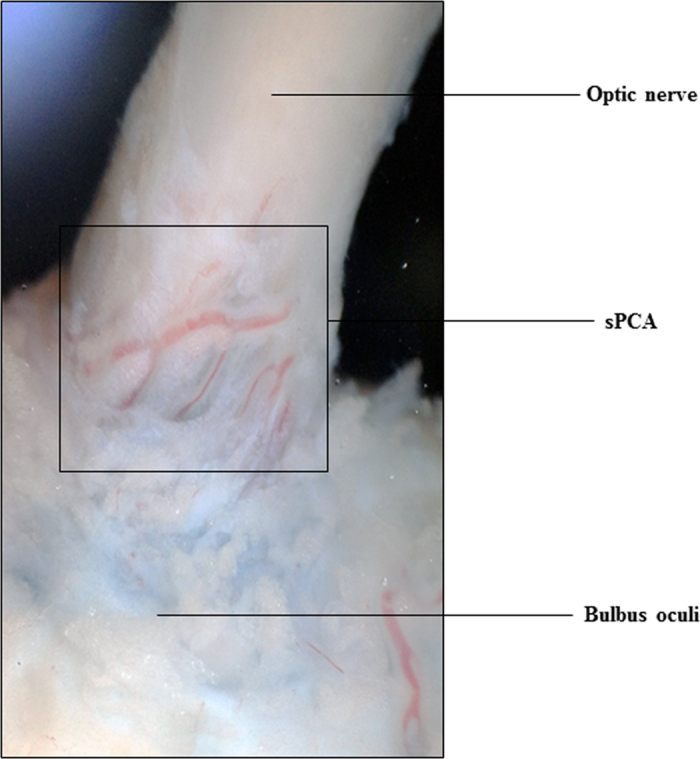
The porcine short posterior ciliary arteries. Photograph showing the short posterior ciliary arteries that enter the eye globe close to the optic nerve.

**Figure 2:**
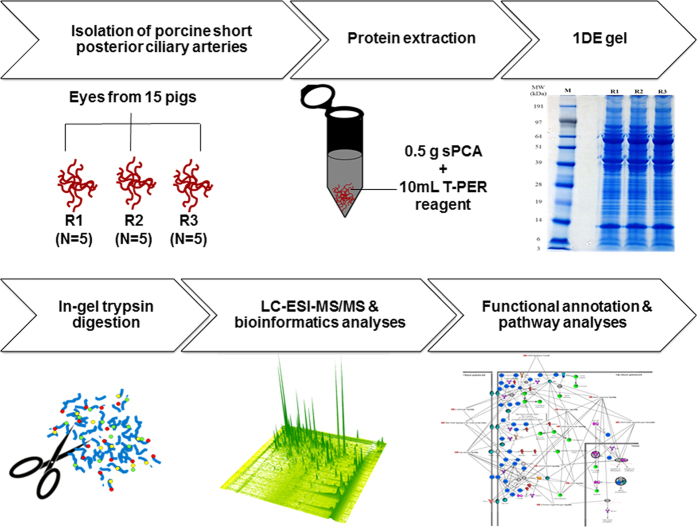
Workflow overview for mapping of the porcine sPCA proteome. Freshly isolated porcine sPCA were pooled equally into three biological replicates, represented by R1, R2 and R3. Samples were extracted with T-PER tissue protein extraction reagent and subjected to 1DE gel electrophoresis. The protein bands were sliced and trypsin-digested prior to proteomic analysis by LC-MS/MS. Finally, the emerging datasets were subjected to robust bioinformatics analyses and functional annotations of the significant gene ontology (GO) terms and pathways employing various tools to produce a comprehensive map of the normal porcine sPCA proteome.

**Figure 3:**
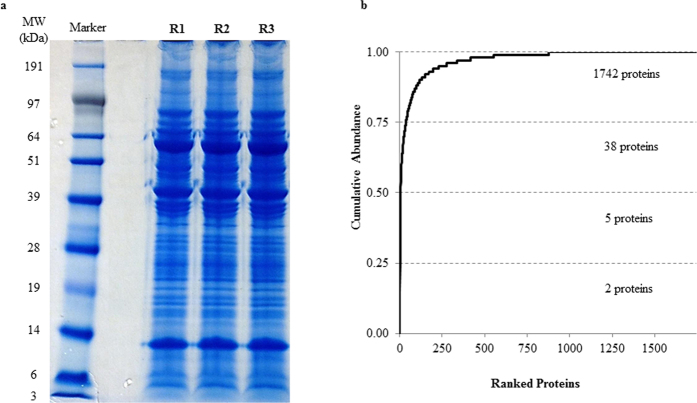
Proteomics analysis employing the 1DE & LC-ESI-MS/MS strategy reveals the inherent characteristics of the porcine sPCA. (**a**) Representative protein profiles of the sPCA resolved in 1DE gel after colloidal blue staining. (**b**) Quantitative analysis employing iBAQ shows the cumulative relative abundance of identified proteins, ranked from the highest to the lowest abundance.

This study endeavoured to profile the proteome of porcine sPCA (Fig. 1). The experimental workflow overview of this study is depicted as a schematic representation in Fig. 2. A total of 1742 proteins and 10 527 peptides were identified with a false discovery rate (FDR) of less than 1% after removal of reverse hits (Supplementary Table S1). The protein profiles of this vascular bed resolved in one-dimensional gel electrophoresis (1DE) is as depicted in Fig. 3a. With the approach of intensity-based absolute quantification (iBAQ), the relative abundance of the identified proteins was estimated. Of the 1742 proteins identified, the five most abundant proteins comprise 50% of the proteome, while 38 most abundant proteins accounted for 75% of the porcine sPCA proteome (Fig. 3b, Table 1).

### Proteomic profiles and functional annotations of sPCA

**Figure 4:**
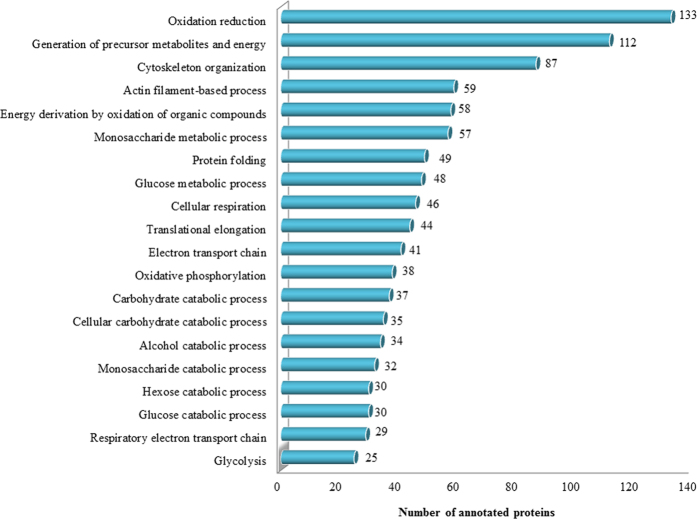
Functional classification of the sPCA proteome associated with over-represented GOBP terms. Top twenty significantly (p < 0.001) enriched biological processes involved in the proper functionality of the porcine sPCA analysed employing the DAVID tool.

**Figure 5:**
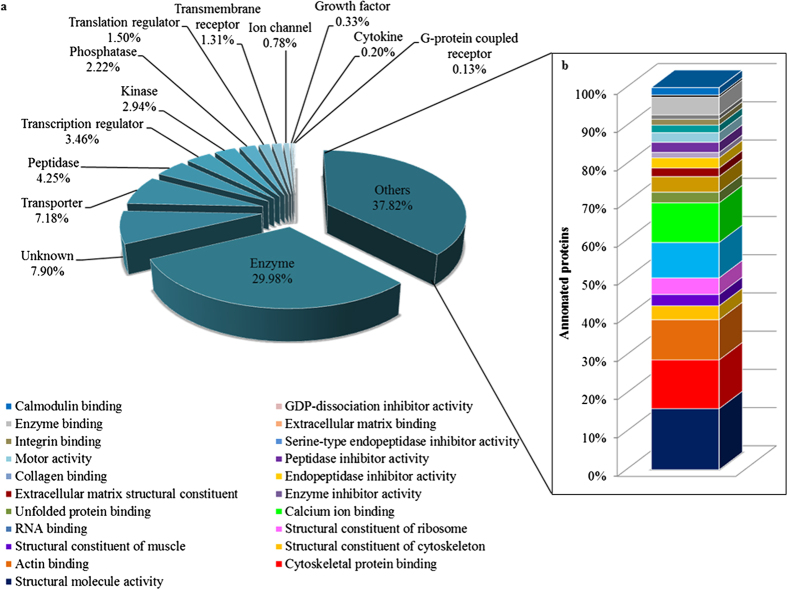
Functional classification of the sPCA proteome associated with over-represented GO molecular type terms. (**a**) Data presented as a bar chart of proteins clustered according to their molecular types analysed employing IPA database. (**b**) Further analysis of the molecular identity of the proteins categorised as ‘others’, represented by stacked bar chart of the most significant (p < 0.001) molecular functions as analysed by DAVID tool.

The classification of the identified sPCA proteins was performed employing the Ingenuity Pathways Analysis software (IPA, Ingenuity QIAGEN Redwood City, CA) (www.qiagen.com/ingenuity), DAVID tool (version 6.7) (http://david.abcc.ncifcrf.gov/home.jsp) and Protein ANalysis THrough Evolutionary Relationships (PANTHER, http://pantherdb.org) functional annotation tools. First, the analysis of the major Gene Ontology Cellular Component terms (GOCCs) employing IPA demonstrated that the top four subcellular localizations of the sPCA proteins corresponded to cytoplasm (56%), while almost 14% and 10% of the total proteins are from the nucleus and plasma membrane, respectively. Only a small percentage (9%) of the total proteins was localized in the extracellular space, while the remaining 11% comprises unknown and other proteins, as shown in Supplementary Fig. S1. Next, to assess the biological relevance of the identified proteins, enrichment analysis employing DAVID was performed. These proteins were classified into 626 distinct categories of GO Biological Process (GOBPs) terms, with 227 significant processes (p < 0.001) involved in the maintenance of physiological functions in the sPCA (Supplementary Table S2). Figure 4 shows that the top twenty significant biological processes comprise proteins which are mainly involved in oxidation-reduction processes (133 proteins), generation of precursor metabolites and energy (112 proteins) and 87 proteins in cytoskeleton organization. Six clusters of proteins were involved in various catabolic processes, composed of the carbohydrate (37 proteins), cellular carbohydrate (35 proteins), alcohol (34 proteins), monosaccharide (32 proteins), hexose and glucose (30 proteins each) catabolic processes. Another two clusters were responsible for electron transport chain and the remaining clusters were implicated in protein folding, translational elongation, oxidative phosphorylation, glucose metabolism and glycolysis. In the category of GO molecular types analysed employing IPA, almost 30% of the total proteins function as enzymes, transporters (7.18%), peptidases (4.25%), transcription regulators (3.46%), kinases (2.94%) and phosphatases (2.22%), while an approximately 38% proteins were assigned into the category of other molecular types, as depicted in Fig. 5a. The molecular identity of these ‘other’ proteins were further dissected employing the DAVID analysis tool and the highly significant (p < 0.001) ones largely represented proteins involved in structural molecule activities (16%), cytoskeletal protein, actin, RNA, calcium ion, unfolded protein, collagen, integrin, extracellular matrix, enzyme and calmodulin binding (13, 11, 9, 10, 3, 1, 2, 1, 5 and 2%, respectively) and other structural constituents (11%), as shown in Fig. 5b.

### Pathway and protein networks analysis

**Figure 6:**
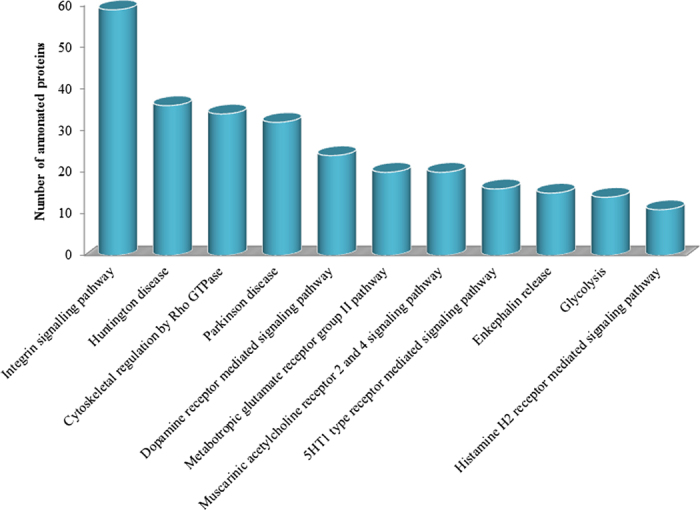
Functional classification of the sPCA proteome according to enriched canonical pathways. Eleven functional and disease pathways were significantly (p < 0.001) implicated in the proteome of sPCA, identified employing the PANTHER tool.

The identified proteins were analysed with PANTHER to classify them according to associated canonical pathways and the pathways that exhibited significant values were distinguished. The following are the notably significant (p < 0.001) pathways implicated in the porcine sPCA (numbers in brackets indicate the number of proteins identified in each pathway and the p value), as demonstrated in Fig. 6: Integrin signalling pathway (59; p = 1.37E-09), Huntington disease pathway (36; p = 3.62E-04), cytoskeletal regulation by Rho GTPase (34; p = 2.45E-07), Parkinson disease pathway (32; p = 5.27E-07), dopamine receptor mediated signalling pathway (24; p = 1.86E-04), metabotropic glutamate receptor group II pathway (20; p = 4.64E-06), muscarinic acetylcholine receptor 2 and 4 signalling pathway (20; p = 2.86E-05), 5HT1 type receptor mediated signalling pathway (16; p = 2.14E-04), enkephalin release (15; p = 2.64E-05), glycolysis (14; p = 2.55E-06) and histamine H2 receptor mediated signalling pathway (11; p = 8.04E-04).

**Figure 7:**
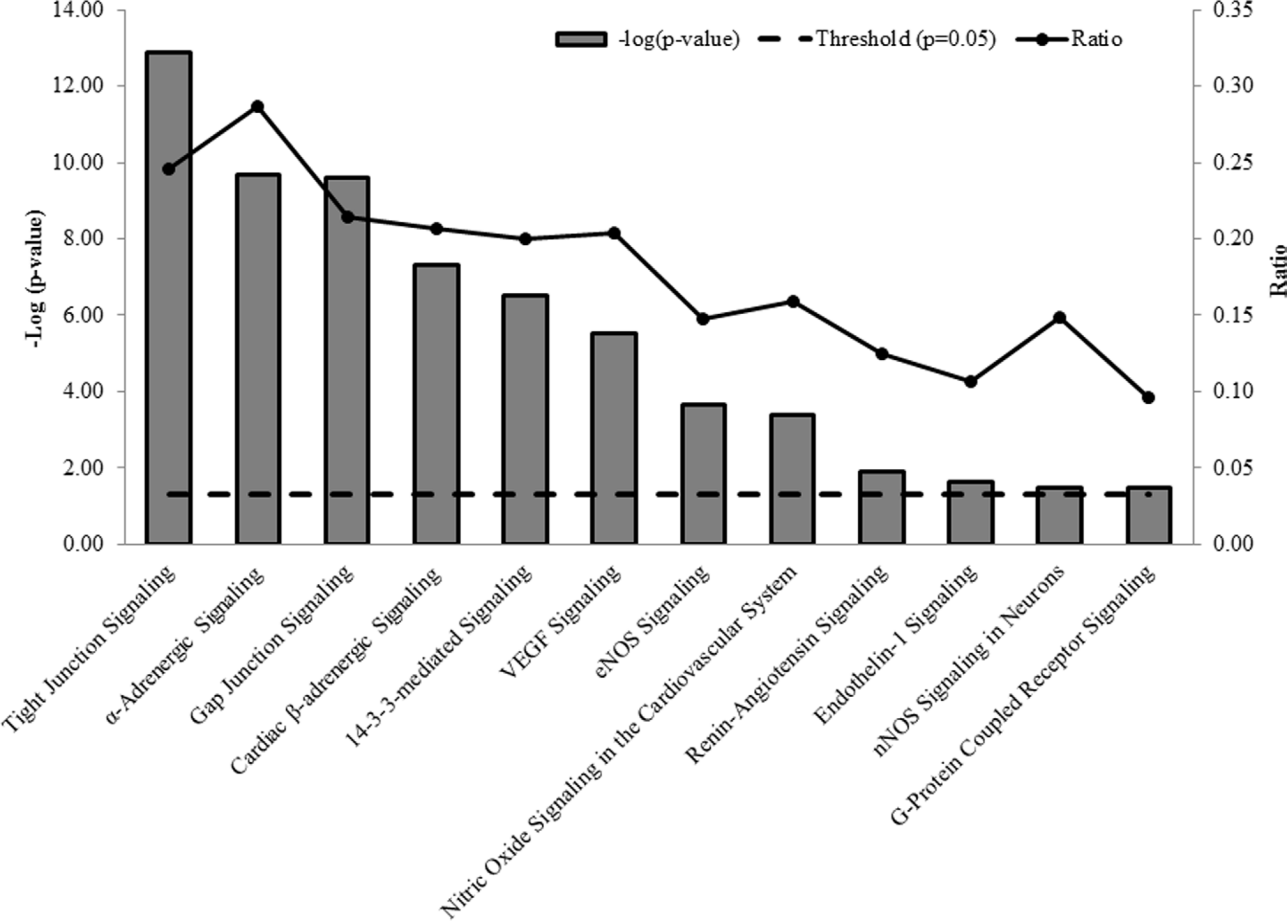
Canonical pathways involved in vasoactivity of the sPCA. This bar chart shows the canonical pathways which were significantly (p < 0.001) implicated in the vasoactivity of the sPCA, analysed employing IPA. The negative of the log_10_ (p-value; bar chart) and ratio (number of focus molecules involved in the pathway/total number of molecules in the pathway; solid line joined by circles) are plotted on the primary and secondary Y-axes, respectively.

**Figure 8:**
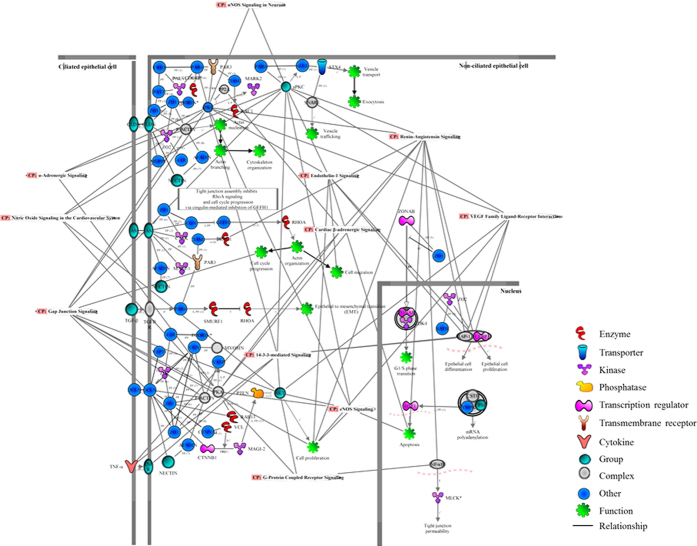
Protein interaction networks of merged vasoactive pathways in the sPCA. The significantly (p < 0.001) expressed vasoactive pathways were merged to elucidate their PPI networks. The different shapes of the nodes indicate the gene or gene products of the annotated proteins and the relationship between nodes is represented as an edge (line).

Next, the proteins identified in the present study were analysed employing the IPA database to characterize pathways which are responsible for vasoactivity. To determine the p-values for protein datasets within the predetermined canonical pathways, the significance was calculated employing Benjamini-Hochberg corrected Fisher’s exact test, and the ratio represented the number of molecules in the dataset associated with a particular pathway to the total number of molecules in that pathway. The top significant (p < 0.05) pathways which are implicated in the maintenance of vasoactivity of the sPCA are shown in Fig. 7 and these included tight junction signalling (p = 1.26E-13; ratio = 0.25), α-adrenergic signalling (p = 2.04E-10; ratio = 0.29), gap junction signalling (p = 2.51E-0.21; ratio = 0.21), cardiac β-adrenergic signalling (p = 5.01E-08; ratio = 0.21), noradrenaline and adrenaline degradation (p = 1.29E-07; ratio = 0.35), 14–3–3-mediated signalling (p = 3.16E-07; ratio = 0.20), vascular endothelium growth factor (VEGF) signalling (p = 3.09E-06; ratio = 0.20), endothelial nitric oxide synthase (eNOS) signalling (p = 2.19E-04; ratio = 0.15), nitric oxide signalling in the cardiovascular system (p = 4.27E-04; ratio = 0.16), renin-angiotensin signalling (p = 1.26E-02; ratio = 0.13), endothelin-1 signalling (p = 2.24E-02; ratio = 0.11), nNOS signalling in neurons (p = 3.31E-02; ratio = 0.15) and G-protein coupled receptor signalling (p = 3.47E-02; ratio = 0.10). Figure 8 is a schematic overview of the interplay between all significant pathways mediating vasoactivity in sPCA, which were merged to observe their protein-protein interactions (PPI). A high number of interactions were found to be clustered in actin cytoskeleton organization and various cellular processes, including cell proliferation, differentiation, apoptosis, migration, cell cycle progression and tight junction permeability. Additionally, there are some transport proteins involved in exocytosis and vesicle trafficking. Interestingly, the renin-angiotensin signalling has the highest PPI, followed by the gap junction and VEGF pathways. Among the interacting proteins, the tight junction protein 1 (*ZO1*) has the highest number of interactions (10 PPI) with other proteins, followed closely by cingulin (*CGN*) with 8 PPI, atypical protein kinase C (*aPKC*) and occludin (*OCLN*) with 6 PPI each and, tight junction protein 2 (*ZO2*) with 5 PPI. In addition to PPI, most proteins namely *CGN, aPKC* and *ZO2* also exhibited inhibitory action on other proteins, while some proteins such as par-6 family cell polarity regulator alpha (*PAR6*), symplekin (*SYMP*) and membrane-associated guanylate kinase (*MAGI-2*) activated other proteins. Full details of the proteins and their interactions in the merged vasoactive signalling pathways can be found in Supplementary Table S3.

## Discussion

The present study provides the first insight into the complexity of the porcine sPCA proteome, represented by 1742 proteins and 10 527 peptides. The clinical and functional studies of sPCA have gained immense interest in recent years attributed to several lines of evidence that link compromised blood flow in these retrobulbar vessels to debilitating ocular pathologies, especially in glaucoma and NAION^2^,^6^,^19^. It is of interest to note that among the numerous signalling pathways identified in sPCA in this study, the top four most significantly detected comprised the integrin, Huntington disease, Rho GTPase and Parkinson disease-related pathways.

Blood vessels are highly dynamic circulatory organs that undergo constant structural and functional adaptations, called vascular remodelling, in response to various stimuli, specifically pulsatility of blood flow and pressure^27^,^28^. This remodelling process involves many crucial physiological mechanisms comprising cell-matrix and cell-to-cell communication, which are mediated primarily by integrins that act as cell adhesion receptors^29^,^30^. It is therefore not surprising that the integrin signalling complexes are referred to as ‘master regulators’ of cellular functions^31^. Integrins are membrane-spanning ubiquitous proteins that orchestrate a wide variety of cellular functions, as well as a myriad of intracellular signalling pathways^30^,^32^. In vascular cells, integrins are key protein entities that have established roles in mechanotransduction to detect and respond to shear stress and pressure, and consequently modify vascular tone to compensate for environmental changes and effectively restore normal vascular functions^33^. Furthermore, endothelial and vascular smooth muscle integrins have emerged as attractive therapeutic candidates for an array of vasculopathies^32^. In respect to integrins’ translational potential in the clinical settings, there is an upsurge of interest in the ocular research community in the role played by these transmembrane glycoproteins in the pathogenesis of glaucomatous optic neuropathy (GON)^34^,^35^,^36^.

On the other hand, integrins are also closely associated with Rho family GTPase in modulating junctional integrity in vascular endothelial cells and in signal transduction for cytoskeletal organization^37^,^38^,^39^. Vascular permeability is tightly regulated under physiological conditions by intercellular tight junctions and vascular endothelial growth factor (VEGF), which work in concert with Rho-GTPases to form semipermeable paracellular barriers that control passage of solutes and also maintain cell polarity^40^,^41^,^42^. Correspondingly, the findings from the current study have also demonstrated that the tight junction and VEGF signalling pathways were implicated in the maintenance of vasoactivity of the sPCA (Fig. 7). Apart from regulating diverse cellular functions to maintain physiological integrity, the Rho family GTPases are intimately implicated in maintaining vascular homeostasis^43^. In blood vessels, the Rho/Rho-associated protein kinase (ROCK) pathway is expressed abundantly in the vascular smooth muscle and is known to regulate vasoreactivity^44^,^45^. Noteworthy in this context is that this family of proteins is recognized as key signalling molecules that regulate arterial blood pressure and are important mediators involved in maintaining vascular tone in pathophysiological conditions, such as hypertension^46^,^47^. Hence, any alterations in the hemodynamic balance and level of activation of the Rho protein family members may compromise the vascular integrity and lead to various vasculopathies^43^,^48^. It is well-known that the ROCK inhibitors are able to relax vascular smooth muscle cells and may enhance ocular blood flow^49^. As such, the Rho/ROCK signalling pathway has emerged as a promising druggable target to treat GON because one of the major causative factors of blindness in glaucoma is recognized to arise from perturbed perfusion at the level of optic nerve head^50^,^51^,^52^.

It is intriguing that apart from identification of proteins involved in the organization of cytoskeleton and maintenance of cellular functions, two other clusters of proteins involved in neurodegenerative disease signalling pathways, the Huntington and Parkinson’s disease, were also identified with high significance in the porcine sPCA. A large proportion of the proteins identified in the Parkinson’s disease pathway consisted of 14-3-3, proteasomes and to a lesser extent, heat shock protein (HSP) 70 kDa and synucleins (Supplementary Table S4). The regulation of 14-3-3 family of proteins is tightly controlled in normal cellular milieu and implicated in a broad spectrum of both general and specialized signalling pathways, indicating their pivotal roles in health and disease^53^,^54^. Albeit their ‘global’ roles in cell cycle and programmed cell death, as well as in protein trafficking in multiple tissue types, these proteins are found most abundantly in neurons of the brain and are associated with the pathogenesis of neurodegenerative disorders, namely Parkinson’s disease^55^,^56^,^57^. Consistent with this, a study by Yacoubian *et al*. employing cellular and animal models of Parkinson’s disease has demonstrated that binding impairment of multiple isoforms of 14-3-3 to α-synuclein led to toxicity in dopaminergic neurons^58^. Interestingly, the 14-3-3-mediated signalling was found to have significant role(s) in the vasomotricity of sPCA in the present study, although the exact mechanism(s) how this signalling network functions in this vascular bed remains to be determined. Basal levels of 14-3-3 isoforms were also detected in other arterial beds such as in the healthy human coronary arteries, with potential function in vascular smooth muscle activation and response to arterial injury^56^. Conversely in disease conditions, for example in diabetic animals with occlusion of the middle cerebral artery, the expression of 14-3-3 proteins was diminished and this exacerbated brain damage^59^. It is well-recognized that the plasticity of the vascular smooth muscle cells is a pivotal adaptation mechanism to physiological and environmental changes, including cell proliferation, extracellular matrix organization and apoptosis^60^,^61^. Since one of the primary functions of this protein family is to antagonize apoptotic signals and thereby, inhibit cell death^62^, the expression levels of the 14-3-3 proteins in porcine sPCA in the current study conceivably reflect its functional relevance in the maintenance of vascular integrity in normal physiological state. It is noteworthy that in the ocular system, abnormalities of 14-3-3-mediated signalling are related to the pathogenesis of glaucoma^63^.

A plethora of proteasome isoforms were also found in the Parkinson’s disease pathway in the porcine sPCA. This multifaceted ubiquitous proteinase governs a wide array of intracellular functions, with particular function in degradation of damaged proteins^64^,^65^. In a study employing normal animals, the inhibition of proteasome system proved lethal and led to the development of vasculopathy, where altered microvascular permeability and cardiac apoptosis were observed, to name a few detrimental effects of proteasome dysfunction^66^. Additionally, angiogenesis is regulated through proteasome and any deleterious changes in its functional activity will affect the formation of blood vessels^67^,^68^. On the other hand, given the vital regulatory role of redox signalling in various vascular smooth muscle cell patho(biology), also as evidenced by the significantly high expression of proteins involved in the oxidation-reduction activities in the porcine sPCA (Fig. 4), it is not wrong to conceptualize the importance of interplay between the ubiquitin-proteasome system and cellular redox processes^69^. The oxido-reductive signalling plays integral roles in vascular remodelling processes, importantly in the regulation of cytoskeletal dynamics and modulation of smooth muscle cell differentiation^27^,^70^. Concomitant identification of actin-related complexes and tubulins in the Huntington disease pathway in the current exploratory study comes as no surprise as these are arguably among the most overwhelmingly expressed proteins in cells, which assemble to construct complex networks of cytoskeleton^71^,^72^, and this is in agreement with the significant detection of the actin-cytoskeleton processes in the porcine sPCA (Fig. 4). It goes without saying that any cytoskeletal aberrations are therefore, responsible for cellular contractile dysfunction^73^.

Interestingly, the proteomic findings emerging from the present study on the pathways involved in the vasoactivity of the porcine sPCA not only provide novel insight at the protein level, but are also in agreement with previous *in vivo* and *in vitro* studies carried out with pharmacological approaches. In retrospect, studies have demonstrated that the α_1_-adrenergic and 5-HT receptors are responsible for mediating vasoconstriction, while the β-adrenergic receptors mediated vasodilation in the human posterior ciliary arteries^74^,^75^. This corroborates with the detection of both α- and β-adrenergic, and 5-HT receptor-mediated signalling pathways with high significance in the current study. Likewise, the endothelin-1 (ET-1) signalling was also significantly detected in this investigation and this protein signalling has been associated with the pathogenesis of glaucoma, regardless of the intraocular pressure. Perturbances in the blood flow velocity were found primarily in the retrobulbar vasculature of glaucoma patients, with most pronounced changes in the sPCA^7^. ET-1 signalling is not only implicated in the regulation of smooth contraction, but also functions as an important mediator in inflammation^76^,^77^. On the other hand, neuronal mediators of vasoactivity comprising the muscarinic acetylcholine receptor (Chrm) 2 and 4 signalling pathways were also detected in this ocular vascular bed in the present investigation. Both receptor subtypes selectively activate the G-proteins of the G_i_/G_o_ family^78^. Studies carried out with Chrm 2^−/−^ mice strongly suggest that Chrm 2 is the predominant autoreceptor that mediates cortical and hippocampal release of acetylcholine^79^,^80^. Since the sPCA are autonomically innervated, acetylcholine released from the cholinergeic nerves seemed to modulate nitroxidergic nerve functions in porcine ciliary arteries^74^,^75^. Presynaptic Chrm 2 was also identified in the mouse cerebral arteries and was implicated in neurogenic vasoconstriction *via* inhibition of nitric oxide synthase (NOS)^81^. Consistent with this mechanism, several NOS pathways were also found to be significantly expressed in the porcine sPCA in this study. Besides, this Chrm subtype was also known to exert anti-apoptotic effects on cardiomyocytes and mediate antinociception^78^,^82^.

Taken together, the vascular integrity of the porcine sPCA is maintained by a plethora of highly dynamic protein networks that ensure proper vessel functionality and physiology, as evidenced by this study. For the first time, the expanding paradigm of exploratory proteomics has provided a sophisticated and unbiased approach to unravel the biological processes governing fundamental cell functions in a retrobulbar vascular bed at the protein level. However, a limitation of the present study is that the proteins identified from the sPCA cannot be particularly ascribed to the different layers that make up the blood vessels i.e. the adventitia, vascular smooth muscle and endothelial cells. It would be therefore interesting to further elucidate, compare and contrast the proteome of the major layers of the sPCA in future investigations utilizing the proteomic and bioinformatics platform established in this study. In conclusion, the findings emerging from our study are envisaged to serve as important benchmark and database for future studies employing porcine sPCA.

## Materials and Methods

### Animals

All experiments were conducted in adherence to the Association for Research in Vision and Ophthalmology (ARVO) Statement for the Use of Animals in Ophthalmic and Vision Research and by institutional guidelines. The study was conducted and approved at the Department of Ophthalmology, University Medical Centre Mainz. Fresh eyes from pigs (Piétrain breed) approximately 3–6 months old were obtained from a local abattoir immediately post-mortem. The enucleated eyes attached with optic nerve and extraocular tissues were transported to the laboratory in ice-cold Krebs-Henseleit buffer at pH 7.4 with the following ionic composition in mM: 118.3 NaCl, 4.7 KCl, 2.5 CaCl_2_, 1.2 MgSO_4_, 1.2 KH_2_PO_4_, 25 NaHCO_3_, and 11 glucose (Carl Roth GmbH, Karlsruhe, Germany).

### Protein extraction from sPCA

The porcine ciliary arterial branches are divided into the short and long posterior ciliary arteries, as described elsewhere^83^,^84^,^85^. Therefore, to standardize the isolation procedure, the arterial branch used in the current study was restricted to the paraoptic sPCA that travel along the optic nerve to enter the eye globe, as shown in Fig. 1. The luminal diameter of the isolated vessels is between 130 and 250 μm. Branches of the sPCA were carefully dissected and cleaned of connective tissues using fine-point tweezers under a dissecting microscope. Thereafter, the isolated blood vessels were gently rinsed in ice-cold phosphate buffered saline (PBS) to remove any blood contaminants and were used for proteomic analysis. The sPCA from five pigs were pooled (which constituted one biologic replicate) and a total of three biological replicates were used in this study. Protein extraction from the porcine sPCA was carried out employing T-PER Tissue Protein Extraction Reagent (Thermo Scientific Inc., Waltham, MA, USA) according to the manufacturer’s instruction with slight modifications. Briefly, the isolated sPCA were weighed immediately after isolation and cleaning, followed by protein extraction with 10 ml T-PER reagent added to 0.5 g samples. Subsequently, the vascular samples in T-PER reagent were homogenized employing the BBY24M Bullet Blender Storm (Next Advance Inc., Averill Park, NY, USA) and centrifuged at 10,000 *× g* for 5 minutes to pellet the tissue debris. The supernatant was collected and subjected to sample cleaning employing the Amicon Ultra 0.5 mL centrifugal filters with 3 K cutoff (Merck Millipore, Carrigtwohill, Ireland). The protein concentration of the obtained eluate was determined using BCA Protein Assay Kit (Pierce, Rockford, IL).

### 1DE

The sPCA eluates were subjected to 1DE (50 μg sample per well) using 4–12% Bis-Tris Gels (Invitrogen, Karlsruhe, Germany) with MOPS running buffer under reducing conditions for 60 min with a constant voltage of 150 V, according to the manufacturer’s instructions. The SeeBlue Plus 2 (Invitrogen, Karlsruhe, Germany) pre-stained protein standard was used as a molecular mass marker. Next, the gels were stained with Colloidal Blue Staining Kit (Invitrogen, Karlsruhe, Germany) according to the manufacturer’s instructions. Then, protein bands (sliced into 39 bands per replicate) were cut into small pieces and subjected to dehydration utilizing neat acetonitrile prior to disulfide bonds cleavage with 10 mM 1,4-Dithiothreitol (DTT) in 100 mM ammonium bicarbonate (NH_4_HCO_3_) and alkylation with 55 mM iodoacetamide (IAA) in 100 mM NH_4_HCO_3_. The reduced and alkylated protein mixtures were digested with sequence grade-modified trypsin (Promega, Madison, USA) resuspended in 10 mM NH_4_HCO_3_ and 10% acetonitrile for 16 h at 37 °C. Subsequently, proteolysis was quenched by acidification of the reaction mixtures with 100 μl of extraction buffer composed of 1: 2 (vol/vol) of 5% formic acid: acetonitrile and, incubated for 15 min at 37 °C in a shaker. The supernatant containing peptides was collected and the remaining peptides in the gel pieces were extracted with two 20 minutes washes in extraction buffer. The supernatants were pooled and concentrated to dryness in SpeedVac (Eppendorf, Darmstadt, Germany) prior to storage at −20 °C^86^,^87^. Next, the peptides recovered from the in-gel digestion were subjected to purification employing ZipTip C18 columns (Millipore, Billerica, MA, USA) according to the manufacturer’s instructions. This peptide-purification procedure was repeated four times for each sample and the combined eluate was dried in the SpeedVac, dissolved in 10 μL of 0.1% trifluoroacetic acid (TFA) solution prior to LC-MS/MS analysis.

### LC-ESI-MS/MS

The LC-ESI-LTQ-Orbitrap MS system is well established in our laboratory and details of this system are described in detail elsewhere^86^,^88^,^89^. Briefly, solvent A which consisted of LC-MS grade water with 0.1% (v/v) formic acid, and solvent B consisting of LC-MS grade acetonitrile with 0.1% (v/v) formic acid were utilized. The gradient was run for 90 min per gel spot as follows; 0–50 min: 10–35% B, 50–70 min: 35–55% B, 70–75 min: 55–90% B, 75–80 min: 90% B, 80–83 min: 90–10% B und 83–90 min: 10% B. Continuum mass spectra data were acquired on an ESI-LTQ-Orbitrap-XL MS (Thermo Scientific, Bremen, Germany) and the general mass spectrometric conditions were as follows: positive-ion electrospray ionization mode, spray, capillary and the tube lens voltages were set at 4.5 kV, 48 V and 120 V, respectively, and the heated capillary temperature was set at 275 °C. The LTQ-Orbitrap was operated in a data-dependent mode of acquisition to automatically switch between Orbitrap-MS and LTQ-MS/MS acquisition. Survey full scan MS spectra (from *m*/*z* 300 to 2000) were acquired in the Orbitrap with a resolution of 30000 at *m/z* 400 and a target automatic gain control (AGC) setting of 1.0 × 10^6^ ions. The lock mass option was enabled in MS mode and the polydimethylcyclosiloxane (PCM) *m/z* 445.120025 ions were used for internal recalibration in real time^90^. The five most intense precursor ions were sequentially isolated for fragmentation in the LTQ with a collision-induced dissociation (CID) fragmentation, the normalized collision energy (NCE) was set to 35% with activation time of 30 ms with repeat count of 3 and dynamic exclusion duration of 600 s. The resulting fragment ions were recorded in the LTQ.

### Bioinformatics and Gene Ontology (GO) functional annotation analysis

The acquired continuum MS datasets were analysed by MaxQuant (version 1.5.2.8, http://www.maxquant.org/) computational proteomics platform and its built-in Andromeda search engine for peptide and protein identification, with iBAQ algorithm enabled^91^,^92^,^93^,^94^. The tandem MS spectra were searched against UniProtSP/TrEMBL (*Homo sapiens* and *Sus scrofa* database (date: 1^st^ July 2016) using standard settings with peptide mass tolerance of ±30 ppm, fragment mass tolerance of ±0.5 Da, with ≥6 amino acid residues and only “unique plus razor peptides” that belong to a protein were chosen^91^. For limiting a certain number of peak matches by chance, a target-decoy-based FDR for peptide and protein identification was set to 0.01. Carbamidomethylation of cysteine was set as a fixed modification, while protein N-terminal acetylation and oxidation of methionine were defined as variable modifications, enzyme: trypsin and maximum number of missed cleavages: 2. The output of the generated “proteingroups.txt” data from the MaxQuant analysis was utilized for subsequent functional annotation and pathway analysis.

The GO functional annotation analysis of identified proteins was carried out with three different bioinformatics tools. These analyses allowed elucidation of the different functions and processes in which the identified and validated proteins would be putatively involved. Three independent sets of ontology were used in the annotation: “the molecular function”, “the biological processes”, in which the proteins participate, and their “cellular component”. Proteins without similarity to database entries were not considered for collation. First, the IPA was used for interpreting the GOCC terms, molecule types and PPI networks associated with the identified proteins. Top canonical pathways involving in the vasoactivity of the identified sPCA proteins were presented, along with a *p*-value calculated using Fisher’s exact test. Next, the molecular interactions networks between proteins associated with top diseases and functions were reported by the PANTHER classification system (version 11.0, released on the 15^th^ July 2016), which includes 13096 protein families with 78442 functionally distinct protein subfamilies^95^. Finally, DAVID tool was used for interpreting the GOBP and GOMF terms of the identified sPCA proteins that could not be specifically dissected employing IPA^96^,^97^. The protein list was uploaded into DAVID and searched for enrichment for GOBP and GOMF terms, and the results were filtered based on threshold count ≥2 and P values < 0.05.

## Additional Information

**How to cite this article**: Manicam, C. *et al*. First insight into the proteome landscape of the porcine short posterior ciliary arteries: Key signalling pathways maintaining physiologic functions. *Sci. Rep.* **6**, 38298; doi: 10.1038/srep38298 (2016).

**Publisher's note:** Springer Nature remains neutral with regard to jurisdictional claims in published maps and institutional affiliations.

## Supplementary Material

Supplementary Data

## Figures and Tables

**Table 1:** Top 38 most abundant proteins in the porcine sPCA.

Rank	Protein Name	Gene Name	Peptides	Sequence Coverage (%)	iBAQ
1	Hemoglobin subunit beta	HBB	17	84.37	4.09E+09
2	Hemoglobin subunit alpha	HBA	12	87.03	3.00E+09
3	Uncharacterized protein	LOC100302368	8	45.50	2.65E+09
4	Cardiac muscle alpha actin 1	ACTC1	33	81.23	2.23E+09
5	Serum albumin	ALB	69	82.37	1.96E+09
6	Ig lambda chain C region	N/A	7	92.40	5.77E+08
7	Uncharacterized protein	LUM	15	47.30	5.16E+08
8	Transgelin	TAGLN	20	76.27	4.78E+08
9	Uncharacterized protein (Fragment)	N/A	5	80.70	4.06E+08
10	Protease serine 1 (Fragment)	PRSS1	1	23.80	3.74E+08
11	Actin, cytoplasmic 2	ACTG1	29	80.00	3.63E+08
12	Decorin	DCN	21	58.97	3.11E+08
13	IgG heavy chain	IGHG	18	51.33	2.66E+08
14	IgG heavy chain	IGHG	17	45.97	2.33E+08
15	Galectin	LGALS1	9	75.80	2.17E+08
16	Serotransferrin	TF	57	78.70	2.16E+08
17	Tropomyosin 1 (Alpha), isoform CRA	TPM1	33	61.43	2.03E+08
18	Osteoglycin/mimecan	OGN	16	44.50	1.86E+08
19	Vimentin	VIM	35	71.83	1.77E+08
20	S-arrestin	SAG	27	82.80	1.67E+08
21	Protein S100-A1	S100A1	2	38.30	1.63E+08
22	Uncharacterized protein	CA2	20	71.63	1.59E+08
23	Creatine kinase B-type	CKB	13	75.00	1.50E+08
24	Glyceraldehyde-3-phosphate dehydrogenase	GAPDH	20	70.60	1.47E+08
25	Protein S100	S100B	3	40.20	1.41E+08
26	Triosephosphate isomerase	TPI1	18	83.63	1.29E+08
27	Actin alpha 2	ACTA2	33	81.23	1.27E+08
28	Phosphatidylethanolamine-binding protein 1	PEBP1	15	79.10	1.26E+08
29	Cysteine and glycine-rich protein 1	CSRP1	11	56.00	1.24E+08
30	Synaptotagmin-7	SYT7	1	6.60	1.23E+08
31	Annexin A2	ANXA2	28	72.60	1.18E+08
32	Uncharacterized protein	TPM2	29	64.30	1.16E+08
33	Creatine kinase M-type	CKM	22	59.67	1.12E+08
34	Uncharacterized protein	ENO1	18	55.83	1.09E+08
35	Myosin light chain 1/3, skeletal muscle isoform	MYL1	16	78.30	1.07E+08
36	Annexin	ANXA5	24	74.60	1.06E+08
37	Glutathione S-transferase	GSTP1	11	61.23	1.03E+08
38	Filamin-A	FLNA	116	56.27	1.03E+08
