# Supplementary material for: First insight into the proteome landscape of the porcine short posterior ciliary arteries: Key signalling pathways maintaining physiologic functions
Source: Sci Rep. 2016 Dec 6;6:38298. doi: 10.1038/srep38298 (PMC5138843; doi:10.1038/srep38298)
Supplement: Supplementary Data [file srep38298-s1.pdf]

# SUPPLEMENTARY DATA

## First insight into the proteome landscape of the porcine short posterior ciliary arteries: Key signalling pathways maintaining physiologic functions

Caroline Manicam*, Natarajan Perumal, Norbert Pfeiffer, Franz H. Grus and Adrian Gericke

Department of Ophthalmology, University Medical Centre of the Johannes Gutenberg University Mainz, Mainz, Germany.

***Corresponding author:**

Dr. Caroline Manicam

Department of Ophthalmology,

University Medical Centre of the Johannes Gutenberg

University Mainz, Langenbeckstr. 1, 55131 Mainz, Germany.

E-mail: cmjc_82@yahoo.com

Tel: +49(0) 6131 17 3330

**Supplementary Table S1:**
**Complete list of the proteins identified from the porcine sPCA employing discovery proteomics strategy.**

| No. | Protein ID | Protein Name | Gene Name | Intensity | Intensity_R1 | Intensity_R2 | Intensity_R3 | iBAQ | iBAQ_R1 | iBAQ_R2 | iBAQ_R3 |
|---|---|---|---|---|---|---|---|---|---|---|---|
| 1 | F1RII7 | Hemoglobin subunit beta | HBB | 4.50E+10 | 1.79E+10 | 1.02E+10 | 1.68E+10 | 4.09E+09 | 1.63E+09 | 9.30E+08 | 1.53E+09 |
| 2 | P01965 | Hemoglobin subunit alpha | HBA | 2.70E+10 | 9.95E+09 | 6.73E+09 | 1.04E+10 | 3.00E+09 | 1.11E+09 | 7.48E+08 | 1.15E+09 |
| 3 | P00761 | Uncharacterized protein | LOC100302368 | 3.18E+10 | 1.59E+10 | 6.85E+09 | 8.99E+09 | 2.65E+09 | 1.33E+09 | 5.71E+08 | 7.49E+08 |
| 4 | B6VNT8 | Cardiac muscle alpha actin 1 | ACTC1 | 5.14E+10 | 1.73E+10 | 1.05E+10 | 2.36E+10 | 2.23E+09 | 7.54E+08 | 4.56E+08 | 1.02E+09 |
| 5 | F1RUN2 | Serum albumin | ALB | 8.80E+10 | 2.30E+10 | 2.55E+10 | 3.95E+10 | 1.96E+09 | 5.10E+08 | 5.67E+08 | 8.79E+08 |
| 6 | P01846 | Ig lambda chain C region |  | 3.46E+09 | 1.31E+09 | 9.25E+08 | 1.23E+09 | 5.77E+08 | 2.18E+08 | 1.54E+08 | 2.05E+08 |
| 7 | F1SQ09 | Uncharacterized protein | LUM | 8.77E+09 | 2.47E+09 | 2.48E+09 | 3.82E+09 | 5.16E+08 | 1.45E+08 | 1.46E+08 | 2.25E+08 |
| 8 | F1SJS8 | Transgelin | TAGLN | 8.13E+09 | 3.10E+09 | 1.74E+09 | 3.29E+09 | 4.78E+08 | 1.82E+08 | 1.03E+08 | 1.94E+08 |
| 9 | F1STC5 | Uncharacterized protein (Fragment) |  | 2.03E+09 | 6.57E+08 | 5.21E+08 | 8.55E+08 | 4.06E+08 | 1.31E+08 | 1.04E+08 | 1.71E+08 |
| 10 | Q86W17 | Protease serine 1 (Fragment) | PRSS1 | 2.24E+09 | 1.13E+09 | 6.49E+08 | 4.69E+08 | 3.74E+08 | 1.88E+08 | 1.08E+08 | 7.82E+07 |
| 11 | I3LVD5 | Actin, cytoplasmic 2 | ACTG1 | 8.34E+09 | 2.92E+09 | 1.51E+09 | 3.92E+09 | 3.63E+08 | 1.27E+08 | 6.55E+07 | 1.70E+08 |
| 12 | Q9XSD9 | Decorin | DCN | 6.53E+09 | 1.92E+09 | 1.68E+09 | 2.93E+09 | 3.11E+08 | 9.12E+07 | 8.02E+07 | 1.39E+08 |
| 13 | L8B0W0 | IgG heavy chain | IGHG | 5.85E+09 | 1.88E+09 | 1.15E+09 | 2.82E+09 | 2.66E+08 | 8.56E+07 | 5.24E+07 | 1.28E+08 |
| 14 | L8B173 | IgG heavy chain | IGHG | 5.83E+09 | 1.61E+09 | 1.67E+09 | 2.55E+09 | 2.33E+08 | 6.43E+07 | 6.69E+07 | 1.02E+08 |
| 15 | F1SKM9 | Galectin | LGALS1 | 2.17E+09 | 7.39E+08 | 8.03E+08 | 6.31E+08 | 2.17E+08 | 7.39E+07 | 8.03E+07 | 6.31E+07 |
| 16 | P09571 | Serotransferrin | TF | 9.91E+09 | 3.48E+09 | 2.79E+09 | 3.64E+09 | 2.16E+08 | 7.57E+07 | 6.06E+07 | 7.92E+07 |
| 17 | Q6ZN40 | Tropomyosin 1 (Alpha), isoform CRA | TPM1 | 5.47E+09 | 2.51E+09 | 1.30E+09 | 1.66E+09 | 2.03E+08 | 9.28E+07 | 4.80E+07 | 6.17E+07 |
| 18 | A0A0H5ANC0 | Osteoglycin/ mimecan | OGN | 3.72E+09 | 1.47E+09 | 8.54E+08 | 1.39E+09 | 1.86E+08 | 7.35E+07 | 4.27E+07 | 6.96E+07 |
| 19 | P02543 | Vimentin | VIM | 6.20E+09 | 2.11E+09 | 1.50E+09 | 2.59E+09 | 1.77E+08 | 6.04E+07 | 4.29E+07 | 7.40E+07 |
| 20 | P79260 | S-arrestin | SAG | 3.85E+09 | 1.02E+09 | 9.12E+08 | 1.92E+09 | 1.67E+08 | 4.42E+07 | 3.97E+07 | 8.36E+07 |
| 21 | A0A0S2Z4H2 | Protein S100-A1 | S100A1 | 4.89E+08 | 1.53E+08 | 1.80E+08 | 1.56E+08 | 1.63E+08 | 5.09E+07 | 6.01E+07 | 5.20E+07 |
| 22 | F1RXC2 | Uncharacterized protein | CA2 | 2.71E+09 | 1.15E+09 | 7.75E+08 | 7.87E+08 | 1.59E+08 | 6.74E+07 | 4.56E+07 | 4.63E+07 |
| 23 | I3LPB5 | Creatine kinase B-type | CKB | 2.10E+09 | 9.48E+08 | 3.65E+08 | 7.86E+08 | 1.50E+08 | 6.77E+07 | 2.61E+07 | 5.62E+07 |
| 24 | P00355 | Glyceraldehyde-3-phosphate dehydrogenase | GAPDH | 3.09E+09 | 1.31E+09 | 8.15E+08 | 9.70E+08 | 1.47E+08 | 6.23E+07 | 3.88E+07 | 4.62E+07 |
| 25 | I3L5R6 | Protein S100 | S100B | 5.65E+08 | 1.91E+08 | 1.48E+08 | 2.26E+08 | 1.41E+08 | 4.78E+07 | 3.69E+07 | 5.65E+07 |
| 26 | D0G7F6 | Triosephosphate isomerase | TPI1 | 2.20E+09 | 7.84E+08 | 6.40E+08 | 7.73E+08 | 1.29E+08 | 4.61E+07 | 3.77E+07 | 4.55E+07 |
| 27 | D2JYH4 | Actin alpha 2 | ACTA2 | 2.92E+09 | 1.23E+09 | 6.02E+08 | 1.08E+09 | 1.27E+08 | 5.37E+07 | 2.62E+07 | 4.70E+07 |
| 28 | F1RKG8 | Phosphatidylethanolamine-binding protein 1 | PEBP1 | 1.77E+09 | 6.66E+08 | 4.04E+08 | 7.00E+08 | 1.26E+08 | 4.76E+07 | 2.88E+07 | 5.00E+07 |
| 29 | I3LL97 | Cysteine and glycine-rich protein 1 | CSRP1 | 1.86E+09 | 7.49E+08 | 5.03E+08 | 6.04E+08 | 1.24E+08 | 4.99E+07 | 3.35E+07 | 4.02E+07 |
| 30 | B4DGK4 | Synaptotagmin-7 | SYT7 | 4.93E+08 | 3.15E+08 | 2.65E+07 | 1.51E+08 | 1.23E+08 | 7.88E+07 | 6.63E+06 | 3.78E+07 |
| 31 | P19620 | Annexin A2 | ANXA2 | 3.07E+09 | 1.36E+09 | 8.41E+08 | 8.70E+08 | 1.18E+08 | 5.24E+07 | 3.23E+07 | 3.35E+07 |
| 32 | F1SG00 | Uncharacterized protein | TPM2 | 2.66E+09 | 9.69E+08 | 5.88E+08 | 1.10E+09 | 1.16E+08 | 4.21E+07 | 2.55E+07 | 4.80E+07 |
| 33 | Q5XLD3 | Creatine kinase M-type | CKM | 2.79E+09 | 1.14E+09 | 5.76E+08 | 1.08E+09 | 1.12E+08 | 4.54E+07 | 2.30E+07 | 4.31E+07 |
| 34 | I3LK59 | Uncharacterized protein | ENO1 | 2.63E+09 | 7.50E+08 | 7.24E+08 | 1.15E+09 | 1.09E+08 | 3.13E+07 | 3.02E+07 | 4.80E+07 |
| 35 | A1XQT6 | Myosin light chain 1/3, skeletal muscle isoform | MYL1 | 1.29E+09 | 3.01E+08 | 3.39E+08 | 6.48E+08 | 1.07E+08 | 2.51E+07 | 2.82E+07 | 5.40E+07 |
| 36 | F2Z5C1 | Annexin | ANXA5 | 2.44E+09 | 7.32E+08 | 6.52E+08 | 1.05E+09 | 1.06E+08 | 3.18E+07 | 2.83E+07 | 4.57E+07 |
| 37 | P80031 | Glutathione S-transferase P | GSTP1 | 1.24E+09 | 4.09E+08 | 3.01E+08 | 5.31E+08 | 1.03E+08 | 3.41E+07 | 2.51E+07 | 4.43E+07 |
| 38 | Q5HY54 | Filamin-A | FLNA | 1.56E+10 | 6.67E+09 | 4.17E+09 | 4.71E+09 | 1.03E+08 | 4.42E+07 | 2.76E+07 | 3.12E+07 |
| 39 | F1RI39 | Alpha-actinin-4 | ACTN4 | 6.17E+09 | 2.46E+09 | 1.50E+09 | 2.21E+09 | 1.01E+08 | 4.04E+07 | 2.46E+07 | 3.62E+07 |
| 40 | Q29568 | Phosphopyruvate hydratase |  | 9.87E+08 | 2.46E+08 | 2.62E+08 | 4.79E+08 | 9.87E+07 | 2.46E+07 | 2.62E+07 | 4.79E+07 |
| 41 | K7GP55 | Biglycan | BGN | 1.86E+09 | 7.64E+08 | 4.58E+08 | 6.36E+08 | 9.78E+07 | 4.02E+07 | 2.41E+07 | 3.35E+07 |
| 42 | L8ECP2 | Alternative protein PTPDC1 | PTPDC1 | 3.61E+08 | 1.95E+08 | 1.00E+08 | 6.61E+07 | 9.03E+07 | 4.87E+07 | 2.51E+07 | 1.65E+07 |
| 43 | Q08092 | Calponin-1 | CNN1 | 1.59E+09 | 7.71E+08 | 3.27E+08 | 4.95E+08 | 8.85E+07 | 4.28E+07 | 1.82E+07 | 2.75E+07 |
| 44 | V9HWH2 | Creatine kinase brain isoform 1 | HEL-S-29 | 2.00E+09 | 7.56E+08 | 4.22E+08 | 8.25E+08 | 8.71E+07 | 3.29E+07 | 1.84E+07 | 3.59E+07 |
| 45 | F8W1R7 | Myosin light polypeptide 6 | MYL6 | 9.57E+08 | 3.24E+08 | 2.06E+08 | 4.27E+08 | 8.70E+07 | 2.94E+07 | 1.88E+07 | 3.88E+07 |
| 46 | Q5XLD2 | Myosin regulatory light chain 2 | HUMMLC2B | 1.04E+09 | 3.07E+08 | 2.71E+08 | 4.65E+08 | 8.70E+07 | 2.56E+07 | 2.26E+07 | 3.87E+07 |
| 47 | A0A0C3SG01 | Apolipoprotein A-I | APOA1 | 1.66E+09 | 5.77E+08 | 4.04E+08 | 6.83E+08 | 8.32E+07 | 2.88E+07 | 2.02E+07 | 3.41E+07 |
| 48 | F1SFI7 | Alpha-2-HS-glycoprotein | AHSG | 1.34E+09 | 3.40E+08 | 3.40E+08 | 6.55E+08 | 7.85E+07 | 2.00E+07 | 2.00E+07 | 3.85E+07 |
| 49 | Q3SX09 | Hemoglobin subunit beta |  | 8.58E+08 | 4.85E+08 | 1.68E+08 | 2.04E+08 | 7.80E+07 | 4.41E+07 | 1.53E+07 | 1.86E+07 |
| 50 | L8B0U3 | IgG heavy chain | IGHG | 1.58E+09 | 5.08E+08 | 3.92E+08 | 6.83E+08 | 7.54E+07 | 2.42E+07 | 1.87E+07 | 3.25E+07 |
| 51 | F1STC2 | Uncharacterized protein (Fragment) |  | 3.74E+08 | 1.20E+08 | 1.11E+08 | 1.42E+08 | 7.47E+07 | 2.41E+07 | 2.22E+07 | 2.84E+07 |
| 52 | V9HW98 | Epididymis luminal protein 2 | HEL2 | 1.31E+09 | 5.07E+08 | 3.19E+08 | 4.88E+08 | 7.30E+07 | 2.82E+07 | 1.77E+07 | 2.71E+07 |
| 53 | F1SHL9 | Pyruvate kinase | PKM | 2.83E+09 | 1.09E+09 | 8.09E+08 | 9.30E+08 | 7.25E+07 | 2.79E+07 | 2.07E+07 | 2.38E+07 |
| 54 | P62936 | Peptidyl-prolyl cis-trans isomerase A | PPIA | 1.01E+09 | 4.97E+08 | 1.86E+08 | 3.30E+08 | 7.23E+07 | 3.55E+07 | 1.33E+07 | 2.36E+07 |
| 55 | D0G6X5 | Glutamine synthetase | GLUL | 1.33E+09 | 4.92E+08 | 2.86E+08 | 5.48E+08 | 6.98E+07 | 2.59E+07 | 1.51E+07 | 2.88E+07 |
| 56 | F1SDX9 | Peroxiredoxin 2 | PRDX2 | 9.71E+08 | 2.92E+08 | 2.31E+08 | 4.49E+08 | 6.93E+07 | 2.08E+07 | 1.65E+07 | 3.20E+07 |
| 57 | F1SCD0 | Uncharacterized protein | LOC396685 | 1.71E+09 | 5.04E+08 | 4.87E+08 | 7.22E+08 | 6.59E+07 | 1.94E+07 | 1.87E+07 | 2.78E+07 |
| 58 | Q8SPS7 | Haptoglobin | HP | 1.51E+09 | 6.08E+08 | 3.61E+08 | 5.43E+08 | 6.58E+07 | 2.65E+07 | 1.57E+07 | 2.36E+07 |
| 59 | Q1KYT0 | Beta-enolase | ENO3 | 2.04E+09 | 6.07E+08 | 5.54E+08 | 8.76E+08 | 6.57E+07 | 1.96E+07 | 1.79E+07 | 2.83E+07 |
| 60 | F1RFY1 | Profilin | PFN1 | 5.80E+08 | 2.66E+08 | 2.12E+08 | 1.02E+08 | 6.44E+07 | 2.95E+07 | 2.36E+07 | 1.13E+07 |
| 61 | F6KPG5 | Albumin (Fragment) | ALB | 2.69E+09 | 7.20E+08 | 9.33E+08 | 1.04E+09 | 6.41E+07 | 1.71E+07 | 2.22E+07 | 2.47E+07 |
| 62 | P50828 | Hemopexin | HPX | 1.63E+09 | 4.88E+08 | 4.81E+08 | 6.62E+08 | 6.27E+07 | 1.88E+07 | 1.85E+07 | 2.55E+07 |
| 63 | A0A075B7H9 | Uncharacterized protein (Fragment) |  | 2.49E+08 | 7.52E+07 | 6.26E+07 | 1.11E+08 | 6.21E+07 | 1.88E+07 | 1.57E+07 | 2.77E+07 |
| 64 | L0R5C7 | Alternative protein SCP2 | SCP2 | 2.45E+08 | 7.98E+07 | 6.69E+07 | 9.79E+07 | 6.12E+07 | 2.00E+07 | 1.67E+07 | 2.45E+07 |
| 65 | L8B0R9 | IgG heavy chain | IGHG | 1.22E+09 | 2.97E+08 | 3.33E+08 | 5.88E+08 | 6.09E+07 | 1.48E+07 | 1.67E+07 | 2.94E+07 |
| 66 | J3QS39 | Polyubiquitin-B (Fragment) | UBB | 5.29E+08 | 1.80E+08 | 1.25E+08 | 2.23E+08 | 5.88E+07 | 2.00E+07 | 1.39E+07 | 2.48E+07 |
| 67 | F1S6B4 | Uncharacterized protein | PRELP | 1.44E+09 | 5.26E+08 | 3.28E+08 | 5.87E+08 | 5.76E+07 | 2.10E+07 | 1.31E+07 | 2.35E+07 |
| 68 | V9HWN7 | Fructose-bisphosphate aldolase | HEL-S-87p | 1.35E+09 | 5.80E+08 | 2.41E+08 | 5.28E+08 | 5.62E+07 | 2.42E+07 | 1.01E+07 | 2.20E+07 |
| 69 | P10668 | Cofilin-1 | CFL1 | 6.46E+08 | 2.48E+08 | 1.52E+08 | 2.45E+08 | 5.38E+07 | 2.07E+07 | 1.27E+07 | 2.04E+07 |
| 70 | P20305 | Gelsolin (Fragment) | GSN | 2.19E+09 | 7.52E+08 | 5.07E+08 | 9.32E+08 | 5.34E+07 | 1.83E+07 | 1.24E+07 | 2.27E+07 |
| 71 | F1SM43 | S-arrestin | SAG | 4.56E+08 | 1.26E+08 | 1.06E+08 | 2.25E+08 | 5.07E+07 | 1.40E+07 | 1.18E+07 | 2.50E+07 |
| 72 | K7GL43 | Uncharacterized protein (Fragment) | THY1 | 3.95E+08 | 1.55E+08 | 1.08E+08 | 1.32E+08 | 4.94E+07 | 1.93E+07 | 1.35E+07 | 1.66E+07 |
| 73 | F1SLA0 | ATP synthase subunit beta | ATP5B | 1.47E+09 | 3.70E+08 | 3.61E+08 | 7.41E+08 | 4.91E+07 | 1.23E+07 | 1.20E+07 | 2.47E+07 |
| 74 | Q6FHU2 | Epididymis secretory protein Li 35 (Fragment) | PGAM1 | 7.36E+08 | 3.06E+08 | 2.52E+08 | 1.78E+08 | 4.91E+07 | 2.04E+07 | 1.68E+07 | 1.19E+07 |
| 75 | D0PNI1 | Epididymis luminal protein 4 | YWHAZ | 9.30E+08 | 3.80E+08 | 2.52E+08 | 2.98E+08 | 4.90E+07 | 2.00E+07 | 1.33E+07 | 1.57E+07 |
| 76 | F1S1G8 | Amine oxidase | AOC3 | 1.55E+09 | 6.15E+08 | 4.00E+08 | 5.39E+08 | 4.86E+07 | 1.92E+07 | 1.25E+07 | 1.69E+07 |
| 77 | F2Z5B6 | Tropomyosin alpha-1 chain | TPM1 | 1.03E+09 | 5.27E+08 | 2.51E+08 | 2.51E+08 | 4.47E+07 | 2.29E+07 | 1.09E+07 | 1.09E+07 |
| 78 | Q5S1U1 | Heat shock protein beta-1 | HSPB1 | 5.77E+08 | 2.04E+08 | 1.47E+08 | 2.26E+08 | 4.44E+07 | 1.57E+07 | 1.13E+07 | 1.74E+07 |
| 79 | Q7SIB7 | Phosphoglycerate kinase 1 | PGK1 | 1.40E+09 | 4.58E+08 | 2.80E+08 | 6.60E+08 | 4.37E+07 | 1.43E+07 | 8.75E+06 | 2.06E+07 |
| 80 | Q562R1 | Beta-actin-like protein 2 | ACTBL2 | 1.02E+09 | 3.65E+08 | 1.72E+08 | 4.84E+08 | 4.25E+07 | 1.52E+07 | 7.17E+06 | 2.02E+07 |
| 81 | A0A024R694 | Actinin, alpha 1, isoform CRA | ACTN1 | 2.44E+09 | 1.01E+09 | 5.87E+08 | 8.43E+08 | 4.21E+07 | 1.74E+07 | 1.01E+07 | 1.45E+07 |
| 82 | F1RJ25 | Fructose-bisphosphate aldolase | ALDOC | 1.00E+09 | 3.36E+08 | 2.05E+08 | 4.62E+08 | 4.18E+07 | 1.40E+07 | 8.54E+06 | 1.92E+07 |
| 83 | P00346 | Malate dehydrogenase, mitochondrial | MDH2 | 9.83E+08 | 4.55E+08 | 1.88E+08 | 3.40E+08 | 4.10E+07 | 1.90E+07 | 7.83E+06 | 1.42E+07 |
| 84 | Q0VAS5 | Histone H4 | HIST1H4H | 2.35E+08 | 1.47E+08 | 5.73E+07 | 3.05E+07 | 3.91E+07 | 2.45E+07 | 9.55E+06 | 5.09E+06 |
| 85 | Q9GMA6 | Alpha-1-antichymotrypsin 2 | SERPINA3-2 | 1.02E+09 | 2.77E+08 | 3.46E+08 | 3.99E+08 | 3.78E+07 | 1.03E+07 | 1.28E+07 | 1.48E+07 |
| 86 | V9HW22 | Epididymis luminal protein 33 | HEL-S-72p | 1.35E+09 | 3.62E+08 | 3.69E+08 | 6.15E+08 | 3.64E+07 | 9.77E+06 | 9.97E+06 | 1.66E+07 |
| 87 | F1S1B2 | Uncharacterized protein | MPZ | 5.08E+08 | 2.83E+08 | 1.11E+08 | 1.14E+08 | 3.63E+07 | 2.02E+07 | 7.94E+06 | 8.14E+06 |
| 88 | F1SJB5 | Annexin | ANXA1 | 7.90E+08 | 3.25E+08 | 1.96E+08 | 2.69E+08 | 3.59E+07 | 1.48E+07 | 8.90E+06 | 1.22E+07 |
| 89 | G9F6X9 | Dihydropyrimidinase-like 2 | DPYSL2 | 1.21E+09 | 4.03E+08 | 3.69E+08 | 4.37E+08 | 3.56E+07 | 1.18E+07 | 1.09E+07 | 1.29E+07 |
| 90 | B4DPZ5 | cDNA FLJ53495, highly similar to Polymerase I and transcript release factor |  | 6.01E+08 | 2.29E+08 | 1.60E+08 | 2.13E+08 | 3.54E+07 | 1.34E+07 | 9.39E+06 | 1.25E+07 |
| 91 | F1RFH9 | Calcium-transporting ATPase | ATP2A1 | 1.74E+09 | 8.23E+08 | 4.17E+08 | 4.97E+08 | 3.41E+07 | 1.61E+07 | 8.19E+06 | 9.74E+06 |
| 92 | P82460 | Thioredoxin | TXN | 2.37E+08 | 6.88E+07 | 8.97E+07 | 7.86E+07 | 3.39E+07 | 9.83E+06 | 1.28E+07 | 1.12E+07 |
| 93 | Q53SB5 | Desmin, isoform CRA | tmp_locus_29 | 1.20E+09 | 5.02E+08 | 3.11E+08 | 3.90E+08 | 3.34E+07 | 1.39E+07 | 8.64E+06 | 1.08E+07 |
| 94 | D0G778 | Macrophage migration inhibitory factor (Fragment) | MIF | 1.67E+08 | 5.21E+07 | 5.61E+07 | 5.83E+07 | 3.33E+07 | 1.04E+07 | 1.12E+07 | 1.17E+07 |
| 95 | P08059 | Glucose-6-phosphate isomerase | GPI | 1.15E+09 | 3.34E+08 | 3.93E+08 | 4.28E+08 | 3.30E+07 | 9.53E+06 | 1.12E+07 | 1.22E+07 |
| 96 | K7ZRK0 | IgA heavy chian constant region (Fragment) | IGHA | 6.20E+08 | 1.92E+08 | 1.58E+08 | 2.70E+08 | 3.26E+07 | 1.01E+07 | 8.31E+06 | 1.42E+07 |
| 97 | Q5T7C4 | High mobility group protein B1 | HMGB1 | 2.58E+08 | 8.80E+07 | 8.04E+07 | 8.94E+07 | 3.22E+07 | 1.10E+07 | 1.00E+07 | 1.12E+07 |
| 98 | F2Z5I9 | Destrin | DSTN | 4.12E+08 | 1.85E+08 | 8.30E+07 | 1.43E+08 | 3.17E+07 | 1.43E+07 | 6.38E+06 | 1.10E+07 |
| 99 | Q0R678 | DJ-1 protein | PARK7 | 3.96E+08 | 1.15E+08 | 1.05E+08 | 1.76E+08 | 3.05E+07 | 8.84E+06 | 8.05E+06 | 1.36E+07 |
| 100 | R4H1Z8 | Adipocyte fatty acid-binding protein | A-FABP | 3.57E+08 | 1.78E+08 | 8.65E+07 | 9.29E+07 | 2.98E+07 | 1.48E+07 | 7.21E+06 | 7.74E+06 |
| 101 | P22626 | Heterogeneous nuclear ribonucleoproteins A2/B1 | HNRNPA2B1 | 5.95E+08 | 2.33E+08 | 1.53E+08 | 2.09E+08 | 2.97E+07 | 1.17E+07 | 7.65E+06 | 1.04E+07 |
| 102 | A0A0S2Z4I4 | Tropomyosin 3 isoform 3 (Fragment) | TPM3 | 6.18E+08 | 2.87E+08 | 1.51E+08 | 1.80E+08 | 2.94E+07 | 1.37E+07 | 7.18E+06 | 8.55E+06 |
| 103 | P26234 | Vinculin | VCL | 2.50E+09 | 1.21E+09 | 5.66E+08 | 7.26E+08 | 2.87E+07 | 1.39E+07 | 6.51E+06 | 8.34E+06 |
| 104 | Q6RVA9 | Caveolin-1 | CAV1 | 3.04E+08 | 2.45E+08 | 4.13E+07 | 1.74E+07 | 2.76E+07 | 2.23E+07 | 3.76E+06 | 1.59E+06 |
| 105 | K7GS06 | Uncharacterized protein | FHL1 | 4.91E+08 | 2.66E+08 | 9.00E+07 | 1.35E+08 | 2.73E+07 | 1.48E+07 | 5.00E+06 | 7.50E+06 |
| 106 | P11708 | Malate dehydrogenase, cytoplasmic | MDH1 | 5.87E+08 | 2.65E+08 | 1.16E+08 | 2.06E+08 | 2.67E+07 | 1.21E+07 | 5.25E+06 | 9.36E+06 |
| 107 | A0A075B7I6 | Uncharacterized protein (Fragment) |  | 1.60E+08 | 6.82E+07 | 4.08E+07 | 5.05E+07 | 2.66E+07 | 1.14E+07 | 6.81E+06 | 8.41E+06 |
| 108 | P28491 | Calreticulin | CALR | 7.06E+08 | 1.47E+08 | 1.89E+08 | 3.71E+08 | 2.52E+07 | 5.25E+06 | 6.74E+06 | 1.32E+07 |
| 109 | Q95ME5 | Superoxide dismutase 1 (Fragment) | SOD1 | 2.50E+08 | 1.03E+08 | 3.22E+07 | 1.15E+08 | 2.50E+07 | 1.03E+07 | 3.22E+06 | 1.15E+07 |
| 110 | K7GMM3 | Protein S100-A10 | S100A10 | 1.23E+08 | 3.84E+07 | 3.90E+07 | 4.54E+07 | 2.46E+07 | 7.68E+06 | 7.81E+06 | 9.08E+06 |
| 111 | H6VRG3 | Keratin 1 | KRT1 | 8.55E+08 | 2.88E+08 | 2.01E+08 | 3.66E+08 | 2.44E+07 | 8.24E+06 | 5.75E+06 | 1.05E+07 |
| 112 | I3L706 | Uncharacterized protein | RCVRN | 4.08E+08 | 1.10E+08 | 1.03E+08 | 1.95E+08 | 2.40E+07 | 6.48E+06 | 6.04E+06 | 1.15E+07 |
| 113 | I3LDM7 | Lactoylglutathione lyase | GLO1 | 2.61E+08 | 9.25E+07 | 5.96E+07 | 1.09E+08 | 2.38E+07 | 8.41E+06 | 5.42E+06 | 9.93E+06 |
| 114 | A0A024R4F1 | Enolase 1, (Alpha), isoform CRA | ENO1 | 6.89E+08 | 3.09E+08 | 1.51E+08 | 2.28E+08 | 2.38E+07 | 1.07E+07 | 5.22E+06 | 7.86E+06 |
| 115 | Q6EEI0 | Vimentin (Fragment) |  | 9.43E+07 | 3.02E+07 | 4.07E+07 | 2.34E+07 | 2.36E+07 | 7.56E+06 | 1.02E+07 | 5.84E+06 |
| 116 | O02705 | Heat shock protein HSP 90-alpha | HSP90AA1 | 8.88E+08 | 3.50E+08 | 2.27E+08 | 3.11E+08 | 2.34E+07 | 9.21E+06 | 5.97E+06 | 8.17E+06 |
| 117 | K7ZJP7 | IgM heavy chain constant region (Fragment) | IGHM | 5.96E+08 | 1.86E+08 | 1.55E+08 | 2.55E+08 | 2.29E+07 | 7.14E+06 | 5.97E+06 | 9.80E+06 |
| 118 | F1RXC1 | Uncharacterized protein | CA1 | 3.40E+08 | 1.37E+08 | 1.04E+08 | 9.97E+07 | 2.27E+07 | 9.11E+06 | 6.93E+06 | 6.64E+06 |
| 119 | P83686 | NADH-cytochrome b5 reductase 3 (Fragment) | CYB5R3 | 3.85E+08 | 2.61E+08 | 6.33E+07 | 6.10E+07 | 2.27E+07 | 1.54E+07 | 3.73E+06 | 3.59E+06 |
| 120 | K9IVR7 | WD repeat-containing protein 1 | WDR1 | 7.93E+08 | 2.60E+08 | 2.53E+08 | 2.81E+08 | 2.27E+07 | 7.42E+06 | 7.22E+06 | 8.02E+06 |
| 121 | P50390 | Transthyretin | TTR | 2.22E+08 | 8.95E+07 | 7.86E+07 | 5.38E+07 | 2.22E+07 | 8.95E+06 | 7.86E+06 | 5.38E+06 |
| 122 | E7EI20 | Rho GDP dissociation inhibitor alpha | ARHGDIA | 3.31E+08 | 1.17E+08 | 1.09E+08 | 1.06E+08 | 2.21E+07 | 7.78E+06 | 7.26E+06 | 7.04E+06 |
| 123 | M3VH45 | Annexin A6 tv1 | ANXA6 | 1.14E+09 | 3.29E+08 | 3.25E+08 | 4.81E+08 | 2.18E+07 | 6.33E+06 | 6.25E+06 | 9.24E+06 |
| 124 | Q9GJX2 | Diazepam binding inhibitor (Fragment) | DBI | 1.29E+08 | 3.42E+07 | 4.18E+07 | 5.28E+07 | 2.15E+07 | 5.70E+06 | 6.97E+06 | 8.80E+06 |
| 125 | B2R6K4 | cDNA, FLJ92996, highly similar to Homo sapiens guanine nucleotide binding protein (G protein), beta polypeptide 1 (GNB1), mRNA |  | 2.74E+08 | 1.43E+08 | 6.04E+07 | 7.08E+07 | 2.11E+07 | 1.10E+07 | 4.65E+06 | 5.44E+06 |
| 126 | I3LCN1 | Uncharacterized protein | ENO2 | 5.45E+08 | 1.67E+08 | 1.13E+08 | 2.64E+08 | 2.10E+07 | 6.43E+06 | 4.36E+06 | 1.02E+07 |
| 127 | F1SDR7 | Uncharacterized protein | YWHAB | 3.45E+08 | 1.33E+08 | 1.17E+08 | 9.47E+07 | 2.03E+07 | 7.85E+06 | 6.87E+06 | 5.57E+06 |
| 128 | B3KT06 | cDNA FLJ37398 fis, clone BRAMY2027467, highly similar to Tubulin alpha-ubiquitous chain |  | 4.57E+08 | 1.82E+08 | 1.05E+08 | 1.70E+08 | 1.91E+07 | 7.57E+06 | 4.39E+06 | 7.10E+06 |
| 129 | C4MXZ1 | ADP-ribosylation factor 1 | ARF1 | 2.05E+08 | 9.83E+07 | 4.15E+07 | 6.54E+07 | 1.87E+07 | 8.94E+06 | 3.77E+06 | 5.95E+06 |
| 130 | Q2VTP6 | Peptidyl-prolyl cis-trans isomerase | FKBP1A | 5.44E+07 | 1.07E+07 | 2.44E+07 | 1.94E+07 | 1.81E+07 | 3.55E+06 | 8.14E+06 | 6.45E+06 |
| 131 | P00336 | L-lactate dehydrogenase B chain | LDHB | 3.53E+08 | 1.64E+08 | 8.72E+07 | 1.01E+08 | 1.76E+07 | 8.21E+06 | 4.36E+06 | 5.06E+06 |
| 132 | Q75NG6 | Troponin T fast skeletal muscle type | tnnt3 | 2.44E+08 | 8.82E+07 | 5.97E+07 | 9.63E+07 | 1.74E+07 | 6.30E+06 | 4.26E+06 | 6.88E+06 |
| 133 | I3LE39 | Histone H2B | HIST2H2BE | 1.92E+08 | 6.98E+07 | 5.78E+07 | 6.41E+07 | 1.74E+07 | 6.34E+06 | 5.26E+06 | 5.83E+06 |
| 134 | O62680 | CD59 glycoprotein | CD59 | 6.86E+07 | 3.72E+07 | 6.23E+06 | 2.52E+07 | 1.71E+07 | 9.29E+06 | 1.56E+06 | 6.30E+06 |
| 135 | P62895 | Cytochrome c | CYCS | 1.36E+08 | 5.75E+07 | 4.83E+07 | 3.03E+07 | 1.70E+07 | 7.19E+06 | 6.03E+06 | 3.79E+06 |
| 136 | F1RPS8 | ATP synthase subunit alpha | ATP5A1 | 5.66E+08 | 1.79E+08 | 1.39E+08 | 2.48E+08 | 1.67E+07 | 5.26E+06 | 4.09E+06 | 7.30E+06 |
| 137 | A3KPC7 | Histone H2A | HIST1H2AH | 1.09E+08 | 4.13E+07 | 3.74E+07 | 3.00E+07 | 1.55E+07 | 5.89E+06 | 5.35E+06 | 4.28E+06 |
| 138 | F1RS36 | 78 kDa glucose-regulated protein | HSPA5 | 5.68E+08 | 1.70E+08 | 1.61E+08 | 2.37E+08 | 1.54E+07 | 4.60E+06 | 4.35E+06 | 6.41E+06 |
| 139 | A0A075B7I5 | Uncharacterized protein (Fragment) |  | 4.54E+07 | 1.67E+07 | 1.16E+07 | 1.71E+07 | 1.51E+07 | 5.57E+06 | 3.86E+06 | 5.72E+06 |
| 140 | K9J6H8 | Alpha-2-macroglobulin | A2M | 1.26E+09 | 5.50E+08 | 3.31E+08 | 3.77E+08 | 1.50E+07 | 6.55E+06 | 3.94E+06 | 4.49E+06 |
| 141 | F2Z5P1 | Histone H2A | LOC100512448 | 8.96E+07 | 3.69E+07 | 2.72E+07 | 2.56E+07 | 1.49E+07 | 6.15E+06 | 4.53E+06 | 4.26E+06 |
| 142 | F1S6B5 | Fibromodulin | FMOD | 1.91E+08 | 4.42E+07 | 5.84E+07 | 8.79E+07 | 1.47E+07 | 3.40E+06 | 4.49E+06 | 6.76E+06 |
| 143 | B5KJG2 | Phosphoglycerate mutase 2 | PGAM2 | 2.46E+08 | 9.27E+07 | 7.81E+07 | 7.51E+07 | 1.45E+07 | 5.46E+06 | 4.59E+06 | 4.42E+06 |
| 144 | Q6UV40 | Fructose-bisphosphate aldolase A (Fragment) | ALDOA | 1.00E+08 | 3.41E+07 | 1.78E+07 | 4.82E+07 | 1.43E+07 | 4.87E+06 | 2.54E+06 | 6.88E+06 |

**Supplementary Table S1: (continued)**

| | | | | | | | | | | | |
|---|---|---|---|---|---|---|---|---|---|---|---|
| **145** | A0A024R5W6 | Tropomyosin 1 (Alpha), isoform CRA | TPM1 | 3.28E+08 | 1.65E+08 | 7.65E+07 | 8.66E+07 | 1.43E+07 | 7.18E+06 | 3.33E+06 | 3.77E+06 |
| **146** | F1RFQ7 | GTP-binding nuclear protein Ran | RAN | 1.68E+08 | 6.68E+07 | 5.11E+07 | 5.04E+07 | 1.40E+07 | 5.57E+06 | 4.26E+06 | 4.20E+06 |
| **147** | Q6IPT9 | Elongation factor 1-alpha | EEF1A1 | 3.51E+08 | 1.20E+08 | 8.31E+07 | 1.48E+08 | 1.40E+07 | 4.79E+06 | 3.32E+06 | 5.91E+06 |
| **148** | B1PSB6 | Adiponectin (Fragment) | ADIPOQ | 2.09E+08 | 8.33E+07 | 5.44E+07 | 7.15E+07 | 1.40E+07 | 5.56E+06 | 3.63E+06 | 4.77E+06 |
| **149** | I3LN42 | Uncharacterized protein | GC | 4.02E+08 | 1.25E+08 | 1.31E+08 | 1.46E+08 | 1.39E+07 | 4.29E+06 | 4.52E+06 | 5.05E+06 |
| **150** | F1SI77 | Uncharacterized protein | LOC100519994 | 3.67E+08 | 1.76E+08 | 8.06E+07 | 1.10E+08 | 1.36E+07 | 6.52E+06 | 2.98E+06 | 4.08E+06 |
| **151** | F1S814 | Uncharacterized protein | PGM1 | 5.13E+08 | 1.63E+08 | 1.46E+08 | 2.04E+08 | 1.35E+07 | 4.29E+06 | 3.85E+06 | 5.36E+06 |
| **152** | G9F6X8 | Protein disulfide-isomerase | | 4.78E+08 | 1.49E+08 | 1.60E+08 | 1.68E+08 | 1.33E+07 | 4.14E+06 | 4.44E+06 | 4.68E+06 |
| **153** | J3QRU4 | Vesicle-associated membrane protein 2 | VAMP2 | 6.62E+07 | 3.20E+07 | 1.10E+07 | 2.32E+07 | 1.32E+07 | 6.41E+06 | 2.19E+06 | 4.64E+06 |
| **154** | P13645 | Keratin, type I cytoskeletal 10 | KRT10 | 3.69E+08 | 1.35E+08 | 7.66E+07 | 1.57E+08 | 1.32E+07 | 4.82E+06 | 2.73E+06 | 5.61E+06 |
| **155** | F1S3E7 | Uncharacterized protein | CALB2 | 2.50E+08 | 8.74E+07 | 7.53E+07 | 8.73E+07 | 1.32E+07 | 4.60E+06 | 3.96E+06 | 4.59E+06 |
| **156** | E1CAJ5 | Protein disulfide-isomerase | grp-58 | 4.68E+08 | 1.74E+08 | 1.47E+08 | 1.47E+08 | 1.30E+07 | 4.82E+06 | 4.09E+06 | 4.08E+06 |
| **157** | Q3ZD69 | Prelamin-A/C | LMNA | 5.61E+08 | 1.74E+08 | 1.27E+08 | 2.60E+08 | 1.27E+07 | 3.96E+06 | 2.89E+06 | 5.90E+06 |
| **158** | Q8IZ29 | Tubulin, beta 2C | TUBB2C | 2.80E+08 | 1.21E+08 | 6.02E+07 | 9.95E+07 | 1.27E+07 | 5.48E+06 | 2.74E+06 | 4.52E+06 |
| **159** | F2Z4Z1 | Uncharacterized protein | YWHAG | 2.29E+08 | 9.51E+07 | 7.50E+07 | 5.86E+07 | 1.27E+07 | 5.28E+06 | 4.17E+06 | 3.26E+06 |
| **160** | P00339 | L-lactate dehydrogenase A chain | LDHA | 3.02E+08 | 1.35E+08 | 5.22E+07 | 1.15E+08 | 1.26E+07 | 5.61E+06 | 2.17E+06 | 4.79E+06 |
| **161** | Q9TSX9 | Peroxiredoxin-6 | PRDX6 | 2.01E+08 | 1.01E+08 | 3.34E+07 | 6.63E+07 | 1.26E+07 | 6.33E+06 | 2.09E+06 | 4.14E+06 |
| **162** | F1SC20 | Alpha-1B-glycoprotein | A1BG | 3.48E+08 | 7.69E+07 | 9.79E+07 | 1.73E+08 | 1.24E+07 | 2.75E+06 | 3.50E+06 | 6.17E+06 |
| **163** | Q6S4N2 | Heat shock 70 kDa protein 1B | HSPA1B | 4.43E+08 | 1.18E+08 | 1.33E+08 | 1.91E+08 | 1.23E+07 | 3.28E+06 | 3.70E+06 | 5.31E+06 |
| **164** | F1SIV3 | Uncharacterized protein | ANP32A | 1.35E+08 | 5.62E+07 | 3.24E+07 | 4.63E+07 | 1.23E+07 | 5.11E+06 | 2.94E+06 | 4.21E+06 |
| **165** | F1SB60 | Superoxide dismutase | SOD2 | 1.34E+08 | 4.55E+07 | 3.37E+07 | 5.51E+07 | 1.22E+07 | 4.13E+06 | 3.07E+06 | 5.01E+06 |
| **166** | P00503 | Aspartate aminotransferase, cytoplasmic | GOT1 | 3.26E+08 | 1.25E+08 | 7.52E+07 | 1.26E+08 | 1.21E+07 | 4.64E+06 | 2.78E+06 | 4.67E+06 |
| **167** | Q29290 | Cystatin-B | CSTB | 9.63E+07 | 2.01E+07 | 3.69E+07 | 3.93E+07 | 1.20E+07 | 2.51E+06 | 4.62E+06 | 4.91E+06 |
| **168** | F1SCC7 | Uncharacterized protein | LOC100156325 | 2.76E+08 | 8.17E+07 | 8.06E+07 | 1.14E+08 | 1.20E+07 | 3.55E+06 | 3.51E+06 | 4.96E+06 |
| **169** | V9HWC6 | Peptidyl-prolyl cis-trans isomerase | HEL-S-39 | 1.92E+08 | 5.51E+07 | 4.65E+07 | 9.06E+07 | 1.20E+07 | 3.45E+06 | 2.91E+06 | 5.67E+06 |
| **170** | F1SA98 | Uncharacterized protein | YWHAQ | 2.28E+08 | 9.14E+07 | 6.76E+07 | 6.92E+07 | 1.20E+07 | 4.81E+06 | 3.56E+06 | 3.64E+06 |
| **171** | A0A140TAK8 | Uncharacterized protein | APOH | 2.64E+08 | 7.31E+07 | 7.64E+07 | 1.14E+08 | 1.20E+07 | 3.32E+06 | 3.47E+06 | 5.19E+06 |
| **172** | F1SCC6 | Uncharacterized protein | LOC100153899 | 3.06E+08 | 9.71E+07 | 9.13E+07 | 1.18E+08 | 1.18E+07 | 3.73E+06 | 3.51E+06 | 4.52E+06 |
| **173** | F1SKJ8 | Uncharacterized protein | PVALB | 1.05E+08 | 3.52E+07 | 3.90E+07 | 3.12E+07 | 1.17E+07 | 3.92E+06 | 4.33E+06 | 3.47E+06 |
| **174** | V9HWE0 | Annexin | HEL-S-7 | 3.03E+08 | 9.13E+07 | 7.66E+07 | 1.35E+08 | 1.16E+07 | 3.51E+06 | 2.94E+06 | 5.19E+06 |
| **175** | Q5MJE5 | Cathepsin D protein (Fragment) | | 2.20E+08 | 8.46E+07 | 7.11E+07 | 6.43E+07 | 1.16E+07 | 4.45E+06 | 3.74E+06 | 3.38E+06 |
| **176** | F1SFI5 | Uncharacterized protein | HRG | 3.57E+08 | 2.34E+08 | 1.10E+08 | 1.26E+07 | 1.15E+07 | 7.54E+06 | 3.56E+06 | 4.08E+05 |
| **177** | K7EK07 | Histone H3 (Fragment) | H3F3B | 6.81E+07 | 4.73E+07 | 1.39E+07 | 6.90E+06 | 1.14E+07 | 7.89E+06 | 2.32E+06 | 1.15E+06 |
| **178** | F1RJW7 | Calsequestrin | CASQ1 | 1.81E+08 | 5.76E+07 | 6.20E+07 | 6.18E+07 | 1.13E+07 | 3.60E+06 | 3.88E+06 | 3.86E+06 |
| **179** | F1RLH7 | Uncharacterized protein | COX6B | 7.65E+07 | 4.10E+07 | 1.63E+07 | 1.92E+07 | 1.09E+07 | 5.85E+06 | 2.33E+06 | 2.75E+06 |
| **180** | P00506 | Aspartate aminotransferase, mitochondrial | GOT2 | 3.38E+08 | 1.76E+08 | 6.32E+07 | 9.88E+07 | 1.09E+07 | 5.66E+06 | 2.04E+06 | 3.19E+06 |
| **181** | J9JIL5 | Protein S100 | LOC100620367 | 5.36E+07 | 2.20E+07 | 1.71E+07 | 1.45E+07 | 1.07E+07 | 4.39E+06 | 3.42E+06 | 2.90E+06 |
| **182** | P50447 | Alpha-1-antitrypsin | SERPINA1 | 2.68E+08 | 5.11E+07 | 1.06E+08 | 1.11E+08 | 1.07E+07 | 2.04E+06 | 4.24E+06 | 4.43E+06 |
| **183** | Q5S1S4 | Carbonic anhydrase 3 | CA3 | 1.70E+08 | 8.49E+07 | 4.16E+07 | 4.35E+07 | 1.06E+07 | 5.30E+06 | 2.60E+06 | 2.72E+06 |
| **184** | F1SCC9 | Uncharacterized protein | | 6.30E+07 | 2.36E+07 | 2.56E+07 | 1.38E+07 | 1.05E+07 | 3.93E+06 | 4.26E+06 | 2.31E+06 |
| **185** | F1SEN2 | Glutamate dehydrogenase 1, mitochondrial | GLUD1 | 3.84E+08 | 1.03E+08 | 1.31E+08 | 1.49E+08 | 1.04E+07 | 2.79E+06 | 3.53E+06 | 4.04E+06 |
| **186** | L8B0S2 | IgG heavy chain | IGHG | 2.38E+08 | 7.28E+07 | 6.21E+07 | 1.03E+08 | 1.04E+07 | 3.16E+06 | 2.70E+06 | 4.49E+06 |
| **187** | K7EQW8 | Tropomyosin alpha-4 chain (Fragment) | TPM4 | 3.10E+07 | 3.10E+07 | 0.00E+00 | 0.00E+00 | 1.03E+07 | 1.03E+07 | 0.00E+00 | 0.00E+00 |
| **188** | L8B0V2 | IgG heavy chain | IGHG | 1.53E+08 | 3.50E+07 | 4.44E+07 | 7.34E+07 | 1.02E+07 | 2.33E+06 | 2.96E+06 | 4.89E+06 |
| **189** | P08132 | Annexin A4 | ANXA4 | 2.32E+08 | 7.42E+07 | 5.73E+07 | 1.01E+08 | 1.01E+07 | 3.23E+06 | 2.49E+06 | 4.38E+06 |
| **190** | P80276 | Aldose reductase | AKR1B1 | 2.09E+08 | 9.87E+07 | 5.71E+07 | 5.34E+07 | 9.96E+06 | 4.70E+06 | 2.72E+06 | 2.54E+06 |
| **191** | F1RUK8 | Rab GDP dissociation inhibitor beta | GDI2 | 2.97E+08 | 8.47E+07 | 8.14E+07 | 1.31E+08 | 9.89E+06 | 2.82E+06 | 2.71E+06 | 4.36E+06 |
| **192** | Q6IBG1 | MYL9 protein | MYL9 | 1.08E+08 | 4.53E+07 | 2.42E+07 | 3.87E+07 | 9.83E+06 | 4.11E+06 | 2.20E+06 | 3.52E+06 |
| **193** | Q29092 | Endoplasmin | HSP90B1 | 4.69E+08 | 2.08E+08 | 1.08E+08 | 1.52E+08 | 9.76E+06 | 4.34E+06 | 2.26E+06 | 3.17E+06 |
| **194** | F1SRC8 | Uncharacterized protein | CLEC3B | 1.16E+08 | 4.20E+07 | 2.44E+07 | 5.00E+07 | 9.70E+06 | 3.50E+06 | 2.03E+06 | 4.17E+06 |
| **195** | Q32Q10 | RSU1 protein (Fragment) | RSU1 | 1.44E+08 | 7.41E+07 | 3.22E+07 | 3.80E+07 | 9.61E+06 | 4.94E+06 | 2.14E+06 | 2.53E+06 |
| **196** | F1S418 | Uncharacterized protein | PRDX3 | 1.42E+08 | 4.43E+07 | 3.66E+07 | 6.14E+07 | 9.49E+06 | 2.95E+06 | 2.44E+06 | 4.09E+06 |
| **197** | P02189 | Myoglobin | MB | 1.13E+08 | 3.60E+07 | 2.73E+07 | 4.92E+07 | 9.37E+06 | 3.00E+06 | 2.28E+06 | 4.10E+06 |
| **198** | F1SS66 | Uncharacterized protein | MYH13 | 1.17E+09 | 8.14E+08 | 2.83E+08 | 7.69E+07 | 9.31E+06 | 6.46E+06 | 2.24E+06 | 6.11E+05 |
| **199** | F1RJT3 | Uncharacterized protein | LOC100525453 | 2.32E+08 | 9.45E+07 | 5.86E+07 | 7.85E+07 | 9.26E+06 | 3.78E+06 | 2.35E+06 | 3.14E+06 |
| **200** | P45846 | Dermatopontin | DPT | 7.30E+07 | 3.92E+07 | 1.25E+07 | 2.13E+07 | 9.13E+06 | 4.90E+06 | 1.56E+06 | 2.67E+06 |
| **201** | A0A0C4DFV9 | Protein SET | SET | 9.12E+07 | 4.90E+07 | 2.02E+07 | 2.20E+07 | 9.12E+06 | 4.90E+06 | 2.02E+06 | 2.20E+06 |
| **202** | Q2EN76 | Nucleoside diphosphate kinase B | NME2 | 1.27E+08 | 5.65E+07 | 1.93E+07 | 5.14E+07 | 9.09E+06 | 4.04E+06 | 1.38E+06 | 3.67E+06 |
| **203** | Q7M2W6 | Alpha-crystallin B chain | CRYAB | 1.18E+08 | 2.71E+07 | 2.75E+07 | 6.35E+07 | 9.09E+06 | 2.09E+06 | 2.11E+06 | 4.89E+06 |
| **204** | Q45FY6 | Hypoxanthine-guanine phosphoribosyltransferase | HPRT1 | 1.17E+08 | 4.08E+07 | 2.74E+07 | 4.87E+07 | 8.99E+06 | 3.14E+06 | 2.11E+06 | 3.74E+06 |
| **205** | F2Z5J9 | Uncharacterized protein | RAB11B | 1.43E+08 | 4.90E+07 | 3.38E+07 | 6.00E+07 | 8.93E+06 | 3.06E+06 | 2.11E+06 | 3.75E+06 |
| **206** | F2Z5E2 | Uncharacterized protein | SERPINC1 | 2.72E+08 | 7.08E+07 | 9.65E+07 | 1.05E+08 | 8.77E+06 | 2.28E+06 | 3.11E+06 | 3.37E+06 |
| **207** | F1SMC0 | Adenylyl cyclase-associated protein | | 2.27E+08 | 6.73E+07 | 5.90E+07 | 1.00E+08 | 8.72E+06 | 2.59E+06 | 2.27E+06 | 3.86E+06 |
| **208** | I3VKE6 | Ceruloplasmin | CP | 5.27E+08 | 2.40E+08 | 1.18E+08 | 1.69E+08 | 8.50E+06 | 3.88E+06 | 1.90E+06 | 2.73E+06 |
| **209** | A8KAH9 | RAP1A, member of RAS oncogene family | RAP1A | 8.42E+07 | 2.36E+07 | 2.30E+07 | 3.77E+07 | 8.42E+06 | 2.36E+06 | 2.30E+06 | 3.77E+06 |
| **210** | F1SRC5 | Aconitate hydratase, mitochondrial | ACO2 | 3.60E+08 | 1.39E+08 | 6.80E+07 | 1.54E+08 | 8.38E+06 | 3.23E+06 | 1.58E+06 | 3.57E+06 |
| **211** | Q29014 | Alpha-1 acid glycoprotein (Fragment) | ORM1 | 1.17E+08 | 2.93E+07 | 3.11E+07 | 5.65E+07 | 8.35E+06 | 2.09E+06 | 2.22E+06 | 4.03E+06 |
| **212** | L8B165 | IgG heavy chain | IGHG | 1.90E+08 | 5.72E+07 | 4.67E+07 | 8.58E+07 | 8.25E+06 | 2.49E+06 | 2.03E+06 | 3.73E+06 |
| **213** | Q9H299 | SH3 domain-binding glutamic acid-rich-like protein 3 | SH3BGRL3 | 3.26E+07 | 1.12E+07 | 4.60E+06 | 1.68E+07 | 8.14E+06 | 2.80E+06 | 1.15E+06 | 4.20E+06 |
| **214** | F1SUE3 | Uncharacterized protein | PPA1 | 1.46E+08 | 8.30E+07 | 2.00E+07 | 4.34E+07 | 8.13E+06 | 4.61E+06 | 1.11E+06 | 2.41E+06 |
| **215** | Q5G6V9 | Cofilin-2 | CFL2 | 1.05E+08 | 3.87E+07 | 2.48E+07 | 4.11E+07 | 8.05E+06 | 2.98E+06 | 1.91E+06 | 3.17E+06 |
| **216** | F1RTN3 | Moesin | MSN | 3.04E+08 | 6.85E+07 | 9.32E+07 | 1.42E+08 | 7.99E+06 | 1.80E+06 | 2.45E+06 | 3.74E+06 |
| **217** | F1SJE6 | Uncharacterized protein | PGM5 | 2.23E+08 | 6.99E+07 | 7.56E+07 | 7.71E+07 | 7.95E+06 | 2.50E+06 | 2.70E+06 | 2.75E+06 |
| **218** | Q007T6 | Superoxide dismutase [Cu-Zn] | | 7.88E+07 | 2.50E+07 | 2.61E+07 | 2.77E+07 | 7.88E+06 | 2.50E+06 | 2.61E+06 | 2.77E+06 |
| **219** | F2Z5G3 | Uncharacterized protein | CALM3 | 7.09E+07 | 1.33E+07 | 7.16E+06 | 5.04E+07 | 7.88E+06 | 1.48E+06 | 7.96E+05 | 5.60E+06 |
| **220** | F2Z5U4 | Ras-related protein Rab-1B | RAB1B | 1.40E+08 | 4.10E+07 | 3.74E+07 | 6.20E+07 | 7.80E+06 | 2.28E+06 | 2.08E+06 | 3.44E+06 |
| **221** | I3LQH7 | Uncharacterized protein | BLVRB | 1.01E+08 | 2.86E+07 | 2.61E+07 | 4.62E+07 | 7.76E+06 | 2.20E+06 | 2.01E+06 | 3.55E+06 |
| **222** | Q06AS6 | GBI2 | GNAI2 | 1.55E+08 | 7.39E+07 | 3.54E+07 | 4.53E+07 | 7.73E+06 | 3.69E+06 | 1.77E+06 | 2.27E+06 |
| **223** | F8W6I7 | Heterogeneous nuclear ribonucleoprotein A1 | HNRNPA1 | 1.38E+08 | 5.09E+07 | 2.87E+07 | 5.79E+07 | 7.64E+06 | 2.83E+06 | 1.59E+06 | 3.22E+06 |
| **224** | Q06830 | Peroxiredoxin-1 | PRDX1 | 1.15E+08 | 3.71E+07 | 2.98E+07 | 4.76E+07 | 7.64E+06 | 2.47E+06 | 1.99E+06 | 3.18E+06 |
| **225** | A7XZE4 | Beta tropomyosin isoform | TPM2b | 1.70E+08 | 8.45E+07 | 2.61E+07 | 5.93E+07 | 7.39E+06 | 3.67E+06 | 1.14E+06 | 2.58E+06 |
| **226** | Q29443 | Serotransferrin | | 3.53E+08 | 1.11E+08 | 9.27E+07 | 1.50E+08 | 7.35E+06 | 2.30E+06 | 1.93E+06 | 3.12E+06 |
| **227** | F1RJ93 | Transgelin | TAGLN2 | 1.02E+08 | 4.38E+07 | 2.13E+07 | 3.65E+07 | 7.26E+06 | 3.13E+06 | 1.52E+06 | 2.60E+06 |
| **228** | Q2EN74 | Epidermal fatty acid-binding protein | FABP5 | 7.89E+07 | 3.44E+07 | 1.12E+07 | 3.33E+07 | 7.17E+06 | 3.13E+06 | 1.02E+06 | 3.02E+06 |
| **229** | Q6UJZ1 | Glutathione peroxidase (Fragment) | GPX3 | 2.86E+07 | 6.17E+06 | 8.99E+06 | 1.34E+07 | 7.15E+06 | 1.54E+06 | 2.25E+06 | 3.36E+06 |
| **230** | K7EIU4 | KN motif and ankyrin repeat domain-containing protein 2 (Fragment) | KANK2 | 7.81E+07 | 1.95E+07 | 2.30E+07 | 3.56E+07 | 7.10E+06 | 1.77E+06 | 2.09E+06 | 3.23E+06 |
| **231** | F1SQE6 | Actin-related protein 2/3 complex subunit 4 | ARPC4 | 9.13E+07 | 3.65E+07 | 2.19E+07 | 3.29E+07 | 7.02E+06 | 2.80E+06 | 1.68E+06 | 2.53E+06 |
| **232** | F1RR02 | Uncharacterized protein | GFAP | 2.36E+08 | 8.20E+07 | 5.91E+07 | 9.47E+07 | 6.94E+06 | 2.41E+06 | 1.74E+06 | 2.78E+06 |
| **233** | Q9MYT8 | ATP synthase subunit e, mitochondrial | ATP5I | 2.73E+07 | 9.48E+06 | 7.75E+06 | 1.00E+07 | 6.81E+06 | 2.37E+06 | 1.94E+06 | 2.51E+06 |
| **234** | F1RFD4 | Uncharacterized protein | USMG5 | 2.02E+07 | 7.68E+06 | 5.78E+06 | 6.77E+06 | 6.74E+06 | 2.56E+06 | 1.93E+06 | 2.26E+06 |
| **235** | Q5SRT3 | Chloride intracellular channel protein | CLIC1 | 1.14E+08 | 5.95E+07 | 2.73E+07 | 2.70E+07 | 6.69E+06 | 3.50E+06 | 1.61E+06 | 1.59E+06 |
| **236** | A1XQV5 | Fast skeletal muscle troponin C | TNNC2 | 7.28E+07 | 1.80E+07 | 1.86E+07 | 3.62E+07 | 6.62E+06 | 1.63E+06 | 1.69E+06 | 3.29E+06 |
| **237** | F1SK00 | Isocitrate dehydrogenase [NADP] | IDH2 | 1.85E+08 | 6.96E+07 | 3.67E+07 | 7.89E+07 | 6.61E+06 | 2.49E+06 | 1.31E+06 | 2.82E+06 |
| **238** | F1SFB0 | Guanine nucleotide-binding protein subunit gamma | LOC100626889 | 5.18E+07 | 2.38E+07 | 9.34E+06 | 1.87E+07 | 6.48E+06 | 2.97E+06 | 1.17E+06 | 2.34E+06 |
| **239** | F1SUT0 | Uncharacterized protein | RAB6A | 1.10E+08 | 4.20E+07 | 2.13E+07 | 4.69E+07 | 6.48E+06 | 2.47E+06 | 1.25E+06 | 2.76E+06 |
| **240** | I3L397 | Eukaryotic translation initiation factor 5A (Fragment) | EIF5A | 5.18E+07 | 1.38E+07 | 1.29E+07 | 2.51E+07 | 6.47E+06 | 1.72E+06 | 1.61E+06 | 3.14E+06 |
| **241** | Q4JH15 | Troponin I | TNNI2 | 7.73E+07 | 4.21E+07 | 8.27E+06 | 2.69E+07 | 6.44E+06 | 3.51E+06 | 6.89E+05 | 2.24E+06 |
| **242** | F1SKZ0 | Uncharacterized protein | TIGAR | 9.66E+07 | 1.07E+06 | 4.88E+07 | 4.67E+07 | 6.44E+06 | 7.15E+04 | 3.25E+06 | 3.11E+06 |
| **243** | P00571 | Adenylate kinase isoenzyme 1 | AK1 | 9.65E+07 | 3.52E+07 | 2.71E+07 | 3.41E+07 | 6.43E+06 | 2.35E+06 | 1.81E+06 | 2.27E+06 |
| **244** | Q53DY7 | Histone H1.3-like protein (Fragment) | HIST1H1E | 5.11E+07 | 1.38E+07 | 1.35E+07 | 2.37E+07 | 6.39E+06 | 1.73E+06 | 1.69E+06 | 2.97E+06 |
| **245** | A0A0D5CDB2 | N(G),N(G)-dimethylarginine dimethylaminohydrolase 2 | DDAH2 | 1.21E+08 | 3.02E+07 | 3.47E+07 | 5.57E+07 | 6.35E+06 | 1.59E+06 | 1.82E+06 | 2.93E+06 |
| **246** | I3L6U3 | Uncharacterized protein (Fragment) | | 3.17E+07 | 1.55E+07 | 7.41E+06 | 8.75E+06 | 6.34E+06 | 3.11E+06 | 1.48E+06 | 1.75E+06 |
| **247** | Q2EN75 | Protein S100-A6 | S100A6 | 3.80E+07 | 9.14E+06 | 1.43E+07 | 1.46E+07 | 6.33E+06 | 1.52E+06 | 2.38E+06 | 2.43E+06 |
| **248** | F1SRB9 | Uncharacterized protein | ST13 | 9.47E+07 | 3.26E+07 | 2.32E+07 | 3.89E+07 | 6.31E+06 | 2.17E+06 | 1.55E+06 | 2.59E+06 |
| **249** | F2Z4Y1 | Uncharacterized protein | YWHAH | 1.12E+08 | 4.38E+07 | 3.67E+07 | 3.18E+07 | 6.24E+06 | 2.44E+06 | 2.04E+06 | 1.77E+06 |
| **250** | F1SQ01 | Uncharacterized protein | PRDX4 | 1.05E+08 | 3.82E+07 | 2.97E+07 | 3.75E+07 | 6.20E+06 | 2.24E+06 | 1.75E+06 | 2.20E+06 |
| **251** | I3LER3 | Uncharacterized protein | | 4.94E+07 | 4.08E+07 | 8.60E+06 | 0.00E+00 | 6.18E+06 | 5.10E+06 | 1.08E+06 | 0.00E+00 |
| **252** | I3L893 | Uncharacterized protein | GDI1 | 1.66E+08 | 5.46E+07 | 3.95E+07 | 7.16E+07 | 6.14E+06 | 2.02E+06 | 1.46E+06 | 2.65E+06 |
| **253** | L8B180 | IgG heavy chain | IGHG | 1.21E+08 | 2.04E+07 | 2.39E+07 | 7.68E+07 | 6.05E+06 | 1.02E+06 | 1.19E+06 | 3.84E+06 |
| **254** | A8U4R4 | Transketolase | tkt | 1.99E+08 | 5.11E+07 | 4.83E+07 | 1.00E+08 | 6.04E+06 | 1.55E+06 | 1.46E+06 | 3.03E+06 |
| **255** | Q6QAQ1 | Actin, cytoplasmic 1 | ACTB | 1.38E+08 | 7.66E+07 | 4.26E+07 | 1.87E+07 | 6.00E+06 | 3.33E+06 | 1.85E+06 | 8.15E+05 |
| **256** | F1ST01 | Uncharacterized protein | SELENBP1 | 1.85E+08 | 6.67E+07 | 4.71E+07 | 7.15E+07 | 5.98E+06 | 2.15E+06 | 1.52E+06 | 2.31E+06 |
| **257** | Q6FGH9 | DNCL1 protein | DNCL1 | 2.99E+07 | 1.16E+07 | 8.11E+06 | 1.02E+07 | 5.98E+06 | 2.32E+06 | 1.62E+06 | 2.04E+06 |
| **258** | P00889 | Citrate synthase, mitochondrial | CS | 1.30E+08 | 4.65E+07 | 2.22E+07 | 6.08E+07 | 5.89E+06 | 2.12E+06 | 1.01E+06 | 2.76E+06 |
| **259** | C9J9K3 | 40S ribosomal protein SA (Fragment) | RPSA | 8.73E+07 | 4.76E+07 | 2.36E+07 | 1.61E+07 | 5.82E+06 | 3.18E+06 | 1.57E+06 | 1.07E+06 |
| **260** | F6PVJ7 | Nucleoside diphosphate kinase | NME1 | 6.95E+07 | 3.81E+07 | 5.94E+06 | 2.54E+07 | 5.79E+06 | 3.18E+06 | 4.95E+05 | 2.12E+06 |
| **261** | A0A075B7J0 | Uncharacterized protein (Fragment) | | 2.30E+07 | 5.52E+06 | 5.19E+06 | 1.23E+07 | 5.75E+06 | 1.38E+06 | 1.30E+06 | 3.08E+06 |
| **262** | F1RMM0 | Uncharacterized protein | PRKCDBP | 8.04E+07 | 2.84E+07 | 1.93E+07 | 3.27E+07 | 5.75E+06 | 2.03E+06 | 1.38E+06 | 2.34E+06 |
| **263** | B7Z4T1 | cDNA FLJ55229, highly similar to Sphingosine kinase 2 (EC 2.7.1.-) | | 6.87E+07 | 2.70E+06 | 6.34E+07 | 2.60E+06 | 5.72E+06 | 2.25E+05 | 5.28E+06 | 2.17E+05 |
| **264** | F1RK48 | Uncharacterized protein | SRL | 2.23E+08 | 9.25E+07 | 5.60E+07 | 7.41E+07 | 5.71E+06 | 2.37E+06 | 1.44E+06 | 1.90E+06 |
| **265** | P42174 | Glutamate dehydrogenase 1, mitochondrial (Fragments) | GLUD1 | 1.71E+07 | 2.51E+06 | 2.06E+06 | 1.25E+07 | 5.71E+06 | 8.36E+05 | 6.88E+05 | 4.18E+06 |
| **266** | K7GKK5 | Uncharacterized protein (Fragment) | UBE2N | 5.69E+07 | 2.69E+07 | 4.20E+06 | 2.58E+07 | 5.69E+06 | 2.69E+06 | 4.20E+05 | 2.58E+06 |
| **267** | Q6WSP6 | Chaperonin 10 | EPFP1 | 5.66E+07 | 9.76E+06 | 1.87E+07 | 2.81E+07 | 5.66E+06 | 9.76E+05 | 1.87E+06 | 2.81E+06 |
| **268** | Q69YG1 | Myotrophin | DKFZp761E1322 | 4.51E+07 | 9.89E+06 | 1.75E+07 | 1.77E+07 | 5.64E+06 | 1.24E+06 | 2.19E+06 | 2.21E+06 |
| **269** | T1RTP3 | PDZ and LIM domain protein 1 | PDLIM1 | 1.06E+08 | 4.01E+07 | 3.36E+07 | 3.24E+07 | 5.59E+06 | 2.11E+06 | 1.77E+06 | 1.70E+06 |
| **270** | F1RJM2 | Endoplasmic reticulum resident protein 29 | ERP29 | 7.16E+07 | 3.33E+07 | 2.00E+07 | 1.83E+07 | 5.51E+06 | 2.56E+06 | 1.54E+06 | 1.41E+06 |
| **271** | A0A087X2D0 | Serine/arginine-rich-splicing factor 3 | SRSF3 | 4.39E+07 | 1.87E+07 | 1.35E+07 | 1.17E+07 | 5.49E+06 | 2.34E+06 | 1.68E+06 | 1.47E+06 |
| **272** | I3LIK6 | Uncharacterized protein | | 8.15E+07 | 4.14E+07 | 1.77E+07 | 2.23E+07 | 5.43E+06 | 2.76E+06 | 1.18E+06 | 1.49E+06 |
| **273** | F1SGS9 | Catalase | CAT | 1.73E+08 | 4.56E+07 | 5.88E+07 | 6.85E+07 | 5.40E+06 | 1.42E+06 | 1.84E+06 | 2.14E+06 |
| **274** | F2Z5J5 | Enhancer of rudimentary homolog | ERH | 3.23E+07 | 6.48E+06 | 1.05E+07 | 1.54E+07 | 5.39E+06 | 1.08E+06 | 1.75E+06 | 2.56E+06 |
| **275** | Q59F44 | Cytochrome b-5 isoform 1 variant (Fragment) | CYB5A | 4.83E+07 | 1.72E+07 | 3.83E+06 | 2.73E+07 | 5.37E+06 | 1.92E+06 | 4.25E+05 | 3.03E+06 |
| **276** | H0YA96 | Heterogeneous nuclear ribonucleoprotein D0 (Fragment) | HNRNPD | 7.49E+07 | 3.45E+07 | 1.63E+07 | 2.42E+07 | 5.35E+06 | 2.46E+06 | 1.16E+06 | 1.73E+06 |
| **277** | F8W1N5 | Nascent polypeptide-associated complex subunit alpha (Fragment) | NACA | 2.67E+07 | 6.27E+06 | 6.27E+06 | 1.41E+07 | 5.33E+06 | 1.25E+06 | 1.25E+06 | 2.83E+06 |
| **278** | Q6IM70 | Reticulon | RTN4 | 3.70E+07 | 2.81E+07 | 4.57E+06 | 4.32E+06 | 5.29E+06 | 4.02E+06 | 6.54E+05 | 6.17E+05 |
| **279** | F1RQQ8 | Alpha-1,4 glucan phosphorylase | PYGM | 3.01E+08 | 1.29E+08 | 8.19E+07 | 9.04E+07 | 5.29E+06 | 2.26E+06 | 1.44E+06 | 1.59E+06 |
| **280** | Q29221 | F-actin-capping protein subunit alpha-2 | CAPZA2 | 8.35E+07 | 5.22E+07 | 1.53E+07 | 1.60E+07 | 5.22E+06 | 3.26E+06 | 9.56E+05 | 9.99E+05 |
| **281** | F1S441 | Uncharacterized protein | CANX | 1.92E+08 | 6.60E+07 | 5.25E+07 | 7.39E+07 | 5.20E+06 | 1.78E+06 | 1.42E+06 | 2.00E+06 |
| **282** | V9HW44 | Epididymis secretory protein Li 303 | HEL-S-303 | 4.66E+07 | 1.95E+07 | 1.38E+07 | 1.33E+07 | 5.18E+06 | 2.17E+06 | 1.53E+06 | 1.48E+06 |
| **283** | I3L929 | Alcohol dehydrogenase [NADP(+)] | AKR1A1 | 1.29E+08 | 6.16E+07 | 3.13E+07 | 3.58E+07 | 5.15E+06 | 2.47E+06 | 1.25E+06 | 1.43E+06 |
| **284** | B9TRW9 | Guanine nucleotide-binding protein subunit gamma | GNG12 | 2.57E+07 | 1.06E+07 | 6.01E+06 | 9.12E+06 | 5.14E+06 | 2.11E+06 | 1.20E+06 | 1.82E+06 |
| **285** | A0A024QZJ6 | Myosin, heavy polypeptide 11, smooth muscle, isoform CRA | MYH11 | 6.32E+08 | 4.62E+08 | 1.28E+08 | 4.32E+07 | 5.10E+06 | 3.72E+06 | 1.03E+06 | 3.48E+05 |
| **286** | I3LVS7 | Uncharacterized protein | RHOA | 6.08E+07 | 2.67E+07 | 1.11E+07 | 2.29E+07 | 5.06E+06 | 2.23E+06 | 9.25E+05 | 1.91E+06 |
| **287** | Q765P8 | Prostaglandin D synthase (Fragment) | PTGDS | 3.54E+07 | 1.44E+07 | 9.34E+06 | 1.17E+07 | 5.06E+06 | 2.06E+06 | 1.33E+06 | 1.67E+06 |
| **288** | F1RVE7 | Integrin beta | ITGB1 | 1.87E+08 | 6.20E+07 | 4.55E+07 | 7.95E+07 | 5.06E+06 | 1.68E+06 | 1.23E+06 | 2.15E+06 |
| **289** | P01025 | Complement C3 | C3 | 5.53E+08 | 1.59E+08 | 1.73E+08 | 2.21E+08 | 5.03E+06 | 1.45E+06 | 1.57E+06 | 2.01E+06 |
| **290** | F1RU49 | Uncharacterized protein | LOC100519531 | 3.06E+08 | 1.07E+08 | 8.94E+07 | 1.10E+08 | 5.02E+06 | 1.76E+06 | 1.46E+06 | 1.80E+06 |
| **291** | V9HW04 | Serine/threonine-protein phosphatase | HEL-S-80p | 9.38E+07 | 4.67E+07 | 2.09E+07 | 2.62E+07 | 4.94E+06 | 2.46E+06 | 1.10E+06 | 1.38E+06 |
| **292** | F1RII5 | Uncharacterized protein | LOC100515788 | 4.92E+07 | 1.76E+07 | 1.04E+07 | 2.12E+07 | 4.92E+06 | 1.76E+06 | 1.04E+06 | 2.12E+06 |

**Supplementary Table S1: (continued)**

| | | | | | | | | | | | |
|---|---|---|---|---|---|---|---|---|---|---|---|
| **293** | G9F6Y0 | Phosducin (Fragment) | PDC | 6.85E+07 | 3.32E+07 | 1.88E+07 | 1.65E+07 | 4.89E+06 | 2.37E+06 | 1.34E+06 | 1.18E+06 |
| **294** | F1RKI3 | Uncharacterized protein | HINT1 | 3.38E+07 | 1.80E+07 | 8.79E+06 | 7.00E+06 | 4.83E+06 | 2.57E+06 | 1.26E+06 | 1.00E+06 |
| **295** | H7BZJ3 | Protein disulfide-isomerase A3 (Fragment) | PDIA3 | 4.32E+07 | 1.59E+07 | 2.05E+07 | 6.74E+06 | 4.80E+06 | 1.77E+06 | 2.28E+06 | 7.49E+05 |
| **296** | F1S6Q7 | ATP synthase subunit delta, mitochondrial | ATP5D | 2.87E+07 | 9.68E+06 | 3.06E+06 | 1.59E+07 | 4.78E+06 | 1.61E+06 | 5.10E+05 | 2.65E+06 |
| **297** | F1RM62 | Uncharacterized protein | HSPB6 | 3.34E+07 | 8.79E+06 | 1.12E+07 | 1.34E+07 | 4.77E+06 | 1.26E+06 | 1.60E+06 | 1.91E+06 |
| **298** | F1T0F7 | Cellular retinoic acid binding protein 1 | CRABP1 | 5.71E+07 | 2.73E+07 | 9.90E+06 | 1.98E+07 | 4.76E+06 | 2.28E+06 | 8.25E+05 | 1.65E+06 |
| **299** | P80310 | Protein S100-A12 | S100A12 | 2.83E+07 | 1.01E+07 | 1.09E+07 | 7.40E+06 | 4.72E+06 | 1.68E+06 | 1.81E+06 | 1.23E+06 |
| **300** | E1CAJ6 | Protein disulfide isomerase P5 | pdi-p5 | 1.18E+08 | 4.79E+07 | 3.13E+07 | 3.85E+07 | 4.71E+06 | 1.92E+06 | 1.25E+06 | 1.54E+06 |
| **301** | L0R4Z3 | Alternative protein KCTD3 | KCTD3 | 2.35E+07 | 3.67E+06 | 7.43E+06 | 1.24E+07 | 4.70E+06 | 7.34E+05 | 1.49E+06 | 2.48E+06 |
| **302** | B0LY42 | CD147 | | 7.99E+07 | 4.28E+07 | 1.79E+07 | 1.93E+07 | 4.70E+06 | 2.52E+06 | 1.05E+06 | 1.13E+06 |
| **303** | P59768 | Guanine nucleotide-binding protein G(I)/G(S)/G(O) subunit gamma-2 | GNG2 | 2.80E+07 | 1.08E+07 | 7.00E+06 | 1.02E+07 | 4.67E+06 | 1.80E+06 | 1.17E+06 | 1.71E+06 |
| **304** | Q3YLA6 | Serine/arginine-rich splicing factor 1 | SRSF1 | 6.99E+07 | 2.98E+07 | 1.79E+07 | 2.22E+07 | 4.66E+06 | 1.99E+06 | 1.19E+06 | 1.48E+06 |
| **305** | F1SLF0 | Uncharacterized protein | SPR | 7.91E+07 | 3.23E+07 | 2.56E+07 | 2.13E+07 | 4.65E+06 | 1.90E+06 | 1.50E+06 | 1.25E+06 |
| **306** | B4DX77 | Nicolin-1 | NICN1 | 6.04E+07 | 4.23E+07 | 1.81E+07 | 0.00E+00 | 4.64E+06 | 3.25E+06 | 1.39E+06 | 0.00E+00 |
| **307** | Q9MZ16 | Voltage-dependent anion-selective channel protein 1 | VDAC1 | 8.29E+07 | 3.85E+07 | 1.28E+07 | 3.16E+07 | 4.61E+06 | 2.14E+06 | 7.12E+05 | 1.75E+06 |
| **308** | F1SIH8 | Transitional endoplasmic reticulum ATPase | VCP | 2.21E+08 | 8.90E+07 | 5.83E+07 | 7.35E+07 | 4.60E+06 | 1.85E+06 | 1.21E+06 | 1.53E+06 |
| **309** | F1SD87 | Uncharacterized protein | FBLN5 | 1.01E+08 | 3.39E+07 | 2.55E+07 | 4.16E+07 | 4.59E+06 | 1.54E+06 | 1.16E+06 | 1.89E+06 |
| **310** | C9J7L5 | Acetyl-coenzyme A synthetase, cytoplasmic (Fragment) | ACSS2 | 3.66E+07 | 3.42E+07 | 2.33E+06 | 0.00E+00 | 4.57E+06 | 4.28E+06 | 2.92E+05 | 0.00E+00 |
| **311** | Q7Z612 | Acidic ribosomal phosphoprotein P1 | RPLP1 | 1.83E+07 | 1.02E+07 | 7.01E+06 | 1.07E+06 | 4.57E+06 | 2.54E+06 | 1.75E+06 | 2.68E+05 |
| **312** | F1S285 | Uncharacterized protein | COL14A1 | 4.10E+08 | 1.96E+08 | 1.11E+08 | 1.02E+08 | 4.55E+06 | 2.18E+06 | 1.24E+06 | 1.13E+06 |
| **313** | F1S0M2 | Uncharacterized protein | HRSP12 | 4.43E+07 | 1.74E+07 | 1.43E+07 | 1.27E+07 | 4.43E+06 | 1.74E+06 | 1.43E+06 | 1.27E+06 |
| **314** | F1S2K3 | Uncharacterized protein | NUTF2 | 3.54E+07 | 1.33E+07 | 1.30E+07 | 9.11E+06 | 4.42E+06 | 1.66E+06 | 1.62E+06 | 1.14E+06 |
| **315** | F1RYZ0 | 60S acidic ribosomal protein P2 | RPLP2 | 2.20E+07 | 7.31E+06 | 7.62E+06 | 7.05E+06 | 4.40E+06 | 1.46E+06 | 1.52E+06 | 1.41E+06 |
| **316** | Q007T3 | Tetraspanin | CD81 | 3.95E+07 | 3.12E+07 | 4.08E+06 | 4.27E+06 | 4.39E+06 | 3.46E+06 | 4.54E+05 | 4.75E+05 |
| **317** | A0A158RFU6 | RAB7, member RAS oncogene family, isoform CRA | RAB7A | 7.45E+07 | 2.71E+07 | 2.05E+07 | 2.69E+07 | 4.38E+06 | 1.59E+06 | 1.21E+06 | 1.58E+06 |
| **318** | L8B0X5 | IgG heavy chain | IGHG | 8.76E+07 | 4.06E+07 | 2.10E+07 | 2.60E+07 | 4.38E+06 | 2.03E+06 | 1.05E+06 | 1.30E+06 |
| **319** | A0A087X2J4 | Vacuolar protein sorting-associated protein 11 homolog (Fragment) | VPS11 | 5.68E+07 | 2.57E+07 | 1.04E+07 | 2.07E+07 | 4.37E+06 | 1.97E+06 | 8.03E+05 | 1.59E+06 |
| **320** | Q6QAT1 | 40S ribosomal protein S28 | RPS28 | 1.30E+07 | 6.04E+06 | 3.07E+06 | 3.88E+06 | 4.33E+06 | 2.01E+06 | 1.02E+06 | 1.29E+06 |
| **321** | Q53Y06 | ATPase, H+ transporting, lysosomal 31kDa, V1 subunit E isoform 1 | ATP6V1E1 | 6.86E+07 | 2.84E+07 | 1.84E+07 | 2.18E+07 | 4.29E+06 | 1.78E+06 | 1.15E+06 | 1.36E+06 |
| **322** | Q9GJT2 | S-formylglutathione hydrolase | ESD | 6.79E+07 | 3.53E+07 | 1.12E+07 | 2.13E+07 | 4.24E+06 | 2.21E+06 | 7.01E+05 | 1.33E+06 |
| **323** | L8B0W9 | IgG heavy chain | IGHG | 7.62E+07 | 2.57E+07 | 2.21E+07 | 2.85E+07 | 4.23E+06 | 1.43E+06 | 1.23E+06 | 1.58E+06 |
| **324** | F1S1R4 | SH3 domain-binding glutamic acid-rich-like protein | SH3BGRL | 3.79E+07 | 1.32E+07 | 1.28E+07 | 1.20E+07 | 4.22E+06 | 1.47E+06 | 1.42E+06 | 1.33E+06 |
| **325** | P10523 | S-arrestin | SAG | 1.10E+08 | 1.37E+07 | 2.02E+07 | 7.57E+07 | 4.21E+06 | 5.27E+05 | 7.77E+05 | 2.91E+06 |
| **326** | I3LD72 | Uncharacterized protein | EHD2 | 1.47E+08 | 6.21E+07 | 3.86E+07 | 4.59E+07 | 4.19E+06 | 1.78E+06 | 1.10E+06 | 1.31E+06 |
| **327** | B9TSR8 | Complement factor B | CFB | 1.84E+08 | 7.34E+07 | 4.88E+07 | 6.15E+07 | 4.17E+06 | 1.67E+06 | 1.11E+06 | 1.40E+06 |
| **328** | Q6FIC5 | Chloride intracellular channel protein | CLIC4 | 7.07E+07 | 3.58E+07 | 1.89E+07 | 1.59E+07 | 4.16E+06 | 2.11E+06 | 1.11E+06 | 9.38E+05 |
| **329** | Q007T2 | Cell division control protein 42 homolog | CDC42 | 3.73E+07 | 1.12E+07 | 9.83E+06 | 1.62E+07 | 4.14E+06 | 1.24E+06 | 1.09E+06 | 1.80E+06 |
| **330** | F1RQP0 | Uncharacterized protein | PRDX5 | 4.12E+07 | 2.07E+07 | 6.32E+06 | 1.42E+07 | 4.12E+06 | 2.07E+06 | 6.32E+05 | 1.42E+06 |
| **331** | F1SAK6 | Uncharacterized protein | ATP5L | 2.88E+07 | 9.07E+06 | 8.11E+06 | 1.17E+07 | 4.12E+06 | 1.30E+06 | 1.16E+06 | 1.66E+06 |
| **332** | F1S5R9 | Guanylate kinase | GUK1 | 5.73E+07 | 1.43E+07 | 1.97E+07 | 2.32E+07 | 4.09E+06 | 1.02E+06 | 1.41E+06 | 1.66E+06 |
| **333** | F1SHD6 | Uncharacterized protein | EEF1B2 | 6.13E+07 | 2.27E+07 | 2.12E+07 | 1.74E+07 | 4.09E+06 | 1.52E+06 | 1.41E+06 | 1.16E+06 |
| **334** | F2Z560 | Uncharacterized protein | RAB5C | 5.72E+07 | 2.26E+07 | 1.24E+07 | 2.22E+07 | 4.08E+06 | 1.61E+06 | 8.84E+05 | 1.58E+06 |
| **335** | F1RHL9 | Uncharacterized protein | ACTN2 | 2.52E+08 | 8.73E+07 | 7.64E+07 | 8.86E+07 | 4.07E+06 | 1.41E+06 | 1.23E+06 | 1.43E+06 |
| **336** | Q5U0A0 | Proteasome subunit alpha type | PSMA5 | 4.85E+07 | 2.28E+07 | 8.30E+06 | 1.74E+07 | 4.04E+06 | 1.90E+06 | 6.92E+05 | 1.45E+06 |
| **337** | F1RYY6 | Transaldolase | TALDO1 | 8.35E+07 | 4.50E+07 | 1.58E+07 | 2.27E+07 | 3.98E+06 | 2.14E+06 | 7.53E+05 | 1.08E+06 |
| **338** | P09623 | Dihydrolipoyl dehydrogenase, mitochondrial | DLD | 1.06E+08 | 3.23E+07 | 3.05E+07 | 4.35E+07 | 3.94E+06 | 1.20E+06 | 1.13E+06 | 1.61E+06 |
| **339** | I3LDW9 | Small nuclear ribonucleoprotein E | SNRPE | 1.57E+07 | 4.05E+06 | 5.44E+06 | 6.22E+06 | 3.93E+06 | 1.01E+06 | 1.36E+06 | 1.56E+06 |
| **340** | F1SMZ7 | Uncharacterized protein | HSPD1 | 1.37E+08 | 5.91E+07 | 4.03E+07 | 3.80E+07 | 3.93E+06 | 1.69E+06 | 1.15E+06 | 1.09E+06 |
| **341** | I3LKF3 | Fascin | LOC100625519 | 1.17E+08 | 3.98E+07 | 3.97E+07 | 3.76E+07 | 3.90E+06 | 1.33E+06 | 1.32E+06 | 1.25E+06 |
| **342** | E7EQG2 | Eukaryotic initiation factor 4A-II | EIF4A2 | 8.58E+07 | 2.63E+07 | 1.94E+07 | 4.01E+07 | 3.90E+06 | 1.20E+06 | 8.80E+05 | 1.82E+06 |
| **343** | Q8MJ30 | Dihydropteridine reductase | QDPR | 6.60E+07 | 2.50E+07 | 2.17E+07 | 1.93E+07 | 3.88E+06 | 1.47E+06 | 1.28E+06 | 1.14E+06 |
| **344** | Q1W2G0 | 14-3-3 sigma protein | Sfn | 6.97E+07 | 2.08E+07 | 2.46E+07 | 2.42E+07 | 3.87E+06 | 1.16E+06 | 1.37E+06 | 1.34E+06 |
| **345** | A0A024R7V6 | RAB2, member RAS oncogene family, isoform CRA | RAB2 | 5.80E+07 | 1.68E+07 | 1.15E+07 | 2.98E+07 | 3.87E+06 | 1.12E+06 | 7.65E+05 | 1.98E+06 |
| **346** | Q52NJ4 | ADP-ribosylation factor-like protein 3 | ARL3 | 4.19E+07 | 1.28E+07 | 1.13E+07 | 1.78E+07 | 3.81E+06 | 1.16E+06 | 1.03E+06 | 1.62E+06 |
| **347** | Q6IAV3 | Eukaryotic translation initiation factor 1, isoform CRA | SUI1 | 1.88E+07 | 8.52E+06 | 5.67E+06 | 4.65E+06 | 3.77E+06 | 1.70E+06 | 1.13E+06 | 9.31E+05 |
| **348** | Q95250 | Membrane-associated progesterone receptor component 1 | PGRMC1 | 3.77E+07 | 1.71E+07 | 9.03E+06 | 1.16E+07 | 3.77E+06 | 1.71E+06 | 9.03E+05 | 1.16E+06 |
| **349** | A0A024R216 | Hepatoma-derived growth factor, related protein 3, isoform CRA | HDGFRP3 | 3.76E+07 | 1.53E+07 | 1.06E+07 | 1.17E+07 | 3.76E+06 | 1.53E+06 | 1.06E+06 | 1.17E+06 |
| **350** | F1RHW4 | Uncharacterized protein | RRAS | 5.63E+07 | 2.42E+07 | 1.42E+07 | 1.78E+07 | 3.75E+06 | 1.61E+06 | 9.48E+05 | 1.19E+06 |
| **351** | F1RX37 | Fibrinogen beta chain | FGB | 1.27E+08 | 5.90E+07 | 2.89E+07 | 3.86E+07 | 3.72E+06 | 1.74E+06 | 8.50E+05 | 1.14E+06 |
| **352** | A0A075B7I7 | Uncharacterized protein (Fragment) | | 2.60E+07 | 1.12E+07 | 5.02E+06 | 9.71E+06 | 3.71E+06 | 1.60E+06 | 7.17E+05 | 1.39E+06 |
| **353** | C9J0J7 | Profilin | PFN2 | 2.22E+07 | 1.26E+07 | 4.22E+06 | 5.42E+06 | 3.71E+06 | 2.10E+06 | 7.03E+05 | 9.04E+05 |
| **354** | F1SJW8 | Uncharacterized protein | SERPING1 | 9.22E+07 | 2.41E+07 | 1.88E+07 | 4.94E+07 | 3.69E+06 | 9.63E+05 | 7.50E+05 | 1.98E+06 |
| **355** | P35527 | Keratin, type I cytoskeletal 9 | KRT9 | 1.11E+08 | 3.67E+07 | 3.78E+07 | 3.62E+07 | 3.69E+06 | 1.22E+06 | 1.26E+06 | 1.21E+06 |
| **356** | Q6FHM2 | GNB2 protein | GNB2 | 4.76E+07 | 2.53E+07 | 9.58E+06 | 1.27E+07 | 3.66E+06 | 1.95E+06 | 7.37E+05 | 9.76E+05 |
| **357** | O02772 | Fatty acid-binding protein, heart | FABP3 | 4.74E+07 | 2.06E+07 | 1.54E+07 | 1.14E+07 | 3.65E+06 | 1.59E+06 | 1.19E+06 | 8.76E+05 |
| **358** | F2Z5D2 | Uncharacterized protein | ACTR3 | 7.94E+07 | 2.94E+07 | 1.70E+07 | 3.30E+07 | 3.61E+06 | 1.34E+06 | 7.72E+05 | 1.50E+06 |
| **359** | I3LAQ0 | Uncharacterized protein (Fragment) | | 2.16E+07 | 6.51E+06 | 6.32E+06 | 8.73E+06 | 3.59E+06 | 1.08E+06 | 1.05E+06 | 1.46E+06 |
| **360** | P63246 | Receptor of activated protein C kinase 1 | RACK1 | 7.85E+07 | 4.52E+07 | 1.04E+07 | 2.29E+07 | 3.57E+06 | 2.05E+06 | 4.73E+05 | 1.04E+06 |
| **361** | K7GQD4 | Protein S100 (Fragment) | S100A16 | 1.78E+07 | 4.42E+06 | 5.82E+06 | 7.58E+06 | 3.56E+06 | 8.85E+05 | 1.16E+06 | 1.52E+06 |
| **362** | B4DKJ4 | cDNA FLJ57738, highly similar to Translationally-controlled tumor protein | TPT1 | 2.13E+07 | 5.99E+06 | 3.68E+06 | 1.16E+07 | 3.55E+06 | 9.98E+05 | 6.14E+05 | 1.94E+06 |
| **363** | Q6FHG6 | PRELP protein (Fragment) | PRELP | 8.86E+07 | 3.76E+07 | 2.14E+07 | 2.96E+07 | 3.54E+06 | 1.50E+06 | 8.58E+05 | 1.18E+06 |
| **364** | F1S3W0 | Cytochrome b-c1 complex subunit 6 | LOC100524873 | 2.82E+07 | 6.50E+06 | 1.10E+07 | 1.08E+07 | 3.53E+06 | 8.12E+05 | 1.37E+06 | 1.35E+06 |
| **365** | H0YMA1 | Proteasome subunit alpha type (Fragment) | PSMA4 | 3.88E+07 | 1.53E+07 | 1.33E+07 | 1.02E+07 | 3.53E+06 | 1.39E+06 | 1.21E+06 | 9.29E+05 |
| **366** | F1RPC8 | Uncharacterized protein | CRYM | 4.91E+07 | 1.94E+07 | 9.58E+06 | 2.01E+07 | 3.51E+06 | 1.39E+06 | 6.85E+05 | 1.43E+06 |
| **367** | I3LFW6 | Uncharacterized protein | UBE2V1 | 4.20E+07 | 1.55E+07 | 3.30E+06 | 2.32E+07 | 3.50E+06 | 1.29E+06 | 2.75E+05 | 1.93E+06 |
| **368** | H3BPD9 | Nucleoside diphosphate kinase 3 (Fragment) | NME3 | 1.39E+07 | 4.26E+06 | 2.77E+06 | 6.90E+06 | 3.48E+06 | 1.07E+06 | 6.92E+05 | 1.72E+06 |
| **369** | K7GMI9 | Tetraspanin (Fragment) | CD9 | 2.08E+07 | 6.00E+06 | 1.50E+06 | 1.33E+07 | 3.47E+06 | 1.00E+06 | 2.50E+05 | 2.22E+06 |
| **370** | K7GRY0 | Uncharacterized protein (Fragment) | UBA1 | 1.90E+08 | 7.59E+07 | 4.27E+07 | 7.10E+07 | 3.45E+06 | 1.38E+06 | 7.77E+05 | 1.29E+06 |
| **371** | I3LS04 | Uncharacterized protein | ACTN1 | 9.85E+07 | 3.93E+07 | 2.69E+07 | 3.23E+07 | 3.40E+06 | 1.36E+06 | 9.27E+05 | 1.11E+06 |
| **372** | I3LHC8 | Uncharacterized protein | LOC100522692 | 5.06E+07 | 2.04E+07 | 1.15E+07 | 1.87E+07 | 3.37E+06 | 1.36E+06 | 7.65E+05 | 1.25E+06 |
| **373** | I3LRX9 | Uncharacterized protein | C14H10orf116 | 1.35E+07 | 4.62E+06 | 3.19E+06 | 5.67E+06 | 3.37E+06 | 1.16E+06 | 7.98E+05 | 1.42E+06 |
| **374** | V9HW56 | Epididymis secretory protein Li 108 | HEL-S-108 | 7.03E+07 | 2.77E+07 | 2.04E+07 | 2.22E+07 | 3.35E+06 | 1.32E+06 | 9.72E+05 | 1.06E+06 |
| **375** | L8AXK3 | IgG heavy chain | IGHG | 6.98E+07 | 2.11E+07 | 2.06E+07 | 2.81E+07 | 3.32E+06 | 1.01E+06 | 9.80E+05 | 1.34E+06 |
| **376** | P00348 | Hydroxyacyl-coenzyme A dehydrogenase, mitochondrial | HADH | 6.31E+07 | 3.03E+07 | 9.82E+06 | 2.30E+07 | 3.32E+06 | 1.59E+06 | 5.17E+05 | 1.21E+06 |
| **377** | Q2XQV4 | Aldehyde dehydrogenase, mitochondrial | ALDH2 | 8.56E+07 | 3.03E+07 | 2.69E+07 | 2.85E+07 | 3.29E+06 | 1.16E+06 | 1.03E+06 | 1.09E+06 |
| **378** | F1RUQ0 | Uncharacterized protein | JCHAIN | 1.97E+07 | 6.67E+06 | 5.39E+06 | 7.65E+06 | 3.28E+06 | 1.11E+06 | 8.98E+05 | 1.28E+06 |
| **379** | Q64L94 | Proteasome activator complex subunit 1 | PSME1 | 5.87E+07 | 3.24E+07 | 1.15E+07 | 1.48E+07 | 3.26E+06 | 1.80E+06 | 6.40E+05 | 8.20E+05 |
| **380** | I3LNK1 | Uncharacterized protein | OLFML1 | 7.80E+07 | 2.78E+07 | 2.12E+07 | 2.89E+07 | 3.25E+06 | 1.16E+06 | 8.85E+05 | 1.20E+06 |
| **381** | A4D2P1 | Ras-related C3 botulinum toxin substrate 1 (Rho family, small GTP binding protein Rac1) | RAC1 | 3.22E+07 | 1.21E+07 | 7.54E+06 | 1.25E+07 | 3.22E+06 | 1.21E+06 | 7.54E+05 | 1.25E+06 |
| **382** | F1SBR6 | Olfactomedin-like protein 3 | OLFML3 | 7.38E+07 | 2.19E+07 | 1.94E+07 | 3.26E+07 | 3.21E+06 | 9.50E+05 | 8.42E+05 | 1.42E+06 |
| **383** | F1SUE4 | Uncharacterized protein | ASPN | 7.05E+07 | 3.08E+07 | 1.11E+07 | 2.86E+07 | 3.20E+06 | 1.40E+06 | 5.04E+05 | 1.30E+06 |
| **384** | K9IWI4 | Talin-1 | TLN1 | 4.84E+08 | 2.23E+08 | 1.21E+08 | 1.40E+08 | 3.19E+06 | 1.47E+06 | 7.99E+05 | 9.19E+05 |
| **385** | A0A075B7H6 | Uncharacterized protein | | 1.58E+07 | 5.55E+06 | 4.44E+06 | 5.79E+06 | 3.16E+06 | 1.11E+06 | 8.88E+05 | 1.16E+06 |
| **386** | A0A024R5K8 | Serpin peptidase inhibitor, clade H (Heat shock protein 47), member 1, (Collagen binding protein 1), isoform CRA | SERPINH1 | 8.20E+07 | 2.80E+07 | 2.06E+07 | 3.34E+07 | 3.15E+06 | 1.08E+06 | 7.91E+05 | 1.29E+06 |
| **387** | F1S340 | Uncharacterized protein | SRI | 4.08E+07 | 1.55E+07 | 6.48E+06 | 1.88E+07 | 3.14E+06 | 1.19E+06 | 4.98E+05 | 1.45E+06 |
| **388** | F1RPD2 | Uncharacterized protein | LOC100524613 | 6.87E+07 | 2.70E+07 | 1.68E+07 | 2.49E+07 | 3.12E+06 | 1.23E+06 | 7.62E+05 | 1.13E+06 |
| **389** | A0A0K1TQQ7 | Carbonyl reductase 3 | CBR3 | 6.52E+07 | 2.49E+07 | 2.04E+07 | 1.99E+07 | 3.10E+06 | 1.19E+06 | 9.69E+05 | 9.49E+05 |
| **390** | B7Z992 | cDNA FLJ53698, highly similar to Gelsolin | GSN | 1.12E+08 | 3.72E+07 | 2.38E+07 | 5.06E+07 | 3.10E+06 | 1.03E+06 | 6.61E+05 | 1.41E+06 |
| **391** | Q05DH1 | Proteasome subunit alpha type (Fragment) | PSMA7 | 4.95E+07 | 2.49E+07 | 1.68E+07 | 7.83E+06 | 3.09E+06 | 1.56E+06 | 1.05E+06 | 4.90E+05 |
| **392** | F1SL99 | Uncharacterized protein | PTGES3 | 2.78E+07 | 1.44E+07 | 4.31E+06 | 9.13E+06 | 3.09E+06 | 1.60E+06 | 4.78E+05 | 1.01E+06 |
| **393** | B4DUD6 | cDNA FLJ54551, highly similar to Septin-7 | SEPT7 | 7.71E+07 | 2.63E+07 | 2.32E+07 | 2.76E+07 | 3.08E+06 | 1.05E+06 | 9.28E+05 | 1.10E+06 |
| **394** | Q7YS78 | Arrestin-C | ARR3 | 6.47E+07 | 2.45E+07 | 1.44E+07 | 2.57E+07 | 3.08E+06 | 1.17E+06 | 6.87E+05 | 1.22E+06 |
| **395** | H0YB18 | Putative hydroxypyruvate isomerase (Fragment) | HYI | 3.37E+07 | 0.00E+00 | 0.00E+00 | 3.37E+07 | 3.07E+06 | 0.00E+00 | 0.00E+00 | 3.07E+06 |
| **396** | Q06AU3 | Ras-related protein Rab-3A | RAB3A | 4.88E+07 | 2.45E+07 | 9.95E+06 | 1.44E+07 | 3.05E+06 | 1.53E+06 | 6.22E+05 | 9.02E+05 |
| **397** | A5A8V8 | LSM2 homolog, U6 small nuclear RNA associated (S. cerevisiae) | LSM2 | 2.13E+07 | 6.54E+06 | 7.21E+06 | 7.58E+06 | 3.05E+06 | 9.34E+05 | 1.03E+06 | 1.08E+06 |
| **398** | F1RZQ6 | Uncharacterized protein | SLC25A4 | 6.38E+07 | 4.42E+07 | 1.47E+07 | 4.91E+06 | 3.04E+06 | 2.10E+06 | 7.00E+05 | 2.34E+05 |
| **399** | J3KNQ4 | Alpha-parvin | PARVA | 7.25E+07 | 3.25E+07 | 1.87E+07 | 2.13E+07 | 3.02E+06 | 1.35E+06 | 7.81E+05 | 8.89E+05 |
| **400** | F1RIF8 | 6-phosphogluconate dehydrogenase, decarboxylating | PGD | 9.35E+07 | 2.74E+07 | 2.34E+07 | 4.27E+07 | 3.02E+06 | 8.83E+05 | 7.54E+05 | 1.38E+06 |
| **401** | F1S325 | Hydroxypyruvate isomerase | HYI | 4.49E+07 | 1.86E+07 | 1.19E+07 | 1.45E+07 | 2.99E+06 | 1.24E+06 | 7.92E+05 | 9.64E+05 |
| **402** | B1AKZ4 | Phosphoprotein enriched in astrocytes 15, isoform CRA | PEA15 | 2.39E+07 | 6.96E+06 | 3.91E+06 | 1.30E+07 | 2.99E+06 | 8.70E+05 | 4.89E+05 | 1.63E+06 |
| **403** | I3LCX3 | Uncharacterized protein | DUSP3 | 2.99E+07 | 1.35E+07 | 6.60E+06 | 9.75E+06 | 2.99E+06 | 1.35E+06 | 6.60E+05 | 9.75E+05 |
| **404** | K7GKE4 | Uncharacterized protein (Fragment) | CD44 | 5.97E+06 | 2.08E+06 | 1.51E+06 | 2.37E+06 | 2.98E+06 | 1.04E+06 | 7.55E+05 | 1.19E+06 |
| **405** | A0A0D5BWD2 | Mitochondrial complement component 1 Q subcomponent-binding protein | C1QBP | 4.16E+07 | 1.25E+07 | 1.54E+07 | 1.37E+07 | 2.97E+06 | 8.94E+05 | 1.10E+06 | 9.82E+05 |
| **406** | A0A087WTH9 | Conserved oligomeric Golgi complex subunit 3 (Fragment) | COG3 | 1.78E+07 | 1.48E+07 | 2.99E+06 | 0.00E+00 | 2.97E+06 | 2.47E+06 | 4.98E+05 | 0.00E+00 |
| **407** | P63208 | S-phase kinase-associated protein 1 | SKP1 | 3.56E+07 | 1.32E+07 | 1.03E+07 | 1.21E+07 | 2.97E+06 | 1.10E+06 | 8.58E+05 | 1.01E+06 |
| **408** | P79319 | Cytochrome C oxidase polypeptide VB (Fragment) | COX5B | 1.78E+07 | 1.07E+07 | 2.74E+06 | 4.37E+06 | 2.96E+06 | 1.78E+06 | 4.57E+05 | 7.28E+05 |
| **409** | F1RX36 | Uncharacterized protein | LOC102158263 | 6.09E+07 | 1.85E+07 | 1.99E+07 | 2.25E+07 | 2.90E+06 | 8.81E+05 | 9.49E+05 | 1.07E+06 |
| **410** | F1S4Y6 | Uncharacterized protein | DYNLRB1 | 1.73E+07 | 4.44E+06 | 5.70E+06 | 7.20E+06 | 2.89E+06 | 7.40E+05 | 9.49E+05 | 1.20E+06 |
| **411** | F1SU52 | Uncharacterized protein | FAHD2A | 5.49E+07 | 2.54E+07 | 1.19E+07 | 1.76E+07 | 2.89E+06 | 1.34E+06 | 6.26E+05 | 9.24E+05 |
| **412** | I3LJ05 | Uncharacterized protein | C13H21orf33 | 5.17E+07 | 2.81E+07 | 9.07E+06 | 1.46E+07 | 2.87E+06 | 1.56E+06 | 5.04E+05 | 8.10E+05 |
| **413** | Q2YHQ3 | Filamin-A (Fragment) | FLNA | 3.45E+07 | 1.17E+07 | 9.99E+06 | 1.28E+07 | 2.87E+06 | 9.73E+05 | 8.32E+05 | 1.07E+06 |
| **414** | P61958 | Small ubiquitin-related modifier 2 | SUMO2 | 1.14E+07 | 3.22E+06 | 2.92E+06 | 5.25E+06 | 2.85E+06 | 8.06E+05 | 7.31E+05 | 1.31E+06 |
| **415** | Q6QAS5 | 60S ribosomal protein L12 (Fragment) | RPL12 | 3.40E+07 | 1.20E+07 | 9.45E+06 | 1.26E+07 | 2.83E+06 | 1.00E+06 | 7.87E+05 | 1.05E+06 |
| **416** | I3LD43 | Uncharacterized protein | | 3.97E+07 | 9.06E+06 | 1.10E+07 | 1.96E+07 | 2.83E+06 | 6.47E+05 | 7.82E+05 | 1.40E+06 |
| **417** | P79263 | Inter-alpha-trypsin inhibitor heavy chain H4 | ITIH4 | 1.52E+08 | 4.81E+07 | 3.53E+07 | 6.84E+07 | 2.76E+06 | 8.75E+05 | 6.42E+05 | 1.24E+06 |
| **418** | D2JYW4 | F-actin capping protein beta subunit variant II | CAPZB | 5.79E+07 | 2.64E+07 | 1.14E+07 | 2.01E+07 | 2.76E+06 | 1.26E+06 | 5.42E+05 | 9.55E+05 |
| **419** | B8Y648 | Ubiquitin-conjugating enzyme E2L 3 | UBE2L3 | 2.19E+07 | 8.78E+06 | 4.62E+06 | 8.54E+06 | 2.74E+06 | 1.10E+06 | 5.77E+05 | 1.07E+06 |
| **420** | Q9NR12 | PDZ and LIM domain protein 7 | PDLIM7 | 8.21E+07 | 3.56E+07 | 2.09E+07 | 2.56E+07 | 2.74E+06 | 1.19E+06 | 6.97E+05 | 8.53E+05 |
| **421** | F1SQ52 | Myosin light chain kinase, smooth muscle | MYLK | 6.84E+07 | 2.24E+07 | 2.25E+07 | 2.35E+07 | 2.73E+06 | 8.94E+05 | 9.01E+05 | 9.39E+05 |
| **422** | Q29551 | Succinyl-CoA:3-ketoacid coenzyme A transferase 1, mitochondrial | OXCT1 | 7.07E+07 | 1.09E+07 | 2.00E+07 | 3.98E+07 | 2.72E+06 | 4.18E+05 | 7.68E+05 | 1.53E+06 |
| **423** | F1SM61 | Fibulin-1 | FBLN1 | 8.70E+07 | 3.51E+07 | 2.21E+07 | 2.98E+07 | 2.72E+06 | 1.10E+06 | 6.90E+05 | 9.32E+05 |
| **424** | Q6IBA2 | PC4 protein | PC4 | 2.71E+07 | 1.12E+07 | 3.79E+06 | 1.21E+07 | 2.71E+06 | 1.12E+06 | 3.79E+05 | 1.21E+06 |
| **425** | I3LCC6 | Uncharacterized protein | PSMB3 | 2.70E+06 | 1.51E+06 | 0.00E+00 | 1.19E+06 | 2.70E+06 | 1.51E+06 | 0.00E+00 | 1.19E+06 |
| **426** | Q29214 | 60S acidic ribosomal protein P0 | RPLP0 | 4.85E+07 | 2.53E+07 | 9.94E+06 | 1.33E+07 | 2.70E+06 | 1.41E+06 | 5.52E+05 | 7.38E+05 |
| **427** | Q94PW4 | ATP synthase protein 8 | ATPase 8 | 1.08E+07 | 3.83E+06 | 2.80E+06 | 4.14E+06 | 2.69E+06 | 9.57E+05 | 6.99E+05 | 1.04E+06 |
| **428** | I3L9F5 | Uncharacterized protein | PCYOX1 | 5.90E+07 | 1.82E+07 | 1.94E+07 | 2.14E+07 | 2.68E+06 | 8.26E+05 | 8.83E+05 | 9.74E+05 |
| **429** | F1RGA9 | Coronin | CORO1C | 6.89E+07 | 2.73E+07 | 1.74E+07 | 2.43E+07 | 2.65E+06 | 1.05E+06 | 6.69E+05 | 9.33E+05 |
| **430** | K7ERI7 | 60S ribosomal protein L22 | RPL22 | 1.59E+07 | 9.84E+06 | 1.15E+06 | 4.91E+06 | 2.65E+06 | 1.64E+06 | 1.91E+05 | 8.18E+05 |
| **431** | I3LIQ9 | Uncharacterized protein | | 4.50E+07 | 3.48E+07 | 7.70E+06 | 2.52E+06 | 2.65E+06 | 2.04E+06 | 4.53E+05 | 1.48E+05 |
| **432** | O77591 | Inositol monophosphatase 1 | IMPA1 | 3.96E+07 | 1.48E+07 | 1.34E+07 | 1.14E+07 | 2.64E+06 | 9.88E+05 | 8.92E+05 | 7.62E+05 |
| **433** | Q04967 | Heat shock 70 kDa protein 6 | HSPA6 | 9.99E+07 | 2.83E+07 | 3.20E+07 | 3.96E+07 | 2.63E+06 | 7.45E+05 | 8.41E+05 | 1.04E+06 |
| **434** | F1RR78 | Uncharacterized protein | SPTAN1 | 4.89E+08 | 2.91E+08 | 9.36E+07 | 1.04E+08 | 2.61E+06 | 1.56E+06 | 5.01E+05 | 5.55E+05 |
| **435** | P80895 | Protein-L-isoaspartate(D-aspartate) O-methyltransferase | PCMT1 | 3.12E+07 | 1.59E+07 | 4.30E+06 | 1.10E+07 | 2.60E+06 | 1.32E+06 | 3.59E+05 | 9.20E+05 |
| **436** | Q95339 | ATP synthase subunit f, mitochondrial | ATP5J2 | 1.03E+07 | 2.61E+06 | 3.65E+06 | 4.09E+06 | 2.59E+06 | 6.51E+05 | 9.12E+05 | 1.02E+06 |
| **437** | Q29545 | Inhibitor of carbonic anhydrase | ICA | 9.90E+07 | 3.63E+07 | 2.73E+07 | 3.54E+07 | 2.54E+06 | 9.30E+05 | 7.00E+05 | 9.07E+05 |
| **438** | I3LVT1 | Adenylyl cyclase-associated protein | | 1.52E+07 | 5.07E+06 | 4.07E+06 | 6.01E+06 | 2.53E+06 | 8.46E+05 | 6.79E+05 | 1.00E+06 |
| **439** | A0A087WUL2 | Proteasome subunit beta type-3 (Fragment) | PSMB3 | 2.26E+07 | 7.24E+06 | 5.35E+06 | 9.99E+06 | 2.51E+06 | 8.05E+05 | 5.95E+05 | 1.11E+06 |
| **440** | A0A024QZ34 | Microfibrillar-associated protein 4, isoform CRA | MFAP4 | 3.51E+07 | 1.28E+07 | 6.14E+06 | 1.62E+07 | 2.51E+06 | 9.16E+05 | 4.38E+05 | 1.15E+06 |

**Supplementary Table S1: (continued)**

| | | | | | | | | | | | |
|---|---|---|---|---|---|---|---|---|---|---|---|
| **441** | F1RX35 | Uncharacterized protein | LOC100627396 | 7.50E+07 | 3.57E+07 | 1.83E+07 | 2.10E+07 | 2.50E+06 | 1.19E+06 | 6.10E+05 | 7.00E+05 |
| **442** | F1RLG5 | Receptor expression-enhancing protein | REEP5 | 1.99E+07 | 9.39E+06 | 4.32E+06 | 6.22E+06 | 2.49E+06 | 1.17E+06 | 5.41E+05 | 7.77E+05 |
| **443** | P24540 | Acylphosphatase-1 | ACYP1 | 1.74E+07 | 4.52E+06 | 6.17E+06 | 6.73E+06 | 2.49E+06 | 6.45E+05 | 8.81E+05 | 9.61E+05 |
| **444** | I3L7T8 | Uncharacterized protein | ATP5H | 1.99E+07 | 7.69E+06 | 7.12E+06 | 5.08E+06 | 2.49E+06 | 9.61E+05 | 8.90E+05 | 6.35E+05 |
| **445** | Q00P29 | Beta actin (Fragment) | ACTB | 4.97E+06 | 4.97E+06 | 0.00E+00 | 0.00E+00 | 2.48E+06 | 2.48E+06 | 0.00E+00 | 0.00E+00 |
| **446** | P02769 | Bovine serum albumin precursor | | 1.12E+08 | 5.62E+07 | 5.18E+07 | 3.65E+06 | 2.48E+06 | 1.25E+06 | 1.15E+06 | 8.12E+04 |
| **447** | Q6I9T8 | Serine/threonine-protein phosphatase | PPP2CA | 4.47E+07 | 2.73E+07 | 9.76E+06 | 7.65E+06 | 2.48E+06 | 1.51E+06 | 5.42E+05 | 4.25E+05 |
| **448** | F1SKM0 | Uncharacterized protein | UQCRC1 | 5.93E+07 | 1.81E+07 | 1.92E+07 | 2.20E+07 | 2.47E+06 | 7.55E+05 | 8.00E+05 | 9.17E+05 |
| **449** | Q53R19 | Actin related protein 2/3 complex, subunit 2, 34kDa, isoform CRA | ARPC2 | 5.56E+07 | 1.90E+07 | 1.19E+07 | 2.47E+07 | 2.42E+06 | 8.25E+05 | 5.17E+05 | 1.07E+06 |
| **450** | Q07717 | Beta-2-microglobulin | B2M | 1.69E+07 | 3.29E+06 | 5.30E+06 | 8.32E+06 | 2.42E+06 | 4.70E+05 | 7.58E+05 | 1.19E+06 |
| **451** | K7GP49 | Uncharacterized protein | MCAM | 7.90E+07 | 3.67E+07 | 1.67E+07 | 2.56E+07 | 2.39E+06 | 1.11E+06 | 5.05E+05 | 7.77E+05 |
| **452** | B4E2F9 | cDNA FLJ57038, highly similar to Filamin-A | | 1.10E+08 | 4.41E+07 | 2.84E+07 | 3.70E+07 | 2.38E+06 | 9.58E+05 | 6.18E+05 | 8.04E+05 |
| **453** | Q000H8 | Glutathione S-transferase mu 2 (Fragment) | | 3.32E+07 | 1.37E+07 | 8.47E+06 | 1.10E+07 | 2.37E+06 | 9.81E+05 | 6.05E+05 | 7.89E+05 |
| **454** | Q5JP53 | Tubulin beta chain | TUBB | 4.95E+07 | 2.51E+07 | 1.46E+07 | 9.78E+06 | 2.36E+06 | 1.20E+06 | 6.96E+05 | 4.66E+05 |
| **455** | F1SR80 | Uncharacterized protein | LOC100158003 | 5.35E+07 | 1.18E+07 | 1.39E+07 | 2.78E+07 | 2.32E+06 | 5.13E+05 | 6.05E+05 | 1.21E+06 |
| **456** | F2Z5N0 | Proteasome subunit alpha type | PSMA6 | 3.94E+07 | 1.60E+07 | 9.59E+06 | 1.38E+07 | 2.32E+06 | 9.41E+05 | 5.64E+05 | 8.14E+05 |
| **457** | F1SSG6 | Uncharacterized protein | ANP32B | 2.78E+07 | 1.16E+07 | 5.94E+06 | 1.02E+07 | 2.32E+06 | 9.70E+05 | 4.95E+05 | 8.53E+05 |
| **458** | B3KY04 | cDNA FLJ46506 fis, clone THYMU3030752, highly similar to BTB/POZ domain-containing protein KCTD12 | KC | 3.94E+07 | 1.86E+07 | 1.29E+07 | 7.81E+06 | 2.32E+06 | 1.10E+06 | 7.61E+05 | 4.59E+05 |
| **459** | F1SK35 | Uncharacterized protein | RLBP1 | 4.16E+07 | 2.26E+07 | 5.40E+06 | 1.37E+07 | 2.31E+06 | 1.25E+06 | 3.00E+05 | 7.59E+05 |
| **460** | I3LUC8 | Peptidyl-prolyl cis-trans isomerase | | 3.45E+07 | 9.14E+06 | 9.35E+06 | 1.60E+07 | 2.30E+06 | 6.09E+05 | 6.23E+05 | 1.07E+06 |
| **461** | F1SKY2 | Uncharacterized protein | NIT2 | 5.52E+07 | 1.82E+07 | 1.68E+07 | 2.02E+07 | 2.30E+06 | 7.57E+05 | 7.00E+05 | 8.41E+05 |
| **462** | F1S1A9 | Uncharacterized protein | APOA2 | 1.38E+07 | 5.88E+06 | 3.11E+06 | 4.79E+06 | 2.30E+06 | 9.80E+05 | 5.19E+05 | 7.98E+05 |
| **463** | Q9N1F5 | Glutathione S-transferase omega-1 | GSTO1 | 3.61E+07 | 1.44E+07 | 1.16E+07 | 1.01E+07 | 2.26E+06 | 9.01E+05 | 7.25E+05 | 6.30E+05 |
| **464** | I3LRH2 | Uncharacterized protein | LOC100519984 | 1.04E+08 | 2.44E+07 | 3.69E+07 | 4.23E+07 | 2.25E+06 | 5.31E+05 | 8.03E+05 | 9.20E+05 |
| **465** | K7GRW9 | Uncharacterized protein | RS1 | 2.92E+07 | 1.47E+07 | 6.59E+06 | 7.98E+06 | 2.25E+06 | 1.13E+06 | 5.07E+05 | 6.14E+05 |
| **466** | F1RHN3 | Catechol O-methyltransferase | COMT | 3.34E+07 | 1.48E+07 | 7.84E+06 | 1.08E+07 | 2.23E+06 | 9.87E+05 | 5.23E+05 | 7.18E+05 |
| **467** | F1SQX9 | Uncharacterized protein | APOD | 2.21E+07 | 8.54E+06 | 7.18E+06 | 6.37E+06 | 2.21E+06 | 8.54E+05 | 7.18E+05 | 6.37E+05 |
| **468** | F2Z5G9 | Uncharacterized protein | SNRPD1 | 1.10E+07 | 4.59E+06 | 3.37E+06 | 3.07E+06 | 2.21E+06 | 9.19E+05 | 6.74E+05 | 6.13E+05 |
| **469** | I3L9V2 | Uncharacterized protein | VAT1 | 4.63E+07 | 2.19E+07 | 6.50E+06 | 1.79E+07 | 2.20E+06 | 1.04E+06 | 3.10E+05 | 8.52E+05 |
| **470** | Q00P25 | Hypoxanthine phosphoribosyltransferase 1 (Fragment) | HPRT1 | 6.60E+06 | 6.60E+06 | 0.00E+00 | 0.00E+00 | 2.20E+06 | 2.20E+06 | 0.00E+00 | 0.00E+00 |
| **471** | A0A024R5Z9 | Pyruvate kinase | PKM2 | 8.57E+07 | 2.85E+07 | 3.25E+07 | 2.47E+07 | 2.20E+06 | 7.30E+05 | 8.34E+05 | 6.33E+05 |
| **472** | F1ST02 | Proteasome subunit beta type | PSMB4 | 2.39E+07 | 5.99E+06 | 5.15E+06 | 1.28E+07 | 2.17E+06 | 5.45E+05 | 4.68E+05 | 1.16E+06 |
| **473** | I3LER5 | Cytochrome c oxidase subunit 4 isoform 1, mitochondrial | COX4I1 | 2.61E+07 | 1.72E+07 | 1.25E+06 | 7.65E+06 | 2.17E+06 | 1.43E+06 | 1.04E+05 | 6.38E+05 |
| **474** | Q49AN9 | SNRPG protein | SNRPG | 4.33E+06 | 1.13E+06 | 9.82E+05 | 2.23E+06 | 2.17E+06 | 5.63E+05 | 4.91E+05 | 1.11E+06 |
| **475** | P80229 | Leukocyte elastase inhibitor | SERPINB1 | 5.76E+07 | 3.12E+07 | 1.46E+07 | 1.18E+07 | 2.13E+06 | 1.16E+06 | 5.39E+05 | 4.38E+05 |
| **476** | I3LR51 | Uncharacterized protein | FKBP3 | 1.92E+07 | 8.03E+06 | 6.51E+06 | 4.63E+06 | 2.13E+06 | 8.92E+05 | 7.24E+05 | 5.15E+05 |
| **477** | P68137 | Actin, alpha skeletal muscle | ACTA1 | 4.85E+07 | 6.24E+06 | 8.87E+06 | 3.34E+07 | 2.11E+06 | 2.71E+05 | 3.86E+05 | 1.45E+06 |
| **478** | Q2LE71 | Actin-related protein 2/3 complex subunit 3 | ARPC3 | 2.52E+07 | 8.31E+06 | 6.95E+06 | 9.97E+06 | 2.10E+06 | 6.92E+05 | 5.79E+05 | 8.31E+05 |
| **479** | A0A024R883 | ATPase, H+ transporting, lysosomal 13kDa, V1 subunit G1, isoform CRA | ATP6V1G1 | 1.47E+07 | 5.79E+06 | 1.55E+06 | 7.37E+06 | 2.10E+06 | 8.27E+05 | 2.21E+05 | 1.05E+06 |
| **480** | I3LQS0 | Uncharacterized protein | HNRNPK | 5.42E+07 | 1.47E+07 | 1.70E+07 | 2.25E+07 | 2.08E+06 | 5.64E+05 | 6.54E+05 | 8.66E+05 |
| **481** | Q86TY5 | Galectin | hCG | 2.29E+07 | 1.23E+07 | 4.54E+06 | 6.05E+06 | 2.08E+06 | 1.12E+06 | 4.13E+05 | 5.50E+05 |
| **482** | I3LLY4 | Uncharacterized protein | NQO2 | 1.04E+07 | 2.65E+06 | 3.25E+06 | 4.51E+06 | 2.08E+06 | 5.29E+05 | 6.51E+05 | 9.02E+05 |
| **483** | F1S232 | 4-trimethylaminobutyraldehyde dehydrogenase | ALDH9A1 | 7.26E+07 | 1.77E+07 | 2.41E+07 | 3.09E+07 | 2.08E+06 | 5.05E+05 | 6.87E+05 | 8.83E+05 |
| **484** | Q2NLD4 | PURA protein (Fragment) | PURA | 3.11E+07 | 9.28E+06 | 5.80E+06 | 1.60E+07 | 2.07E+06 | 6.19E+05 | 3.87E+05 | 1.07E+06 |
| **485** | F1RUI1 | Uncharacterized protein | HSD17B10 | 3.31E+07 | 1.74E+07 | 9.02E+06 | 6.64E+06 | 2.07E+06 | 1.09E+06 | 5.64E+05 | 4.15E+05 |
| **486** | I3LQ51 | Proteasome subunit beta type | PSMB1 | 2.88E+07 | 9.75E+06 | 4.94E+06 | 1.41E+07 | 2.06E+06 | 6.97E+05 | 3.53E+05 | 1.01E+06 |
| **487** | I3LAQ6 | Uncharacterized protein | ABHD14B | 2.25E+07 | 7.56E+06 | 6.28E+06 | 8.67E+06 | 2.05E+06 | 6.87E+05 | 5.71E+05 | 7.88E+05 |
| **488** | C3S7K6 | Calcium-binding protein A9 | S100A9 | 1.22E+07 | 3.11E+06 | 2.35E+06 | 6.74E+06 | 2.03E+06 | 5.18E+05 | 3.92E+05 | 1.12E+06 |
| **489** | F1S8H5 | DNA-(apurinic or apyrimidinic site) lyase | APEX1 | 3.85E+07 | 2.91E+07 | 2.71E+06 | 6.69E+06 | 2.03E+06 | 1.53E+06 | 1.42E+05 | 3.52E+05 |
| **490** | D6R9P3 | Heterogeneous nuclear ribonucleoprotein A/B | HNRNPAB | 2.83E+07 | 1.32E+07 | 6.31E+06 | 8.79E+06 | 2.02E+06 | 9.41E+05 | 4.50E+05 | 6.28E+05 |
| **491** | P37111 | Aminoacylase-1 | ACY1 | 4.82E+07 | 1.36E+07 | 1.29E+07 | 2.16E+07 | 2.01E+06 | 5.68E+05 | 5.36E+05 | 9.02E+05 |
| **492** | O94875 | Sorbin and SH3 domain-containing protein 2 | SORBS2 | 1.26E+08 | 5.08E+07 | 2.85E+07 | 4.67E+07 | 2.00E+06 | 8.06E+05 | 4.53E+05 | 7.41E+05 |
| **493** | Q0Z8R0 | Cystatin | CST3 | 1.59E+07 | 5.59E+06 | 6.52E+06 | 3.78E+06 | 1.99E+06 | 6.99E+05 | 8.15E+05 | 4.73E+05 |
| **494** | K7GQW5 | Ectonucleoside triphosphate diphosphohydrolase 1 (Fragment) | ENTPD1 | 1.74E+07 | 5.90E+06 | 4.41E+06 | 7.13E+06 | 1.94E+06 | 6.56E+05 | 4.89E+05 | 7.92E+05 |
| **495** | F1RHJ2 | Uncharacterized protein | HDGF | 3.46E+07 | 1.15E+07 | 1.16E+07 | 1.15E+07 | 1.92E+06 | 6.38E+05 | 6.45E+05 | 6.37E+05 |
| **496** | F1ST88 | Uncharacterized protein | GLIPR2 | 1.89E+07 | 6.58E+06 | 4.47E+06 | 7.82E+06 | 1.89E+06 | 6.58E+05 | 4.47E+05 | 7.82E+05 |
| **497** | P86412 | Myelin P2 protein | PMP2 | 2.26E+07 | 1.27E+07 | 3.69E+06 | 6.24E+06 | 1.88E+06 | 1.06E+06 | 3.07E+05 | 5.20E+05 |
| **498** | P51525 | Prophenin-2 | | 3.20E+07 | 1.39E+07 | 8.71E+06 | 9.43E+06 | 1.88E+06 | 8.18E+05 | 5.12E+05 | 5.54E+05 |
| **499** | Q53GF5 | Proteasome subunit alpha type (Fragment) | | 3.00E+07 | 1.11E+07 | 8.40E+06 | 1.05E+07 | 1.88E+06 | 6.95E+05 | 5.25E+05 | 6.57E+05 |
| **500** | F1RL38 | Uncharacterized protein | DDT | 1.47E+07 | 3.40E+06 | 5.95E+06 | 5.40E+06 | 1.84E+06 | 4.24E+05 | 7.44E+05 | 6.75E+05 |
| **501** | F1RYJ8 | Uncharacterized protein | CSRP2 | 2.56E+07 | 1.18E+07 | 6.52E+06 | 7.34E+06 | 1.83E+06 | 8.41E+05 | 4.66E+05 | 5.24E+05 |
| **502** | I3L9I6 | Uncharacterized protein | BCAM | 6.76E+07 | 1.38E+07 | 2.12E+07 | 3.26E+07 | 1.83E+06 | 3.72E+05 | 5.74E+05 | 8.80E+05 |
| **503** | M3UZ63 | UPF0556 protein C19orf10 | C19orf10 | 2.18E+07 | 1.17E+07 | 1.37E+06 | 8.73E+06 | 1.82E+06 | 9.74E+05 | 1.14E+05 | 7.27E+05 |
| **504** | Q710C4 | Adenosylhomocysteinase | AHCY | 4.30E+07 | 1.74E+07 | 1.04E+07 | 1.53E+07 | 1.79E+06 | 7.24E+05 | 4.33E+05 | 6.37E+05 |
| **505** | F1RPW9 | Elongation factor 1-gamma | EEF1G | 4.47E+07 | 1.82E+07 | 9.79E+06 | 1.66E+07 | 1.79E+06 | 7.30E+05 | 3.92E+05 | 6.65E+05 |
| **506** | F2Z5P9 | Uncharacterized protein | LOC100739296 | 1.24E+07 | 1.54E+06 | 4.66E+06 | 6.23E+06 | 1.78E+06 | 2.20E+05 | 6.66E+05 | 8.90E+05 |
| **507** | D6RAW0 | Ubiquitin-conjugating enzyme E2 D3 (Fragment) | UBE2D3 | 5.31E+06 | 4.04E+06 | 0.00E+00 | 1.28E+06 | 1.77E+06 | 1.35E+06 | 0.00E+00 | 4.26E+05 |
| **508** | F1SAC1 | Uncharacterized protein | ECHS1 | 3.18E+07 | 1.43E+07 | 9.53E+06 | 7.94E+06 | 1.77E+06 | 7.96E+05 | 5.29E+05 | 4.41E+05 |
| **509** | Q6UAQ8 | Electron transfer flavoprotein subunit beta | ETFB | 2.47E+07 | 1.22E+07 | 8.90E+06 | 3.56E+06 | 1.76E+06 | 8.73E+05 | 6.36E+05 | 2.54E+05 |
| **510** | F1SFV3 | Uncharacterized protein | S100A14 | 1.41E+07 | 6.08E+06 | 3.72E+06 | 4.25E+06 | 1.76E+06 | 7.60E+05 | 4.66E+05 | 5.32E+05 |
| **511** | Q9UEH5 | 24-kDa subunit of complex I (Fragment) | NDUFV2 | 2.45E+07 | 8.78E+06 | 6.48E+06 | 9.20E+06 | 1.75E+06 | 6.27E+05 | 4.63E+05 | 6.57E+05 |
| **512** | F1RZR6 | Uncharacterized protein | CUTA | 1.22E+07 | 4.71E+06 | 4.00E+06 | 3.50E+06 | 1.74E+06 | 6.73E+05 | 5.71E+05 | 5.00E+05 |
| **513** | P35908 | Keratin, type II cytoskeletal 2 epidermal | KRT2 | 7.45E+07 | 2.92E+07 | 1.52E+07 | 3.01E+07 | 1.73E+06 | 6.79E+05 | 3.54E+05 | 7.01E+05 |
| **514** | I3LC07 | Uncharacterized protein | RAB18 | 2.43E+07 | 8.78E+06 | 6.92E+06 | 8.56E+06 | 1.73E+06 | 6.27E+05 | 4.94E+05 | 6.11E+05 |
| **515** | F1STY7 | Uncharacterized protein | AAMDC | 8.60E+06 | 4.59E+06 | 1.99E+06 | 2.02E+06 | 1.72E+06 | 9.19E+05 | 3.98E+05 | 4.04E+05 |
| **516** | L8AXL9 | IgG heavy chain | IGHG | 3.08E+07 | 1.36E+07 | 1.15E+07 | 5.73E+06 | 1.71E+06 | 7.56E+05 | 6.38E+05 | 3.18E+05 |
| **517** | F1SJ34 | Uncharacterized protein | COX5A | 2.05E+07 | 2.96E+06 | 9.37E+06 | 8.20E+06 | 1.71E+06 | 2.46E+05 | 7.81E+05 | 6.83E+05 |
| **518** | A6YNL5 | Calpain 2 | Capn2 | 6.33E+07 | 2.08E+07 | 1.68E+07 | 2.58E+07 | 1.71E+06 | 5.61E+05 | 4.53E+05 | 6.96E+05 |
| **519** | F1S956 | Uncharacterized protein | PGLS | 2.04E+07 | 8.94E+06 | 6.45E+06 | 5.04E+06 | 1.70E+06 | 7.45E+05 | 5.37E+05 | 4.20E+05 |
| **520** | A0A024R4X0 | NADH-cytochrome b5 reductase | CYB5R3 | 2.72E+07 | 1.30E+07 | 5.81E+06 | 8.33E+06 | 1.70E+06 | 8.13E+05 | 3.63E+05 | 5.21E+05 |
| **521** | F1RGJ3 | Stress-70 protein, mitochondrial | HSPA9 | 7.22E+07 | 2.62E+07 | 1.72E+07 | 2.88E+07 | 1.68E+06 | 6.09E+05 | 3.99E+05 | 6.71E+05 |
| **522** | F1SAD9 | Protein disulfide-isomerase | PDIA4 | 7.53E+07 | 2.73E+07 | 2.40E+07 | 2.40E+07 | 1.67E+06 | 6.07E+05 | 5.34E+05 | 5.33E+05 |
| **523** | Q8SPJ9 | Cytochrome c oxidase subunit 7A1, mitochondrial | COX7A1 | 8.25E+06 | 3.45E+06 | 1.71E+06 | 3.09E+06 | 1.65E+06 | 6.90E+05 | 3.42E+05 | 6.19E+05 |
| **524** | K9J6J2 | Erythrocyte band 7 integral membrane protein isoform a | STOM_tv1 | 2.79E+07 | 1.45E+07 | 7.73E+06 | 5.74E+06 | 1.64E+06 | 8.50E+05 | 4.55E+05 | 3.38E+05 |
| **525** | K9IW69 | Calcium-transporting ATPase | ATP2A3_tv1 | 8.66E+07 | 2.98E+07 | 2.67E+07 | 3.01E+07 | 1.63E+06 | 5.63E+05 | 5.04E+05 | 5.67E+05 |
| **526** | P27485 | Retinol-binding protein 4 | RBP4 | 1.63E+07 | 6.46E+06 | 3.08E+06 | 6.75E+06 | 1.63E+06 | 6.46E+05 | 3.08E+05 | 6.75E+05 |
| **527** | Q1T7A8 | Type VI collagen alpha-1 chain (Fragment) | COL6A1 | 8.12E+06 | 3.96E+06 | 1.91E+06 | 2.25E+06 | 1.62E+06 | 7.92E+05 | 3.83E+05 | 4.49E+05 |
| **528** | F2Z5L7 | Proteasome subunit alpha type | PSMA1 | 2.89E+07 | 9.20E+06 | 1.02E+07 | 9.51E+06 | 1.60E+06 | 5.11E+05 | 5.64E+05 | 5.28E+05 |
| **529** | Q6FHV6 | ENO2 protein | ENO2 | 4.33E+07 | 1.24E+07 | 9.39E+06 | 2.15E+07 | 1.60E+06 | 4.59E+05 | 3.48E+05 | 7.96E+05 |
| **530** | F2Z536 | Uncharacterized protein | DYNLL2 | 7.98E+06 | 2.49E+06 | 2.41E+06 | 3.08E+06 | 1.60E+06 | 4.98E+05 | 4.82E+05 | 6.15E+05 |
| **531** | I3LII3 | Uncharacterized protein | EEF2 | 8.44E+07 | 4.68E+07 | 1.84E+07 | 1.91E+07 | 1.59E+06 | 8.84E+05 | 3.47E+05 | 3.61E+05 |
| **532** | Q2QLE2 | Caveolin-2 | CAV2 | 1.59E+07 | 1.13E+07 | 4.57E+06 | 0.00E+00 | 1.59E+06 | 1.13E+06 | 4.57E+05 | 0.00E+00 |
| **533** | F1SB57 | Uncharacterized protein | ARMC10 | 1.74E+07 | 1.00E+07 | 5.43E+06 | 2.00E+06 | 1.59E+06 | 9.10E+05 | 4.94E+05 | 1.82E+05 |
| **534** | A0A075B6X5 | Protein TRAV18 (Fragment) | TRAV18 | 1.27E+07 | 1.68E+06 | 2.95E+06 | 8.03E+06 | 1.58E+06 | 2.10E+05 | 3.69E+05 | 1.00E+06 |
| **535** | B2R5W2 | Heterogeneous nuclear ribonucleoproteins C1/C2 | HNRNPC | 3.63E+07 | 1.32E+07 | 1.16E+07 | 1.15E+07 | 1.58E+06 | 5.74E+05 | 5.03E+05 | 5.02E+05 |
| **536** | F5H1S8 | Malectin (Fragment) | MLEC | 1.90E+07 | 7.66E+06 | 4.63E+06 | 6.66E+06 | 1.58E+06 | 6.38E+05 | 3.86E+05 | 5.55E+05 |
| **537** | I3LQ17 | Uncharacterized protein | | 1.39E+08 | 5.96E+07 | 4.33E+07 | 3.57E+07 | 1.57E+06 | 6.77E+05 | 4.92E+05 | 4.06E+05 |
| **538** | E5RI99 | 60S ribosomal protein L30 (Fragment) | RPL30 | 1.26E+07 | 5.98E+06 | 3.96E+06 | 2.61E+06 | 1.57E+06 | 7.47E+05 | 4.95E+05 | 3.27E+05 |
| **539** | A0A075B7I9 | Uncharacterized protein (Fragment) | | 6.27E+06 | 1.02E+06 | 2.28E+06 | 2.97E+06 | 1.57E+06 | 2.55E+05 | 5.70E+05 | 7.42E+05 |
| **540** | F1SNL7 | Uncharacterized protein | CNDP2 | 4.21E+07 | 2.32E+07 | 4.79E+06 | 1.41E+07 | 1.56E+06 | 8.58E+05 | 1.77E+05 | 5.24E+05 |
| **541** | F1SHU0 | Uncharacterized protein | LOC100516841 | 1.09E+07 | 3.36E+06 | 3.29E+06 | 4.26E+06 | 1.56E+06 | 4.80E+05 | 4.70E+05 | 6.09E+05 |
| **542** | Q6FGB3 | PCBD protein (Fragment) | PCBD | 1.24E+07 | 5.58E+06 | 2.56E+06 | 4.21E+06 | 1.54E+06 | 6.97E+05 | 3.20E+05 | 5.27E+05 |
| **543** | Q4W5A6 | Putative uncharacterized protein KIAA0992 (Fragment) | KIAA0992 | 1.23E+07 | 5.96E+06 | 3.60E+06 | 2.78E+06 | 1.54E+06 | 7.46E+05 | 4.50E+05 | 3.47E+05 |
| **544** | Q9GKX6 | Aldose 1-epimerase | GALM | 2.93E+07 | 1.68E+07 | 7.21E+06 | 5.22E+06 | 1.54E+06 | 8.85E+05 | 3.79E+05 | 2.75E+05 |
| **545** | Q5EFJ2 | Alkali myosin light chain 3 A2 catalytic | MLC3F | 1.54E+07 | 5.49E+06 | 1.46E+06 | 8.43E+06 | 1.54E+06 | 5.49E+05 | 1.46E+05 | 8.43E+05 |
| **546** | A8D737 | T-cadherin | | 5.51E+07 | 2.57E+07 | 1.13E+07 | 1.81E+07 | 1.53E+06 | 7.14E+05 | 3.13E+05 | 5.02E+05 |
| **547** | F1SSL6 | Proteasome subunit alpha type | PSMA3 | 2.28E+07 | 9.87E+06 | 6.67E+06 | 6.28E+06 | 1.52E+06 | 6.58E+05 | 4.45E+05 | 4.18E+05 |
| **548** | K7GPQ7 | Uncharacterized protein (Fragment) | AGT | 1.37E+07 | 1.07E+06 | 3.49E+06 | 9.12E+06 | 1.52E+06 | 1.19E+05 | 3.88E+05 | 1.01E+06 |
| **549** | I3LIN8 | Core histone macro-H2A | H2AFY | 2.58E+07 | 9.33E+06 | 5.86E+06 | 1.06E+07 | 1.52E+06 | 5.49E+05 | 3.44E+05 | 6.24E+05 |
| **550** | I3L7P7 | Uncharacterized protein | ATOX1 | 6.01E+06 | 1.31E+06 | 1.59E+06 | 3.11E+06 | 1.50E+06 | 3.27E+05 | 3.98E+05 | 7.78E+05 |
| **551** | F2Z4Y0 | Uncharacterized protein | SNRPD3 | 1.35E+07 | 3.70E+06 | 4.07E+06 | 5.71E+06 | 1.50E+06 | 4.11E+05 | 4.53E+05 | 6.35E+05 |
| **552** | F1SFI6 | Uncharacterized protein | FETUB | 2.97E+07 | 7.27E+06 | 8.16E+06 | 1.43E+07 | 1.48E+06 | 3.63E+05 | 4.08E+05 | 7.13E+05 |
| **553** | Q6SEG5 | Ubiquitin carboxyl-terminal hydrolase isozyme L1 | UCHL1 | 1.92E+07 | 8.49E+06 | 1.74E+06 | 9.00E+06 | 1.48E+06 | 6.53E+05 | 1.34E+05 | 6.92E+05 |
| **554** | I3LR43 | Uncharacterized protein | | 1.47E+07 | 7.40E+06 | 4.00E+06 | 3.34E+06 | 1.47E+06 | 7.40E+05 | 4.00E+05 | 3.34E+05 |
| **555** | F1SLY7 | Uncharacterized protein | CNPY2 | 1.75E+07 | 6.60E+06 | 3.50E+06 | 7.44E+06 | 1.46E+06 | 5.50E+05 | 2.92E+05 | 6.20E+05 |
| **556** | Q9UJC5 | SH3 domain-binding glutamic acid-rich-like protein 2 | SH3BGRL2 | 1.16E+07 | 2.65E+06 | 4.16E+06 | 4.83E+06 | 1.45E+06 | 3.31E+05 | 5.21E+05 | 6.03E+05 |
| **557** | P79403 | Neutral alpha-glucosidase AB | GANAB | 7.97E+07 | 2.67E+07 | 2.49E+07 | 2.81E+07 | 1.45E+06 | 4.86E+05 | 4.52E+05 | 5.11E+05 |
| **558** | B0LY44 | Mitochondrial ornithine aminotransferase | OAT | 3.90E+07 | 1.61E+07 | 7.47E+06 | 1.54E+07 | 1.44E+06 | 5.97E+05 | 2.77E+05 | 5.70E+05 |
| **559** | V9HW38 | Epididymis secretory protein Li 106 | HEL-S-106 | 4.76E+07 | 1.25E+07 | 1.26E+07 | 2.25E+07 | 1.44E+06 | 3.78E+05 | 3.82E+05 | 6.83E+05 |
| **560** | A5A8W8 | Complement component 4A | C4A | 1.32E+08 | 3.73E+07 | 3.50E+07 | 6.01E+07 | 1.42E+06 | 4.01E+05 | 3.77E+05 | 6.47E+05 |
| **561** | B7Z478 | Proteasome (Prosome, macropain) subunit, beta type, 2, isoform CRA | PSMB2 | 1.56E+07 | 4.15E+06 | 2.66E+06 | 8.83E+06 | 1.42E+06 | 3.77E+05 | 2.42E+05 | 8.03E+05 |
| **562** | X6RI56 | CSC1-like protein 1 (Fragment) | TMEM63A | 8.51E+06 | 0.00E+00 | 2.69E+06 | 5.82E+06 | 1.42E+06 | 0.00E+00 | 4.49E+05 | 9.70E+05 |
| **563** | F1SSH8 | Uncharacterized protein | OGDH | 7.09E+07 | 2.29E+07 | 1.67E+07 | 3.13E+07 | 1.42E+06 | 4.57E+05 | 3.35E+05 | 6.26E+05 |
| **564** | I3LRP7 | Uncharacterized protein | LOC100626941 | 2.27E+07 | 1.12E+07 | 6.20E+06 | 5.26E+06 | 1.42E+06 | 7.02E+05 | 3.88E+05 | 3.29E+05 |
| **565** | I3LN84 | Uncharacterized protein | LXN | 1.13E+07 | 4.00E+06 | 4.11E+06 | 3.20E+06 | 1.41E+06 | 5.00E+05 | 5.14E+05 | 4.00E+05 |
| **566** | Q56P20 | ADP-ribosylation factor 4 | ARF4 | 1.55E+07 | 7.92E+06 | 3.62E+06 | 3.92E+06 | 1.41E+06 | 7.20E+05 | 3.29E+05 | 3.56E+05 |
| **567** | E0YLM4 | Mesencephalic astrocyte-derived neurotrophic factor | MANF | 1.53E+07 | 7.69E+06 | 3.62E+06 | 4.03E+06 | 1.39E+06 | 6.99E+05 | 3.29E+05 | 3.66E+05 |
| **568** | F1RPR7 | Integrin beta | ITGB6 | 1.67E+07 | 7.32E+06 | 4.38E+06 | 5.02E+06 | 1.39E+06 | 6.10E+05 | 3.65E+05 | 4.18E+05 |
| **569** | I3L8I5 | Eukaryotic translation initiation factor 6 | EIF6 | 1.38E+07 | 7.49E+06 | 2.69E+06 | 3.62E+06 | 1.38E+06 | 7.49E+05 | 2.69E+05 | 3.62E+05 |
| **570** | O18734 | Protein phosphatase 1 regulatory subunit 14A | CPI17 | 9.64E+06 | 1.99E+06 | 2.40E+06 | 5.25E+06 | 1.38E+06 | 2.85E+05 | 3.42E+05 | 7.50E+05 |
| **571** | F1SH92 | Inter-alpha-trypsin inhibitor heavy chain H4 | ITIH4 | 5.78E+07 | 1.59E+07 | 1.53E+07 | 2.67E+07 | 1.38E+06 | 3.78E+05 | 3.64E+05 | 6.35E+05 |
| **572** | Q2EN79 | Ubiquinol-cytochrome c reductase complex | UQCR10 | 5.49E+06 | 5.96E+05 | 6.42E+05 | 4.25E+06 | 1.37E+06 | 1.49E+05 | 1.61E+05 | 1.06E+06 |
| **573** | Q15746 | Myosin light chain kinase, smooth muscle | MYLK | 1.58E+08 | 6.04E+07 | 4.54E+07 | 5.17E+07 | 1.37E+06 | 5.25E+05 | 3.95E+05 | 4.50E+05 |
| **574** | B5APV0 | Actin-related protein 2/3 complex subunit 5 | ARPC5 | 1.64E+07 | 9.78E+06 | 2.60E+05 | 6.39E+06 | 1.37E+06 | 8.15E+05 | 2.16E+04 | 5.33E+05 |
| **575** | K7ERG4 | Small nuclear ribonucleoprotein Sm D2 | SNRPD2 | 6.84E+06 | 0.00E+00 | 1.47E+06 | 5.37E+06 | 1.37E+06 | 0.00E+00 | 2.93E+05 | 1.07E+06 |
| **576** | F1RQU2 | Uncharacterized protein | HSP90AB1 | 5.15E+07 | 2.78E+07 | 8.18E+06 | 1.55E+07 | 1.35E+06 | 7.32E+05 | 2.15E+05 | 4.07E+05 |
| **577** | Q9TV62 | Myosin-4 | MYH4 | 1.73E+08 | 1.03E+08 | 4.81E+07 | 2.18E+07 | 1.35E+06 | 8.04E+05 | 3.76E+05 | 1.70E+05 |
| **578** | B4E3E6 | cDNA FLJ58832, highly similar to Heterogeneous nuclear ribonucleoprotein A3 | HNRPA3 | 2.28E+07 | 1.01E+07 | 7.89E+06 | 4.75E+06 | 1.34E+06 | 5.97E+05 | 4.64E+05 | 2.79E+05 |
| **579** | F1SPG1 | Uncharacterized protein | H1FX | 1.74E+07 | 4.27E+06 | 5.71E+06 | 7.41E+06 | 1.34E+06 | 3.28E+05 | 4.39E+05 | 5.70E+05 |
| **580** | Q007T0 | Succinate dehydrogenase [ubiquinone] iron-sulfur subunit, mitochondrial | SDHB | 2.79E+07 | 1.48E+07 | 8.99E+06 | 4.09E+06 | 1.33E+06 | 7.04E+05 | 4.28E+05 | 1.95E+05 |
| **581** | V9HW53 | Dimethylarginine dimethylaminohydrolase 2, isoform CRA | HEL-S-277 | 2.39E+07 | 8.75E+06 | 6.35E+06 | 8.76E+06 | 1.33E+06 | 4.86E+05 | 3.53E+05 | 4.87E+05 |
| **582** | I3LEE6 | Uncharacterized protein | PCOLCE | 3.30E+07 | 2.21E+07 | 8.88E+06 | 2.09E+06 | 1.32E+06 | 8.82E+05 | 3.55E+05 | 8.35E+04 |
| **583** | O19069 | Succinyl-CoA ligase [ADP/GDP-forming] subunit alpha, mitochondrial | SUCLG1 | 1.83E+07 | 1.19E+07 | 3.57E+06 | 2.77E+06 | 1.31E+06 | 8.53E+05 | 2.55E+05 | 1.98E+05 |
| **584** | J3KQA0 | Synaptotagmin I, isoform CRA | SYT1 | 2.98E+07 | 1.03E+07 | 7.96E+06 | 1.16E+07 | 1.30E+06 | 4.48E+05 | 3.46E+05 | 5.03E+05 |
| **585** | F1SU97 | Saposin-B-Val | PSAP | 3.89E+07 | 8.86E+06 | 1.31E+07 | 1.69E+07 | 1.30E+06 | 2.95E+05 | 4.38E+05 | 5.63E+05 |
| **586** | W5QT72 | ATP synthase subunit a | ATP6 | 2.58E+06 | 2.25E+06 | 1.52E+05 | 1.72E+05 | 1.29E+06 | 1.13E+06 | 7.62E+04 | 8.62E+04 |
| **587** | B8ZZF8 | U6 snRNA-associated Sm-like protein LSm5 | LSM5 | 5.08E+06 | 1.41E+06 | 1.81E+06 | 1.86E+06 | 1.27E+06 | 3.52E+05 | 4.53E+05 | 4.66E+05 |
| **588** | I3LPP1 | Fumarate hydratase, mitochondrial | FH | 3.79E+07 | 1.69E+07 | 1.01E+07 | 1.09E+07 | 1.26E+06 | 5.63E+05 | 3.38E+05 | 3.62E+05 |

**Supplementary Table S1: (continued)**

| | | | | | | | | | | | |
|---|---|---|---|---|---|---|---|---|---|---|---|
| **589** | Q53F20 | Acidic (Leucine-rich) nuclear phosphoprotein 32 family, member E variant (Fragment) | ANP32E | 1.25E+07 | 4.71E+06 | 2.72E+06 | 5.10E+06 | 1.25E+06 | 4.71E+05 | 2.72E+05 | 5.10E+05 |
| **590** | K7EM02 | Katanin p60 ATPase-containing subunit A-like 2 (Fragment) | KATNAL2 | 1.13E+07 | 1.72E+06 | 4.25E+06 | 5.30E+06 | 1.25E+06 | 1.91E+05 | 4.72E+05 | 5.89E+05 |
| **591** | F1S9Q3 | Heat shock 70 kDa protein 1B | HSPA1B | 4.76E+07 | 1.39E+07 | 1.50E+07 | 1.87E+07 | 1.25E+06 | 3.66E+05 | 3.95E+05 | 4.91E+05 |
| **592** | A1XQS3 | Mitochondrial NDUFA4 | NDUFA4 | 7.50E+06 | 4.26E+06 | 2.21E+06 | 1.03E+06 | 1.25E+06 | 7.11E+05 | 3.68E+05 | 1.72E+05 |
| **593** | F1SB33 | Uncharacterized protein | SYPL1 | 1.24E+07 | 5.38E+06 | 3.83E+06 | 3.23E+06 | 1.24E+06 | 5.38E+05 | 3.83E+05 | 3.23E+05 |
| **594** | F1RM51 | Glucosamine-6-phosphate isomerase | GNPDA1 | 2.11E+07 | 7.66E+06 | 5.92E+06 | 7.51E+06 | 1.24E+06 | 4.50E+05 | 3.48E+05 | 4.42E+05 |
| **595** | F1S9H9 | Uncharacterized protein | | 1.24E+07 | 4.84E+06 | 3.79E+06 | 3.75E+06 | 1.24E+06 | 4.84E+05 | 3.79E+05 | 3.75E+05 |
| **596** | I3LDC7 | Isocitrate dehydrogenase [NADP] | IDH1 | 3.83E+07 | 1.66E+07 | 7.16E+06 | 1.46E+07 | 1.24E+06 | 5.34E+05 | 2.31E+05 | 4.71E+05 |
| **597** | P46405 | 40S ribosomal protein S12 | RPS12 | 8.59E+06 | 1.45E+06 | 5.82E+06 | 1.32E+06 | 1.23E+06 | 2.07E+05 | 8.32E+05 | 1.88E+05 |
| **598** | Q5TCU3 | Tropomyosin beta chain | TPM2 | 2.81E+07 | 1.35E+07 | 5.12E+06 | 9.44E+06 | 1.22E+06 | 5.88E+05 | 2.23E+05 | 4.10E+05 |
| **599** | K7ENX8 | Cold-inducible RNA-binding protein (Fragment) | CIRBP | 6.09E+06 | 1.71E+06 | 2.82E+06 | 1.56E+06 | 1.22E+06 | 3.41E+05 | 5.65E+05 | 3.11E+05 |
| **600** | F1SL37 | Leukocyte surface antigen CD47 | CD47 | 1.34E+07 | 4.21E+06 | 3.16E+06 | 6.01E+06 | 1.22E+06 | 3.82E+05 | 2.87E+05 | 5.46E+05 |
| **601** | F1RKY2 | Uncharacterized protein | SERPIND1 | 3.26E+07 | 1.53E+07 | 1.04E+07 | 6.98E+06 | 1.21E+06 | 5.67E+05 | 3.84E+05 | 2.59E+05 |
| **602** | F1SMF6 | Uncharacterized protein | ITGA1 | 7.20E+07 | 3.11E+07 | 1.81E+07 | 2.27E+07 | 1.20E+06 | 5.19E+05 | 3.02E+05 | 3.79E+05 |
| **603** | A2A2D0 | Stathmin (Fragment) | STMN1 | 7.19E+06 | 1.89E+06 | 1.64E+06 | 3.66E+06 | 1.20E+06 | 3.16E+05 | 2.73E+05 | 6.10E+05 |
| **604** | K9IWF9 | Ras GTPase-activating-like protein IQGAP1 | IQGAP1 | 1.12E+08 | 5.54E+07 | 2.01E+07 | 3.70E+07 | 1.20E+06 | 5.89E+05 | 2.14E+05 | 3.93E+05 |
| **605** | F1RIA7 | Uncharacterized protein | TMEM109 | 1.20E+07 | 5.80E+06 | 2.78E+06 | 3.38E+06 | 1.20E+06 | 5.80E+05 | 2.78E+05 | 3.38E+05 |
| **606** | F1SK45 | GTP:AMP phosphotransferase AK3, mitochondrial | AK3 | 2.27E+07 | 1.30E+07 | 4.34E+06 | 5.37E+06 | 1.19E+06 | 6.84E+05 | 2.28E+05 | 2.83E+05 |
| **607** | H6VRG2 | Keratin 1 | KRT1 | 4.17E+07 | 1.26E+07 | 1.08E+07 | 1.84E+07 | 1.19E+06 | 3.59E+05 | 3.08E+05 | 5.26E+05 |
| **608** | Q29052 | Inter-alpha-trypsin inhibitor heavy chain H1 | ITIH1 | 5.95E+07 | 2.11E+07 | 1.95E+07 | 1.89E+07 | 1.19E+06 | 4.22E+05 | 3.90E+05 | 3.78E+05 |
| **609** | A0A024RB09 | RAB5B, member RAS oncogene family, isoform CRA | RAB5B | 1.66E+07 | 7.00E+06 | 3.55E+06 | 6.10E+06 | 1.19E+06 | 5.00E+05 | 2.53E+05 | 4.36E+05 |
| **610** | C9JMW4 | Protein IQCJ-SCHIP1 (Fragment) | IQCJ-SCHIP1 | 7.12E+06 | 1.96E+06 | 3.17E+06 | 2.00E+06 | 1.19E+06 | 3.26E+05 | 5.29E+05 | 3.33E+05 |
| **611** | F1SM07 | Uncharacterized protein | COPS8 | 1.07E+07 | 1.34E+06 | 4.24E+06 | 5.08E+06 | 1.18E+06 | 1.49E+05 | 4.71E+05 | 5.65E+05 |
| **612** | F1RU33 | Uncharacterized protein | BANF1 | 9.43E+06 | 3.29E+06 | 2.40E+06 | 3.74E+06 | 1.18E+06 | 4.11E+05 | 3.00E+05 | 4.68E+05 |
| **613** | F1RYS5 | Uncharacterized protein | SEPT11 | 3.06E+07 | 7.01E+06 | 8.28E+06 | 1.53E+07 | 1.18E+06 | 2.70E+05 | 3.19E+05 | 5.87E+05 |
| **614** | A8K6S3 | cDNA FLJ77570 | | 4.58E+07 | 1.95E+07 | 1.19E+07 | 1.44E+07 | 1.18E+06 | 5.00E+05 | 3.06E+05 | 3.70E+05 |
| **615** | P02662 | Alpha-S1-casein | | 1.29E+07 | 0.00E+00 | 0.00E+00 | 1.29E+07 | 1.17E+06 | 0.00E+00 | 0.00E+00 | 1.17E+06 |
| **616** | C1PIG4 | cAMP-dependent protein kinase regulatory subunit type II alpha | PRKAR2A | 2.58E+07 | 1.12E+07 | 5.40E+06 | 9.26E+06 | 1.17E+06 | 5.08E+05 | 2.45E+05 | 4.21E+05 |
| **617** | F1S2Q3 | Uncharacterized protein | TMED10 | 1.39E+07 | 2.26E+06 | 5.72E+06 | 5.94E+06 | 1.16E+06 | 1.88E+05 | 4.77E+05 | 4.95E+05 |
| **618** | G8FUN5 | Y box binding protein 1 (Fragment) | YBX1 | 1.15E+07 | 7.54E+06 | 3.66E+06 | 3.21E+05 | 1.15E+06 | 7.54E+05 | 3.66E+05 | 3.21E+04 |
| **619** | Q6IB24 | PDE6D protein | PDE6D | 5.73E+06 | 5.39E+06 | 3.40E+05 | 0.00E+00 | 1.15E+06 | 1.08E+06 | 6.81E+04 | 0.00E+00 |
| **620** | A0A023T787 | RNA-binding protein 8A | RBM8 | 9.16E+06 | 2.97E+06 | 1.11E+06 | 5.08E+06 | 1.15E+06 | 3.71E+05 | 1.38E+05 | 6.35E+05 |
| **621** | F8VSA6 | NEDD8 | NEDD8 | 3.42E+06 | 1.70E+06 | 6.08E+05 | 1.11E+06 | 1.14E+06 | 5.66E+05 | 2.03E+05 | 3.71E+05 |
| **622** | F1RGE3 | NADH dehydrogenase [ubiquinone] 1 alpha subcomplex subunit 2 | NDUFA2 | 9.08E+06 | 2.94E+06 | 2.70E+06 | 3.43E+06 | 1.14E+06 | 3.68E+05 | 3.38E+05 | 4.29E+05 |
| **623** | F1RPD4 | Uncharacterized protein | LOC100620271 | 5.67E+06 | 1.93E+06 | 1.02E+06 | 2.72E+06 | 1.13E+06 | 3.86E+05 | 2.04E+05 | 5.45E+05 |
| **624** | Q53GD8 | Peptidyl-prolyl cis-trans isomerase (Fragment) | FKBP3 | 1.59E+07 | 6.62E+06 | 5.72E+06 | 3.54E+06 | 1.13E+06 | 4.73E+05 | 4.09E+05 | 2.53E+05 |
| **625** | P27917 | Apolipoprotein C-III | APOC3 | 8.98E+06 | 4.76E+06 | 1.24E+06 | 2.97E+06 | 1.12E+06 | 5.95E+05 | 1.55E+05 | 3.72E+05 |
| **626** | Q8MHY0 | MYL2 | MLC-2V | 1.23E+07 | 3.44E+06 | 2.91E+06 | 5.97E+06 | 1.12E+06 | 3.13E+05 | 2.65E+05 | 5.43E+05 |
| **627** | I3L6A4 | Uncharacterized protein | | 8.88E+06 | 2.63E+06 | 2.91E+06 | 3.35E+06 | 1.11E+06 | 3.29E+05 | 3.63E+05 | 4.18E+05 |
| **628** | F1SQW8 | Uncharacterized protein | ARHGDIB | 1.53E+07 | 4.93E+06 | 4.94E+06 | 5.44E+06 | 1.09E+06 | 3.52E+05 | 3.53E+05 | 3.88E+05 |
| **629** | I3LG67 | Uncharacterized protein | MARCKS | 1.09E+07 | 8.41E+06 | 2.50E+06 | 0.00E+00 | 1.09E+06 | 8.41E+05 | 2.50E+05 | 0.00E+00 |
| **630** | V9HW65 | Annexin | HEL-S-270 | 2.72E+07 | 1.23E+07 | 5.94E+06 | 9.05E+06 | 1.09E+06 | 4.90E+05 | 2.38E+05 | 3.62E+05 |
| **631** | K7ZMG1 | Putative cysteine-rich protein 2 | CRIP2 | 1.41E+07 | 1.26E+07 | 5.97E+05 | 9.05E+05 | 1.08E+06 | 9.68E+05 | 4.59E+04 | 6.96E+04 |
| **632** | F8SIP2 | EGF-containing fibulin-like extracellular matrix protein 1 | EFEMP1 | 2.82E+07 | 8.53E+06 | 1.07E+07 | 8.94E+06 | 1.08E+06 | 3.28E+05 | 4.11E+05 | 3.44E+05 |
| **633** | V9HWI5 | Cofilin 1 (Non-muscle), isoform CRA | HEL-S-15 | 1.30E+07 | 3.38E+06 | 2.76E+06 | 6.83E+06 | 1.08E+06 | 2.82E+05 | 2.30E+05 | 5.69E+05 |
| **634** | F1RQ06 | Peptidyl-prolyl cis-trans isomerase | FKBP2 | 8.62E+06 | 4.11E+06 | 2.87E+06 | 1.64E+06 | 1.08E+06 | 5.13E+05 | 3.58E+05 | 2.05E+05 |
| **635** | A0A068F143 | Glutathione peroxidase | GPX1 | 1.62E+07 | 6.43E+06 | 2.52E+06 | 7.21E+06 | 1.08E+06 | 4.29E+05 | 1.68E+05 | 4.80E+05 |
| **636** | I3LAJ6 | Uncharacterized protein | RALA | 1.07E+07 | 6.73E+06 | 2.59E+06 | 1.42E+06 | 1.07E+06 | 6.73E+05 | 2.59E+05 | 1.42E+05 |
| **637** | F8VS02 | Alpha-aminoadipic semialdehyde dehydrogenase | ALDH7A1 | 2.47E+07 | 1.01E+07 | 5.64E+06 | 8.87E+06 | 1.07E+06 | 4.41E+05 | 2.45E+05 | 3.85E+05 |
| **638** | A8K8B9 | cDNA FLJ77368 | | 2.02E+07 | 4.36E+06 | 7.18E+06 | 8.69E+06 | 1.06E+06 | 2.29E+05 | 3.78E+05 | 4.58E+05 |
| **639** | A9LM01 | Progesterone receptor membrane component 2 | PGRMC2 | 1.28E+07 | 4.28E+06 | 2.33E+06 | 6.14E+06 | 1.06E+06 | 3.57E+05 | 1.94E+05 | 5.12E+05 |
| **640** | Q2EN81 | ATP synthase subunit O, mitochondrial | ATP5O | 1.36E+07 | 4.21E+06 | 3.47E+06 | 5.96E+06 | 1.05E+06 | 3.24E+05 | 2.67E+05 | 4.59E+05 |
| **641** | F1RNN0 | Vacuolar protein sorting-associated protein 29 | VPS29 | 1.15E+07 | 4.34E+06 | 2.54E+06 | 4.65E+06 | 1.05E+06 | 3.95E+05 | 2.31E+05 | 4.22E+05 |
| **642** | Q29024 | Spliceosome RNA helicase DDX39B | DDX39B | 2.51E+07 | 1.09E+07 | 6.99E+06 | 7.22E+06 | 1.04E+06 | 4.53E+05 | 2.91E+05 | 3.01E+05 |
| **643** | F1SEL7 | Retinol-binding protein 3 | RBP3 | 6.16E+07 | 4.39E+07 | 7.51E+06 | 1.02E+07 | 1.04E+06 | 7.43E+05 | 1.27E+05 | 1.73E+05 |
| **644** | I3LQ84 | Uncharacterized protein | COL6A2 | 5.84E+07 | 2.99E+07 | 1.38E+07 | 1.48E+07 | 1.04E+06 | 5.33E+05 | 2.46E+05 | 2.64E+05 |
| **645** | I3LR69 | Ferritin | FTH1 | 1.25E+07 | 5.56E+06 | 2.43E+06 | 4.54E+06 | 1.04E+06 | 4.63E+05 | 2.02E+05 | 3.78E+05 |
| **646** | B5APU3 | Actin-related protein 2-like protein | ACTR2 | 2.19E+07 | 1.60E+07 | 0.00E+00 | 5.91E+06 | 1.04E+06 | 7.61E+05 | 0.00E+00 | 2.81E+05 |
| **647** | B7Z361 | Reticulon | RTN3 | 7.26E+06 | 5.29E+06 | 0.00E+00 | 1.97E+06 | 1.04E+06 | 7.56E+05 | 0.00E+00 | 2.81E+05 |
| **648** | Q863Z0 | Proteasome activator complex subunit 2 | PSME2 | 1.86E+07 | 9.61E+06 | 4.64E+06 | 4.35E+06 | 1.03E+06 | 5.34E+05 | 2.58E+05 | 2.42E+05 |
| **649** | K9IWG6 | Spectrin beta chain, brain 1 (Fragment) | SPTBN1 | 1.65E+08 | 8.44E+07 | 4.62E+07 | 3.46E+07 | 1.03E+06 | 5.28E+05 | 2.89E+05 | 2.16E+05 |
| **650** | G3DRF8 | Proteasome subunit beta type | PSMB6 | 1.13E+07 | 3.12E+06 | 4.48E+06 | 3.74E+06 | 1.03E+06 | 2.84E+05 | 4.07E+05 | 3.40E+05 |
| **651** | I3L594 | Uncharacterized protein | EIF4H | 1.64E+07 | 6.35E+06 | 6.46E+06 | 3.63E+06 | 1.03E+06 | 3.97E+05 | 4.04E+05 | 2.27E+05 |
| **652** | A0A087X0T8 | Cell adhesion molecule 1 | CADM1 | 2.47E+07 | 8.07E+06 | 7.42E+06 | 9.16E+06 | 1.03E+06 | 3.36E+05 | 3.09E+05 | 3.82E+05 |
| **653** | F1SIP0 | Uncharacterized protein | SEPT 2 | 2.25E+07 | 1.15E+07 | 5.57E+06 | 5.41E+06 | 1.02E+06 | 5.23E+05 | 2.53E+05 | 2.46E+05 |
| **654** | A5YV76 | Fatty acid synthase | FASN | 1.39E+08 | 4.73E+07 | 4.51E+07 | 4.65E+07 | 1.02E+06 | 3.48E+05 | 3.32E+05 | 3.42E+05 |
| **655** | Q29582 | Pyruvate kinase M2 (Fragment) | | 5.09E+06 | 5.09E+06 | 0.00E+00 | 0.00E+00 | 1.02E+06 | 1.02E+06 | 0.00E+00 | 0.00E+00 |
| **656** | J3KS05 | Chromobox protein homolog 1 (Fragment) | CBX1 | 1.01E+07 | 4.93E+06 | 1.42E+06 | 3.79E+06 | 1.01E+06 | 4.93E+05 | 1.42E+05 | 3.79E+05 |
| **657** | F1RKU0 | Isocitrate dehydrogenase [NAD] subunit, mitochondrial | IDH3A | 2.33E+07 | 1.20E+07 | 5.73E+06 | 5.57E+06 | 1.01E+06 | 5.20E+05 | 2.49E+05 | 2.42E+05 |
| **658** | M3UZ42 | Ubiquitin-fold modifier 1 | UFM1 | 5.05E+06 | 1.45E+06 | 1.38E+06 | 2.22E+06 | 1.01E+06 | 2.89E+05 | 2.77E+05 | 4.44E+05 |
| **659** | A0A024R7R3 | Beta-synuclein (Fragment) | SNCB | 8.05E+06 | 4.35E+06 | 7.86E+05 | 2.92E+06 | 1.01E+06 | 5.43E+05 | 9.82E+04 | 3.65E+05 |
| **660** | P04574 | Calpain small subunit 1 | CAPNS1 | 1.40E+07 | 6.64E+06 | 2.87E+06 | 4.54E+06 | 1.00E+06 | 4.74E+05 | 2.05E+05 | 3.24E+05 |
| **661** | A4D110 | Putative uncharacterized protein | LOC401309 | 5.01E+06 | 9.61E+05 | 1.68E+06 | 2.37E+06 | 1.00E+06 | 1.92E+05 | 3.36E+05 | 4.75E+05 |
| **662** | Q53J08 | Cellular retinol binding protein 1 | CRBP1 | 1.19E+07 | 8.35E+06 | 6.21E+05 | 2.94E+06 | 9.93E+05 | 6.96E+05 | 5.17E+04 | 2.45E+05 |
| **663** | C0MHR2 | Clathrin heavy chain | CLTC | 9.71E+07 | 4.82E+07 | 2.56E+07 | 2.33E+07 | 9.91E+05 | 4.92E+05 | 2.61E+05 | 2.37E+05 |
| **664** | O02668 | Inter-alpha-trypsin inhibitor heavy chain H2 | ITIH2 | 5.34E+07 | 1.04E+07 | 2.05E+07 | 2.25E+07 | 9.90E+05 | 1.93E+05 | 3.79E+05 | 4.17E+05 |
| **665** | O77773 | Uncharacterized protein | CACNA2D1 | 5.64E+07 | 3.88E+07 | 8.07E+06 | 9.48E+06 | 9.89E+05 | 6.81E+05 | 1.42E+05 | 1.66E+05 |
| **666** | F1S663 | Uncharacterized protein | LAMC1 | 8.40E+07 | 2.79E+07 | 2.63E+07 | 2.98E+07 | 9.88E+05 | 3.28E+05 | 3.09E+05 | 3.51E+05 |
| **667** | F2Z5N8 | Ras-related protein Rab-14 | RAB14 | 1.58E+07 | 5.21E+06 | 3.22E+06 | 7.36E+06 | 9.87E+05 | 3.25E+05 | 2.01E+05 | 4.60E+05 |
| **668** | I3LD01 | Uncharacterized protein | FIS1 | 9.85E+06 | 3.72E+06 | 1.16E+06 | 4.98E+06 | 9.85E+05 | 3.72E+05 | 1.16E+05 | 4.98E+05 |
| **669** | A0A024QZV0 | HCG1811539, isoform CRA | hCG_1811539 | 1.97E+07 | 4.91E+06 | 4.19E+06 | 1.06E+07 | 9.83E+05 | 2.45E+05 | 2.09E+05 | 5.28E+05 |
| **670** | F2Z5V6 | Uncharacterized protein | LOC733637 | 1.08E+07 | 3.78E+06 | 3.75E+06 | 3.23E+06 | 9.79E+05 | 3.44E+05 | 3.41E+05 | 2.94E+05 |
| **671** | P50667 | Cytochrome c oxidase subunit 2 | MT-CO2 | 7.81E+06 | 3.75E+06 | 0.00E+00 | 4.06E+06 | 9.76E+05 | 4.69E+05 | 0.00E+00 | 5.07E+05 |
| **672** | A4D7T6 | Brain-type fatty acid-binding protein | fabp7 | 1.06E+07 | 5.14E+06 | 3.64E+06 | 1.86E+06 | 9.67E+05 | 4.67E+05 | 3.31E+05 | 1.69E+05 |
| **673** | Q0Z8U2 | 40S ribosomal protein S3 | RPS3 | 1.82E+07 | 6.69E+06 | 5.46E+06 | 6.02E+06 | 9.56E+05 | 3.52E+05 | 2.88E+05 | 3.17E+05 |
| **674** | B4DKS8 | cDNA FLJ57121 | HNRNPF | 1.88E+07 | 3.73E+06 | 5.36E+06 | 9.67E+06 | 9.38E+05 | 1.86E+05 | 2.68E+05 | 4.83E+05 |
| **675** | F1S765 | Uncharacterized protein | C1orf123 | 1.02E+07 | 3.45E+06 | 1.77E+06 | 4.95E+06 | 9.24E+05 | 3.13E+05 | 1.61E+05 | 4.50E+05 |
| **676** | A0PFK5 | Capping protein (Actin filament) muscle Z-line, alpha 1 | CP alpha1 | 1.48E+07 | 9.56E+06 | 3.57E+06 | 1.63E+06 | 9.22E+05 | 5.97E+05 | 2.23E+05 | 1.02E+05 |
| **677** | K7ZPU8 | IgG heavy chian constant region (Fragment) | IGHG5-1 | 1.56E+07 | 5.21E+06 | 4.38E+06 | 5.99E+06 | 9.16E+05 | 3.06E+05 | 2.57E+05 | 3.52E+05 |
| **678** | D6R9A6 | High mobility group protein B2 (Fragment) | HMGB2 | 7.32E+06 | 9.29E+05 | 2.31E+06 | 4.09E+06 | 9.15E+05 | 1.16E+05 | 2.88E+05 | 5.11E+05 |
| **679** | F1SLY2 | Uncharacterized protein | NDUFA5 | 6.36E+06 | 2.43E+06 | 3.11E+06 | 8.25E+05 | 9.09E+05 | 3.47E+05 | 4.44E+05 | 1.18E+05 |
| **680** | Q6IM77 | Reticulon | RTN2 | 7.19E+06 | 3.96E+06 | 1.35E+06 | 1.88E+06 | 8.99E+05 | 4.95E+05 | 1.68E+05 | 2.35E+05 |
| **681** | Q6FID4 | SARA1 protein | SARA1 | 9.87E+06 | 3.51E+06 | 3.03E+06 | 3.33E+06 | 8.97E+05 | 3.19E+05 | 2.75E+05 | 3.03E+05 |
| **682** | B4DQY1 | cDNA FLJ56133 | | 3.29E+07 | 8.36E+06 | 1.25E+07 | 1.20E+07 | 8.88E+05 | 2.26E+05 | 3.38E+05 | 3.24E+05 |
| **683** | F1RQI1 | Uncharacterized protein | COX7A2 | 4.43E+06 | 1.95E+06 | 6.27E+05 | 1.85E+06 | 8.87E+05 | 3.90E+05 | 1.25E+05 | 3.71E+05 |
| **684** | Q8MI72 | Complement regulator factor H | FH | 5.84E+07 | 3.91E+07 | 9.32E+06 | 1.00E+07 | 8.85E+05 | 5.92E+05 | 1.41E+05 | 1.52E+05 |
| **685** | P79280 | Myosin light chain kinase, smooth muscle (Fragment) | MYLK | 6.19E+06 | 9.51E+05 | 7.05E+05 | 4.54E+06 | 8.85E+05 | 1.36E+05 | 1.01E+05 | 6.48E+05 |
| **686** | F2Z539 | 40S ribosomal protein S20 | RPS20 | 5.29E+06 | 2.98E+06 | 1.30E+06 | 1.01E+06 | 8.81E+05 | 4.96E+05 | 2.16E+05 | 1.69E+05 |
| **687** | F1SI85 | Phosphoinositide phospholipase C | PLCL1 | 4.49E+07 | 1.25E+07 | 1.22E+07 | 2.02E+07 | 8.79E+05 | 2.45E+05 | 2.39E+05 | 3.95E+05 |
| **688** | I3LRS5 | Uncharacterized protein | ALDH1A1 | 1.32E+07 | 3.79E+06 | 4.29E+06 | 5.09E+06 | 8.78E+05 | 2.52E+05 | 2.86E+05 | 3.39E+05 |
| **689** | A0A0A0MR02 | Voltage-dependent anion-selective channel protein 2 (Fragment) | VDAC2 | 1.32E+07 | 8.72E+06 | 1.88E+06 | 2.56E+06 | 8.77E+05 | 5.81E+05 | 1.25E+05 | 1.70E+05 |
| **690** | F1SIF2 | Uncharacterized protein | NDUFS3 | 1.75E+07 | 1.01E+07 | 5.02E+06 | 2.42E+06 | 8.76E+05 | 5.04E+05 | 2.51E+05 | 1.21E+05 |
| **691** | I3LS73 | Uncharacterized protein | TIMM13 | 5.24E+06 | 9.89E+05 | 2.23E+06 | 2.01E+06 | 8.73E+05 | 1.65E+05 | 3.72E+05 | 3.36E+05 |
| **692** | F1S9C9 | Proteasome subunit beta type | PSMB5 | 1.65E+07 | 4.74E+06 | 5.00E+06 | 6.72E+06 | 8.66E+05 | 2.49E+05 | 2.63E+05 | 3.54E+05 |
| **693** | F1SM72 | Uncharacterized protein | NCAM1 | 4.41E+07 | 2.05E+07 | 1.05E+07 | 1.32E+07 | 8.66E+05 | 4.02E+05 | 2.06E+05 | 2.58E+05 |
| **694** | Q15124 | Phosphoglucomutase-like protein 5 | PGM5 | 2.89E+07 | 8.58E+06 | 6.03E+06 | 1.43E+07 | 8.49E+05 | 2.52E+05 | 1.77E+05 | 4.19E+05 |
| **695** | P35750 | Calpain-1 catalytic subunit | CAPN1 | 3.37E+07 | 1.01E+07 | 9.09E+06 | 1.45E+07 | 8.42E+05 | 2.52E+05 | 2.27E+05 | 3.62E+05 |
| **696** | F1SUG4 | Uncharacterized protein | TCEB2 | 9.26E+06 | 5.55E+06 | 5.67E+05 | 3.14E+06 | 8.42E+05 | 5.04E+05 | 5.15E+04 | 2.86E+05 |
| **697** | F1RLB6 | Uncharacterized protein | NAPA | 1.76E+07 | 8.90E+06 | 3.74E+06 | 4.96E+06 | 8.38E+05 | 4.24E+05 | 1.78E+05 | 2.36E+05 |
| **698** | Q96MT4 | Uncharacterized protein encoded by LINC01600 | LINC01600 | 5.86E+06 | 5.86E+06 | 0.00E+00 | 0.00E+00 | 8.37E+05 | 8.37E+05 | 0.00E+00 | 0.00E+00 |
| **699** | P04366 | Protein AMBP (Fragment) | AMBP | 1.59E+07 | 7.36E+06 | 4.21E+06 | 4.30E+06 | 8.35E+05 | 3.87E+05 | 2.22E+05 | 2.26E+05 |
| **700** | F8WBT4 | Ribulose-phosphate 3-epimerase | RPE | 3.34E+06 | 7.93E+05 | 1.08E+06 | 1.47E+06 | 8.34E+05 | 1.98E+05 | 2.69E+05 | 3.67E+05 |
| **701** | B4DF38 | cDNA FLJ52123 | | 8.32E+06 | 3.19E+06 | 2.26E+06 | 2.87E+06 | 8.32E+05 | 3.19E+05 | 2.26E+05 | 2.87E+05 |
| **702** | Q8WVC2 | 40S ribosomal protein S21 | RPS21 | 5.78E+06 | 2.25E+06 | 1.32E+06 | 2.21E+06 | 8.26E+05 | 3.21E+05 | 1.89E+05 | 3.16E+05 |
| **703** | F1RMZ8 | Uncharacterized protein | LOC106504095 | 2.37E+07 | 9.68E+06 | 6.77E+06 | 7.28E+06 | 8.18E+05 | 3.34E+05 | 2.33E+05 | 2.51E+05 |
| **704** | I3L728 | Uncharacterized protein | | 4.91E+06 | 1.99E+06 | 1.15E+06 | 1.76E+06 | 8.18E+05 | 3.32E+05 | 1.92E+05 | 2.93E+05 |
| **705** | I3L656 | Uncharacterized protein | NUDT5 | 9.79E+06 | 8.53E+06 | 9.15E+05 | 3.48E+05 | 8.16E+05 | 7.11E+05 | 7.63E+04 | 2.90E+04 |
| **706** | V9HW48 | SH3 domain-binding glutamic acid-rich-like protein | HEL-S-115 | 7.33E+06 | 3.73E+06 | 1.55E+06 | 2.05E+06 | 8.14E+05 | 4.15E+05 | 1.72E+05 | 2.27E+05 |
| **707** | F1RRX1 | Uncharacterized protein | LCN2 | 9.72E+06 | 3.98E+06 | 2.10E+06 | 3.65E+06 | 8.10E+05 | 3.32E+05 | 1.75E+05 | 3.04E+05 |
| **708** | B4DQT8 | cDNA FLJ61158 | | 9.70E+06 | 3.67E+06 | 1.58E+06 | 4.45E+06 | 8.09E+05 | 3.06E+05 | 1.32E+05 | 3.71E+05 |
| **709** | P02668 | Kappa-casein | | 5.65E+06 | 0.00E+00 | 0.00E+00 | 5.65E+06 | 8.07E+05 | 0.00E+00 | 0.00E+00 | 8.07E+05 |
| **710** | B2CZF8 | Lactadherin | MFGE8 | 2.10E+07 | 1.41E+07 | 4.71E+06 | 2.15E+06 | 8.07E+05 | 5.43E+05 | 1.81E+05 | 8.28E+04 |
| **711** | Q6UAQ9 | Electron transfer flavoprotein alpha subunit (Fragment) | ETFA | 1.69E+07 | 9.30E+06 | 3.20E+06 | 4.39E+06 | 8.04E+05 | 4.43E+05 | 1.52E+05 | 2.09E+05 |
| **712** | Q2HYU2 | ATP-dependent 6-phosphofructokinase, muscle type | PFKM | 3.19E+07 | 6.82E+06 | 9.02E+06 | 1.61E+07 | 7.98E+05 | 1.71E+05 | 2.26E+05 | 4.02E+05 |
| **713** | O46560 | Pyridoxal kinase | PDXK | 1.43E+07 | 6.40E+06 | 3.72E+06 | 4.22E+06 | 7.97E+05 | 3.56E+05 | 2.07E+05 | 2.34E+05 |
| **714** | Q56P28 | PRA1 family protein 3 | ARL6IP5 | 3.98E+06 | 3.98E+06 | 0.00E+00 | 0.00E+00 | 7.96E+05 | 7.96E+05 | 0.00E+00 | 0.00E+00 |
| **715** | F1RUL6 | C-X-C motif chemokine | LOC100524373 | 4.76E+06 | 1.11E+06 | 2.45E+06 | 1.20E+06 | 7.94E+05 | 1.85E+05 | 4.09E+05 | 2.00E+05 |
| **716** | Q8IUL9 | Hemoglobin beta chain variant Hb.Sinai-Bel Air (Fragment) | HBB | 6.33E+06 | 6.33E+06 | 0.00E+00 | 0.00E+00 | 7.91E+05 | 7.91E+05 | 0.00E+00 | 0.00E+00 |
| **717** | F5H895 | Dolichyl-diphosphooligosaccharide--protein glycosyltransferase subunit DAD1 | DAD1 | 1.58E+06 | 5.42E+05 | 5.07E+05 | 5.30E+05 | 7.89E+05 | 2.71E+05 | 2.54E+05 | 2.65E+05 |
| **718** | F2Z5G8 | Uncharacterized protein | RPS25 | 4.73E+06 | 1.18E+06 | 1.72E+06 | 1.83E+06 | 7.88E+05 | 1.97E+05 | 2.86E+05 | 3.05E+05 |
| **719** | V9HW45 | Epididymis secretory protein Li 302 | HEL-S-302 | 1.18E+07 | 4.00E+06 | 1.85E+06 | 5.94E+06 | 7.86E+05 | 2.66E+05 | 1.23E+05 | 3.96E+05 |
| **720** | H0Y6E7 | RNA-binding motif protein, X chromosome (Fragment) | RBMX | 1.96E+07 | 4.59E+06 | 6.85E+06 | 8.18E+06 | 7.85E+05 | 1.84E+05 | 2.74E+05 | 3.27E+05 |
| **721** | B3VFB6 | Calbindin 1 | CALB1 | 1.18E+07 | 6.04E+06 | 4.84E+06 | 8.85E+05 | 7.84E+05 | 4.03E+05 | 3.22E+05 | 5.90E+04 |
| **722** | I3LRS8 | Uncharacterized protein | PITPNA | 2.04E+07 | 8.93E+06 | 4.79E+06 | 6.66E+06 | 7.84E+05 | 3.43E+05 | 1.84E+05 | 2.56E+05 |
| **723** | Q58D68 | Ectonucleotide pyrophosphatase/phosphodiesterase family member 6 | ENPP6 | 2.19E+07 | 1.00E+07 | 4.74E+06 | 7.19E+06 | 7.84E+05 | 3.57E+05 | 1.69E+05 | 2.57E+05 |
| **724** | F1SBN7 | Uncharacterized protein | ATP5F1 | 1.33E+07 | 3.78E+06 | 2.98E+06 | 6.54E+06 | 7.83E+05 | 2.23E+05 | 1.75E+05 | 3.85E+05 |
| **725** | Q08ES8 | Cell growth-inhibiting protein 34 | | 7.79E+06 | 3.07E+06 | 9.20E+05 | 3.80E+06 | 7.79E+05 | 3.07E+05 | 9.20E+04 | 3.80E+05 |
| **726** | B9W5V0 | Elongation factor 1-alpha | EEF1A2 | 1.93E+07 | 6.75E+06 | 6.56E+06 | 6.01E+06 | 7.73E+05 | 2.70E+05 | 2.63E+05 | 2.40E+05 |
| **727** | P02475 | Alpha-crystallin A chain | CRYAA | 9.23E+06 | 2.56E+06 | 1.92E+06 | 4.76E+06 | 7.70E+05 | 2.13E+05 | 1.60E+05 | 3.97E+05 |
| **728** | I3LP02 | Uncharacterized protein | ACAT1 | 1.76E+07 | 6.91E+06 | 5.03E+06 | 5.70E+06 | 7.67E+05 | 3.00E+05 | 2.19E+05 | 2.48E+05 |
| **729** | F1RSG9 | Uncharacterized protein | LYPLA1 | 7.65E+06 | 2.08E+06 | 2.98E+06 | 2.58E+06 | 7.65E+05 | 2.08E+05 | 2.98E+05 | 2.58E+05 |
| **730** | A0A024QZ42 | HCG1985580, isoform CRA | PDCD6 | 6.10E+06 | 2.43E+06 | 1.59E+06 | 2.09E+06 | 7.63E+05 | 3.04E+05 | 1.98E+05 | 2.61E+05 |
| **731** | S4R371 | Fatty acid-binding protein, heart (Fragment) | FABP3 | 1.07E+07 | 6.39E+06 | 2.27E+06 | 2.02E+06 | 7.62E+05 | 4.56E+05 | 1.62E+05 | 1.44E+05 |
| **732** | A0A140VK94 | RAN binding protein 1, isoform CRA | RANBP1 | 8.33E+06 | 3.63E+06 | 3.18E+06 | 1.52E+06 | 7.57E+05 | 3.30E+05 | 2.89E+05 | 1.38E+05 |
| **733** | F1SKF9 | Uncharacterized protein | LOC100519091 | 1.81E+07 | 6.99E+06 | 4.80E+06 | 6.31E+06 | 7.54E+05 | 2.91E+05 | 2.00E+05 | 2.63E+05 |
| **734** | Q68RU1 | Ovarian and testicular apolipoprotein N | ApoN | 9.78E+06 | 1.11E+06 | 3.03E+06 | 5.64E+06 | 7.52E+05 | 8.52E+04 | 2.33E+05 | 4.34E+05 |
| **735** | Q5RLJ0 | CLE | | 1.35E+07 | 5.34E+06 | 6.07E+06 | 2.12E+06 | 7.52E+05 | 2.97E+05 | 3.37E+05 | 1.18E+05 |
| **736** | B4DMT4 | cDNA FLJ52695, highly similar to Alpha-centractin | | 1.28E+07 | 3.77E+06 | 5.18E+06 | 3.82E+06 | 7.51E+05 | 2.22E+05 | 3.05E+05 | 2.24E+05 |

**Supplementary Table S1: (continued)**

| | | | | | | | | | | | |
|---|---|---|---|---|---|---|---|---|---|---|---|
| **737** | D0G7F1 | Sterol carrier protein 2 | SCP2 | 2.48E+07 | 2.05E+06 | 0.00E+00 | 2.27E+07 | 7.50E+05 | 6.22E+04 | 0.00E+00 | 6.88E+05 |
| **738** | V9HW83 | Aldehyde dehydrogenase 1 family, member A1, isoform CRA | HEL-S-53e | 1.87E+07 | 4.70E+06 | 6.96E+06 | 7.01E+06 | 7.47E+05 | 1.88E+05 | 2.78E+05 | 2.80E+05 |
| **739** | I3LUR7 | Uncharacterized protein | COL6A3 | 1.42E+08 | 7.11E+07 | 3.21E+07 | 3.92E+07 | 7.41E+05 | 3.70E+05 | 1.67E+05 | 2.04E+05 |
| **740** | Q9N0F1 | Dihydrolipoyllysine-residue succinyltransferase component of 2-oxoglutarate dehydrogenase complex, mitochondrial | DLST | 1.93E+07 | 6.83E+06 | 5.13E+06 | 7.31E+06 | 7.41E+05 | 2.63E+05 | 1.97E+05 | 2.81E+05 |
| **741** | B5APU6 | Actin related protein 2/3 complex subunit 1B | ARPC1B | 1.48E+07 | 1.05E+07 | 2.91E+06 | 1.36E+06 | 7.41E+05 | 5.27E+05 | 1.46E+05 | 6.78E+04 |
| **742** | B4DI38 | Adenylyl cyclase-associated protein | CAP1 | 2.44E+07 | 1.17E+07 | 3.67E+06 | 9.02E+06 | 7.40E+05 | 3.55E+05 | 1.11E+05 | 2.73E+05 |
| **743** | K7GMM2 | Uncharacterized protein (Fragment) | NCALD | 1.04E+07 | 5.00E+06 | 1.44E+06 | 3.92E+06 | 7.39E+05 | 3.57E+05 | 1.03E+05 | 2.80E+05 |
| **744** | F1RKJ9 | Uncharacterized protein | ISOC1 | 1.18E+07 | 3.69E+06 | 3.60E+06 | 4.54E+06 | 7.39E+05 | 2.30E+05 | 2.25E+05 | 2.84E+05 |
| **745** | B6DX83 | V-crk sarcoma virus CT10 oncogene-like protein isoform b | CRK | 1.11E+07 | 6.15E+06 | 1.81E+06 | 3.12E+06 | 7.38E+05 | 4.10E+05 | 1.21E+05 | 2.08E+05 |
| **746** | F1RVL5 | Uncharacterized protein | LOC100523824 | 5.45E+07 | 2.96E+07 | 1.29E+07 | 1.20E+07 | 7.37E+05 | 4.00E+05 | 1.75E+05 | 1.62E+05 |
| **747** | Q6ICN0 | GRB2 protein (Fragment) | GRB2 | 1.40E+07 | 5.38E+06 | 2.18E+06 | 6.40E+06 | 7.35E+05 | 2.83E+05 | 1.15E+05 | 3.37E+05 |
| **748** | I3LDY2 | Uncharacterized protein | LOC100739304 | 1.02E+07 | 3.40E+06 | 2.83E+06 | 4.02E+06 | 7.32E+05 | 2.43E+05 | 2.02E+05 | 2.87E+05 |
| **749** | E5RK64 | Vesicle-associated membrane protein-associated protein B/C | VAPB | 2.92E+06 | 1.19E+06 | 6.82E+05 | 1.05E+06 | 7.29E+05 | 2.97E+05 | 1.71E+05 | 2.62E+05 |
| **750** | B7Z5Q2 | cDNA FLJ58075, highly similar to Ceruloplasmin (EC 1.16.3.1) | | 3.64E+07 | 2.23E+07 | 6.62E+06 | 7.55E+06 | 7.29E+05 | 4.45E+05 | 1.32E+05 | 1.51E+05 |
| **751** | M0QXH0 | Thioredoxin, mitochondrial | TXN2 | 2.18E+06 | 0.00E+00 | 8.30E+05 | 1.35E+06 | 7.28E+05 | 0.00E+00 | 2.77E+05 | 4.51E+05 |
| **752** | F1SEN8 | Uncharacterized protein | LDB3 | 2.31E+07 | 9.52E+06 | 5.66E+06 | 7.97E+06 | 7.23E+05 | 2.97E+05 | 1.77E+05 | 2.49E+05 |
| **753** | A2IPI6 | HRV Fab 027-VL (Fragment) | | 4.33E+06 | 4.33E+06 | 0.00E+00 | 0.00E+00 | 7.21E+05 | 7.21E+05 | 0.00E+00 | 0.00E+00 |
| **754** | F5H7R9 | Parathymosin (Fragment) | PTMS | 2.16E+06 | 0.00E+00 | 0.00E+00 | 2.16E+06 | 7.20E+05 | 0.00E+00 | 0.00E+00 | 7.20E+05 |
| **755** | P62760 | Visinin-like protein 1 | VSNL1 | 1.08E+07 | 2.98E+06 | 2.68E+06 | 5.14E+06 | 7.20E+05 | 1.98E+05 | 1.79E+05 | 3.43E+05 |
| **756** | I3LH71 | Uncharacterized protein | LOC100511115 | 1.43E+07 | 9.54E+06 | 4.77E+06 | 0.00E+00 | 7.16E+05 | 4.77E+05 | 2.39E+05 | 0.00E+00 |
| **757** | F1S0C1 | S-(hydroxymethyl)glutathione dehydrogenase | ADH5 | 1.57E+07 | 1.24E+07 | 1.23E+06 | 2.10E+06 | 7.13E+05 | 5.61E+05 | 5.59E+04 | 9.54E+04 |
| **758** | F1RP34 | NAD(P)H-hydrate epimerase | APOA1BP | 1.06E+07 | 9.49E+06 | 0.00E+00 | 1.08E+06 | 7.05E+05 | 6.32E+05 | 0.00E+00 | 7.23E+04 |
| **759** | F1SME6 | Uncharacterized protein | | 2.11E+07 | 3.56E+06 | 7.97E+06 | 9.56E+06 | 7.03E+05 | 1.19E+05 | 2.66E+05 | 3.19E+05 |
| **760** | F1RL81 | Uncharacterized protein | HSD17B14 | 1.12E+07 | 4.76E+06 | 5.49E+06 | 9.80E+05 | 7.02E+05 | 2.97E+05 | 3.43E+05 | 6.13E+04 |
| **761** | H9KV75 | Alpha-actinin-1 | ACTN1 | 3.78E+07 | 2.64E+07 | 2.24E+06 | 9.17E+06 | 7.00E+05 | 4.89E+05 | 4.14E+04 | 1.70E+05 |
| **762** | F2Z2F3 | Uncharacterized protein | MTAP-ANRIL fusion | 7.69E+06 | 3.14E+06 | 1.95E+06 | 2.60E+06 | 6.99E+05 | 2.85E+05 | 1.78E+05 | 2.36E+05 |
| **763** | P50897 | Palmitoyl-protein thioesterase 1 | PPT1 | 8.97E+06 | 4.46E+06 | 1.43E+06 | 3.09E+06 | 6.90E+05 | 3.43E+05 | 1.10E+05 | 2.37E+05 |
| **764** | F2Z584 | Histone H2B | HIST1H2BD | 7.52E+06 | 3.51E+06 | 2.11E+06 | 1.90E+06 | 6.84E+05 | 3.19E+05 | 1.92E+05 | 1.73E+05 |
| **765** | H0YB39 | Heterogeneous nuclear ribonucleoprotein H (Fragment) | HNRNPH1 | 1.23E+07 | 5.26E+06 | 4.90E+06 | 2.13E+06 | 6.83E+05 | 2.92E+05 | 2.72E+05 | 1.19E+05 |
| **766** | F1SV23 | Uncharacterized protein | NDUFS5 | 4.77E+06 | 2.59E+06 | 1.67E+06 | 5.06E+05 | 6.82E+05 | 3.70E+05 | 2.39E+05 | 7.23E+04 |
| **767** | C9J8R8 | Glycogenin-1 (Fragment) | GYG1 | 2.71E+06 | 4.92E+05 | 2.70E+05 | 1.95E+06 | 6.78E+05 | 1.23E+05 | 6.76E+04 | 4.87E+05 |
| **768** | F1RWJ6 | Uncharacterized protein | NPEPPS | 3.85E+07 | 2.04E+07 | 7.36E+06 | 1.07E+07 | 6.75E+05 | 3.58E+05 | 1.29E+05 | 1.88E+05 |
| **769** | P48819 | Vitronectin | VTN | 1.88E+07 | 4.73E+06 | 6.60E+06 | 7.44E+06 | 6.70E+05 | 1.69E+05 | 2.36E+05 | 2.66E+05 |
| **770** | Q28936 | Fibrinogen A-alpha-chain (Fragment) | | 1.67E+07 | 4.55E+06 | 5.54E+06 | 6.62E+06 | 6.68E+05 | 1.82E+05 | 2.22E+05 | 2.65E+05 |
| **771** | H3BQQ9 | SUMO-conjugating enzyme (Fragment) | UBE2I | 5.34E+06 | 3.71E+06 | 8.47E+05 | 7.80E+05 | 6.67E+05 | 4.64E+05 | 1.06E+05 | 9.75E+04 |
| **772** | F1S530 | 60S ribosomal protein L5 | RPL5 | 1.13E+07 | 5.11E+06 | 2.87E+06 | 3.33E+06 | 6.65E+05 | 3.01E+05 | 1.69E+05 | 1.96E+05 |
| **773** | F1SGH5 | Uncharacterized protein | PDHB | 1.40E+07 | 8.88E+06 | 3.51E+06 | 1.57E+06 | 6.65E+05 | 4.23E+05 | 1.67E+05 | 7.47E+04 |
| **774** | Q29583 | Glutathione transferase mu (Fragment) | | 5.95E+06 | 8.23E+05 | 1.75E+06 | 3.38E+06 | 6.61E+05 | 9.14E+04 | 1.94E+05 | 3.75E+05 |
| **775** | F1SMN6 | V-type proton ATPase subunit F | ATP6V1F | 5.28E+06 | 5.63E+05 | 2.05E+06 | 2.67E+06 | 6.61E+05 | 7.03E+04 | 2.57E+05 | 3.33E+05 |
| **776** | L8AXM5 | IgG heavy chain | IGHG | 1.32E+07 | 3.39E+06 | 3.25E+06 | 6.51E+06 | 6.58E+05 | 1.70E+05 | 1.62E+05 | 3.26E+05 |
| **777** | F1RPW8 | Uncharacterized protein | ASRGL1 | 9.19E+06 | 4.06E+06 | 2.46E+06 | 2.67E+06 | 6.56E+05 | 2.90E+05 | 1.75E+05 | 1.91E+05 |
| **778** | Q29561 | UMP-CMP kinase | CMPK1 | 7.87E+06 | 4.04E+06 | 1.74E+06 | 2.09E+06 | 6.56E+05 | 3.37E+05 | 1.45E+05 | 1.74E+05 |
| **779** | F2Z522 | Uncharacterized protein | LOC100623805 | 5.90E+06 | 2.23E+06 | 1.73E+06 | 1.94E+06 | 6.55E+05 | 2.47E+05 | 1.93E+05 | 2.15E+05 |
| **780** | B3KX15 | cDNA FLJ44468 fis | SRSF2 | 6.55E+06 | 5.28E+06 | 0.00E+00 | 1.27E+06 | 6.55E+05 | 5.28E+05 | 0.00E+00 | 1.27E+05 |
| **781** | F1RNZ1 | Cytochrome b-c1 complex subunit Rieske, mitochondrial | UQCRFS1 | 1.05E+07 | 5.17E+06 | 3.20E+06 | 2.10E+06 | 6.54E+05 | 3.23E+05 | 2.00E+05 | 1.31E+05 |
| **782** | K7GNZ6 | Uncharacterized protein (Fragment) | SSR4 | 5.22E+06 | 2.54E+06 | 4.24E+05 | 2.25E+06 | 6.52E+05 | 3.18E+05 | 5.30E+04 | 2.81E+05 |
| **783** | F1RVM1 | Uncharacterized protein | APOBEC2 | 8.48E+06 | 4.66E+06 | 1.14E+06 | 2.68E+06 | 6.52E+05 | 3.58E+05 | 8.77E+04 | 2.06E+05 |
| **784** | P10775 | Ribonuclease inhibitor | RNH1 | 1.69E+07 | 1.07E+07 | 5.06E+06 | 1.18E+06 | 6.51E+05 | 4.11E+05 | 1.95E+05 | 4.53E+04 |
| **785** | F1S5D0 | Uncharacterized protein | BST1 | 1.04E+07 | 4.19E+06 | 1.91E+06 | 4.31E+06 | 6.51E+05 | 2.62E+05 | 1.20E+05 | 2.70E+05 |
| **786** | A0A024R7G2 | RAB3D, member RAS oncogene family, isoform CRA | RAB3D | 1.04E+07 | 4.15E+06 | 2.89E+06 | 3.32E+06 | 6.48E+05 | 2.59E+05 | 1.81E+05 | 2.07E+05 |
| **787** | A8KA83 | cDNA FLJ78586 | | 1.03E+07 | 4.36E+06 | 2.79E+06 | 3.14E+06 | 6.43E+05 | 2.72E+05 | 1.75E+05 | 1.96E+05 |
| **788** | K9J4R8 | Protein S100-A13 | S100A13 | 5.13E+06 | 4.51E+05 | 2.14E+06 | 2.54E+06 | 6.41E+05 | 5.64E+04 | 2.68E+05 | 3.17E+05 |
| **789** | V9HW51 | Epididymis secretory protein Li 114 | HEL-S-114 | 9.57E+06 | 6.73E+06 | 1.10E+06 | 1.73E+06 | 6.38E+05 | 4.49E+05 | 7.36E+04 | 1.16E+05 |
| **790** | F1STC3 | Uncharacterized protein | | 3.19E+06 | 2.22E+06 | 0.00E+00 | 9.67E+05 | 6.38E+05 | 4.44E+05 | 0.00E+00 | 1.93E+05 |
| **791** | P36968 | Phospholipid hydroperoxide glutathione peroxidase, mitochondrial | GPX4 | 9.52E+06 | 3.10E+06 | 2.16E+06 | 4.27E+06 | 6.35E+05 | 2.06E+05 | 1.44E+05 | 2.84E+05 |
| **792** | Q8SPA2 | MHC class II antigen | SLA-DRB | 1.07E+07 | 4.72E+06 | 3.03E+06 | 3.00E+06 | 6.32E+05 | 2.78E+05 | 1.78E+05 | 1.76E+05 |
| **793** | H7C547 | Sodium/potassium-transporting ATPase subunit beta-3 (Fragment) | ATP1B3 | 1.89E+06 | 1.40E+06 | 4.90E+05 | 0.00E+00 | 6.29E+05 | 4.66E+05 | 1.63E+05 | 0.00E+00 |
| **794** | F1SSS1 | Uncharacterized protein | LOC100153701 | 1.26E+07 | 6.21E+06 | 2.36E+06 | 4.01E+06 | 6.29E+05 | 3.11E+05 | 1.18E+05 | 2.00E+05 |
| **795** | F1SEY8 | Uncharacterized protein | LOC100738775 | 2.77E+07 | 2.42E+06 | 0.00E+00 | 2.52E+07 | 6.29E+05 | 5.49E+04 | 0.00E+00 | 5.74E+05 |
| **796** | D3DTX7 | Collagen, type I, alpha 1, isoform CRA | COL1A1 | 3.33E+07 | 1.82E+07 | 8.25E+06 | 6.84E+06 | 6.28E+05 | 3.43E+05 | 1.56E+05 | 1.29E+05 |
| **797** | F1S8H8 | Purine nucleoside phosphorylase | PNP | 1.44E+07 | 3.71E+06 | 4.50E+06 | 6.23E+06 | 6.28E+05 | 1.62E+05 | 1.96E+05 | 2.71E+05 |
| **798** | F1SUP1 | Uncharacterized protein | AKR7A2 | 1.00E+07 | 6.26E+06 | 1.31E+06 | 2.44E+06 | 6.25E+05 | 3.91E+05 | 8.17E+04 | 1.52E+05 |
| **799** | Z4YP82 | SPARC | SPARC | 9.37E+06 | 2.06E+06 | 4.33E+06 | 2.98E+06 | 6.25E+05 | 1.37E+05 | 2.89E+05 | 1.99E+05 |
| **800** | I3LSI3 | Adenylate kinase 2, mitochondrial | AK2 | 1.06E+07 | 5.94E+06 | 2.33E+06 | 2.35E+06 | 6.25E+05 | 3.49E+05 | 1.37E+05 | 1.38E+05 |
| **801** | Q3HUX1 | Fatty acid translocase/CD36 | CD36 | 1.06E+07 | 2.41E+06 | 6.48E+06 | 1.72E+06 | 6.24E+05 | 1.42E+05 | 3.81E+05 | 1.01E+05 |
| **802** | F1SDC7 | Uncharacterized protein | ALDH3A2 | 1.18E+07 | 6.48E+06 | 2.94E+06 | 2.38E+06 | 6.21E+05 | 3.41E+05 | 1.55E+05 | 1.25E+05 |
| **803** | Q06AS8 | GBAK | GNAI3 | 1.05E+07 | 4.84E+06 | 2.75E+06 | 2.91E+06 | 6.18E+05 | 2.85E+05 | 1.62E+05 | 1.71E+05 |
| **804** | I3LH27 | Uncharacterized protein | CRTC3 | 1.17E+07 | 1.17E+07 | 0.00E+00 | 0.00E+00 | 6.14E+05 | 6.14E+05 | 0.00E+00 | 0.00E+00 |
| **805** | F1S766 | Uncharacterized protein | MAGOH | 8.60E+06 | 4.71E+06 | 7.83E+05 | 3.11E+06 | 6.14E+05 | 3.36E+05 | 5.59E+04 | 2.22E+05 |
| **806** | F2Z5C5 | Uncharacterized protein | LSM6 | 3.06E+06 | 1.62E+06 | 5.53E+05 | 8.94E+05 | 6.12E+05 | 3.23E+05 | 1.11E+05 | 1.79E+05 |
| **807** | A0A087WWQ5 | Arrestin-C | ARR3 | 1.10E+07 | 4.77E+06 | 2.01E+06 | 4.24E+06 | 6.12E+05 | 2.65E+05 | 1.12E+05 | 2.35E+05 |
| **808** | I3LS72 | Uncharacterized protein | COL6A1 | 1.83E+07 | 7.76E+06 | 4.39E+06 | 6.13E+06 | 6.10E+05 | 2.59E+05 | 1.46E+05 | 2.04E+05 |
| **809** | I3LJ03 | Uncharacterized protein | | 1.83E+06 | 1.06E+06 | 0.00E+00 | 7.70E+05 | 6.10E+05 | 3.53E+05 | 0.00E+00 | 2.57E+05 |
| **810** | F1SKQ7 | Uncharacterized protein | GOLGA1 | 3.41E+07 | 2.75E+07 | 0.00E+00 | 6.61E+06 | 6.09E+05 | 4.91E+05 | 0.00E+00 | 1.18E+05 |
| **811** | M9TGS8 | Mitochondrial delta3,delta2-dienoyl-CoA isomerase | Eci1 | 1.03E+07 | 4.07E+06 | 3.65E+06 | 2.60E+06 | 6.07E+05 | 2.39E+05 | 2.15E+05 | 1.53E+05 |
| **812** | F1S133 | Uncharacterized protein | CFI | 2.35E+07 | 1.38E+07 | 5.41E+06 | 4.35E+06 | 6.03E+05 | 3.53E+05 | 1.39E+05 | 1.12E+05 |
| **813** | Q0IIN1 | Keratin 77 | Krt77 Keratin, type II c | 2.34E+07 | 4.98E+06 | 8.37E+06 | 1.01E+07 | 6.00E+05 | 1.28E+05 | 2.15E+05 | 2.58E+05 |
| **814** | F1RKM0 | Uncharacterized protein | LMNB1 | 2.28E+07 | 7.29E+06 | 7.94E+06 | 7.53E+06 | 5.99E+05 | 1.92E+05 | 2.09E+05 | 1.98E+05 |
| **815** | F1SH25 | Uncharacterized protein | RAB21 | 8.97E+06 | 3.33E+06 | 1.43E+06 | 4.21E+06 | 5.98E+05 | 2.22E+05 | 9.55E+04 | 2.81E+05 |
| **816** | F1RG31 | Uncharacterized protein (Fragment) | | 1.20E+06 | 0.00E+00 | 3.17E+05 | 8.79E+05 | 5.98E+05 | 0.00E+00 | 1.58E+05 | 4.39E+05 |
| **817** | A2NYV3 | Light chain Fab (Fragment) | | 2.38E+06 | 2.38E+06 | 0.00E+00 | 0.00E+00 | 5.94E+05 | 5.94E+05 | 0.00E+00 | 0.00E+00 |
| **818** | F1S0V3 | Annexin | ANXA6 | 2.25E+07 | 7.23E+06 | 8.22E+06 | 7.06E+06 | 5.92E+05 | 1.90E+05 | 2.16E+05 | 1.86E+05 |
| **819** | I3LVJ7 | Uncharacterized protein | PSMA7 | 8.88E+06 | 5.33E+06 | 3.55E+06 | 0.00E+00 | 5.92E+05 | 3.56E+05 | 2.36E+05 | 0.00E+00 |
| **820** | I3LNZ2 | Uncharacterized protein | LOC100510904 | 4.14E+06 | 0.00E+00 | 1.59E+06 | 2.54E+06 | 5.91E+05 | 0.00E+00 | 2.28E+05 | 3.63E+05 |
| **821** | B6CVD6 | Thioredoxin domain-containing 4 | TXNDC4 | 1.35E+07 | 3.12E+06 | 4.22E+06 | 6.18E+06 | 5.88E+05 | 1.36E+05 | 1.84E+05 | 2.69E+05 |
| **822** | A0A087X0S5 | Collagen alpha-1(VI) chain | COL6A1 | 3.64E+07 | 1.62E+07 | 1.00E+07 | 1.03E+07 | 5.88E+05 | 2.61E+05 | 1.61E+05 | 1.65E+05 |
| **823** | A5A755 | Guanine nucleotide-binding protein (Fragment) | GNAQ | 1.33E+07 | 2.93E+06 | 3.46E+06 | 6.95E+06 | 5.80E+05 | 1.28E+05 | 1.50E+05 | 3.02E+05 |
| **824** | Q562Z4 | Actin-like protein (Fragment) | ACT | 3.47E+06 | 1.45E+06 | 6.80E+05 | 1.34E+06 | 5.78E+05 | 2.42E+05 | 1.13E+05 | 2.23E+05 |
| **825** | Q9UEN1 | U2 snRNP-specific A protein | | 5.75E+05 | 0.00E+00 | 5.75E+05 | 0.00E+00 | 5.75E+05 | 0.00E+00 | 5.75E+05 | 0.00E+00 |
| **826** | Q6QAP7 | 40S ribosomal protein S17 | RPS17 | 3.44E+06 | 2.31E+06 | 5.26E+05 | 6.10E+05 | 5.74E+05 | 3.85E+05 | 8.76E+04 | 1.02E+05 |
| **827** | F1S604 | Uncharacterized protein | GSTM2 | 4.55E+06 | 0.00E+00 | 4.55E+06 | 0.00E+00 | 5.69E+05 | 0.00E+00 | 5.69E+05 | 0.00E+00 |
| **828** | B2R6U8 | cDNA, FLJ93125 | | 1.02E+07 | 5.16E+06 | 9.04E+05 | 4.15E+06 | 5.67E+05 | 2.87E+05 | 5.02E+04 | 2.30E+05 |
| **829** | Q29307 | ATPase inhibitor, mitochondrial | ATPIF1 | 4.53E+06 | 1.38E+05 | 1.92E+06 | 2.48E+06 | 5.67E+05 | 1.72E+04 | 2.40E+05 | 3.09E+05 |
| **830** | F2Z518 | Uncharacterized protein | LAMTOR2 | 3.39E+06 | 1.22E+06 | 1.14E+06 | 1.03E+06 | 5.65E+05 | 2.03E+05 | 1.90E+05 | 1.72E+05 |
| **831** | F1S3H1 | Uncharacterized protein | ALDH6A1 | 1.86E+07 | 9.17E+06 | 4.42E+06 | 5.00E+06 | 5.63E+05 | 2.78E+05 | 1.34E+05 | 1.51E+05 |
| **832** | F1S2E2 | Annexin | ANXA11 | 1.24E+07 | 7.01E+06 | 1.13E+06 | 4.23E+06 | 5.63E+05 | 3.19E+05 | 5.15E+04 | 1.92E+05 |
| **833** | P81405 | Saposin-B-Val | PSAP | 2.81E+06 | 0.00E+00 | 6.81E+05 | 2.13E+06 | 5.61E+05 | 0.00E+00 | 1.36E+05 | 4.25E+05 |
| **834** | F1RGI2 | Uncharacterized protein | LOC100514845 | 9.54E+06 | 3.01E+06 | 3.88E+06 | 2.65E+06 | 5.61E+05 | 1.77E+05 | 2.28E+05 | 1.56E+05 |
| **835** | I3LCI0 | Uncharacterized protein | LOC100525869 | 9.51E+06 | 6.09E+06 | 1.96E+06 | 1.46E+06 | 5.59E+05 | 3.58E+05 | 1.15E+05 | 8.61E+04 |
| **836** | F2Z5S4 | Uncharacterized protein | LSM3 | 2.23E+06 | 0.00E+00 | 2.23E+06 | 0.00E+00 | 5.59E+05 | 0.00E+00 | 5.59E+05 | 0.00E+00 |
| **837** | F1RIP3 | Ferritin | FTL | 6.70E+06 | 2.46E+06 | 2.00E+06 | 2.24E+06 | 5.59E+05 | 2.05E+05 | 1.67E+05 | 1.87E+05 |
| **838** | Q19AZ8 | Prothrombin | F2 | 1.83E+07 | 7.19E+06 | 6.50E+06 | 4.64E+06 | 5.55E+05 | 2.18E+05 | 1.97E+05 | 1.41E+05 |
| **839** | A9XXE1 | Gamma-synuclein | SNCG | 2.77E+06 | 1.15E+06 | 1.41E+06 | 1.98E+05 | 5.53E+05 | 2.31E+05 | 2.83E+05 | 3.97E+04 |
| **840** | Q53XB4 | Full-length cDNA clone CS0DF032YM23 of Fetal brain of Homo sapiens (human) | RAB1 | 5.44E+06 | 2.13E+06 | 1.08E+06 | 2.23E+06 | 5.44E+05 | 2.13E+05 | 1.08E+05 | 2.23E+05 |
| **841** | K7GMF7 | Uncharacterized protein (Fragment) | ILF2 | 7.60E+06 | 2.57E+06 | 1.79E+06 | 3.24E+06 | 5.43E+05 | 1.83E+05 | 1.28E+05 | 2.31E+05 |
| **842** | F1RGM3 | Uncharacterized protein | TXNDC17 | 4.33E+06 | 1.82E+06 | 1.11E+06 | 1.40E+06 | 5.41E+05 | 2.27E+05 | 1.39E+05 | 1.75E+05 |
| **843** | M3V7X9 | Lectin, galactoside-binding, soluble, 3 binding protein | LGALS3BP | 1.30E+07 | 1.50E+06 | 3.42E+06 | 8.05E+06 | 5.40E+05 | 6.26E+04 | 1.42E+05 | 3.35E+05 |
| **844** | P07802 | cAMP-dependent protein kinase type I-alpha regulatory subunit | PRKAR1A | 1.13E+07 | 6.20E+06 | 2.68E+06 | 2.42E+06 | 5.38E+05 | 2.95E+05 | 1.27E+05 | 1.15E+05 |
| **845** | I3LH70 | Uncharacterized protein | COL18A1 | 8.05E+06 | 5.10E+06 | 1.55E+06 | 1.41E+06 | 5.37E+05 | 3.40E+05 | 1.03E+05 | 9.37E+04 |
| **846** | F1ST98 | Uncharacterized protein | CBX3 | 4.82E+06 | 9.33E+05 | 5.60E+05 | 3.33E+06 | 5.36E+05 | 1.04E+05 | 6.22E+04 | 3.70E+05 |
| **847** | F1RR44 | Serine/threonine-protein phosphatase 2A activator | PPP2R4 | 1.17E+07 | 5.88E+06 | 4.02E+06 | 1.85E+06 | 5.34E+05 | 2.67E+05 | 1.83E+05 | 8.39E+04 |
| **848** | F2Z5T5 | Tubulin alpha-1A chain | TUBA1A | 1.23E+07 | 8.17E+06 | 1.77E+06 | 2.34E+06 | 5.34E+05 | 3.55E+05 | 7.69E+04 | 1.02E+05 |
| **849** | Q3TTY5 | Keratin, type II cytoskeletal 2 epidermal | Krt2 Keratin, type II cy | 2.29E+07 | 2.76E+05 | 0.00E+00 | 2.26E+07 | 5.33E+05 | 6.41E+03 | 0.00E+00 | 5.27E+05 |
| **850** | B7Z2R2 | Cytochrome b-c1 complex subunit 7 | UQCRB | 6.93E+06 | 3.88E+06 | 1.28E+06 | 1.76E+06 | 5.33E+05 | 2.99E+05 | 9.83E+04 | 1.36E+05 |
| **851** | Q5ISC6 | Heme-binding protein 1 | HEBP1 | 7.45E+06 | 2.51E+06 | 1.46E+06 | 3.48E+06 | 5.32E+05 | 1.79E+05 | 1.04E+05 | 2.49E+05 |
| **852** | K9IVR0 | Histone-binding protein RBBP4 | RBBP4 | 9.03E+06 | 2.65E+06 | 5.17E+06 | 1.21E+06 | 5.31E+05 | 1.56E+05 | 3.04E+05 | 7.10E+04 |
| **853** | F1S1L1 | Uncharacterized protein | TIMM8A | 3.19E+06 | 0.00E+00 | 1.96E+06 | 1.23E+06 | 5.31E+05 | 0.00E+00 | 3.27E+05 | 2.04E+05 |
| **854** | J3QL48 | Uncharacterized protein (Fragment) | PSMB10 | 2.12E+06 | 5.76E+05 | 0.00E+00 | 1.55E+06 | 5.31E+05 | 1.44E+05 | 0.00E+00 | 3.87E+05 |
| **855** | O60493 | Sorting nexin-3 | SNX3 | 5.83E+06 | 3.65E+06 | 1.02E+06 | 1.16E+06 | 5.30E+05 | 3.32E+05 | 9.31E+04 | 1.06E+05 |
| **856** | F1SRI8 | Uncharacterized protein | MYBPC1 | 3.76E+07 | 2.02E+07 | 7.39E+06 | 9.96E+06 | 5.30E+05 | 2.85E+05 | 1.04E+05 | 1.40E+05 |
| **857** | A0SEH3 | Complement component C8G | C8G | 6.82E+06 | 2.88E+06 | 2.54E+06 | 1.40E+06 | 5.25E+05 | 2.21E+05 | 1.96E+05 | 1.08E+05 |
| **858** | F1SHL3 | Uncharacterized protein | SEC23A | 2.04E+07 | 7.21E+06 | 3.59E+06 | 9.64E+06 | 5.24E+05 | 1.85E+05 | 9.21E+04 | 2.47E+05 |
| **859** | F1S2I8 | Uncharacterized protein | TPPP3 | 7.30E+06 | 3.71E+06 | 1.89E+06 | 1.69E+06 | 5.21E+05 | 2.65E+05 | 1.35E+05 | 1.21E+05 |
| **860** | F1SRL9 | Uncharacterized protein | BASP1 | 7.81E+06 | 1.49E+06 | 3.56E+06 | 2.77E+06 | 5.21E+05 | 9.91E+04 | 2.37E+05 | 1.84E+05 |
| **861** | C9JRU6 | DCN1-like protein (Fragment) | DCUN1D1 | 3.12E+06 | 1.31E+06 | 9.40E+05 | 8.70E+05 | 5.20E+05 | 2.18E+05 | 1.57E+05 | 1.45E+05 |
| **862** | F1SVB0 | Uncharacterized protein | CAPG | 8.82E+06 | 3.15E+06 | 3.69E+06 | 1.98E+06 | 5.19E+05 | 1.86E+05 | 2.17E+05 | 1.16E+05 |
| **863** | P79274 | Long-chain specific acyl-CoA dehydrogenase, mitochondrial | ACADL | 1.30E+07 | 8.24E+06 | 3.45E+06 | 1.26E+06 | 5.18E+05 | 3.30E+05 | 1.38E+05 | 5.04E+04 |
| **864** | Q6S8J3 | POTE ankyrin domain family member E | POTEE | 3.30E+07 | 1.58E+07 | 4.42E+06 | 1.27E+07 | 5.15E+05 | 2.48E+05 | 6.91E+04 | 1.98E+05 |
| **865** | F1S2G3 | Uncharacterized protein | TBCA | 3.56E+06 | 1.53E+06 | 1.05E+06 | 9.76E+05 | 5.08E+05 | 2.19E+05 | 1.50E+05 | 1.39E+05 |
| **866** | F1SNC2 | Guanine nucleotide-binding protein subunit gamma | GNG10 | 2.52E+06 | 9.35E+05 | 5.93E+05 | 9.95E+05 | 5.05E+05 | 1.87E+05 | 1.19E+05 | 1.99E+05 |
| **867** | Q05DN2 | IFIT2 protein (Fragment) | IFIT2 | 1.50E+07 | 9.77E+06 | 5.27E+06 | 0.00E+00 | 5.01E+05 | 3.26E+05 | 1.76E+05 | 0.00E+00 |
| **868** | Q29549 | Clusterin | CLU | 1.10E+07 | 6.95E+06 | 8.41E+05 | 3.23E+06 | 5.01E+05 | 3.16E+05 | 3.82E+04 | 1.47E+05 |
| **869** | F1RYA4 | Uncharacterized protein | NAP1L4 | 9.49E+06 | 3.38E+06 | 2.99E+06 | 3.11E+06 | 4.99E+05 | 1.78E+05 | 1.58E+05 | 1.64E+05 |
| **870** | Q2HYU1 | Mitochondrial creatine kinase 2 | CKMT2 | 1.40E+07 | 4.11E+06 | 4.12E+06 | 5.74E+06 | 4.99E+05 | 1.47E+05 | 1.47E+05 | 2.05E+05 |
| **871** | Q6QAS9 | 60S ribosomal protein L7 (Fragment) | RPL7 | 4.47E+06 | 2.35E+06 | 2.12E+06 | 0.00E+00 | 4.96E+05 | 2.61E+05 | 2.35E+05 | 0.00E+00 |
| **872** | K7GNN3 | Uncharacterized protein | MAP3K8 | 5.96E+06 | 1.64E+06 | 3.04E+06 | 1.29E+06 | 4.96E+05 | 1.36E+05 | 2.53E+05 | 1.07E+05 |
| **873** | Q06AU6 | Ras-related protein Rab-5A | RAB5A | 6.83E+06 | 4.13E+06 | 1.03E+06 | 1.66E+06 | 4.88E+05 | 2.95E+05 | 7.37E+04 | 1.19E+05 |
| **874** | F1SAS3 | Uncharacterized protein | APMAP | 1.16E+07 | 5.67E+06 | 2.86E+06 | 3.12E+06 | 4.85E+05 | 2.36E+05 | 1.19E+05 | 1.30E+05 |
| **875** | Q0MVN8 | Quinone oxidoreductase | CRYZ | 9.67E+06 | 7.07E+06 | 1.33E+06 | 1.27E+06 | 4.83E+05 | 3.53E+05 | 6.65E+04 | 6.36E+04 |
| **876** | F1SMX6 | Uncharacterized protein | SP100 | 9.12E+06 | 0.00E+00 | 0.00E+00 | 9.12E+06 | 4.80E+05 | 0.00E+00 | 0.00E+00 | 4.80E+05 |
| **877** | V9HWI1 | Epididymis secretory protein Li 10 | HEL-S-10 | 5.69E+06 | 3.33E+06 | 6.46E+05 | 1.72E+06 | 4.74E+05 | 2.77E+05 | 5.38E+04 | 1.43E+05 |
| **878** | F1RP64 | Uncharacterized protein | SLITRK1 | 1.61E+07 | 1.61E+07 | 0.00E+00 | 0.00E+00 | 4.74E+05 | 4.74E+05 | 0.00E+00 | 0.00E+00 |
| **879** | F1RHJ9 | Uncharacterized protein | GLOD4 | 1.66E+07 | 1.02E+07 | 4.30E+06 | 2.08E+06 | 4.73E+05 | 2.91E+05 | 1.23E+05 | 5.94E+04 |
| **880** | F1SMN5 | Uncharacterized protein | FLNC | 7.75E+07 | 2.10E+07 | 2.75E+07 | 2.90E+07 | 4.67E+05 | 1.27E+05 | 1.66E+05 | 1.74E+05 |
| **881** | F1SFD6 | Uncharacterized protein | GMFB | 4.63E+06 | 3.39E+06 | 3.83E+05 | 8.61E+05 | 4.63E+05 | 3.39E+05 | 3.83E+04 | 8.61E+04 |
| **882** | H9XFA0 | Myosin heavy chain 15 protein (Fragment) | MYH15 | 1.39E+06 | 1.39E+06 | 0.00E+00 | 0.00E+00 | 4.63E+05 | 4.63E+05 | 0.00E+00 | 0.00E+00 |
| **883** | J9JIL3 | Sodium/potassium-transporting ATPase subunit beta | ATP1B1 | 6.01E+06 | 6.01E+06 | 0.00E+00 | 0.00E+00 | 4.63E+05 | 4.63E+05 | 0.00E+00 | 0.00E+00 |
| **884** | F1SK66 | Uncharacterized protein | CADM2 | 6.92E+06 | 2.84E+06 | 2.14E+06 | 1.95E+06 | 4.62E+05 | 1.89E+05 | 1.43E+05 | 1.30E+05 |

| | | | | | | | | | | | |
|---|---|---|---|---|---|---|---|---|---|---|---|
| | | **Supplementary Table S1: (continued)** | | | | | | | | | |
| **885** | H3BQW8 | Hydroxyacylglutathione hydrolase, mitochondrial (Fragment) | HAGH | 5.53E+06 | 2.45E+06 | 1.48E+06 | 1.60E+06 | 4.61E+05 | 2.04E+05 | 1.23E+05 | 1.33E+05 |
| **886** | I3LDC3 | Uncharacterized protein | NDUFB10 | 5.98E+06 | 2.76E+06 | 1.25E+06 | 1.97E+06 | 4.60E+05 | 2.12E+05 | 9.62E+04 | 1.52E+05 |
| **887** | I3LUP5 | Synaptosomal-associated protein | LOC100627770 | 4.58E+06 | 3.31E+06 | 3.70E+05 | 9.02E+05 | 4.58E+05 | 3.31E+05 | 3.70E+04 | 9.02E+04 |
| **888** | F1RKM1 | Uncharacterized protein | ALDH7A1 | 1.01E+07 | 6.21E+06 | 1.60E+06 | 2.26E+06 | 4.58E+05 | 2.82E+05 | 7.27E+04 | 1.03E+05 |
| **889** | F2Z576 | Histone H3 | HIST1H3E | 2.74E+06 | 2.26E+06 | 4.80E+05 | 0.00E+00 | 4.57E+05 | 3.77E+05 | 8.00E+04 | 0.00E+00 |
| **890** | K7GST8 | Signal transducer and activator of transcription 1 (Fragment) | STAT1 | 7.31E+06 | 3.17E+05 | 2.55E+05 | 6.74E+06 | 4.57E+05 | 1.98E+04 | 1.59E+04 | 4.21E+05 |
| **891** | A0A024R280 | Phosphoserine aminotransferase 1, isoform CRA | PSAT1 | 1.09E+07 | 5.36E+06 | 2.71E+06 | 2.79E+06 | 4.53E+05 | 2.23E+05 | 1.13E+05 | 1.16E+05 |
| **892** | B1ALA9 | Ribose-phosphate pyrophosphokinase 1 | PRPS1 | 7.22E+06 | 4.43E+06 | 2.01E+06 | 7.76E+05 | 4.51E+05 | 2.77E+05 | 1.26E+05 | 4.85E+04 |
| **893** | F1SEQ7 | Uncharacterized protein | FAM213A | 6.30E+06 | 3.52E+06 | 1.10E+06 | 1.68E+06 | 4.50E+05 | 2.51E+05 | 7.88E+04 | 1.20E+05 |
| **894** | K7GQA9 | Uncharacterized protein (Fragment) | GSTZ1 | 6.30E+06 | 1.60E+06 | 1.65E+06 | 3.05E+06 | 4.50E+05 | 1.14E+05 | 1.18E+05 | 2.18E+05 |
| **895** | I3L7B0 | Uncharacterized protein | RPS7 | 3.60E+06 | 1.68E+06 | 1.26E+06 | 6.51E+05 | 4.50E+05 | 2.10E+05 | 1.58E+05 | 8.14E+04 |
| **896** | I3LUZ8 | Uncharacterized protein | LOC100516656 | 4.04E+06 | 2.51E+06 | 0.00E+00 | 1.53E+06 | 4.49E+05 | 2.78E+05 | 0.00E+00 | 1.70E+05 |
| **897** | A0PA01 | Serine protease inhibitor 9 | PI-9 | 9.87E+06 | 4.58E+06 | 1.79E+06 | 3.50E+06 | 4.49E+05 | 2.08E+05 | 8.12E+04 | 1.59E+05 |
| **898** | F1SQR8 | Leukotriene A(4) hydrolase | LTA4H | 1.75E+07 | 6.25E+06 | 5.82E+06 | 5.42E+06 | 4.48E+05 | 1.60E+05 | 1.49E+05 | 1.39E+05 |
| **899** | F1SB63 | Uncharacterized protein | TCP1 | 1.57E+07 | 4.43E+06 | 6.09E+06 | 5.16E+06 | 4.48E+05 | 1.27E+05 | 1.74E+05 | 1.47E+05 |
| **900** | I3LEV7 | Uncharacterized protein | SH3BGR | 4.01E+06 | 1.01E+06 | 1.03E+06 | 1.98E+06 | 4.46E+05 | 1.12E+05 | 1.14E+05 | 2.20E+05 |
| **901** | A8K6Y1 | cDNA FLJ75526 | PA2G4 | 1.02E+07 | 4.11E+06 | 2.26E+06 | 3.85E+06 | 4.44E+05 | 1.79E+05 | 9.81E+04 | 1.67E+05 |
| **902** | P19619 | Annexin A1 | ANXA1 | 9.68E+06 | 5.05E+06 | 8.56E+05 | 3.77E+06 | 4.40E+05 | 2.30E+05 | 3.89E+04 | 1.71E+05 |
| **903** | D0G0C8 | Chaperonin containing TCP1, subunit 2 (Beta) | CCT2 | 1.58E+07 | 4.54E+06 | 7.41E+06 | 3.86E+06 | 4.39E+05 | 1.26E+05 | 2.06E+05 | 1.07E+05 |
| **904** | A0A087WZS0 | Ly-6/neurotoxin-like protein 1 (Fragment) | LYNX1 | 1.31E+06 | 4.20E+05 | 4.74E+05 | 4.12E+05 | 4.35E+05 | 1.40E+05 | 1.58E+05 | 1.37E+05 |
| **905** | Q69DK8 | Complement C1s subcomponent | | 1.04E+07 | 2.56E+06 | 3.44E+06 | 4.43E+06 | 4.35E+05 | 1.07E+05 | 1.43E+05 | 1.85E+05 |
| **906** | A0A0S2Z377 | Annexin (Fragment) | ANXA6 | 2.17E+07 | 6.75E+06 | 6.30E+06 | 8.67E+06 | 4.35E+05 | 1.35E+05 | 1.26E+05 | 1.73E+05 |
| **907** | Q7Z517 | Transgelin | TAGLN3 | 7.38E+06 | 3.21E+06 | 1.54E+06 | 2.63E+06 | 4.34E+05 | 1.89E+05 | 9.08E+04 | 1.54E+05 |
| **908** | V9HW91 | Epididymis secretory sperm binding protein Li 8a | HEL-S-8a | 9.10E+06 | 3.17E+06 | 2.01E+06 | 3.92E+06 | 4.33E+05 | 1.51E+05 | 9.55E+04 | 1.87E+05 |
| **909** | F1SSK4 | Uncharacterized protein | TIMM9 | 2.59E+06 | 3.95E+05 | 9.97E+05 | 1.20E+06 | 4.32E+05 | 6.58E+04 | 1.66E+05 | 2.00E+05 |
| **910** | C9JTK6 | Obg-like ATPase 1 (Fragment) | OLA1 | 3.89E+06 | 1.36E+06 | 1.44E+06 | 1.09E+06 | 4.32E+05 | 1.51E+05 | 1.60E+05 | 1.21E+05 |
| **911** | F1S393 | Uncharacterized protein | CYB5B | 4.32E+06 | 1.69E+06 | 1.15E+06 | 1.48E+06 | 4.32E+05 | 1.69E+05 | 1.15E+05 | 1.48E+05 |
| **912** | Q9UPN1 | Serine/threonine-protein phosphatase (Fragment) | PPP1CC | 7.33E+06 | 2.84E+06 | 1.68E+06 | 2.80E+06 | 4.31E+05 | 1.67E+05 | 9.89E+04 | 1.65E+05 |
| **913** | Q1HBI0 | ATP synthase protein 8 | ATP8 | 1.72E+06 | 6.75E+05 | 4.22E+05 | 6.19E+05 | 4.29E+05 | 1.69E+05 | 1.06E+05 | 1.55E+05 |
| **914** | F1RIE0 | Uncharacterized protein | MESDC2 | 6.85E+06 | 2.04E+06 | 2.47E+06 | 2.35E+06 | 4.28E+05 | 1.27E+05 | 1.54E+05 | 1.47E+05 |
| **915** | P23687 | Prolyl endopeptidase | PREP | 2.14E+07 | 7.15E+06 | 6.13E+06 | 8.10E+06 | 4.28E+05 | 1.43E+05 | 1.23E+05 | 1.62E+05 |
| **916** | F1STB2 | Uncharacterized protein | HINT2 | 5.13E+06 | 1.57E+06 | 3.01E+06 | 5.45E+05 | 4.27E+05 | 1.31E+05 | 2.51E+05 | 4.54E+04 |
| **917** | V9HVX8 | Epididymis luminal protein 109 | HEL-109 | 1.41E+07 | 9.24E+06 | 4.86E+06 | 0.00E+00 | 4.27E+05 | 2.80E+05 | 1.47E+05 | 0.00E+00 |
| **918** | Q0QF01 | Succinate dehydrogenase [ubiquinone] flavoprotein subunit, mitochondrial | SDHA | 1.54E+07 | 4.75E+06 | 5.85E+06 | 4.75E+06 | 4.26E+05 | 1.32E+05 | 1.62E+05 | 1.32E+05 |
| **919** | F1S0M9 | Peptidyl-prolyl cis-trans isomerase | FKBP10 | 8.47E+06 | 1.99E+06 | 2.32E+06 | 4.16E+06 | 4.24E+05 | 9.95E+04 | 1.16E+05 | 2.08E+05 |
| **920** | Q53F48 | Heterogeneous nuclear ribonucleoprotein H3 isoform a variant (Fragment) | HNRPH3 | 8.41E+06 | 3.00E+06 | 1.41E+06 | 3.99E+06 | 4.20E+05 | 1.50E+05 | 7.06E+04 | 2.00E+05 |
| **921** | D6RDM7 | Ubiquitin-conjugating enzyme E2 K (Fragment) | UBE2K | 4.20E+06 | 2.61E+06 | 6.98E+05 | 8.87E+05 | 4.20E+05 | 2.61E+05 | 6.98E+04 | 8.87E+04 |
| **922** | F1RZM4 | Uncharacterized protein | LAMA4 | 3.62E+07 | 1.46E+07 | 1.01E+07 | 1.16E+07 | 4.17E+05 | 1.68E+05 | 1.16E+05 | 1.33E+05 |
| **923** | F1RLC5 | Ferritin | FTMT | 7.49E+06 | 7.49E+06 | 0.00E+00 | 0.00E+00 | 4.16E+05 | 4.16E+05 | 0.00E+00 | 0.00E+00 |
| **924** | F1S543 | Calponin | CNN3 | 7.87E+06 | 3.73E+06 | 8.05E+05 | 3.33E+06 | 4.14E+05 | 1.97E+05 | 4.24E+04 | 1.75E+05 |
| **925** | I3LEI8 | 1,2-dihydroxy-3-keto-5-methylthiopentene dioxygenase | ADI1 | 5.38E+06 | 1.36E+06 | 2.39E+06 | 1.63E+06 | 4.14E+05 | 1.04E+05 | 1.84E+05 | 1.25E+05 |
| **926** | F1S596 | Uncharacterized protein | PRKCSH | 8.65E+06 | 1.12E+06 | 3.13E+06 | 4.40E+06 | 4.12E+05 | 5.34E+04 | 1.49E+05 | 2.10E+05 |
| **927** | P01762 | Ig heavy chain V-III region TRO | | 2.45E+06 | 2.45E+06 | 0.00E+00 | 0.00E+00 | 4.09E+05 | 4.09E+05 | 0.00E+00 | 0.00E+00 |
| **928** | H7BXF3 | Transformer-2 protein homolog beta (Fragment) | TRA2B | 2.86E+06 | 2.07E+06 | 7.82E+05 | 0.00E+00 | 4.08E+05 | 2.96E+05 | 1.12E+05 | 0.00E+00 |
| **929** | J3QL22 | COP9 signalosome complex subunit 3 | COPS3 | 8.16E+05 | 0.00E+00 | 4.78E+05 | 3.38E+05 | 4.08E+05 | 0.00E+00 | 2.39E+05 | 1.69E+05 |
| **930** | R4GN98 | Protein S100 (Fragment) | S100A6 | 2.03E+06 | 2.03E+06 | 0.00E+00 | 0.00E+00 | 4.07E+05 | 4.07E+05 | 0.00E+00 | 0.00E+00 |
| **931** | Q9HAV0 | Guanine nucleotide-binding protein subunit beta-4 | GNB4 | 5.70E+06 | 4.25E+06 | 5.58E+05 | 8.83E+05 | 4.07E+05 | 3.04E+05 | 3.99E+04 | 6.30E+04 |
| **932** | F1SEX0 | Uncharacterized protein | DDAH1 | 4.88E+06 | 3.75E+06 | 0.00E+00 | 1.12E+06 | 4.06E+05 | 3.13E+05 | 0.00E+00 | 9.34E+04 |
| **933** | F1RUJ0 | Adenylyl cyclase-associated protein | CAP2 | 1.13E+07 | 3.36E+06 | 3.91E+06 | 3.99E+06 | 4.02E+05 | 1.20E+05 | 1.40E+05 | 1.42E+05 |
| **934** | H0YB22 | 40S ribosomal protein S14 (Fragment) | RPS14 | 2.38E+06 | 1.07E+06 | 5.27E+05 | 7.86E+05 | 3.97E+05 | 1.78E+05 | 8.78E+04 | 1.31E+05 |
| **935** | Q7YS28 | Pyruvate carboxylase | PC | 2.66E+07 | 1.49E+07 | 4.03E+06 | 7.67E+06 | 3.96E+05 | 2.22E+05 | 6.02E+04 | 1.14E+05 |
| **936** | I3LRH3 | Uncharacterized protein | LOC100625256 | 6.71E+06 | 1.40E+06 | 2.37E+06 | 2.95E+06 | 3.95E+05 | 8.21E+04 | 1.40E+05 | 1.73E+05 |
| **937** | I3LIL4 | Uncharacterized protein | MYO1C | 2.57E+07 | 1.57E+07 | 9.94E+06 | 0.00E+00 | 3.95E+05 | 2.42E+05 | 1.53E+05 | 0.00E+00 |
| **938** | H0YHM7 | Peptidyl-prolyl cis-trans isomerase FKBP11 (Fragment) | FKBP11 | 1.58E+06 | 1.16E+06 | 4.20E+05 | 0.00E+00 | 3.94E+05 | 2.89E+05 | 1.05E+05 | 0.00E+00 |
| **939** | D3GGC9 | Actinin-associated LIM protein 3 | PDLIM3 | 7.09E+06 | 3.94E+06 | 1.48E+06 | 1.67E+06 | 3.94E+05 | 2.19E+05 | 8.20E+04 | 9.28E+04 |
| **940** | D3DRL4 | HCG2011315, isoform CRA | hCG_2011315 | 2.75E+06 | 2.35E+06 | 0.00E+00 | 4.06E+05 | 3.94E+05 | 3.35E+05 | 0.00E+00 | 5.80E+04 |
| **941** | F1SBE5 | Uncharacterized protein | PTGIS | 1.13E+07 | 6.46E+06 | 4.14E+06 | 7.25E+05 | 3.91E+05 | 2.23E+05 | 1.43E+05 | 2.50E+04 |
| **942** | Q2YGT9 | 60S ribosomal protein L6 | RPL6 | 6.61E+06 | 3.97E+06 | 2.35E+06 | 2.89E+05 | 3.89E+05 | 2.33E+05 | 1.38E+05 | 1.70E+04 |
| **943** | A8K304 | cDNA FLJ75636, highly similar to Homo sapiens FYVE, RhoGEF and PH domain containing 2, mRNA | FGD2 | 1.82E+07 | 5.55E+06 | 4.61E+06 | 8.02E+06 | 3.87E+05 | 1.18E+05 | 9.80E+04 | 1.71E+05 |
| **944** | F1RIF3 | Uncharacterized protein | FAH | 9.26E+06 | 2.83E+06 | 2.85E+06 | 3.58E+06 | 3.86E+05 | 1.18E+05 | 1.19E+05 | 1.49E+05 |
| **945** | A0A087WW18 | Protein archease (Fragment) | ZBTB8OS | 7.64E+05 | 3.94E+05 | 3.71E+05 | 0.00E+00 | 3.82E+05 | 1.97E+05 | 1.85E+05 | 0.00E+00 |
| **946** | F1SLR1 | NADH dehydrogenase [ubiquinone] 1 alpha subcomplex subunit 8 | NDUFA8 | 4.58E+06 | 2.10E+06 | 7.40E+05 | 1.74E+06 | 3.81E+05 | 1.75E+05 | 6.17E+04 | 1.45E+05 |
| **947** | F1S0P3 | 2,3-cyclic-nucleotide 3-phosphodiesterase | CNP | 1.18E+07 | 4.63E+06 | 1.73E+06 | 5.41E+06 | 3.80E+05 | 1.49E+05 | 5.57E+04 | 1.75E+05 |
| **948** | I3LH91 | Uncharacterized protein | PALLD | 5.60E+06 | 9.18E+05 | 2.02E+06 | 2.66E+06 | 3.73E+05 | 6.12E+04 | 1.35E+05 | 1.78E+05 |
| **949** | I3LRI0 | Inosine-5-monophosphate dehydrogenase | IMPDH1 | 9.97E+06 | 4.91E+06 | 3.59E+06 | 1.46E+06 | 3.69E+05 | 1.82E+05 | 1.33E+05 | 5.43E+04 |
| **950** | F1SS26 | Uncharacterized protein | THBS1 | 2.48E+07 | 1.42E+07 | 4.92E+06 | 5.72E+06 | 3.65E+05 | 2.08E+05 | 7.24E+04 | 8.41E+04 |
| **951** | F1SGP8 | Uncharacterized protein | RCN1 | 5.83E+06 | 1.66E+06 | 1.56E+06 | 2.62E+06 | 3.64E+05 | 1.04E+05 | 9.73E+04 | 1.64E+05 |
| **952** | A0A0H3W5Q8 | Beta-crystallin B2 (Fragment) | CRYBB2 | 4.73E+06 | 2.09E+06 | 8.45E+05 | 1.80E+06 | 3.64E+05 | 1.61E+05 | 6.50E+04 | 1.38E+05 |
| **953** | F1SKI0 | Myosin-11 | MYH11 | 1.56E+07 | 1.03E+07 | 3.53E+06 | 1.80E+06 | 3.63E+05 | 2.39E+05 | 8.21E+04 | 4.19E+04 |
| **954** | P59083 | 14 kDa phosphohistidine phosphatase (Fragments) | PHPT1 | 1.45E+06 | 8.74E+05 | 0.00E+00 | 5.77E+05 | 3.63E+05 | 2.18E+05 | 0.00E+00 | 1.44E+05 |
| **955** | V9HWF0 | Epididymis secretory protein Li 28 | HEL-S-28 | 1.01E+07 | 6.75E+06 | 1.39E+06 | 2.01E+06 | 3.62E+05 | 2.41E+05 | 4.96E+04 | 7.17E+04 |
| **956** | Q7YQ92 | MHC class II antigen (Fragment) | SLA-DRA1 | 4.69E+06 | 2.04E+06 | 1.03E+06 | 1.62E+06 | 3.61E+05 | 1.57E+05 | 7.88E+04 | 1.25E+05 |
| **957** | Q6P5S3 | Uncharacterized protein | | 3.61E+06 | 3.61E+06 | 0.00E+00 | 0.00E+00 | 3.61E+05 | 3.61E+05 | 0.00E+00 | 0.00E+00 |
| **958** | F1SU03 | Uncharacterized protein | | 2.70E+07 | 1.15E+07 | 1.11E+07 | 4.43E+06 | 3.61E+05 | 1.54E+05 | 1.48E+05 | 5.90E+04 |
| **959** | Q0PM28 | Pigment epithelium-derived factor | SERPINF1 | 8.29E+06 | 5.80E+06 | 1.43E+06 | 1.05E+06 | 3.60E+05 | 2.52E+05 | 6.22E+04 | 4.57E+04 |
| **960** | Q9BUA6 | Myosin regulatory light chain 10 | MYL10 | 5.04E+06 | 0.00E+00 | 1.41E+06 | 3.63E+06 | 3.60E+05 | 0.00E+00 | 1.00E+05 | 2.59E+05 |
| **961** | I3LCA2 | Uncharacterized protein | CCT8 | 1.44E+07 | 3.44E+06 | 8.15E+06 | 2.79E+06 | 3.60E+05 | 8.61E+04 | 2.04E+05 | 6.97E+04 |
| **962** | P02535 | Isoform 1 of Keratin, type I cytoskeletal 10 | | 1.00E+07 | 0.00E+00 | 0.00E+00 | 1.00E+07 | 3.57E+05 | 0.00E+00 | 0.00E+00 | 3.57E+05 |
| **963** | F1SH96 | Inter-alpha-trypsin inhibitor heavy chain H1 | ITIH1 | 1.67E+07 | 5.73E+06 | 6.04E+06 | 4.93E+06 | 3.55E+05 | 1.22E+05 | 1.28E+05 | 1.05E+05 |
| **964** | I7HD36 | Sodium/potassium-transporting ATPase subunit alpha (Fragment) | atp1a1 | 1.84E+07 | 1.03E+07 | 4.94E+06 | 3.17E+06 | 3.54E+05 | 1.98E+05 | 9.51E+04 | 6.10E+04 |
| **965** | K7GSE5 | Uncharacterized protein | NDUFB9 | 3.54E+06 | 9.08E+05 | 0.00E+00 | 2.63E+06 | 3.54E+05 | 9.08E+04 | 0.00E+00 | 2.63E+05 |
| **966** | P00819 | Acylphosphatase-2 | ACYP2 | 2.81E+06 | 1.54E+06 | 5.70E+05 | 7.06E+05 | 3.51E+05 | 1.92E+05 | 7.12E+04 | 8.82E+04 |
| **967** | I3LKZ4 | Uncharacterized protein | HNRNPR | 1.37E+07 | 6.10E+06 | 3.36E+06 | 4.22E+06 | 3.51E+05 | 1.56E+05 | 8.60E+04 | 1.08E+05 |
| **968** | F1S4D5 | Uncharacterized protein | CCBL2 | 7.00E+06 | 1.86E+06 | 2.00E+06 | 3.14E+06 | 3.50E+05 | 9.28E+04 | 9.99E+04 | 1.57E+05 |
| **969** | Q684M6 | CDC37 cell division cycle 37 protein | CDC37 | 6.64E+06 | 2.73E+06 | 1.90E+06 | 2.00E+06 | 3.49E+05 | 1.44E+05 | 1.00E+05 | 1.05E+05 |
| **970** | Q6LBM3 | Fibroblast growth factor | fgf gene | 1.75E+06 | 0.00E+00 | 4.74E+05 | 1.27E+06 | 3.49E+05 | 0.00E+00 | 9.49E+04 | 2.54E+05 |
| **971** | C9J6B1 | Ras-related protein Ral-B (Fragment) | RALB | 3.13E+06 | 1.99E+06 | 1.14E+06 | 0.00E+00 | 3.48E+05 | 2.21E+05 | 1.27E+05 | 0.00E+00 |
| **972** | Q9BV28 | TUBB3 protein (Fragment) | TUBB3 | 6.95E+06 | 4.46E+05 | 3.30E+06 | 3.20E+06 | 3.47E+05 | 2.23E+04 | 1.65E+05 | 1.60E+05 |
| **973** | M0QZN2 | 40S ribosomal protein S5 | RPS5 | 2.08E+06 | 1.98E+06 | 0.00E+00 | 9.98E+04 | 3.46E+05 | 3.30E+05 | 0.00E+00 | 1.66E+04 |
| **974** | L8AXL3 | IgG heavy chain | IGHG | 6.22E+06 | 4.18E+06 | 1.40E+06 | 6.36E+05 | 3.45E+05 | 2.32E+05 | 7.78E+04 | 3.53E+04 |
| **975** | P06753 | Tropomyosin alpha-3 chain | TPM3 | 8.29E+06 | 7.00E+06 | 5.64E+05 | 7.18E+05 | 3.45E+05 | 2.92E+05 | 2.35E+04 | 2.99E+04 |
| **976** | F1RX84 | Uncharacterized protein | PROSC | 5.85E+06 | 2.42E+06 | 1.79E+06 | 1.64E+06 | 3.44E+05 | 1.42E+05 | 1.05E+05 | 9.62E+04 |
| **977** | K7GRT9 | Tetraspanin | CD151 | 4.43E+06 | 3.65E+06 | 0.00E+00 | 7.78E+05 | 3.41E+05 | 2.81E+05 | 0.00E+00 | 5.98E+04 |
| **978** | B7ZBT8 | Protocadherin-15 | PCDH15 | 1.87E+07 | 1.68E+07 | 0.00E+00 | 1.90E+06 | 3.41E+05 | 3.06E+05 | 0.00E+00 | 3.46E+04 |
| **979** | F1SII5 | Uncharacterized protein | INMT | 3.06E+06 | 2.35E+06 | 7.14E+05 | 0.00E+00 | 3.40E+05 | 2.61E+05 | 7.93E+04 | 0.00E+00 |
| **980** | C9J3L8 | Translocon-associated protein subunit alpha | SSR1 | 3.74E+06 | 1.98E+06 | 5.63E+05 | 1.20E+06 | 3.40E+05 | 1.80E+05 | 5.12E+04 | 1.09E+05 |
| **981** | I3L688 | Uncharacterized protein | LOC100516938 | 2.71E+06 | 9.02E+05 | 1.18E+06 | 6.26E+05 | 3.39E+05 | 1.13E+05 | 1.48E+05 | 7.83E+04 |
| **982** | A0A024R1X3 | Vacuolar protein sorting 25 (Yeast), isoform CRA | VPS25 | 3.73E+06 | 1.19E+06 | 1.01E+06 | 1.53E+06 | 3.39E+05 | 1.08E+05 | 9.15E+04 | 1.39E+05 |
| **983** | B4DL14 | ATP synthase subunit gamma | ATP5C1 | 5.07E+06 | 1.39E+06 | 1.91E+06 | 1.77E+06 | 3.38E+05 | 9.29E+04 | 1.27E+05 | 1.18E+05 |
| **984** | L7PBE6 | T-complex protein 1 subunit epsilon | CCT5 | 1.38E+07 | 4.78E+06 | 4.06E+06 | 4.99E+06 | 3.37E+05 | 1.17E+05 | 9.91E+04 | 1.22E+05 |
| **985** | A6NMH6 | Septin-8 | SEPT8 | 8.06E+06 | 0.00E+00 | 3.37E+06 | 4.68E+06 | 3.36E+05 | 0.00E+00 | 1.40E+05 | 1.95E+05 |
| **986** | Q9TV69 | Trans-1,2-dihydrobenzene-1,2-diol dehydrogenase | DHDH | 6.36E+06 | 2.84E+06 | 1.65E+06 | 1.87E+06 | 3.35E+05 | 1.50E+05 | 8.67E+04 | 9.82E+04 |
| **987** | F1ST73 | Uncharacterized protein | GRHPR | 4.33E+06 | 3.01E+06 | 7.13E+05 | 6.03E+05 | 3.33E+05 | 2.32E+05 | 5.49E+04 | 4.64E+04 |
| **988** | F1RNS2 | Uncharacterized protein | DIABLO | 4.97E+06 | 1.41E+06 | 9.80E+05 | 2.59E+06 | 3.32E+05 | 9.38E+04 | 6.54E+04 | 1.72E+05 |
| **989** | F1STY1 | NADH dehydrogenase [ubiquinone] 1 subunit C2 | NDUFC2 | 3.32E+06 | 8.76E+05 | 1.12E+06 | 1.32E+06 | 3.32E+05 | 8.76E+04 | 1.12E+05 | 1.32E+05 |
| **990** | K9J4R6 | ATP-dependent 6-phosphofructokinase | PFKP_tv1 | 1.19E+07 | 5.57E+06 | 1.54E+06 | 4.82E+06 | 3.31E+05 | 1.55E+05 | 4.29E+04 | 1.34E+05 |
| **991** | I3LDR9 | Uncharacterized protein | SDPR | 7.26E+06 | 1.65E+06 | 2.07E+06 | 3.53E+06 | 3.30E+05 | 7.51E+04 | 9.41E+04 | 1.61E+05 |
| **992** | B4DI57 | cDNA FLJ54111, highly similar to Serotransferrin | | 1.22E+07 | 5.26E+06 | 5.95E+06 | 9.70E+05 | 3.29E+05 | 1.42E+05 | 1.61E+05 | 2.62E+04 |
| **993** | I3LAI1 | Uncharacterized protein | | 6.90E+06 | 6.33E+06 | 2.72E+05 | 3.01E+05 | 3.29E+05 | 3.01E+05 | 1.29E+04 | 1.43E+04 |
| **994** | F1SDJ8 | Uncharacterized protein | GLRX3 | 6.88E+06 | 4.21E+06 | 1.06E+06 | 1.61E+06 | 3.28E+05 | 2.01E+05 | 5.05E+04 | 7.64E+04 |
| **995** | A0A0X9V9B3 | MS-F1 light chain variable region (Fragment) | | 1.95E+06 | 8.13E+05 | 0.00E+00 | 1.13E+06 | 3.25E+05 | 1.36E+05 | 0.00E+00 | 1.89E+05 |
| **996** | F1S2A8 | Uncharacterized protein | BCAP31 | 5.15E+06 | 1.28E+06 | 2.15E+06 | 1.73E+06 | 3.22E+05 | 7.99E+04 | 1.34E+05 | 1.08E+05 |
| **997** | J3K000 | PEPD protein | PEPD | 8.69E+06 | 5.19E+06 | 3.50E+06 | 0.00E+00 | 3.22E+05 | 1.92E+05 | 1.30E+05 | 0.00E+00 |
| **998** | F1RYT3 | Uncharacterized protein | SCARB2 | 8.68E+06 | 3.90E+06 | 2.59E+06 | 2.18E+06 | 3.22E+05 | 1.45E+05 | 9.60E+04 | 8.09E+04 |
| **999** | Q96QK1 | Vacuolar protein sorting-associated protein 35 | VPS35 | 1.51E+07 | 4.34E+06 | 2.86E+06 | 7.87E+06 | 3.21E+05 | 9.24E+04 | 6.09E+04 | 1.67E+05 |
| **1000** | F1SKQ9 | Actin-related protein 2/3 complex subunit 5 | ARPC5L | 3.85E+06 | 2.07E+06 | 5.75E+05 | 1.21E+06 | 3.21E+05 | 1.72E+05 | 4.79E+04 | 1.01E+05 |
| **1001** | F1SFF3 | Uncharacterized protein | NID2 | 2.27E+07 | 1.22E+07 | 5.15E+06 | 5.38E+06 | 3.20E+05 | 1.71E+05 | 7.26E+04 | 7.58E+04 |
| **1002** | F1RVD2 | Uncharacterized protein | ENOPH1 | 3.84E+06 | 1.54E+06 | 1.27E+06 | 1.03E+06 | 3.20E+05 | 1.28E+05 | 1.06E+05 | 8.60E+04 |
| **1003** | K9IWD4 | Hypoxia up-regulated protein 1 | HYOU1_tv1 | 1.88E+07 | 5.78E+06 | 6.44E+06 | 6.61E+06 | 3.19E+05 | 9.80E+04 | 1.09E+05 | 1.12E+05 |
| **1004** | Q9UK54 | Hemoglobin beta subunit variant (Fragment) | HBB | 2.87E+06 | 1.98E+06 | 0.00E+00 | 8.98E+05 | 3.19E+05 | 2.19E+05 | 0.00E+00 | 9.98E+04 |
| **1005** | I3LAA4 | Uncharacterized protein | LOC102165939 | 5.72E+06 | 4.81E+06 | 9.15E+05 | 0.00E+00 | 3.18E+05 | 2.67E+05 | 5.08E+04 | 0.00E+00 |
| **1006** | F1S3T0 | Glucosamine-6-phosphate isomerase | GNPDA2 | 5.37E+06 | 2.41E+06 | 1.94E+06 | 1.02E+06 | 3.16E+05 | 1.42E+05 | 1.14E+05 | 5.98E+04 |
| **1007** | H3BT58 | Coactosin-like protein | COTL1 | 1.58E+06 | 1.21E+06 | 0.00E+00 | 3.69E+05 | 3.16E+05 | 2.42E+05 | 0.00E+00 | 7.39E+04 |
| **1008** | Q3SYF1 | Sorting nexin 12 | SNX12 | 3.47E+06 | 1.84E+06 | 1.10E+06 | 5.39E+05 | 3.16E+05 | 1.67E+05 | 9.97E+04 | 4.90E+04 |
| **1009** | D6QST6 | 2,4-dienoyl-CoA reductase 1 | DECR1 | 7.23E+06 | 5.42E+06 | 4.83E+05 | 1.33E+06 | 3.14E+05 | 2.36E+05 | 2.10E+04 | 5.77E+04 |
| **1010** | I3L5L1 | Uncharacterized protein | RAP1B | 3.76E+06 | 1.83E+06 | 0.00E+00 | 1.93E+06 | 3.13E+05 | 1.52E+05 | 0.00E+00 | 1.61E+05 |
| **1011** | H0YHC3 | Nucleosome assembly protein 1-like 1 (Fragment) | NAP1L1 | 3.13E+06 | 1.47E+06 | 7.52E+05 | 9.10E+05 | 3.13E+05 | 1.47E+05 | 7.52E+04 | 9.10E+04 |
| **1012** | E5RHG8 | Transcription elongation factor B polypeptide 1 (Fragment) | TCEB1 | 2.50E+06 | 1.21E+06 | 1.12E+06 | 1.66E+05 | 3.13E+05 | 1.51E+05 | 1.40E+05 | 2.08E+04 |
| **1013** | J9JIK0 | Glutathione peroxidase | GPX6 | 3.12E+06 | 1.27E+06 | 6.27E+05 | 1.22E+06 | 3.12E+05 | 1.27E+05 | 6.27E+04 | 1.22E+05 |
| **1014** | A4Z6H0 | Adenylosuccinate synthetase isozyme 1 | ADSSL1 | 8.73E+06 | 2.89E+06 | 3.03E+06 | 2.82E+06 | 3.12E+05 | 1.03E+05 | 1.08E+05 | 1.01E+05 |
| **1015** | F1S4Z2 | Uncharacterized protein | CHMP4B | 4.97E+06 | 2.79E+06 | 0.00E+00 | 2.19E+06 | 3.11E+05 | 1.74E+05 | 0.00E+00 | 1.37E+05 |
| **1016** | C9JIE4 | Synemin | SYNM | 8.39E+06 | 5.66E+06 | 2.21E+06 | 5.12E+05 | 3.11E+05 | 2.10E+05 | 8.19E+04 | 1.90E+04 |
| **1017** | F1S6A9 | Uncharacterized protein | HEBP2 | 3.10E+06 | 1.31E+06 | 1.37E+06 | 4.10E+05 | 3.10E+05 | 1.31E+05 | 1.37E+05 | 4.10E+04 |
| **1018** | F1SDP2 | Uncharacterized protein | LHPP | 3.70E+06 | 2.35E+06 | 1.15E+06 | 1.91E+05 | 3.08E+05 | 1.96E+05 | 9.60E+04 | 1.59E+04 |
| **1019** | Q58FF8 | Putative heat shock protein HSP 90-beta 2 | HSP90AB2P | 7.02E+06 | 3.00E+06 | 1.55E+06 | 2.48E+06 | 3.05E+05 | 1.30E+05 | 6.72E+04 | 1.08E+05 |
| **1020** | F1RVN1 | Uncharacterized protein | NDUFV1 | 8.24E+06 | 5.05E+06 | 3.20E+06 | 0.00E+00 | 3.05E+05 | 1.87E+05 | 1.18E+05 | 0.00E+00 |
| **1021** | I3L5L2 | 40S ribosomal protein S26 | RPS26 | 1.22E+06 | 3.04E+05 | 3.06E+05 | 6.11E+05 | 3.05E+05 | 7.60E+04 | 7.64E+04 | 1.53E+05 |
| **1022** | A4USB5 | Cystatin B | CstB | 2.44E+06 | 3.36E+05 | 1.01E+06 | 1.09E+06 | 3.04E+05 | 4.20E+04 | 1.27E+05 | 1.36E+05 |
| **1023** | E3UV40 | Mitogen-activated protein kinase | ERK2 | 7.00E+06 | 1.66E+06 | 1.86E+06 | 3.48E+06 | 3.04E+05 | 7.20E+04 | 8.10E+04 | 1.51E+05 |
| **1024** | A0A087WTP3 | Far upstream element-binding protein 2 | KHSRP | 1.21E+07 | 8.51E+06 | 1.25E+06 | 2.38E+06 | 3.03E+05 | 2.13E+05 | 3.11E+04 | 5.95E+04 |
| **1025** | B1VD75 | Perilipin (Fragment) | PLIN | 7.88E+06 | 3.92E+06 | 3.96E+06 | 0.00E+00 | 3.03E+05 | 1.51E+05 | 1.52E+05 | 0.00E+00 |
| **1026** | Q5JUP3 | Allograft inflammatory factor 1-like | AIF1L | 2.42E+06 | 1.25E+06 | 0.00E+00 | 1.16E+06 | 3.02E+05 | 1.57E+05 | 0.00E+00 | 1.45E+05 |
| **1027** | F1SCH1 | Uncharacterized protein | NDUFB7 | 1.81E+06 | 1.14E+06 | 9.21E+04 | 5.75E+05 | 3.02E+05 | 1.91E+05 | 1.54E+04 | 9.59E+04 |
| **1028** | F1SNK3 | S-methyl-5-thioadenosine phosphorylase | MTAP | 5.42E+06 | 1.91E+06 | 2.14E+06 | 1.36E+06 | 3.01E+05 | 1.06E+05 | 1.19E+05 | 7.55E+04 |
| **1029** | K7XUQ9 | MHC class II antigen (Fragment) | SLA-DQA | 2.41E+06 | 1.05E+06 | 5.90E+05 | 7.65E+05 | 3.01E+05 | 1.31E+05 | 7.37E+04 | 9.56E+04 |
| **1030** | F1RR60 | Uncharacterized protein | PHYHD1 | 4.81E+06 | 2.50E+06 | 6.78E+05 | 1.63E+06 | 3.01E+05 | 1.56E+05 | 4.24E+04 | 1.02E+05 |
| **1031** | Q7IIB9 | ATP synthase protein 8 | ATPase 8 | 1.20E+06 | 3.49E+05 | 2.93E+05 | 5.53E+05 | 2.99E+05 | 8.72E+04 | 7.34E+04 | 1.38E+05 |
| **1032** | C3S7K5 | Protein S100 | S100A8 | 2.08E+06 | 8.08E+05 | 5.16E+05 | 7.56E+05 | 2.97E+05 | 1.15E+05 | 7.37E+04 | 1.08E+05 |

**Supplementary Table S1: (continued)**

| | | | | | | | | | | | |
|---|---|---|---|---|---|---|---|---|---|---|---|
| **1033** | I3L8S3 | Uncharacterized protein | CMBL | 3.25E+06 | 1.48E+06 | 1.16E+06 | 6.11E+05 | 2.95E+05 | 1.34E+05 | 1.05E+05 | 5.56E+04 |
| **1034** | B7Z3I9 | Delta-aminolevulinic acid dehydratase | | 5.31E+06 | 2.47E+06 | 0.00E+00 | 2.84E+06 | 2.95E+05 | 1.37E+05 | 0.00E+00 | 1.58E+05 |
| **1035** | F1SA70 | Uncharacterized protein | HSPA2 | 1.18E+07 | 3.34E+06 | 3.84E+06 | 4.62E+06 | 2.95E+05 | 8.35E+04 | 9.59E+04 | 1.16E+05 |
| **1036** | F5GYN4 | Ubiquitin thioesterase OTUB1 | OTUB1 | 4.42E+06 | 1.70E+06 | 1.30E+06 | 1.42E+06 | 2.95E+05 | 1.14E+05 | 8.68E+04 | 9.44E+04 |
| **1037** | I4AY87 | Macrophage migration inhibitory factor (Fragment) | MIF | 1.47E+06 | 0.00E+00 | 7.84E+05 | 6.85E+05 | 2.94E+05 | 0.00E+00 | 1.57E+05 | 1.37E+05 |
| **1038** | B7Z722 | Tropomyosin 1 (Alpha), isoform CRA | TPM1 | 6.14E+06 | 1.40E+06 | 2.40E+06 | 2.34E+06 | 2.92E+05 | 6.68E+04 | 1.14E+05 | 1.11E+05 |
| **1039** | A0A087WYV2 | IQ and AAA domain-containing protein 1-like | IQCA1L | 1.46E+07 | 0.00E+00 | 1.17E+07 | 2.88E+06 | 2.92E+05 | 0.00E+00 | 2.34E+05 | 5.76E+04 |
| **1040** | Q6DUB5 | Peripherial benzodiazepine receptor transcript variant | TSPO | 8.75E+05 | 8.75E+05 | 0.00E+00 | 0.00E+00 | 2.92E+05 | 2.92E+05 | 0.00E+00 | 0.00E+00 |
| **1041** | F1RUM1 | Uncharacterized protein | AFM | 1.08E+07 | 2.96E+06 | 3.37E+06 | 4.43E+06 | 2.91E+05 | 8.00E+04 | 9.09E+04 | 1.20E+05 |
| **1042** | F1SFR6 | Succinyl-CoA ligase subunit beta | SUCLG2 | 8.14E+06 | 8.14E+06 | 0.00E+00 | 0.00E+00 | 2.91E+05 | 2.91E+05 | 0.00E+00 | 0.00E+00 |
| **1043** | Q5T8U5 | Surfeit 4 | SURF4 | 2.61E+06 | 2.61E+06 | 0.00E+00 | 0.00E+00 | 2.90E+05 | 2.90E+05 | 0.00E+00 | 0.00E+00 |
| **1044** | Q06AU7 | Ras-related protein Rab-1B | RAB1B | 5.22E+06 | 1.73E+05 | 2.00E+06 | 3.05E+06 | 2.90E+05 | 9.59E+03 | 1.11E+05 | 1.70E+05 |
| **1045** | X5FUA3 | CDGSH iron sulfur domain 1 | C10orf70 | 1.74E+06 | 0.00E+00 | 9.44E+05 | 7.94E+05 | 2.90E+05 | 0.00E+00 | 1.57E+05 | 1.32E+05 |
| **1046** | J3QT28 | Mitotic checkpoint protein BUB3 (Fragment) | BUB3 | 4.33E+06 | 2.04E+06 | 8.11E+05 | 1.48E+06 | 2.89E+05 | 1.36E+05 | 5.41E+04 | 9.89E+04 |
| **1047** | M0R3H0 | 40S ribosomal protein S16 | RPS16 | 2.29E+06 | 7.81E+05 | 1.27E+05 | 1.38E+06 | 2.86E+05 | 9.76E+04 | 1.58E+04 | 1.73E+05 |
| **1048** | F1RKU4 | Uncharacterized protein | WDR61 | 4.57E+06 | 3.42E+06 | 0.00E+00 | 1.15E+06 | 2.86E+05 | 2.14E+05 | 0.00E+00 | 7.21E+04 |
| **1049** | F1SHD7 | Uncharacterized protein | NDUFS1 | 1.28E+07 | 4.42E+06 | 4.41E+06 | 4.00E+06 | 2.85E+05 | 9.83E+04 | 9.80E+04 | 8.88E+04 |
| **1050** | F1SK70 | Uncharacterized protein | PROS | 1.70E+06 | 2.78E+05 | 5.18E+05 | 9.08E+05 | 2.84E+05 | 4.63E+04 | 8.64E+04 | 1.51E+05 |
| **1051** | F1SPT5 | Uncharacterized protein | LAMB2 | 2.88E+07 | 8.46E+06 | 1.01E+07 | 1.02E+07 | 2.82E+05 | 8.29E+04 | 9.90E+04 | 1.00E+05 |
| **1052** | Q95287 | Sucrase-isomaltase, intestinal (Fragment) | | 1.40E+06 | 0.00E+00 | 0.00E+00 | 1.40E+06 | 2.81E+05 | 0.00E+00 | 0.00E+00 | 2.81E+05 |
| **1053** | I3LEZ3 | Uncharacterized protein | LSAMP | 2.51E+06 | 2.51E+06 | 0.00E+00 | 0.00E+00 | 2.79E+05 | 2.79E+05 | 0.00E+00 | 0.00E+00 |
| **1054** | Q7JFN3 | Calcium/calmodulin-dependent protein kinase II isoform gamma-C protein kinase II | CAMK2G | 7.80E+06 | 4.56E+06 | 1.36E+06 | 1.89E+06 | 2.79E+05 | 1.63E+05 | 4.86E+04 | 6.73E+04 |
| **1055** | D2XN65 | Glycerol-3-phosphate dehydrogenase [NAD(+)] | cGPD | 6.65E+06 | 5.15E+06 | 1.01E+06 | 4.98E+05 | 2.77E+05 | 2.14E+05 | 4.20E+04 | 2.08E+04 |
| **1056** | F6U1T9 | Calcineurin subunit B type 1 | PPP3R1 | 2.77E+06 | 0.00E+00 | 5.06E+05 | 2.26E+06 | 2.77E+05 | 0.00E+00 | 5.06E+04 | 2.26E+05 |
| **1057** | S4R435 | Protein RPS10-NUDT3 (Fragment) | RPS10-NUDT3 | 5.54E+06 | 2.03E+06 | 1.46E+06 | 2.05E+06 | 2.77E+05 | 1.01E+05 | 7.31E+04 | 1.02E+05 |
| **1058** | A0A0S2Z473 | Protein phosphatase 1 regulatory subunit 12B isoform 1 (Fragment) | PPP1R12B | 5.25E+06 | 5.25E+06 | 0.00E+00 | 0.00E+00 | 2.76E+05 | 2.76E+05 | 0.00E+00 | 0.00E+00 |
| **1059** | B2R8A2 | cDNA, FLJ93804, highly similar to Homo sapiens gp25L2 protein (HSGP25L2G), mRNA | TM | 4.10E+06 | 2.59E+06 | 0.00E+00 | 1.51E+06 | 2.74E+05 | 1.73E+05 | 0.00E+00 | 1.01E+05 |
| **1060** | P12309 | Glutaredoxin-1 | GLRX | 2.18E+06 | 0.00E+00 | 6.19E+05 | 1.56E+06 | 2.72E+05 | 0.00E+00 | 7.73E+04 | 1.95E+05 |
| **1061** | F1RX93 | Uncharacterized protein | MIEN1 | 2.18E+06 | 0.00E+00 | 5.47E+05 | 1.63E+06 | 2.72E+05 | 0.00E+00 | 6.83E+04 | 2.04E+05 |
| **1062** | Q96J80 | Mammalian ependymin related protein 1 | MERP1 | 5.43E+06 | 1.94E+06 | 1.52E+06 | 1.97E+06 | 2.71E+05 | 9.71E+04 | 7.58E+04 | 9.86E+04 |
| **1063** | F1SEN4 | Uncharacterized protein | LOC100517243 | 1.08E+06 | 0.00E+00 | 3.40E+05 | 7.45E+05 | 2.71E+05 | 0.00E+00 | 8.50E+04 | 1.86E+05 |
| **1064** | K7GPE2 | Eukaryotic translation initiation factor 1b | EIF1B | 1.35E+06 | 4.99E+05 | 5.47E+05 | 3.08E+05 | 2.71E+05 | 9.97E+04 | 1.09E+05 | 6.16E+04 |
| **1065** | F1S6R7 | Polypyrimidine tract-binding protein 1 | PTBP1 | 5.67E+06 | 1.79E+06 | 1.19E+06 | 2.69E+06 | 2.70E+05 | 8.54E+04 | 5.67E+04 | 1.28E+05 |
| **1066** | B2CNZ7 | Cathepsin B | CTSB | 4.59E+06 | 3.06E+06 | 4.04E+05 | 1.13E+06 | 2.70E+05 | 1.80E+05 | 2.38E+04 | 6.63E+04 |
| **1067** | F1SES5 | Uncharacterized protein | ASAH1 | 6.45E+06 | 2.05E+06 | 1.79E+06 | 2.62E+06 | 2.69E+05 | 8.53E+04 | 7.46E+04 | 1.09E+05 |
| **1068** | E9KL42 | Epididymis secretory sperm binding protein Li 188n | | 6.98E+06 | 2.19E+06 | 1.32E+06 | 3.48E+06 | 2.69E+05 | 8.43E+04 | 5.06E+04 | 1.34E+05 |
| **1069** | Q6IPN0 | Reticulon | RTN4 | 2.95E+06 | 2.95E+06 | 0.00E+00 | 0.00E+00 | 2.68E+05 | 2.68E+05 | 0.00E+00 | 0.00E+00 |
| **1070** | F1SB42 | Uncharacterized protein | EZR | 5.36E+06 | 1.00E+06 | 1.80E+06 | 2.56E+06 | 2.68E+05 | 5.01E+04 | 8.99E+04 | 1.28E+05 |
| **1071** | A7YB26 | Peroxisomal enoyl coenzyme A hydratase 1 (Fragment) | ECH1 | 4.27E+06 | 1.40E+06 | 9.07E+05 | 1.97E+06 | 2.67E+05 | 8.73E+04 | 5.67E+04 | 1.23E+05 |
| **1072** | F1S031 | NADH dehydrogenase [ubiquinone] iron-sulfur protein 6, mitochondrial | NDUFS6 | 2.40E+06 | 6.63E+05 | 1.16E+06 | 5.83E+05 | 2.67E+05 | 7.37E+04 | 1.28E+05 | 6.48E+04 |
| **1073** | K7ESJ4 | Programmed cell death protein 5 | PDCD5 | 1.06E+06 | 2.34E+05 | 5.51E+05 | 2.77E+05 | 2.65E+05 | 5.84E+04 | 1.38E+05 | 6.92E+04 |
| **1074** | B7Z1C9 | Chaperonin containing TCP1, subunit 7 (Eta), isoform CRA | CCT7 | 6.61E+06 | 5.85E+05 | 2.76E+06 | 3.27E+06 | 2.65E+05 | 2.34E+04 | 1.10E+05 | 1.31E+05 |
| **1075** | F1S3H7 | Uncharacterized protein | PTGR2 | 3.96E+06 | 3.03E+06 | 9.25E+05 | 0.00E+00 | 2.64E+05 | 2.02E+05 | 6.17E+04 | 0.00E+00 |
| **1076** | Q5T8U3 | 60S ribosomal protein L7a (Fragment) | RPL7A | 2.63E+06 | 4.63E+05 | 1.39E+06 | 7.78E+05 | 2.63E+05 | 4.63E+04 | 1.39E+05 | 7.78E+04 |
| **1077** | C9JFR7 | Cytochrome c (Fragment) | CYCS | 2.10E+06 | 0.00E+00 | 0.00E+00 | 2.10E+06 | 2.63E+05 | 0.00E+00 | 0.00E+00 | 2.63E+05 |
| **1078** | B5APU7 | Actin related protein 2/3 complex subunit 2 | | 6.03E+06 | 4.03E+06 | 0.00E+00 | 2.00E+06 | 2.62E+05 | 1.75E+05 | 0.00E+00 | 8.72E+04 |
| **1079** | F1SR76 | Uncharacterized protein | DNPEP | 5.76E+06 | 2.11E+06 | 2.36E+06 | 1.30E+06 | 2.62E+05 | 9.57E+04 | 1.07E+05 | 5.90E+04 |
| **1080** | F2Z5F2 | Uncharacterized protein | RAB10 | 4.44E+06 | 1.90E+06 | 4.21E+05 | 2.12E+06 | 2.61E+05 | 1.12E+05 | 2.48E+04 | 1.25E+05 |
| **1081** | P10114 | Ras-related protein Rap-2a | RAP2A | 3.13E+06 | 1.27E+06 | 4.84E+05 | 1.37E+06 | 2.60E+05 | 1.06E+05 | 4.03E+04 | 1.14E+05 |
| **1082** | B7Z871 | cDNA FLJ50930, highly similar to Lipoma-preferred partner | | 5.46E+06 | 3.64E+06 | 0.00E+00 | 1.82E+06 | 2.60E+05 | 1.73E+05 | 0.00E+00 | 8.66E+04 |
| **1083** | I3LDQ9 | Phosphotransferase | | 2.06E+06 | 1.93E+06 | 0.00E+00 | 1.31E+05 | 2.57E+05 | 2.41E+05 | 0.00E+00 | 1.64E+04 |
| **1084** | A0A0S2Z410 | Hydroxysteroid dehydrogenase 10 isoform 1 (Fragment) | HSD17B10 | 4.11E+06 | 1.94E+06 | 2.18E+06 | 0.00E+00 | 2.57E+05 | 1.21E+05 | 1.36E+05 | 0.00E+00 |
| **1085** | F1SQR4 | Uncharacterized protein | SNRPF | 1.29E+06 | 5.50E+05 | 3.06E+05 | 4.29E+05 | 2.57E+05 | 1.10E+05 | 6.12E+04 | 8.59E+04 |
| **1086** | F1RJJ3 | Uncharacterized protein | SBDS | 4.37E+06 | 3.36E+06 | 0.00E+00 | 1.01E+06 | 2.57E+05 | 1.98E+05 | 0.00E+00 | 5.92E+04 |
| **1087** | M3VK17 | CD276 molecule tv1 | CD276 | 6.42E+06 | 1.27E+06 | 1.59E+06 | 3.56E+06 | 2.57E+05 | 5.06E+04 | 6.37E+04 | 1.42E+05 |
| **1088** | I3LF44 | Uncharacterized protein | CPPED1 | 3.58E+06 | 1.55E+06 | 6.72E+05 | 1.36E+06 | 2.56E+05 | 1.11E+05 | 4.80E+04 | 9.72E+04 |
| **1089** | F1RVH5 | Peptidyl-prolyl cis-trans isomerase | LOC100524880 | 3.06E+06 | 4.50E+05 | 9.96E+05 | 1.62E+06 | 2.55E+05 | 3.75E+04 | 8.30E+04 | 1.35E+05 |
| **1090** | H0YJE9 | 26S protease regulatory subunit 10B | PSMC6 | 1.02E+06 | 4.71E+05 | 2.74E+05 | 2.74E+05 | 2.55E+05 | 1.18E+05 | 6.86E+04 | 6.84E+04 |
| **1091** | I3LC25 | Uncharacterized protein | LMNB2 | 1.07E+07 | 4.30E+06 | 1.93E+06 | 4.43E+06 | 2.54E+05 | 1.02E+05 | 4.60E+04 | 1.06E+05 |
| **1092** | H0YJN9 | Legumain (Fragment) | LGMN | 1.27E+06 | 4.09E+05 | 3.22E+05 | 5.36E+05 | 2.54E+05 | 8.18E+04 | 6.45E+04 | 1.07E+05 |
| **1093** | Q53FH6 | Mitogen-activated protein kinase kinase 1 interacting protein 1 variant (Fragment) | | 2.28E+06 | 8.74E+05 | 9.10E+05 | 4.91E+05 | 2.53E+05 | 9.72E+04 | 1.01E+05 | 5.46E+04 |
| **1094** | J3KS22 | L-xylulose reductase (Fragment) | DCXR | 3.79E+06 | 2.09E+06 | 8.98E+05 | 8.09E+05 | 2.53E+05 | 1.39E+05 | 5.99E+04 | 5.39E+04 |
| **1095** | L8AXM9 | IgG heavy chain | IGHG | 5.05E+06 | 1.05E+06 | 1.60E+06 | 2.39E+06 | 2.52E+05 | 5.26E+04 | 8.01E+04 | 1.20E+05 |
| **1096** | Q6I9V5 | SLC25A6 protein | SLC25A6 | 5.47E+06 | 3.43E+06 | 2.05E+06 | 0.00E+00 | 2.49E+05 | 1.56E+05 | 9.31E+04 | 0.00E+00 |
| **1097** | O11780 | Transforming growth factor-beta-induced protein ig-h3 | TGFBI | 1.02E+07 | 3.29E+06 | 2.80E+06 | 4.08E+06 | 2.48E+05 | 8.03E+04 | 6.83E+04 | 9.96E+04 |
| **1098** | K7GKY0 | Uncharacterized protein | CD109 | 1.76E+07 | 7.26E+06 | 5.61E+06 | 4.73E+06 | 2.48E+05 | 1.02E+05 | 7.90E+04 | 6.66E+04 |
| **1099** | F1SK98 | Uncharacterized protein | NRIP2 | 3.96E+06 | 1.30E+06 | 3.67E+05 | 2.30E+06 | 2.48E+05 | 8.14E+04 | 2.30E+04 | 1.43E+05 |
| **1100** | F1RNR7 | Uncharacterized protein | PSMD9 | 2.97E+06 | 1.80E+06 | 5.78E+05 | 5.86E+05 | 2.47E+05 | 1.50E+05 | 4.82E+04 | 4.89E+04 |
| **1101** | K7GRD9 | Uncharacterized protein (Fragment) | LAMP2 | 2.47E+06 | 1.47E+06 | 4.44E+05 | 5.54E+05 | 2.47E+05 | 1.47E+05 | 4.44E+04 | 5.54E+04 |
| **1102** | F1SAB6 | Acyl carrier protein | NDUFAB1 | 2.22E+06 | 7.02E+05 | 6.68E+05 | 8.51E+05 | 2.47E+05 | 7.80E+04 | 7.42E+04 | 9.46E+04 |
| **1103** | G9M4N3 | Toll interacting protein | Tollip | 3.45E+06 | 1.44E+06 | 1.04E+06 | 9.65E+05 | 2.46E+05 | 1.03E+05 | 7.44E+04 | 6.89E+04 |
| **1104** | F1SVA0 | Uncharacterized protein | PEF1 | 1.97E+06 | 1.24E+06 | 2.19E+05 | 5.04E+05 | 2.46E+05 | 1.55E+05 | 2.73E+04 | 6.30E+04 |
| **1105** | F1SL34 | Uncharacterized protein (Fragment) | ALCAM | 8.09E+06 | 5.82E+06 | 1.12E+06 | 1.16E+06 | 2.45E+05 | 1.76E+05 | 3.38E+04 | 3.51E+04 |
| **1106** | Q1KLJ4 | Proliferating cell nuclear antigen (Fragment) | PCNA | 2.70E+06 | 8.24E+05 | 7.54E+05 | 1.12E+06 | 2.45E+05 | 7.49E+04 | 6.86E+04 | 1.02E+05 |
| **1107** | F1SR53 | Uncharacterized protein | ITGA5 | 1.17E+07 | 5.20E+06 | 2.54E+06 | 3.95E+06 | 2.44E+05 | 1.08E+05 | 5.30E+04 | 8.24E+04 |
| **1108** | I3LCR9 | Uncharacterized protein | MYOM1 | 2.42E+07 | 1.54E+07 | 3.83E+06 | 4.97E+06 | 2.42E+05 | 1.54E+05 | 3.83E+04 | 4.97E+04 |
| **1109** | A0A024R4T4 | Ubiquitin-conjugating enzyme E2M (UBC12 homolog, yeast), isoform CRA | UBE2M | 2.90E+06 | 1.86E+06 | 3.43E+05 | 7.01E+05 | 2.42E+05 | 1.55E+05 | 2.86E+04 | 5.84E+04 |
| **1110** | B4DIC4 | cDNA FLJ52195, highly similar to LIM and SH3 domain protein 1 | LASP1 | 2.42E+06 | 2.42E+06 | 0.00E+00 | 0.00E+00 | 2.42E+05 | 2.42E+05 | 0.00E+00 | 0.00E+00 |
| **1111** | Q5TD07 | Ribosyldihydronicotinamide dehydrogenase [quinone] | NQO2 | 3.37E+06 | 5.53E+05 | 4.78E+05 | 2.34E+06 | 2.41E+05 | 3.95E+04 | 3.42E+04 | 1.67E+05 |
| **1112** | Q86W20 | Protease serine 1 (Fragment) | PRSS1 | 1.68E+06 | 1.45E+06 | 0.00E+00 | 2.30E+05 | 2.40E+05 | 2.07E+05 | 0.00E+00 | 3.29E+04 |
| **1113** | A0A0J9YYJ0 | Protein canopy homolog 4 (Fragment) | CNPY4 | 1.67E+06 | 6.74E+05 | 2.22E+05 | 7.70E+05 | 2.38E+05 | 9.63E+04 | 3.17E+04 | 1.10E+05 |
| **1114** | K7ENT6 | Tropomyosin alpha-4 chain (Fragment) | TPM4 | 4.04E+06 | 2.93E+06 | 1.11E+06 | 0.00E+00 | 2.38E+05 | 1.72E+05 | 6.55E+04 | 0.00E+00 |
| **1115** | F1SKL2 | Sulfurtransferase | TST | 4.50E+06 | 4.50E+06 | 0.00E+00 | 0.00E+00 | 2.37E+05 | 2.37E+05 | 0.00E+00 | 0.00E+00 |
| **1116** | E5RIK5 | Dynactin subunit 6 | DCTN6 | 4.73E+05 | 4.73E+05 | 0.00E+00 | 0.00E+00 | 2.36E+05 | 2.36E+05 | 0.00E+00 | 0.00E+00 |
| **1117** | D3K5N7 | ELAV-like protein | ELAVL1 | 4.22E+06 | 2.75E+06 | 1.07E+06 | 3.89E+05 | 2.34E+05 | 1.53E+05 | 5.96E+04 | 2.16E+04 |
| **1118** | Q2XVP5 | Microtubule-associated protein RP/EB family member 1 | MAPRE1 | 4.44E+06 | 3.19E+06 | 3.26E+05 | 9.26E+05 | 2.34E+05 | 1.68E+05 | 1.71E+04 | 4.87E+04 |
| **1119** | Q8HXL4 | Cytoskeleton-associated protein 1 | CKAP1 | 4.20E+06 | 2.58E+06 | 1.12E+06 | 4.92E+05 | 2.33E+05 | 1.43E+05 | 6.25E+04 | 2.73E+04 |
| **1120** | B7Z2V6 | cDNA FLJ53889 | ATP6V1A | 5.34E+06 | 1.24E+06 | 1.51E+06 | 2.60E+06 | 2.32E+05 | 5.39E+04 | 6.54E+04 | 1.13E+05 |
| **1121** | B2RCZ7 | Ethylmalonic encephalopathy 1, isoform CRA | ETHE1 | 3.48E+06 | 2.56E+06 | 0.00E+00 | 9.17E+05 | 2.32E+05 | 1.71E+05 | 0.00E+00 | 6.11E+04 |
| **1122** | C9JQ41 | Coiled-coil domain-containing protein 58 | CCDC58 | 1.62E+06 | 6.03E+05 | 1.65E+05 | 8.55E+05 | 2.32E+05 | 8.61E+04 | 2.36E+04 | 1.22E+05 |
| **1123** | F1S192 | Uncharacterized protein | PFDN2 | 2.78E+06 | 8.43E+05 | 5.53E+05 | 1.38E+06 | 2.31E+05 | 7.03E+04 | 4.61E+04 | 1.15E+05 |
| **1124** | K7GP35 | Uncharacterized protein | PLP2 | 9.18E+05 | 0.00E+00 | 1.38E+05 | 7.80E+05 | 2.30E+05 | 0.00E+00 | 3.45E+04 | 1.95E+05 |
| **1125** | Q53SS8 | Epididymis secretory protein Li 85 | PCBP1 | 4.81E+06 | 3.23E+06 | 9.52E+05 | 6.28E+05 | 2.29E+05 | 1.54E+05 | 4.54E+04 | 2.99E+04 |
| **1126** | F1SII1 | Uncharacterized protein | LOC100624445 | 2.06E+06 | 9.15E+05 | 4.19E+05 | 7.24E+05 | 2.29E+05 | 1.02E+05 | 4.66E+04 | 8.04E+04 |
| **1127** | F1S4V8 | Uncharacterized protein | CPNE1 | 5.26E+06 | 5.27E+05 | 2.74E+06 | 1.99E+06 | 2.29E+05 | 2.29E+04 | 1.19E+05 | 8.64E+04 |
| **1128** | Q8MJ49 | teoclast-stimulating factor 1 | OSTF1 | 2.97E+06 | 2.02E+06 | 6.15E+05 | 3.29E+05 | 2.28E+05 | 1.56E+05 | 4.73E+04 | 2.53E+04 |
| **1129** | H0YLY7 | Calcineurin B homologous protein 1 (Fragment) | CHP1 | 1.59E+06 | 5.32E+05 | 0.00E+00 | 1.06E+06 | 2.27E+05 | 7.60E+04 | 0.00E+00 | 1.51E+05 |
| **1130** | F1S8G6 | Uncharacterized protein | SGTA | 3.17E+06 | 3.17E+06 | 0.00E+00 | 0.00E+00 | 2.27E+05 | 2.27E+05 | 0.00E+00 | 0.00E+00 |
| **1131** | A0A0A0MSW4 | Phosphatidylinositol transfer protein beta isoform | PITPNB | 5.43E+06 | 4.28E+06 | 1.15E+06 | 0.00E+00 | 2.26E+05 | 1.78E+05 | 4.77E+04 | 0.00E+00 |
| **1132** | A0A024RCA7 | Ribosomal protein, large, P2, isoform CRA | RPLP2 | 1.13E+06 | 0.00E+00 | 0.00E+00 | 1.13E+06 | 2.26E+05 | 0.00E+00 | 0.00E+00 | 2.26E+05 |
| **1133** | F1RUE0 | Uncharacterized protein | ACOT13 | 1.80E+06 | 1.57E+06 | 2.32E+05 | 0.00E+00 | 2.25E+05 | 1.96E+05 | 2.90E+04 | 0.00E+00 |
| **1134** | F1S750 | Uncharacterized protein | ECHDC2 | 2.68E+06 | 2.16E+06 | 5.22E+05 | 0.00E+00 | 2.23E+05 | 1.80E+05 | 4.35E+04 | 0.00E+00 |
| **1135** | F1S2X3 | Uncharacterized protein | ECHDC1 | 4.69E+06 | 1.91E+06 | 8.14E+05 | 1.96E+06 | 2.23E+05 | 9.12E+04 | 3.87E+04 | 9.33E+04 |
| **1136** | B4E3P1 | cDNA FLJ57036 | | 4.90E+06 | 0.00E+00 | 4.90E+06 | 0.00E+00 | 2.23E+05 | 0.00E+00 | 2.23E+05 | 0.00E+00 |
| **1137** | Q6FHM6 | NHP2 non-histone chromosome protein 2-like 1 (S. cerevisiae) | NHP2L1 | 2.21E+06 | 1.30E+06 | 4.53E+05 | 4.56E+05 | 2.21E+05 | 1.30E+05 | 4.53E+04 | 4.56E+04 |
| **1138** | I3LM66 | Uncharacterized protein | | 1.54E+06 | 1.24E+06 | 0.00E+00 | 3.00E+05 | 2.20E+05 | 1.77E+05 | 0.00E+00 | 4.29E+04 |
| **1139** | Q6FH24 | Prefoldin subunit 3 | VBP1 | 2.86E+06 | 2.37E+06 | 0.00E+00 | 4.88E+05 | 2.20E+05 | 1.83E+05 | 0.00E+00 | 3.75E+04 |
| **1140** | F1RIP6 | Uncharacterized protein | NUCB1 | 6.16E+06 | 1.90E+06 | 3.03E+06 | 1.23E+06 | 2.20E+05 | 6.78E+04 | 1.08E+05 | 4.38E+04 |
| **1141** | F1RWI4 | Uncharacterized protein | SBSPON | 2.18E+06 | 1.06E+06 | 5.10E+05 | 6.09E+05 | 2.18E+05 | 1.06E+05 | 5.10E+04 | 6.09E+04 |
| **1142** | A0A0S2Z4G7 | Nucleophosmin (Nucleolar phosphoprotein B23, numatrin), isoform CRA | NPM1 | 3.26E+06 | 8.14E+05 | 1.86E+06 | 5.84E+05 | 2.17E+05 | 5.42E+04 | 1.24E+05 | 3.90E+04 |
| **1143** | Q7Z4Q5 | Heterogeneous nuclear ribonucleoprotein U (Scaffold attachment factor A), isoform CRA | HNRPU | 8.44E+06 | 4.67E+06 | 2.10E+06 | 1.67E+06 | 2.16E+05 | 1.20E+05 | 5.38E+04 | 4.28E+04 |
| **1144** | F1RXD5 | Uncharacterized protein | CPNE3 | 5.17E+06 | 1.93E+06 | 1.56E+06 | 1.69E+06 | 2.16E+05 | 8.03E+04 | 6.48E+04 | 7.04E+04 |
| **1145** | F1S7E0 | Uncharacterized protein | COPE | 4.50E+06 | 3.64E+06 | 0.00E+00 | 8.52E+05 | 2.14E+05 | 1.74E+05 | 0.00E+00 | 4.06E+04 |
| **1146** | B6CVD7 | ERO1-like protein alpha | ERO1A | 6.82E+06 | 0.00E+00 | 3.82E+06 | 3.00E+06 | 2.13E+05 | 0.00E+00 | 1.19E+05 | 9.36E+04 |
| **1147** | I3LUL5 | Uncharacterized protein | LOC100624689 | 7.45E+06 | 7.45E+06 | 0.00E+00 | 0.00E+00 | 2.13E+05 | 2.13E+05 | 0.00E+00 | 0.00E+00 |
| **1148** | P51779 | Complement factor D | CFD | 3.40E+06 | 2.72E+06 | 0.00E+00 | 6.75E+05 | 2.12E+05 | 1.70E+05 | 0.00E+00 | 4.22E+04 |
| **1149** | J3QKT5 | RNA-binding protein Musashi homolog 2 | MSI2 | 1.06E+06 | 5.72E+05 | 4.86E+05 | 0.00E+00 | 2.11E+05 | 1.14E+05 | 9.71E+04 | 0.00E+00 |
| **1150** | F1SJ30 | Mannose-6-phosphate isomerase | MPI | 4.86E+06 | 1.76E+06 | 1.32E+06 | 1.77E+06 | 2.11E+05 | 7.66E+04 | 5.76E+04 | 7.70E+04 |
| **1151** | Q5K4G2 | Lysosome-associated membrane glycoprotein 1 | LAMP-1 | 3.99E+06 | 1.13E+06 | 1.07E+06 | 1.79E+06 | 2.10E+05 | 5.95E+04 | 5.64E+04 | 9.41E+04 |
| **1152** | F1RRU7 | Uncharacterized protein (Fragment) | MRC2 | 1.37E+07 | 6.38E+06 | 3.63E+06 | 3.64E+06 | 2.10E+05 | 9.81E+04 | 5.59E+04 | 5.60E+04 |
| **1153** | B5ATG0 | Clathrin light chain (CLTA) protein (Fragment) | CLTA | 1.88E+06 | 1.12E+06 | 0.00E+00 | 7.64E+05 | 2.09E+05 | 1.24E+05 | 0.00E+00 | 8.48E+04 |
| **1154** | K7GMK3 | Uncharacterized protein (Fragment) | PIR | 2.50E+06 | 8.88E+05 | 5.80E+05 | 1.03E+06 | 2.09E+05 | 7.40E+04 | 4.83E+04 | 8.62E+04 |
| **1155** | Q09666 | Neuroblast differentiation-associated protein AHNAK | AHNAK | 8.58E+07 | 5.03E+07 | 1.85E+07 | 1.70E+07 | 2.08E+05 | 1.22E+05 | 4.50E+04 | 4.12E+04 |
| **1156** | C9JL25 | 60 kDa heat shock protein, mitochondrial (Fragment) | HSPD1 | 2.49E+06 | 7.49E+05 | 0.00E+00 | 1.74E+06 | 2.08E+05 | 6.24E+04 | 0.00E+00 | 1.45E+05 |
| **1157** | Q7YS20 | Lactoferrin | LTF | 8.91E+06 | 3.66E+06 | 2.76E+06 | 2.49E+06 | 2.07E+05 | 8.51E+04 | 6.41E+04 | 5.80E+04 |
| **1158** | F8VVM2 | Phosphate carrier protein, mitochondrial | SLC25A3 | 3.93E+06 | 2.50E+06 | 9.23E+05 | 5.06E+05 | 2.07E+05 | 1.32E+05 | 4.86E+04 | 2.66E+04 |
| **1159** | F1SP56 | Uncharacterized protein | NIPSNAP3A | 3.31E+06 | 1.90E+06 | 0.00E+00 | 1.41E+06 | 2.07E+05 | 1.19E+05 | 0.00E+00 | 8.84E+04 |
| **1160** | E7EMV2 | Neurofilament medium polypeptide | NEFM | 9.50E+06 | 8.97E+06 | 2.30E+05 | 3.02E+05 | 2.07E+05 | 1.95E+05 | 5.01E+03 | 6.57E+03 |
| **1161** | B4DDW1 | cDNA FLJ52156, moderately similar to Mus musculus methyltransferase like 7A (Mettl7a), mRNA | METTL7A | 1.86E+06 | 1.86E+06 | 0.00E+00 | 0.00E+00 | 2.07E+05 | 2.07E+05 | 0.00E+00 | 0.00E+00 |
| **1162** | I3LJA0 | Uncharacterized protein | DDX52 | 5.78E+06 | 3.59E+06 | 1.76E+06 | 4.34E+05 | 2.06E+05 | 1.28E+05 | 6.28E+04 | 1.55E+04 |
| **1163** | M0QZ21 | AP-2 complex subunit sigma | AP2S1 | 1.23E+06 | 1.23E+06 | 0.00E+00 | 0.00E+00 | 2.05E+05 | 2.05E+05 | 0.00E+00 | 0.00E+00 |
| **1164** | Q5JPU2 | Pyruvate dehydrogenase E1 component subunit alpha, somatic form, mitochondrial (Fragment) | PDHA1 | 2.05E+06 | 1.18E+06 | 8.65E+05 | 0.00E+00 | 2.05E+05 | 1.18E+05 | 8.65E+04 | 0.00E+00 |
| **1165** | Q29243 | Dystroglycan | DAG1 | 9.62E+06 | 5.18E+06 | 1.85E+06 | 2.59E+06 | 2.05E+05 | 1.10E+05 | 3.93E+04 | 5.52E+04 |
| **1166** | A0A0J9YX89 | 15 kDa selenoprotein (Fragment) | SEPT15 | 1.42E+06 | 5.17E+05 | 6.36E+05 | 2.71E+05 | 2.03E+05 | 7.39E+04 | 9.09E+04 | 3.87E+04 |
| **1167** | I3LPU8 | Uncharacterized protein | HNRNPL | 2.44E+06 | 1.88E+06 | 5.55E+05 | 0.00E+00 | 2.03E+05 | 1.57E+05 | 4.62E+04 | 0.00E+00 |
| **1168** | Q29197 | 40S ribosomal protein S9 (Fragment) | RPS9 | 2.01E+06 | 0.00E+00 | 2.01E+06 | 0.00E+00 | 2.01E+05 | 0.00E+00 | 2.01E+05 | 0.00E+00 |
| **1169** | F1RNW4 | Uncharacterized protein | LOC100739263 | 2.61E+06 | 1.38E+06 | 1.23E+06 | 0.00E+00 | 2.01E+05 | 1.06E+05 | 9.46E+04 | 0.00E+00 |
| **1170** | I3LPP6 | Uncharacterized protein | | 1.21E+06 | 1.21E+06 | 0.00E+00 | 0.00E+00 | 2.01E+05 | 2.01E+05 | 0.00E+00 | 0.00E+00 |
| **1171** | D6RJ91 | Myosin light chain 5 (Fragment) | MYL5 | 2.00E+06 | 1.38E+06 | 0.00E+00 | 6.22E+05 | 2.00E+05 | 1.38E+05 | 0.00E+00 | 6.22E+04 |
| **1172** | F2Z5K9 | Histone H3 | HIST2H3D | 1.20E+06 | 1.20E+06 | 0.00E+00 | 0.00E+00 | 2.00E+05 | 2.00E+05 | 0.00E+00 | 0.00E+00 |
| **1173** | I3LNG8 | Uncharacterized protein | STIP1 | 5.99E+06 | 1.72E+06 | 3.23E+06 | 1.03E+06 | 2.00E+05 | 5.75E+04 | 1.08E+05 | 3.44E+04 |
| **1174** | F1RWY7 | Uncharacterized protein | BPHL | 3.77E+06 | 1.14E+06 | 1.18E+06 | 1.45E+06 | 1.98E+05 | 5.97E+04 | 6.22E+04 | 7.64E+04 |
| **1175** | A0A0S2Z3X8 | GDP dissociation inhibitor 1 isoform 1 (Fragment) | GDI1 | 5.35E+06 | 0.00E+00 | 0.00E+00 | 5.35E+06 | 1.98E+05 | 0.00E+00 | 0.00E+00 | 1.98E+05 |
| **1176** | Q6J2R3 | Myelin basic protein | MBP | 2.76E+06 | 4.96E+05 | 9.59E+05 | 1.31E+06 | 1.97E+05 | 3.54E+04 | 6.85E+04 | 9.36E+04 |
| **1177** | K7ELP0 | Tropomyosin alpha-4 chain (Fragment) | TPM4 | 9.87E+05 | 9.87E+05 | 0.00E+00 | 0.00E+00 | 1.97E+05 | 1.97E+05 | 0.00E+00 | 0.00E+00 |
| **1178** | A0A0J9YYJ1 | Serum paraoxonase/arylesterase 2 (Fragment) | PON2 | 1.17E+06 | 0.00E+00 | 1.54E+05 | 1.02E+06 | 1.96E+05 | 0.00E+00 | 2.57E+04 | 1.70E+05 |
| **1179** | I3L5W3 | Ficolin-2 | FCN2 | 3.52E+06 | 1.77E+06 | 1.75E+06 | 0.00E+00 | 1.96E+05 | 9.85E+04 | 9.71E+04 | 0.00E+00 |
| **1180** | M0R1Y1 | EVI5-like protein (Fragment) | EVI5L | 1.57E+06 | 1.57E+06 | 0.00E+00 | 0.00E+00 | 1.96E+05 | 1.96E+05 | 0.00E+00 | 0.00E+00 |

**Supplementary Table S1: (continued)**

| | | | | | | | | | | | |
|---|---|---|---|---|---|---|---|---|---|---|---|
| **1181** | F1S788 | Uncharacterized protein | C8A | 6.06E+06 | 0.00E+00 | 2.41E+06 | 3.64E+06 | 1.95E+05 | 0.00E+00 | 7.79E+04 | 1.17E+05 |
| **1182** | I3L6T2 | Uncharacterized protein | UBE2V2 | 2.15E+06 | 1.65E+06 | 0.00E+00 | 5.01E+05 | 1.95E+05 | 1.50E+05 | 0.00E+00 | 4.56E+04 |
| **1183** | I6XPV3 | Voltage-dependent anion channel 3 (Fragment) | VDAC3 | 2.92E+06 | 1.13E+06 | 6.64E+05 | 1.12E+06 | 1.95E+05 | 7.56E+04 | 4.43E+04 | 7.47E+04 |
| **1184** | F1S5Z8 | Uncharacterized protein | SYPL2 | 2.14E+06 | 1.44E+06 | 7.03E+05 | 0.00E+00 | 1.95E+05 | 1.31E+05 | 6.39E+04 | 0.00E+00 |
| **1185** | B1PK17 | Aspartoacylase | ASPA | 4.27E+06 | 3.28E+06 | 0.00E+00 | 9.87E+05 | 1.94E+05 | 1.49E+05 | 0.00E+00 | 4.48E+04 |
| **1186** | A0A0A0MS07 | Ig gamma-1 chain C region (Fragment) | IGHG1 | 2.51E+06 | 2.51E+06 | 0.00E+00 | 0.00E+00 | 1.93E+05 | 1.93E+05 | 0.00E+00 | 0.00E+00 |
| **1187** | Q6JLA8 | Four and a half LIM domains 3 (Fragment) | FHL3 | 2.31E+06 | 1.28E+06 | 7.57E+05 | 2.71E+05 | 1.93E+05 | 1.07E+05 | 6.31E+04 | 2.26E+04 |
| **1188** | C9JDZ2 | Fas apoptotic inhibitory molecule 1 (Fragment) | FAIM | 1.35E+06 | 3.25E+05 | 1.44E+05 | 8.78E+05 | 1.92E+05 | 4.64E+04 | 2.06E+04 | 1.25E+05 |
| **1189** | I3LNB4 | Uncharacterized protein | ALDH4A1 | 5.95E+06 | 1.56E+06 | 2.92E+06 | 1.48E+06 | 1.92E+05 | 5.03E+04 | 9.41E+04 | 4.76E+04 |
| **1190** | F1RWL7 | NADH dehydrogenase [ubiquinone] 1 beta subcomplex subunit 8, mitochondrial | NDUFB8 | 2.30E+06 | 2.11E+06 | 1.96E+05 | 0.00E+00 | 1.92E+05 | 1.75E+05 | 1.64E+04 | 0.00E+00 |
| **1191** | Q8MIZ3 | m7GpppX diphosphatase | DCPS | 3.83E+06 | 1.52E+06 | 1.53E+06 | 7.84E+05 | 1.91E+05 | 7.60E+04 | 7.63E+04 | 3.92E+04 |
| **1192** | Q9BS10 | Similar to ribosomal protein S8 (Fragment) | RPS8 | 1.14E+06 | 3.31E+05 | 3.03E+05 | 5.06E+05 | 1.90E+05 | 5.52E+04 | 5.05E+04 | 8.44E+04 |
| **1193** | F8VXH9 | Poly(rC)-binding protein 2 (Fragment) | PCBP2 | 2.27E+06 | 1.49E+06 | 7.83E+05 | 0.00E+00 | 1.89E+05 | 1.24E+05 | 6.53E+04 | 0.00E+00 |
| **1194** | Q8SQ26 | Lambda-crystallin homolog | CRYL1 | 3.60E+06 | 2.79E+06 | 0.00E+00 | 8.04E+05 | 1.89E+05 | 1.47E+05 | 0.00E+00 | 4.23E+04 |
| **1195** | F1SAE9 | Uncharacterized protein | LAMB1 | 1.77E+07 | 4.37E+06 | 6.25E+06 | 7.07E+06 | 1.88E+05 | 4.65E+04 | 6.65E+04 | 7.52E+04 |
| **1196** | F1SEC0 | Uncharacterized protein | DCTN3 | 2.43E+06 | 7.25E+05 | 3.51E+05 | 1.36E+06 | 1.87E+05 | 5.58E+04 | 2.70E+04 | 1.04E+05 |
| **1197** | F1RZD0 | Uncharacterized protein | FKBP7 | 3.37E+06 | 1.60E+06 | 6.48E+05 | 1.12E+06 | 1.87E+05 | 8.88E+04 | 3.60E+04 | 6.24E+04 |
| **1198** | F1SI09 | Uncharacterized protein | PPP1R7 | 4.10E+06 | 2.27E+06 | 8.86E+05 | 9.43E+05 | 1.86E+05 | 1.03E+05 | 4.03E+04 | 4.29E+04 |
| **1199** | V9HW80 | Epididymis luminal protein 220 | HEL-S-70 | 8.93E+06 | 2.74E+06 | 2.03E+06 | 4.16E+06 | 1.86E+05 | 5.71E+04 | 4.23E+04 | 8.67E+04 |
| **1200** | I3LMU6 | Uncharacterized protein | RCN3 | 2.60E+06 | 1.55E+06 | 0.00E+00 | 1.05E+06 | 1.85E+05 | 1.10E+05 | 0.00E+00 | 7.50E+04 |
| **1201** | F1SK86 | Uncharacterized protein | FKBP4 | 6.62E+06 | 5.97E+05 | 3.36E+06 | 2.66E+06 | 1.84E+05 | 1.66E+04 | 9.35E+04 | 7.39E+04 |
| **1202** | P63096 | Guanine nucleotide-binding protein G(i) subunit alpha-1 | GNAI1 | 3.12E+06 | 2.19E+06 | 9.35E+05 | 0.00E+00 | 1.84E+05 | 1.29E+05 | 5.50E+04 | 0.00E+00 |
| **1203** | F1RIE2 | Uncharacterized protein | DDB1 | 1.06E+07 | 2.55E+06 | 1.44E+06 | 6.59E+06 | 1.82E+05 | 4.39E+04 | 2.48E+04 | 1.14E+05 |
| **1204** | Q19PY1 | Alpha-1,4 glucan phosphorylase (Fragment) | PYGM | 9.11E+06 | 4.03E+06 | 2.18E+06 | 2.90E+06 | 1.82E+05 | 8.06E+04 | 4.35E+04 | 5.80E+04 |
| **1205** | E5RHX8 | Transcription and mRNA export factor ENY2 | ENY2 | 1.26E+06 | 3.36E+05 | 4.91E+05 | 4.37E+05 | 1.81E+05 | 4.80E+04 | 7.02E+04 | 6.25E+04 |
| **1206** | I3LJW4 | Aconitate hydratase | ACO1 | 8.84E+06 | 4.45E+06 | 2.09E+06 | 2.30E+06 | 1.80E+05 | 9.08E+04 | 4.27E+04 | 4.69E+04 |
| **1207** | F1RLQ2 | Prelamin-A/C | LMNA | 7.91E+06 | 5.03E+06 | 2.19E+06 | 6.89E+05 | 1.80E+05 | 1.14E+05 | 4.98E+04 | 1.57E+04 |
| **1208** | K9IVR9 | Importin subunit beta-1 | KPNB1 | 7.89E+06 | 3.74E+06 | 2.48E+06 | 1.67E+06 | 1.79E+05 | 8.51E+04 | 5.63E+04 | 3.79E+04 |
| **1209** | F1RPY1 | Uncharacterized protein | ATL3 | 5.36E+06 | 2.60E+06 | 1.78E+06 | 9.78E+05 | 1.79E+05 | 8.67E+04 | 5.93E+04 | 3.26E+04 |
| **1210** | A0A087WXS7 | ATPase ASNA1 | ASNA1 | 2.83E+06 | 1.71E+06 | 1.12E+06 | 0.00E+00 | 1.77E+05 | 1.07E+05 | 6.98E+04 | 0.00E+00 |
| **1211** | I3LBB2 | Vacuolar protein sorting-associated protein 35 | VPS35 | 6.66E+06 | 1.60E+06 | 1.59E+06 | 3.46E+06 | 1.75E+05 | 4.22E+04 | 4.20E+04 | 9.12E+04 |
| **1212** | B7Z230 | cDNA FLJ53833, highly similar to FK506-binding protein 9 (EC 5.2.1.8) | FKBP9 | 3.85E+06 | 1.55E+06 | 1.53E+06 | 7.57E+05 | 1.75E+05 | 7.06E+04 | 6.97E+04 | 3.44E+04 |
| **1213** | A0A0E3KLW5 | Nucleoside diphosphate linked moiety X-type motif 3 | NUDT3 | 1.92E+06 | 7.52E+05 | 4.35E+05 | 7.34E+05 | 1.75E+05 | 6.84E+04 | 3.95E+04 | 6.67E+04 |
| **1214** | E5RI90 | Uncharacterized protein C1orf198 (Fragment) | C1orf198 | 1.90E+06 | 1.90E+06 | 0.00E+00 | 0.00E+00 | 1.72E+05 | 1.72E+05 | 0.00E+00 | 0.00E+00 |
| **1215** | G3V5X4 | Nesprin-2 | SYNE2 | 7.73E+07 | 3.30E+07 | 0.00E+00 | 4.43E+07 | 1.72E+05 | 7.35E+04 | 0.00E+00 | 9.87E+04 |
| **1216** | O97580 | Succinyl-CoA ligase [ADP-forming] subunit beta, mitochondrial (Fragment) | SUCLA2 | 4.41E+06 | 3.55E+06 | 8.62E+05 | 0.00E+00 | 1.70E+05 | 1.36E+05 | 3.32E+04 | 0.00E+00 |
| **1217** | F8VUA6 | 60S ribosomal protein L18 (Fragment) | RPL18 | 1.01E+06 | 0.00E+00 | 1.01E+06 | 0.00E+00 | 1.69E+05 | 0.00E+00 | 1.69E+05 | 0.00E+00 |
| **1218** | F1RT62 | Uncharacterized protein | ITGA3 | 7.41E+06 | 1.80E+06 | 1.29E+06 | 4.32E+06 | 1.68E+05 | 4.10E+04 | 2.94E+04 | 9.81E+04 |
| **1219** | Q6Q2J0 | Amine oxidase [flavin-containing] A | MAOA | 4.54E+06 | 1.45E+06 | 7.33E+05 | 2.36E+06 | 1.68E+05 | 5.36E+04 | 2.71E+04 | 8.72E+04 |
| **1220** | I3LHZ6 | Uncharacterized protein | DHX9 | 1.24E+07 | 7.18E+06 | 2.28E+06 | 2.96E+06 | 1.68E+05 | 9.71E+04 | 3.08E+04 | 4.00E+04 |
| **1221** | I3LJY8 | Uncharacterized protein | HSD17B12 | 1.98E+06 | 0.00E+00 | 6.13E+05 | 1.37E+06 | 1.65E+05 | 0.00E+00 | 5.11E+04 | 1.14E+05 |
| **1222** | Q9NZ48 | Uncharacterized hematopoietic stem/progenitor cells protein MDS026 | PPP | 1.32E+06 | 2.71E+05 | 2.90E+05 | 7.55E+05 | 1.64E+05 | 3.39E+04 | 3.63E+04 | 9.43E+04 |
| **1223** | H3BNG3 | 40S ribosomal protein S2 (Fragment) | RPS2 | 3.28E+05 | 0.00E+00 | 3.28E+05 | 0.00E+00 | 1.64E+05 | 0.00E+00 | 1.64E+05 | 0.00E+00 |
| **1224** | B5KN59 | NADH-ubiquinone oxidoreductase chain 1 | ND1 | 1.15E+06 | 2.36E+05 | 2.81E+05 | 6.28E+05 | 1.64E+05 | 3.38E+04 | 4.02E+04 | 8.97E+04 |
| **1225** | B3KNZ3 | cDNA FLJ30801 fis, clone FEBRA2001217, highly similar to Homo sapiens LIM and senescent cell antigen-like domains 2 (LIMS2), mRNA | | 3.59E+06 | 3.29E+06 | 2.97E+05 | 0.00E+00 | 1.63E+05 | 1.50E+05 | 1.35E+04 | 0.00E+00 |
| **1226** | P02584 | Profilin-1 | | 1.47E+06 | 5.34E+05 | 7.09E+05 | 2.25E+05 | 1.63E+05 | 5.93E+04 | 7.88E+04 | 2.50E+04 |
| **1227** | A6NJH9 | Eukaryotic translation initiation factor 1A, Y-chromosomal | EIF1AY | 1.30E+06 | 1.30E+06 | 0.00E+00 | 0.00E+00 | 1.62E+05 | 1.62E+05 | 0.00E+00 | 0.00E+00 |
| **1228** | F1SEN1 | Annexin | ANXA8 | 3.86E+06 | 2.23E+06 | 1.63E+06 | 0.00E+00 | 1.61E+05 | 9.29E+04 | 6.79E+04 | 0.00E+00 |
| **1229** | Q0VGA5 | SARS protein | SARS | 4.98E+06 | 8.57E+05 | 2.39E+06 | 1.74E+06 | 1.61E+05 | 2.77E+04 | 7.70E+04 | 5.61E+04 |
| **1230** | D0G0B3 | Acetyl-Coenzyme A acyltransferase 2 | ACAA2 | 3.53E+06 | 1.48E+06 | 8.37E+05 | 1.22E+06 | 1.61E+05 | 6.72E+04 | 3.80E+04 | 5.54E+04 |
| **1231** | M3V839 | Mitogen-activated protein kinase | MAPK3 | 3.69E+06 | 3.69E+06 | 0.00E+00 | 0.00E+00 | 1.60E+05 | 1.60E+05 | 0.00E+00 | 0.00E+00 |
| **1232** | F1S6K5 | Uncharacterized protein | APRT | 2.23E+06 | 1.23E+06 | 5.39E+05 | 4.60E+05 | 1.59E+05 | 8.80E+04 | 3.85E+04 | 3.29E+04 |
| **1233** | M0R2L9 | 40S ribosomal protein S19 (Fragment) | RPS19 | 9.51E+05 | 7.83E+05 | 0.00E+00 | 1.68E+05 | 1.58E+05 | 1.30E+05 | 0.00E+00 | 2.80E+04 |
| **1234** | Q9TV36 | Fibrillin-1 | FBN1 | 2.57E+07 | 1.38E+07 | 1.19E+07 | 0.00E+00 | 1.58E+05 | 8.49E+04 | 7.28E+04 | 0.00E+00 |
| **1235** | B8ZZ77 | Peptidyl-prolyl cis-trans isomerase | PPIL3 | 2.19E+06 | 9.44E+05 | 4.89E+05 | 7.60E+05 | 1.57E+05 | 6.74E+04 | 3.49E+04 | 5.43E+04 |
| **1236** | Q06AB3 | Ubiquitin carboxyl-terminal hydrolase isozyme L3 | UCHL3 | 2.03E+06 | 1.09E+06 | 9.39E+05 | 0.00E+00 | 1.56E+05 | 8.38E+04 | 7.22E+04 | 0.00E+00 |
| **1237** | A8KAQ5 | cDNA FLJ77404, highly similar to Homo sapiens small nuclear ribonucleoprotein 70kDa polypeptide (RNP antigen) (SNRP70), transcript variant 1, mRNA | | 2.96E+06 | 2.25E+06 | 7.17E+05 | 0.00E+00 | 1.56E+05 | 1.18E+05 | 3.77E+04 | 0.00E+00 |
| **1238** | A0A140VJZ1 | Testicular tissue protein Li 218 | | 7.00E+06 | 3.29E+06 | 1.75E+06 | 1.96E+06 | 1.56E+05 | 7.31E+04 | 3.89E+04 | 4.35E+04 |
| **1239** | F1RFE2 | Uncharacterized protein | | 4.32E+06 | 0.00E+00 | 0.00E+00 | 4.32E+06 | 1.54E+05 | 0.00E+00 | 0.00E+00 | 1.54E+05 |
| **1240** | F1SP18 | Uncharacterized protein | TARS | 6.16E+06 | 1.20E+06 | 1.21E+06 | 3.75E+06 | 1.54E+05 | 3.01E+04 | 3.02E+04 | 9.37E+04 |
| **1241** | B1AK87 | Capping protein (Actin filament) muscle Z-line, beta, isoform CRA | CAPZB | 2.76E+06 | 1.21E+06 | 3.53E+05 | 1.20E+06 | 1.54E+05 | 6.75E+04 | 1.96E+04 | 6.64E+04 |
| **1242** | F1SPG9 | Uncharacterized protein | ESYT1 | 8.89E+06 | 3.48E+06 | 2.74E+06 | 2.67E+06 | 1.53E+05 | 6.00E+04 | 4.73E+04 | 4.60E+04 |
| **1243** | Q29529 | Carbonyl reductase [NADPH] 2 | CBR2 | 2.29E+06 | 1.34E+06 | 9.59E+05 | 0.00E+00 | 1.53E+05 | 8.90E+04 | 6.40E+04 | 0.00E+00 |
| **1244** | A0A0K0K1J6 | Epididymis secretory protein Li 96 (Fragment) | HEL-S-96 | 1.37E+06 | 0.00E+00 | 1.37E+06 | 0.00E+00 | 1.53E+05 | 0.00E+00 | 1.53E+05 | 0.00E+00 |
| **1245** | P12271 | Retinaldehyde-binding protein 1 | RLBP1 | 2.74E+06 | 2.35E+06 | 0.00E+00 | 3.83E+05 | 1.52E+05 | 1.31E+05 | 0.00E+00 | 2.13E+04 |
| **1246** | I3LHS7 | Uncharacterized protein | COQ9 | 2.73E+06 | 2.03E+06 | 5.59E+05 | 1.44E+05 | 1.52E+05 | 1.13E+05 | 3.10E+04 | 8.00E+03 |
| **1247** | B5B2Z3 | Alpha v integrin subunit | ITGAV | 9.31E+06 | 4.82E+06 | 3.00E+06 | 1.50E+06 | 1.50E+05 | 7.77E+04 | 4.83E+04 | 2.42E+04 |
| **1248** | F1S193 | Uncharacterized protein | NIT1 | 2.69E+06 | 1.60E+06 | 6.59E+05 | 4.33E+05 | 1.50E+05 | 8.90E+04 | 3.66E+04 | 2.41E+04 |
| **1249** | Q29554 | Trifunctional enzyme subunit alpha, mitochondrial | HADHA | 6.70E+06 | 2.38E+06 | 1.84E+06 | 2.47E+06 | 1.49E+05 | 5.30E+04 | 4.10E+04 | 5.49E+04 |
| **1250** | Q6JHY5 | MHC class II antigen (Fragment) | SLA-DQB1 | 2.68E+06 | 2.68E+06 | 0.00E+00 | 0.00E+00 | 1.49E+05 | 1.49E+05 | 0.00E+00 | 0.00E+00 |
| **1251** | Q05CK9 | SYNCRIP protein (Fragment) | SYNCRIP | 4.12E+06 | 1.65E+06 | 2.46E+06 | 0.00E+00 | 1.47E+05 | 5.91E+04 | 8.80E+04 | 0.00E+00 |
| **1252** | F1S3U9 | Uncharacterized protein | PRDX1 | 2.05E+06 | 2.05E+06 | 0.00E+00 | 0.00E+00 | 1.47E+05 | 1.47E+05 | 0.00E+00 | 0.00E+00 |
| **1253** | I3LRH0 | Uncharacterized protein | PRX | 1.06E+07 | 6.32E+06 | 1.95E+06 | 2.36E+06 | 1.46E+05 | 8.66E+04 | 2.67E+04 | 3.23E+04 |
| **1254** | Q29558 | NADP-dependent malic enzyme (Fragment) | ME1 | 4.63E+06 | 0.00E+00 | 3.58E+06 | 1.05E+06 | 1.45E+05 | 0.00E+00 | 1.12E+05 | 3.27E+04 |
| **1255** | V9HWF5 | Peptidyl-prolyl cis-trans isomerase | HEL-S-69p | 2.02E+06 | 1.34E+06 | 6.78E+05 | 0.00E+00 | 1.44E+05 | 9.57E+04 | 4.85E+04 | 0.00E+00 |
| **1256** | K7GLU6 | Uncharacterized protein (Fragment) | ERAP1 | 6.77E+06 | 2.28E+06 | 1.98E+06 | 2.51E+06 | 1.44E+05 | 4.86E+04 | 4.20E+04 | 5.34E+04 |
| **1257** | K7GLP2 | Uncharacterized protein | LOC100737768 | 1.01E+06 | 0.00E+00 | 0.00E+00 | 1.01E+06 | 1.44E+05 | 0.00E+00 | 0.00E+00 | 1.44E+05 |
| **1258** | F1RYI8 | Uncharacterized protein | COL3A1 | 1.18E+07 | 3.05E+06 | 3.64E+06 | 5.07E+06 | 1.43E+05 | 3.72E+04 | 4.44E+04 | 6.18E+04 |
| **1259** | Q4ZG77 | Putative uncharacterized protein PSMD14 (Fragment) | PSMD14 | 5.70E+05 | 5.70E+05 | 0.00E+00 | 0.00E+00 | 1.43E+05 | 1.43E+05 | 0.00E+00 | 0.00E+00 |
| **1260** | F1SIX3 | UTP--glucose-1-phosphate uridylyltransferase | UGP2 | 3.56E+06 | 2.65E+06 | 6.69E+05 | 2.36E+05 | 1.42E+05 | 1.06E+05 | 2.68E+04 | 9.46E+03 |
| **1261** | Q13885 | Tubulin beta-2A chain | TUBB2A | 3.10E+06 | 2.65E+06 | 3.65E+05 | 9.49E+04 | 1.41E+05 | 1.20E+05 | 1.66E+04 | 4.31E+03 |
| **1262** | I3LBK0 | Uncharacterized protein | | 7.01E+05 | 7.01E+05 | 0.00E+00 | 0.00E+00 | 1.40E+05 | 1.40E+05 | 0.00E+00 | 0.00E+00 |
| **1263** | I3L7K2 | Phosphoinositide phospholipase C | PLCD1 | 5.18E+06 | 2.51E+06 | 3.47E+05 | 2.33E+06 | 1.40E+05 | 6.78E+04 | 9.38E+03 | 6.29E+04 |
| **1264** | I3L999 | Uncharacterized protein | RARRES2 | 9.78E+05 | 5.79E+05 | 4.00E+05 | 0.00E+00 | 1.40E+05 | 8.27E+04 | 5.71E+04 | 0.00E+00 |
| **1265** | Q9H3Z4 | DnaJ homolog subfamily C member 5 | DNAJC5 | 9.78E+05 | 0.00E+00 | 9.78E+05 | 0.00E+00 | 1.40E+05 | 0.00E+00 | 1.40E+05 | 0.00E+00 |
| **1266** | F1STC1 | Ribose-5-phosphate isomerase | RPIA | 2.51E+06 | 1.90E+06 | 6.15E+05 | 0.00E+00 | 1.40E+05 | 1.05E+05 | 3.42E+04 | 0.00E+00 |
| **1267** | K7EN45 | Peptidylprolyl isomerase (Fragment) | PIN1 | 6.97E+05 | 6.97E+05 | 0.00E+00 | 0.00E+00 | 1.39E+05 | 1.39E+05 | 0.00E+00 | 0.00E+00 |
| **1268** | F1SGZ8 | Uncharacterized protein | TWF1 | 3.34E+06 | 1.42E+06 | 5.54E+05 | 1.37E+06 | 1.39E+05 | 5.93E+04 | 2.31E+04 | 5.69E+04 |
| **1269** | A6NKE1 | Trafficking protein particle complex subunit 3 | TRAPPC3 | 9.70E+05 | 7.29E+05 | 2.41E+05 | 0.00E+00 | 1.39E+05 | 1.04E+05 | 3.44E+04 | 0.00E+00 |
| **1270** | A0A0S2Z4C3 | Fumarate hydratase isoform 1 (Fragment) | FH | 4.28E+06 | 1.15E+06 | 3.13E+06 | 0.00E+00 | 1.38E+05 | 3.71E+04 | 1.01E+05 | 0.00E+00 |
| **1271** | B4DTA2 | cDNA FLJ60148, highly similar to Homo sapiens heterogeneous nuclear ribonucleoprotein D-like (HNRPDL), transcript variant 2, mRNA | | 1.79E+06 | 1.79E+06 | 0.00E+00 | 0.00E+00 | 1.38E+05 | 1.38E+05 | 0.00E+00 | 0.00E+00 |
| **1272** | K7ES31 | Eukaryotic translation initiation factor 3 subunit K | EIF3K | 9.61E+05 | 9.61E+05 | 0.00E+00 | 0.00E+00 | 1.37E+05 | 1.37E+05 | 0.00E+00 | 0.00E+00 |
| **1273** | K7GSR6 | Uncharacterized protein | NT5E | 4.38E+06 | 9.26E+05 | 1.12E+06 | 2.34E+06 | 1.37E+05 | 2.89E+04 | 3.49E+04 | 7.32E+04 |
| **1274** | Q95N04 | Acetyltransferase component of pyruvate dehydrogenase complex | DLAT | 5.32E+06 | 1.48E+06 | 1.26E+06 | 2.59E+06 | 1.36E+05 | 3.79E+04 | 3.22E+04 | 6.63E+04 |
| **1275** | B4DNN2 | DnaJ (Hsp40) homolog, subfamily B, member 4, isoform CRA | DNAJB4 | 1.63E+06 | 1.33E+06 | 2.98E+05 | 0.00E+00 | 1.36E+05 | 1.11E+05 | 2.49E+04 | 0.00E+00 |
| **1276** | K7GT68 | Uncharacterized protein (Fragment) | ITGA6 | 4.06E+06 | 1.19E+06 | 8.23E+05 | 2.05E+06 | 1.35E+05 | 3.98E+04 | 2.74E+04 | 6.82E+04 |
| **1277** | I3LU31 | Uncharacterized protein | LOC102159373 | 1.49E+06 | 1.49E+06 | 0.00E+00 | 0.00E+00 | 1.35E+05 | 1.35E+05 | 0.00E+00 | 0.00E+00 |
| **1278** | F8VZN8 | Protein phosphatase 1 regulatory subunit 12A (Fragment) | PPP1R12A | 5.13E+06 | 2.31E+06 | 6.78E+05 | 2.14E+06 | 1.35E+05 | 6.09E+04 | 1.78E+04 | 5.62E+04 |
| **1279** | I3LDF4 | Uncharacterized protein | ADA | 3.10E+06 | 0.00E+00 | 2.24E+06 | 8.60E+05 | 1.35E+05 | 0.00E+00 | 9.73E+04 | 3.74E+04 |
| **1280** | F1RRD6 | Uncharacterized protein | PDCD6IP | 6.19E+06 | 1.25E+06 | 1.95E+06 | 2.99E+06 | 1.35E+05 | 2.72E+04 | 4.24E+04 | 6.49E+04 |
| **1281** | B3KX20 | cDNA FLJ44510 fis, clone UTERU3001652, highly similar to Leiomodin-1 | | 4.16E+06 | 1.36E+06 | 1.60E+06 | 1.20E+06 | 1.34E+05 | 4.38E+04 | 5.17E+04 | 3.87E+04 |
| **1282** | F1S1V1 | Uncharacterized protein | SSB | 3.34E+06 | 1.16E+06 | 1.52E+06 | 6.59E+05 | 1.34E+05 | 4.64E+04 | 6.09E+04 | 2.64E+04 |
| **1283** | F1SV18 | Uncharacterized protein | KDELC2 | 4.26E+06 | 1.13E+06 | 1.89E+06 | 1.25E+06 | 1.33E+05 | 3.52E+04 | 5.91E+04 | 3.90E+04 |
| **1284** | V9HWC1 | Epididymis luminal protein 71 | HEL71 | 1.69E+07 | 9.70E+06 | 5.01E+06 | 2.15E+06 | 1.33E+05 | 7.64E+04 | 3.95E+04 | 1.69E+04 |
| **1285** | Q4U1U3 | Cathepsin D | CTSD | 2.65E+06 | 1.57E+06 | 1.08E+06 | 0.00E+00 | 1.33E+05 | 7.84E+04 | 5.42E+04 | 0.00E+00 |
| **1286** | A0A087WUD3 | Oligosaccharyltransferase complex subunit TC | OSTC | 3.92E+05 | 3.92E+05 | 0.00E+00 | 0.00E+00 | 1.31E+05 | 1.31E+05 | 0.00E+00 | 0.00E+00 |
| **1287** | U3KQS7 | U6 snRNA-associated Sm-like protein LSm4 (Fragment) | LSM4 | 9.06E+05 | 0.00E+00 | 0.00E+00 | 9.06E+05 | 1.29E+05 | 0.00E+00 | 0.00E+00 | 1.29E+05 |
| **1288** | F1SU12 | Dolichyl-diphosphooligosaccharide--protein glycosyltransferase 48 kDa subunit | DDOST | 3.36E+06 | 1.31E+06 | 1.52E+06 | 5.31E+05 | 1.29E+05 | 5.05E+04 | 5.83E+04 | 2.04E+04 |
| **1289** | I3LCJ5 | Uncharacterized protein | CYFIP1 | 2.07E+06 | 2.07E+06 | 0.00E+00 | 0.00E+00 | 1.29E+05 | 1.29E+05 | 0.00E+00 | 0.00E+00 |
| **1290** | E9PNN3 | 6-pyruvoyl tetrahydrobiopterin synthase | PTS | 6.44E+05 | 6.44E+05 | 0.00E+00 | 0.00E+00 | 1.29E+05 | 1.29E+05 | 0.00E+00 | 0.00E+00 |
| **1291** | K7GL63 | Uncharacterized protein | CNTN1 | 7.72E+06 | 4.55E+06 | 9.54E+05 | 2.23E+06 | 1.29E+05 | 7.58E+04 | 1.59E+04 | 3.71E+04 |
| **1292** | A0A0B4J1Z1 | Serine/arginine-rich-splicing factor 7 | SRSF7 | 1.41E+06 | 0.00E+00 | 1.41E+06 | 0.00E+00 | 1.28E+05 | 0.00E+00 | 1.28E+05 | 0.00E+00 |
| **1293** | B2R5U1 | cDNA, FLJ92620, highly similar to Homo sapiens staphylococcal nuclease domain containing 1 (SND1),mRNA | | 7.62E+06 | 2.60E+06 | 2.97E+06 | 2.05E+06 | 1.27E+05 | 4.33E+04 | 4.95E+04 | 3.41E+04 |
| **1294** | I3L5X4 | Uncharacterized protein | HBXIP | 3.79E+05 | 0.00E+00 | 3.79E+05 | 0.00E+00 | 1.26E+05 | 0.00E+00 | 1.26E+05 | 0.00E+00 |
| **1295** | P02554 | Tubulin beta chain | TUBB2C | 2.78E+06 | 9.24E+05 | 6.80E+05 | 1.18E+06 | 1.26E+05 | 4.20E+04 | 3.09E+04 | 5.34E+04 |
| **1296** | F1S9K3 | Uncharacterized protein | BPNT1 | 2.86E+06 | 2.06E+06 | 7.98E+05 | 0.00E+00 | 1.24E+05 | 8.97E+04 | 3.47E+04 | 0.00E+00 |
| **1297** | V9HW40 | Epididymis secretory protein Li 25 | HEL-S-25 | 2.60E+06 | 1.42E+06 | 5.78E+05 | 5.98E+05 | 1.24E+05 | 6.78E+04 | 2.75E+04 | 2.85E+04 |
| **1298** | O19063 | Serum amyloid P-component | APCS | 1.61E+06 | 1.61E+06 | 0.00E+00 | 0.00E+00 | 1.24E+05 | 1.24E+05 | 0.00E+00 | 0.00E+00 |
| **1299** | F1RWW4 | Uncharacterized protein | PDLIM5 | 4.56E+06 | 2.11E+06 | 1.26E+06 | 1.19E+06 | 1.23E+05 | 5.71E+04 | 3.40E+04 | 3.21E+04 |
| **1300** | R4GNH2 | F-box only protein 2 (Fragment) | FBXO2 | 7.39E+05 | 4.57E+05 | 2.82E+05 | 0.00E+00 | 1.23E+05 | 7.62E+04 | 4.70E+04 | 0.00E+00 |
| **1301** | A0A024R732 | Syntrophin, beta 2 (Dystrophin-associated protein A1, 59kDa, basic component 2), isoform CRA | SNTB2 | 3.94E+06 | 3.94E+06 | 0.00E+00 | 0.00E+00 | 1.23E+05 | 1.23E+05 | 0.00E+00 | 0.00E+00 |
| **1302** | F1STR6 | Uncharacterized protein | NUDC | 2.81E+06 | 1.20E+06 | 3.18E+05 | 1.30E+06 | 1.22E+05 | 5.20E+04 | 1.38E+04 | 5.65E+04 |
| **1303** | F1RM03 | Uncharacterized protein | SAE1 | 2.80E+06 | 0.00E+00 | 0.00E+00 | 2.80E+06 | 1.22E+05 | 0.00E+00 | 0.00E+00 | 1.22E+05 |
| **1304** | A0A087X0K1 | Calcium-binding protein 39 | CAB39 | 2.90E+06 | 1.75E+06 | 9.57E+05 | 1.91E+05 | 1.21E+05 | 7.29E+04 | 3.99E+04 | 7.96E+03 |
| **1305** | K7GR72 | Anion exchange protein | SLC4A1 | 5.06E+06 | 2.82E+06 | 1.39E+06 | 8.44E+05 | 1.20E+05 | 6.72E+04 | 3.31E+04 | 2.01E+04 |
| **1306** | K9IVV5 | Tryptophan--tRNA ligase, cytoplasmic isoform a | WARS | 3.47E+06 | 2.08E+06 | 1.39E+06 | 0.00E+00 | 1.20E+05 | 7.18E+04 | 4.79E+04 | 0.00E+00 |
| **1307** | P26044 | Radixin | RDX | 4.40E+06 | 2.40E+05 | 2.31E+06 | 1.85E+06 | 1.19E+05 | 6.48E+03 | 6.25E+04 | 5.01E+04 |
| **1308** | I3L3P7 | 40S ribosomal protein S15a | RPS15A | 9.51E+05 | 9.51E+05 | 0.00E+00 | 0.00E+00 | 1.19E+05 | 1.19E+05 | 0.00E+00 | 0.00E+00 |
| **1309** | B4DUR8 | T-complex protein 1 subunit gamma | CCT3 | 3.91E+06 | 0.00E+00 | 1.81E+06 | 2.10E+06 | 1.18E+05 | 0.00E+00 | 5.48E+04 | 6.36E+04 |
| **1310** | B4E2M1 | cDNA FLJ58478, highly similar to Glypican-6 | GPC6 | 4.02E+06 | 1.32E+06 | 5.68E+05 | 2.13E+06 | 1.18E+05 | 3.89E+04 | 1.67E+04 | 6.25E+04 |
| **1311** | X6RHB9 | PITH domain-containing protein 1 (Fragment) | PITHD1 | 5.91E+05 | 5.91E+05 | 0.00E+00 | 0.00E+00 | 1.18E+05 | 1.18E+05 | 0.00E+00 | 0.00E+00 |
| **1312** | H3BV48 | 72 kDa type IV collagenase (Fragment) | MMP2 | 9.42E+05 | 0.00E+00 | 9.42E+05 | 0.00E+00 | 1.18E+05 | 0.00E+00 | 1.18E+05 | 0.00E+00 |
| **1313** | C9JZ20 | Prohibitin (Fragment) | PHB | 1.76E+06 | 8.40E+05 | 0.00E+00 | 9.23E+05 | 1.18E+05 | 5.60E+04 | 0.00E+00 | 6.15E+04 |
| **1314** | F1RH20 | Uncharacterized protein | MYBPC2 | 8.31E+06 | 1.13E+06 | 2.80E+06 | 4.38E+06 | 1.17E+05 | 1.59E+04 | 3.95E+04 | 6.16E+04 |
| **1315** | F1SSF5 | Biliverdin reductase A | BLVRA | 2.34E+06 | 1.96E+06 | 3.83E+05 | 0.00E+00 | 1.17E+05 | 9.78E+04 | 1.92E+04 | 0.00E+00 |
| **1316** | Q96FQ6 | Protein S100-A16 | S100A16 | 6.99E+05 | 1.64E+05 | 2.84E+05 | 2.51E+05 | 1.16E+05 | 2.73E+04 | 4.73E+04 | 4.19E+04 |
| **1317** | F1STS4 | Uncharacterized protein | ME3 | 3.84E+06 | 0.00E+00 | 2.74E+06 | 1.10E+06 | 1.16E+05 | 0.00E+00 | 8.30E+04 | 3.34E+04 |
| **1318** | A0A024RD58 | Glutathione S-transferase A4, isoform CRA | GSTA4 | 1.73E+06 | 1.04E+06 | 0.00E+00 | 6.88E+05 | 1.15E+05 | 6.96E+04 | 0.00E+00 | 4.59E+04 |
| **1319** | F1SFE3 | Uncharacterized protein | FERMT2 | 3.33E+06 | 1.57E+06 | 2.95E+05 | 1.46E+06 | 1.15E+05 | 5.41E+04 | 1.02E+04 | 5.04E+04 |
| **1320** | O00186 | Syntaxin-binding protein 3 | STXBP3 | 4.12E+06 | 4.12E+06 | 0.00E+00 | 0.00E+00 | 1.15E+05 | 1.15E+05 | 0.00E+00 | 0.00E+00 |
| **1321** | Q9N0Y9 | Ubiquitous tropomodulin U-Tmod | TMOD3 | 2.52E+06 | 8.40E+05 | 1.68E+06 | 0.00E+00 | 1.14E+05 | 3.82E+04 | 7.61E+04 | 0.00E+00 |
| **1322** | V9HVZ6 | Epididymis luminal protein 68 | HEL-68 | 2.63E+06 | 2.63E+06 | 0.00E+00 | 0.00E+00 | 1.14E+05 | 1.14E+05 | 0.00E+00 | 0.00E+00 |
| **1323** | H7C531 | 26S proteasome non-ATPase regulatory subunit 6 (Fragment) | PSMD6 | 9.12E+05 | 2.87E+05 | 4.18E+05 | 2.07E+05 | 1.14E+05 | 3.59E+04 | 5.22E+04 | 2.59E+04 |
| **1324** | Q9TUF6 | Long-chain 3-ketoacyl-CoA thiolase | LCTHIO | 2.96E+06 | 2.47E+06 | 4.91E+05 | 0.00E+00 | 1.14E+05 | 9.50E+04 | 1.89E+04 | 0.00E+00 |
| **1325** | F1S557 | Uncharacterized protein | AGL | 9.68E+06 | 3.91E+06 | 4.52E+06 | 1.24E+06 | 1.14E+05 | 4.60E+04 | 5.32E+04 | 1.46E+04 |
| **1326** | F1S3J0 | Uncharacterized protein | ACOT6 | 2.61E+06 | 1.04E+06 | 5.94E+05 | 9.75E+05 | 1.14E+05 | 4.53E+04 | 2.58E+04 | 4.24E+04 |
| **1327** | D0G6X4 | Farnesyl diphosphate synthase | FDPS | 2.72E+06 | 1.67E+06 | 1.05E+06 | 0.00E+00 | 1.13E+05 | 6.95E+04 | 4.39E+04 | 0.00E+00 |
| **1328** | F1SRG2 | NADH dehydrogenase [ubiquinone] 1 alpha subcomplex subunit 6 | LOC100737997 | 1.13E+06 | 0.00E+00 | 1.13E+06 | 0.00E+00 | 1.13E+05 | 0.00E+00 | 1.13E+05 | 0.00E+00 |

**Supplementary Table S1: (continued)**

| | | | | | | | | | | |
|---|---|---|---|---|---|---|---|---|---|---|
| **1329** | H9LB34 | Protein phosphatase 1B magnesium-dependent beta isoform (Fragment) | PPM1B | 1.47E+06 | 0.00E+00 | 7.01E+05 | 7.68E+05 | 1.13E+05 | 0.00E+00 | 5.39E+04 | 5.91E+04 |
| **1330** | I3LKP0 | Uncharacterized protein | SYNM | 9.13E+06 | 3.79E+06 | 2.77E+06 | 2.56E+06 | 1.13E+05 | 4.68E+04 | 3.42E+04 | 3.16E+04 |
| **1331** | A7UIU7 | ATP-citrate synthase | ACL | 7.29E+06 | 4.79E+06 | 1.51E+06 | 9.91E+05 | 1.12E+05 | 7.38E+04 | 2.32E+04 | 1.52E+04 |
| **1332** | G8ENM3 | Ribokinase | RBKS | 1.90E+06 | 6.52E+05 | 2.91E+05 | 9.56E+05 | 1.12E+05 | 3.84E+04 | 1.71E+04 | 5.62E+04 |
| **1333** | Q76N53 | Ribosomal protein L31 (Fragment) | RPL31 | 3.35E+05 | 0.00E+00 | 3.35E+05 | 0.00E+00 | 1.12E+05 | 0.00E+00 | 1.12E+05 | 0.00E+00 |
| **1334** | K9IVL7 | Prolow-density lipoprotein receptor-related protein 1 | LRP1 | 2.84E+07 | 9.51E+06 | 8.16E+06 | 1.07E+07 | 1.11E+05 | 3.71E+04 | 3.19E+04 | 4.20E+04 |
| **1335** | P63267 | Actin, gamma-enteric smooth muscle | ACTG2 | 2.55E+06 | 2.55E+06 | 0.00E+00 | 0.00E+00 | 1.11E+05 | 1.11E+05 | 0.00E+00 | 0.00E+00 |
| **1336** | V9HW72 | Epididymis secretory sperm binding protein Li 94n | HEL-S-94n | 4.64E+06 | 5.53E+05 | 8.75E+05 | 3.21E+06 | 1.10E+05 | 1.32E+04 | 2.08E+04 | 7.65E+04 |
| **1337** | K7GRN9 | Uncharacterized protein | FAM129A | 3.74E+06 | 2.28E+06 | 9.01E+05 | 5.61E+05 | 1.10E+05 | 6.71E+04 | 2.65E+04 | 1.65E+04 |
| **1338** | F1RR45 | Uncharacterized protein | CRAT | 3.85E+06 | 9.69E+05 | 1.10E+06 | 1.78E+06 | 1.10E+05 | 2.77E+04 | 3.15E+04 | 5.09E+04 |
| **1339** | F1S605 | Uncharacterized protein | GSTM3 | 2.17E+06 | 1.27E+06 | 2.70E+05 | 6.34E+05 | 1.09E+05 | 6.35E+04 | 1.35E+04 | 3.17E+04 |
| **1340** | D3DQ64 | Adenylate kinase 4, mitochondrial | AK4 | 1.30E+06 | 6.39E+05 | 0.00E+00 | 6.64E+05 | 1.09E+05 | 5.32E+04 | 0.00E+00 | 5.54E+04 |
| **1341** | B4DTY8 | cDNA FLJ61587, highly similar to Integrin alpha-1 (Fragment) | ITGA1 | 6.71E+06 | 2.18E+06 | 1.78E+06 | 2.75E+06 | 1.08E+05 | 3.52E+04 | 2.87E+04 | 4.44E+04 |
| **1342** | A0A024RAB6 | Heparan sulfate proteoglycan 2 (Perlecan), isoform CRA | HSPG2 | 2.03E+07 | 1.31E+07 | 5.27E+06 | 1.87E+06 | 1.08E+05 | 6.98E+04 | 2.80E+04 | 9.97E+03 |
| **1343** | F1SI50 | Uncharacterized protein | NDUFB3 | 5.36E+05 | 0.00E+00 | 2.85E+05 | 2.51E+05 | 1.07E+05 | 0.00E+00 | 5.70E+04 | 5.02E+04 |
| **1344** | F1RY03 | Uncharacterized protein | FHL5 | 2.35E+06 | 2.35E+06 | 0.00E+00 | 0.00E+00 | 1.07E+05 | 1.07E+05 | 0.00E+00 | 0.00E+00 |
| **1345** | E9PKR8 | Sulfotransferase (Fragment) | SULT1A2 | 1.69E+06 | 1.16E+06 | 5.35E+05 | 0.00E+00 | 1.06E+05 | 7.23E+04 | 3.35E+04 | 0.00E+00 |
| **1346** | F1RK01 | Uncharacterized protein | CPB2 | 2.54E+06 | 0.00E+00 | 1.31E+06 | 1.23E+06 | 1.06E+05 | 0.00E+00 | 5.46E+04 | 5.11E+04 |
| **1347** | B2R5U3 | EH-domain containing 1, isoform CRA | EHD1 | 3.48E+06 | 3.29E+05 | 1.89E+06 | 1.26E+06 | 1.06E+05 | 9.97E+03 | 5.74E+04 | 3.81E+04 |
| **1348** | I3L812 | Uncharacterized protein | TPP1 | 2.63E+06 | 8.60E+05 | 8.16E+05 | 9.58E+05 | 1.05E+05 | 3.44E+04 | 3.26E+04 | 3.83E+04 |
| **1349** | I3LTR9 | Uncharacterized protein | | 1.05E+06 | 1.05E+06 | 0.00E+00 | 0.00E+00 | 1.05E+05 | 1.05E+05 | 0.00E+00 | 0.00E+00 |
| **1350** | F1SU59 | Annexin | ANXA7 | 2.73E+06 | 1.49E+06 | 1.08E+06 | 1.60E+05 | 1.05E+05 | 5.74E+04 | 4.14E+04 | 6.15E+03 |
| **1351** | P11216 | Glycogen phosphorylase, brain form | PYGB | 5.77E+06 | 4.27E+06 | 8.67E+05 | 6.37E+05 | 1.05E+05 | 7.76E+04 | 1.58E+04 | 1.16E+04 |
| **1352** | F1SK65 | Uncharacterized protein | | 1.68E+06 | 5.61E+05 | 0.00E+00 | 1.12E+06 | 1.05E+05 | 3.51E+04 | 0.00E+00 | 6.97E+04 |
| **1353** | B4DNA3 | Adenylyl cyclase-associated protein | | 3.25E+06 | 3.17E+05 | 6.91E+05 | 2.24E+06 | 1.05E+05 | 1.02E+04 | 2.23E+04 | 7.22E+04 |
| **1354** | F1SLI4 | Uncharacterized protein | DHX30 | 8.90E+06 | 5.39E+06 | 0.00E+00 | 3.51E+06 | 1.05E+05 | 6.34E+04 | 0.00E+00 | 4.13E+04 |
| **1355** | F1SUF2 | Uncharacterized protein | HK1 | 4.59E+06 | 2.15E+06 | 1.40E+06 | 1.04E+06 | 1.04E+05 | 4.88E+04 | 3.19E+04 | 2.36E+04 |
| **1356** | F1SU06 | Uncharacterized protein | HP1BP3 | 3.02E+06 | 9.20E+05 | 1.24E+06 | 8.63E+05 | 1.04E+05 | 3.17E+04 | 4.27E+04 | 2.98E+04 |
| **1357** | P56537 | Eukaryotic translation initiation factor 6 | EIF6 | 1.04E+06 | 1.04E+06 | 0.00E+00 | 0.00E+00 | 1.04E+05 | 1.04E+05 | 0.00E+00 | 0.00E+00 |
| **1358** | Q59FS8 | Eukaryotic translation initiation factor 3, subunit 9 eta, 116kDa isoform b variant (Fragment) | EIF3 | 1.13E+06 | 0.00E+00 | 0.00E+00 | 1.13E+06 | 1.03E+05 | 0.00E+00 | 0.00E+00 | 1.03E+05 |
| **1359** | I3LFZ2 | Uncharacterized protein | RPA3 | 8.11E+05 | 0.00E+00 | 6.15E+05 | 1.96E+05 | 1.01E+05 | 0.00E+00 | 7.69E+04 | 2.45E+04 |
| **1360** | Q6PIX2 | SFPQ protein (Fragment) | SFPQ | 2.63E+06 | 1.48E+06 | 5.06E+05 | 6.47E+05 | 1.01E+05 | 5.69E+04 | 1.95E+04 | 2.49E+04 |
| **1361** | B4DEX8 | Methionine adenosyltransferase II, alpha, isoform CRA | MAT2A | 1.81E+06 | 1.81E+06 | 0.00E+00 | 0.00E+00 | 1.01E+05 | 1.01E+05 | 0.00E+00 | 0.00E+00 |
| **1362** | F1SQ05 | Uncharacterized protein | PHEX | 4.53E+06 | 1.12E+06 | 1.23E+06 | 2.17E+06 | 1.01E+05 | 2.49E+04 | 2.73E+04 | 4.83E+04 |
| **1363** | F2Z5I8 | Uncharacterized protein | RAB8A | 1.70E+06 | 5.67E+05 | 3.89E+05 | 7.43E+05 | 9.99E+04 | 3.34E+04 | 2.29E+04 | 4.37E+04 |
| **1364** | Q8IWR8 | Ribosomal protein L19 (Fragment) | RPL19 | 7.96E+05 | 0.00E+00 | 7.96E+05 | 0.00E+00 | 9.95E+04 | 0.00E+00 | 9.95E+04 | 0.00E+00 |
| **1365** | Q2TJA5 | Aldo-keto reductase | akr | 2.38E+06 | 1.63E+06 | 7.50E+05 | 0.00E+00 | 9.93E+04 | 6.80E+04 | 3.13E+04 | 0.00E+00 |
| **1366** | M3TYI1 | Phosphoglucomutase 2 | PGM2 | 3.92E+06 | 7.60E+05 | 6.93E+05 | 2.47E+06 | 9.80E+04 | 1.90E+04 | 1.73E+04 | 6.17E+04 |
| **1367** | I3LNJ2 | Uncharacterized protein | FMNL2 | 6.36E+06 | 0.00E+00 | 0.00E+00 | 6.36E+06 | 9.79E+04 | 0.00E+00 | 0.00E+00 | 9.79E+04 |
| **1368** | A5A759 | Keratin 2A | KRT2A | 4.11E+06 | 0.00E+00 | 0.00E+00 | 4.11E+06 | 9.78E+04 | 0.00E+00 | 0.00E+00 | 9.78E+04 |
| **1369** | I3LRH5 | Uncharacterized protein | EPDR1 | 5.83E+05 | 5.83E+05 | 0.00E+00 | 0.00E+00 | 9.71E+04 | 9.71E+04 | 0.00E+00 | 0.00E+00 |
| **1370** | F1SFF8 | Alpha-1,4 glucan phosphorylase | PYGL | 5.53E+06 | 3.92E+06 | 1.02E+06 | 5.86E+05 | 9.70E+04 | 6.89E+04 | 1.79E+04 | 1.03E+04 |
| **1371** | F1RXD4 | Uncharacterized protein | RMDN1 | 2.13E+06 | 2.13E+06 | 0.00E+00 | 0.00E+00 | 9.69E+04 | 9.69E+04 | 0.00E+00 | 0.00E+00 |
| **1372** | Q6NVY0 | Calcyclin binding protein | CACYBP | 1.55E+06 | 5.34E+05 | 1.02E+06 | 0.00E+00 | 9.69E+04 | 3.34E+04 | 6.35E+04 | 0.00E+00 |
| **1373** | F1SSI2 | Uncharacterized protein | DHRS7 | 2.03E+06 | 1.53E+06 | 5.00E+05 | 0.00E+00 | 9.68E+04 | 7.30E+04 | 2.38E+04 | 0.00E+00 |
| **1374** | P18650 | Apolipoprotein E | APOE | 2.42E+06 | 1.02E+06 | 4.85E+05 | 9.18E+05 | 9.68E+04 | 4.07E+04 | 1.94E+04 | 3.67E+04 |
| **1375** | Q53FX5 | Lin-7 homolog C variant (Fragment) | LIN7C | 1.55E+06 | 8.10E+05 | 0.00E+00 | 7.38E+05 | 9.68E+04 | 5.06E+04 | 0.00E+00 | 4.62E+04 |
| **1376** | A0ZYT4 | Elongation initiation factor 4E (Fragment) | EIF4E | 1.06E+06 | 1.06E+06 | 0.00E+00 | 0.00E+00 | 9.66E+04 | 9.66E+04 | 0.00E+00 | 0.00E+00 |
| **1377** | F1SSN5 | Uncharacterized protein | KTN1 | 8.84E+06 | 2.88E+06 | 2.47E+06 | 3.49E+06 | 9.61E+04 | 3.13E+04 | 2.68E+04 | 3.79E+04 |
| **1378** | V9GYF8 | Rab GDP dissociation inhibitor beta (Fragment) | GDI2 | 9.59E+05 | 9.59E+05 | 0.00E+00 | 0.00E+00 | 9.59E+04 | 9.59E+04 | 0.00E+00 | 0.00E+00 |
| **1379** | F1RIU3 | Uncharacterized protein | LOC102163415 | 2.68E+06 | 7.61E+05 | 7.79E+05 | 1.14E+06 | 9.58E+04 | 2.72E+04 | 2.78E+04 | 4.08E+04 |
| **1380** | A0A0F7G8J1 | Plasminogen | PLG | 4.73E+06 | 1.06E+06 | 8.44E+05 | 2.83E+06 | 9.47E+04 | 2.12E+04 | 1.69E+04 | 5.66E+04 |
| **1381** | A8K8U3 | Calcium-transporting ATPase | ATP2B4 | 5.95E+06 | 4.66E+06 | 1.06E+06 | 2.24E+05 | 9.44E+04 | 7.40E+04 | 1.68E+04 | 3.56E+03 |
| **1382** | V9HVZ4 | Glyceraldehyde-3-phosphate dehydrogenase | HEL-S-162eP | 1.79E+06 | 1.79E+06 | 0.00E+00 | 0.00E+00 | 9.41E+04 | 9.41E+04 | 0.00E+00 | 0.00E+00 |
| **1383** | I3LPN0 | Tryptase | MCT7 | 1.41E+06 | 4.53E+05 | 4.64E+05 | 4.93E+05 | 9.40E+04 | 3.02E+04 | 3.09E+04 | 3.29E+04 |
| **1384** | I3LDP5 | Uncharacterized protein | SUSD2 | 3.19E+06 | 1.51E+06 | 1.68E+06 | 0.00E+00 | 9.37E+04 | 4.43E+04 | 4.94E+04 | 0.00E+00 |
| **1385** | B8YQC1 | Galectin (Fragment) | LGALSL | 8.34E+05 | 8.34E+05 | 0.00E+00 | 0.00E+00 | 9.27E+04 | 9.27E+04 | 0.00E+00 | 0.00E+00 |
| **1386** | Q8HXW0 | L-gulonolactone oxidase | GULO | 2.04E+06 | 0.00E+00 | 1.10E+06 | 9.40E+05 | 9.25E+04 | 0.00E+00 | 4.98E+04 | 4.27E+04 |
| **1387** | Q9BWJ5 | Splicing factor 3B subunit 5 | SF3B5 | 5.52E+05 | 0.00E+00 | 1.93E+05 | 3.59E+05 | 9.19E+04 | 0.00E+00 | 3.21E+04 | 5.98E+04 |
| **1388** | G3V5E4 | Glucosamine 6-phosphate N-acetyltransferase | GNPNAT1 | 6.38E+05 | 3.65E+05 | 0.00E+00 | 2.73E+05 | 9.12E+04 | 5.22E+04 | 0.00E+00 | 3.90E+04 |
| **1389** | V9HWJ7 | Epididymis secretory protein Li 37 | HEL-S-37 | 3.72E+06 | 1.96E+06 | 1.10E+06 | 6.61E+05 | 9.07E+04 | 4.79E+04 | 2.67E+04 | 1.61E+04 |
| **1390** | L7X0X2 | Fas-associated death domain protein (Fragment) | FADD | 3.61E+05 | 3.61E+05 | 0.00E+00 | 0.00E+00 | 9.02E+04 | 9.02E+04 | 0.00E+00 | 0.00E+00 |
| **1391** | F1S4D7 | Uncharacterized protein | GBP1 | 2.97E+06 | 2.49E+06 | 4.86E+05 | 0.00E+00 | 9.01E+04 | 7.54E+04 | 1.47E+04 | 0.00E+00 |
| **1392** | Q8IXW6 | Dihydropyrimidinase-like 3 | DPYSL3 | 2.97E+06 | 0.00E+00 | 0.00E+00 | 2.97E+06 | 9.00E+04 | 0.00E+00 | 0.00E+00 | 9.00E+04 |
| **1393** | F1RWX8 | Uncharacterized protein | UBA1 | 5.29E+06 | 5.32E+05 | 3.51E+06 | 1.25E+06 | 8.97E+04 | 9.02E+03 | 5.94E+04 | 2.12E+04 |
| **1394** | A5GFX7 | Cathepsin Z | CTSZ | 1.61E+06 | 1.61E+06 | 0.00E+00 | 0.00E+00 | 8.94E+04 | 8.94E+04 | 0.00E+00 | 0.00E+00 |
| **1395** | K9J6K1 | Tapasin | TAPBP | 1.07E+06 | 1.07E+06 | 0.00E+00 | 0.00E+00 | 8.94E+04 | 8.94E+04 | 0.00E+00 | 0.00E+00 |
| **1396** | I3L0P9 | Centrobin (Fragment) | CNTROB | 1.16E+06 | 0.00E+00 | 0.00E+00 | 1.16E+06 | 8.92E+04 | 0.00E+00 | 0.00E+00 | 8.92E+04 |
| **1397** | G3V325 | Protein ATP5J2-PTCD1 | ATP5J2-PTCD1 | 3.83E+06 | 2.38E+06 | 1.46E+06 | 0.00E+00 | 8.91E+04 | 5.52E+04 | 3.39E+04 | 0.00E+00 |
| **1398** | Q59FM5 | Guanine nucleotide-binding protein G, alpha subunit variant (Fragment) | GNA11 | 2.04E+06 | 1.26E+06 | 0.00E+00 | 7.86E+05 | 8.87E+04 | 5.46E+04 | 0.00E+00 | 3.42E+04 |
| **1399** | E2QRI1 | Prolyl 3-hydroxylase 1 (Fragment) | P3H1 | 7.09E+05 | 7.09E+05 | 0.00E+00 | 0.00E+00 | 8.87E+04 | 8.87E+04 | 0.00E+00 | 0.00E+00 |
| **1400** | F1RMN7 | Hemopexin | HPX | 2.31E+06 | 0.00E+00 | 1.04E+06 | 1.26E+06 | 8.87E+04 | 0.00E+00 | 4.01E+04 | 4.85E+04 |
| **1401** | I3LN38 | Uncharacterized protein | | 2.92E+06 | 0.00E+00 | 1.19E+06 | 1.73E+06 | 8.86E+04 | 0.00E+00 | 3.62E+04 | 5.25E+04 |
| **1402** | F1S8M9 | Uncharacterized protein | LRPAP1 | 2.12E+06 | 1.02E+06 | 6.80E+05 | 4.19E+05 | 8.84E+04 | 4.27E+04 | 2.83E+04 | 1.75E+04 |
| **1403** | F1RGY5 | Uncharacterized protein | | 1.59E+06 | 1.23E+06 | 0.00E+00 | 3.61E+05 | 8.83E+04 | 6.82E+04 | 0.00E+00 | 2.01E+04 |
| **1404** | F1RLE5 | Uncharacterized protein | TMED7 | 1.23E+06 | 3.44E+05 | 0.00E+00 | 8.90E+05 | 8.81E+04 | 2.45E+04 | 0.00E+00 | 6.36E+04 |
| **1405** | Q5T6H7 | Xaa-Pro aminopeptidase 1 | XPNPEP1 | 3.08E+06 | 9.32E+05 | 1.01E+06 | 1.14E+06 | 8.80E+04 | 2.66E+04 | 2.89E+04 | 3.25E+04 |
| **1406** | M0R0Y3 | RuvB-like helicase | RUVBL2 | 1.67E+06 | 1.05E+06 | 6.18E+05 | 0.00E+00 | 8.76E+04 | 5.51E+04 | 3.25E+04 | 0.00E+00 |
| **1407** | Q53QT6 | Putative uncharacterized protein FRZB (Fragment) | FRZB | 6.99E+05 | 6.99E+05 | 0.00E+00 | 0.00E+00 | 8.74E+04 | 8.74E+04 | 0.00E+00 | 0.00E+00 |
| **1408** | F1S9V9 | Uncharacterized protein | TMEM38A | 8.65E+05 | 8.65E+05 | 0.00E+00 | 0.00E+00 | 8.65E+04 | 8.65E+04 | 0.00E+00 | 0.00E+00 |
| **1409** | F5H7C4 | Methionine-R-sulfoxide reductase B3 (Fragment) | MSRB3 | 7.76E+05 | 5.29E+05 | 0.00E+00 | 2.47E+05 | 8.62E+04 | 5.88E+04 | 0.00E+00 | 2.74E+04 |
| **1410** | A0A142CHG9 | GO2-q chimeric G-protein | | 1.89E+06 | 1.89E+06 | 0.00E+00 | 0.00E+00 | 8.60E+04 | 8.60E+04 | 0.00E+00 | 0.00E+00 |
| **1411** | Q6DKJ4 | Nucleoredoxin | NXN | 1.97E+06 | 0.00E+00 | 1.45E+06 | 5.24E+05 | 8.58E+04 | 0.00E+00 | 6.30E+04 | 2.28E+04 |
| **1412** | F1S6C3 | Uncharacterized protein | ACAD8 | 1.03E+06 | 6.66E+05 | 3.64E+05 | 0.00E+00 | 8.58E+04 | 5.55E+04 | 3.03E+04 | 0.00E+00 |
| **1413** | O77564 | Glutamate carboxypeptidase 2 | FOLH1 | 3.94E+06 | 1.14E+06 | 1.47E+06 | 1.32E+06 | 8.56E+04 | 2.48E+04 | 3.20E+04 | 2.88E+04 |
| **1414** | P81605 | Dermcidin | DCD | 5.96E+05 | 3.83E+05 | 0.00E+00 | 2.12E+05 | 8.51E+04 | 5.48E+04 | 0.00E+00 | 3.03E+04 |
| **1415** | O97507 | Coagulation factor XII | F12 | 2.12E+06 | 0.00E+00 | 8.74E+05 | 1.25E+06 | 8.48E+04 | 0.00E+00 | 3.50E+04 | 4.98E+04 |
| **1416** | I3LTB8 | Uncharacterized protein | | 2.12E+06 | 2.12E+06 | 0.00E+00 | 0.00E+00 | 8.47E+04 | 8.47E+04 | 0.00E+00 | 0.00E+00 |
| **1417** | F1SQX8 | Uncharacterized protein | LOC100737308 | 1.01E+06 | 3.26E+05 | 0.00E+00 | 6.80E+05 | 8.39E+04 | 2.72E+04 | 0.00E+00 | 5.67E+04 |
| **1418** | E9PGT1 | Translin | TSN | 1.24E+06 | 1.24E+06 | 0.00E+00 | 0.00E+00 | 8.29E+04 | 8.29E+04 | 0.00E+00 | 0.00E+00 |
| **1419** | H0YM59 | Zinc finger protein 770 (Fragment) | ZNF770 | 9.10E+05 | 0.00E+00 | 3.22E+05 | 5.89E+05 | 8.28E+04 | 0.00E+00 | 2.93E+04 | 5.35E+04 |
| **1420** | K7GN52 | Uncharacterized protein | TRIM72 | 1.82E+06 | 9.37E+05 | 8.80E+05 | 0.00E+00 | 8.26E+04 | 4.26E+04 | 4.00E+04 | 0.00E+00 |
| **1421** | U3R8H0 | Aminopeptidase N | APN | 3.71E+06 | 3.10E+06 | 2.20E+05 | 3.90E+05 | 8.25E+04 | 6.90E+04 | 4.90E+03 | 8.66E+03 |
| **1422** | B7Z8X5 | cDNA FLJ61541, highly similar to Homo sapiens PDZ and LIM domain 5 (PDLIM5), transcript variant 2, mRNA | PDLIM5 | 2.39E+06 | 0.00E+00 | 1.12E+06 | 1.27E+06 | 8.24E+04 | 0.00E+00 | 3.87E+04 | 4.38E+04 |
| **1423** | Q53GB2 | SEC13-like 1 isoform b variant (Fragment) | SEC13 | 1.07E+06 | 1.07E+06 | 0.00E+00 | 0.00E+00 | 8.24E+04 | 8.24E+04 | 0.00E+00 | 0.00E+00 |
| **1424** | I3LEB7 | Uncharacterized protein | PODN | 3.79E+06 | 1.66E+06 | 8.13E+05 | 1.31E+06 | 8.24E+04 | 3.62E+04 | 1.77E+04 | 2.85E+04 |
| **1425** | I3LLD8 | Uncharacterized protein | LOC100626701 | 1.65E+06 | 0.00E+00 | 5.89E+05 | 1.06E+06 | 8.24E+04 | 0.00E+00 | 2.94E+04 | 5.29E+04 |
| **1426** | I3L742 | RuvB-like helicase | LOC100518219 | 1.97E+06 | 1.07E+06 | 9.02E+05 | 0.00E+00 | 8.22E+04 | 4.46E+04 | 3.76E+04 | 0.00E+00 |
| **1427** | I1SRC5 | UBE2L3/KRAS fusion protein | | 1.40E+06 | 1.40E+06 | 0.00E+00 | 0.00E+00 | 8.21E+04 | 8.21E+04 | 0.00E+00 | 0.00E+00 |
| **1428** | I3LBY0 | Uncharacterized protein | ARCN1 | 2.55E+06 | 1.89E+06 | 3.02E+05 | 3.59E+05 | 8.21E+04 | 6.08E+04 | 9.73E+03 | 1.16E+04 |
| **1429** | Q5VSQ6 | Procollagen-proline, 2-oxoglutarate 4-dioxygenase (Proline 4-hydroxylase), alpha polypeptide I variant (Fragment) | P4HA1 | 2.62E+06 | 1.61E+06 | 1.01E+06 | 0.00E+00 | 8.20E+04 | 5.03E+04 | 3.17E+04 | 0.00E+00 |
| **1430** | Q53FN1 | Cysteine-rich protein 2 variant (Fragment) | CRIP2 | 1.06E+06 | 1.06E+06 | 0.00E+00 | 0.00E+00 | 8.16E+04 | 8.16E+04 | 0.00E+00 | 0.00E+00 |
| **1431** | Q8WNV7 | Dehydrogenase/reductase SDR family member 4 | DHRS4 | 1.22E+06 | 1.22E+06 | 0.00E+00 | 0.00E+00 | 8.15E+04 | 8.15E+04 | 0.00E+00 | 0.00E+00 |
| **1432** | P79388 | Folate binding protein | FOLR2 | 1.06E+06 | 1.06E+06 | 0.00E+00 | 0.00E+00 | 8.15E+04 | 8.15E+04 | 0.00E+00 | 0.00E+00 |
| **1433** | F1S764 | Uncharacterized protein | CPT2 | 3.73E+06 | 1.64E+06 | 8.26E+05 | 1.27E+06 | 8.11E+04 | 3.56E+04 | 1.80E+04 | 2.75E+04 |
| **1434** | B7Z2F4 | T-complex protein 1 subunit delta | | 2.01E+06 | 0.00E+00 | 1.79E+06 | 2.28E+05 | 8.06E+04 | 0.00E+00 | 7.14E+04 | 9.11E+03 |
| **1435** | C9J931 | GTP-binding protein Rheb | RHEB | 6.39E+05 | 0.00E+00 | 0.00E+00 | 6.39E+05 | 7.99E+04 | 0.00E+00 | 0.00E+00 | 7.99E+04 |
| **1436** | H0Y7F4 | Guanine nucleotide-binding protein G(s) subunit alpha isoforms short (Fragment) | GNAS | 8.79E+05 | 8.79E+05 | 0.00E+00 | 0.00E+00 | 7.99E+04 | 7.99E+04 | 0.00E+00 | 0.00E+00 |
| **1437** | F1SRU2 | Uncharacterized protein | ZYX | 6.36E+05 | 6.36E+05 | 0.00E+00 | 0.00E+00 | 7.94E+04 | 7.94E+04 | 0.00E+00 | 0.00E+00 |
| **1438** | K7EIK7 | Echinoderm microtubule-associated protein-like 2 | EML2 | 2.86E+06 | 0.00E+00 | 2.86E+06 | 0.00E+00 | 7.94E+04 | 0.00E+00 | 7.94E+04 | 0.00E+00 |
| **1439** | H3BT82 | Syntaxin-1B (Fragment) | STX1B | 1.19E+06 | 1.19E+06 | 0.00E+00 | 0.00E+00 | 7.92E+04 | 7.92E+04 | 0.00E+00 | 0.00E+00 |
| **1440** | E7EQV9 | Ribosomal protein L15 (Fragment) | RPL15 | 9.48E+05 | 0.00E+00 | 9.48E+05 | 0.00E+00 | 7.90E+04 | 0.00E+00 | 7.90E+04 | 0.00E+00 |
| **1441** | F1RHA7 | Transforming growth factor-beta-induced protein ig-h3 | TGFBI | 2.74E+06 | 0.00E+00 | 7.94E+05 | 1.95E+06 | 7.84E+04 | 0.00E+00 | 2.27E+04 | 5.57E+04 |
| **1442** | F1S0J2 | Uncharacterized protein | C4BPA | 2.74E+06 | 1.61E+06 | 0.00E+00 | 1.13E+06 | 7.83E+04 | 4.61E+04 | 0.00E+00 | 3.22E+04 |
| **1443** | H3BPF6 | Prefoldin subunit 5 (Fragment) | PFDN5 | 8.60E+05 | 0.00E+00 | 4.58E+05 | 4.03E+05 | 7.82E+04 | 0.00E+00 | 4.16E+04 | 3.66E+04 |
| **1444** | Q4FAT7 | Beta-glucuronidase | GUSB | 2.49E+06 | 3.32E+05 | 7.17E+05 | 1.44E+06 | 7.79E+04 | 1.04E+04 | 2.24E+04 | 4.51E+04 |
| **1445** | I3LUU1 | Uncharacterized protein | PCBD2 | 6.22E+05 | 3.13E+05 | 0.00E+00 | 3.09E+05 | 7.77E+04 | 3.91E+04 | 0.00E+00 | 3.86E+04 |
| **1446** | O14983 | Sarcoplasmic/endoplasmic reticulum calcium ATPase 1 | ATP2A1 | 4.03E+06 | 1.90E+06 | 1.02E+06 | 1.10E+06 | 7.75E+04 | 3.66E+04 | 1.97E+04 | 2.11E+04 |
| **1447** | B4DUT7 | cDNA FLJ57604, highly similar to GMP synthase (glutamine-hydrolyzing) (EC 6.3.5.2) | | 3.59E+06 | 2.15E+05 | 5.09E+05 | 2.86E+06 | 7.64E+04 | 4.58E+03 | 1.08E+04 | 6.10E+04 |
| **1448** | F1RFA5 | Uncharacterized protein | PGP | 1.45E+06 | 1.45E+06 | 0.00E+00 | 0.00E+00 | 7.63E+04 | 7.63E+04 | 0.00E+00 | 0.00E+00 |
| **1449** | K7GR86 | Uncharacterized protein (Fragment) | NCAM1 | 3.17E+06 | 1.50E+06 | 8.16E+05 | 8.54E+05 | 7.55E+04 | 3.57E+04 | 1.94E+04 | 2.03E+04 |
| **1450** | E9PKD5 | 26S protease regulatory subunit 6A (Fragment) | PSMC3 | 1.27E+06 | 1.27E+06 | 0.00E+00 | 0.00E+00 | 7.44E+04 | 7.44E+04 | 0.00E+00 | 0.00E+00 |
| **1451** | P19205 | Acylamino-acid-releasing enzyme | APEH | 2.65E+06 | 2.65E+06 | 0.00E+00 | 0.00E+00 | 7.37E+04 | 7.37E+04 | 0.00E+00 | 0.00E+00 |
| **1452** | F1S365 | Uroporphyrinogen decarboxylase | UROD | 1.09E+06 | 9.31E+05 | 0.00E+00 | 1.60E+05 | 7.27E+04 | 6.20E+04 | 0.00E+00 | 1.07E+04 |
| **1453** | F1RN71 | Uncharacterized protein | BLMH | 7.96E+05 | 0.00E+00 | 7.96E+05 | 0.00E+00 | 7.23E+04 | 0.00E+00 | 7.23E+04 | 0.00E+00 |
| **1454** | Q28956 | 17beta-estradiol dehydrogenase | HSD17B4 | 3.25E+06 | 1.93E+06 | 5.53E+05 | 7.73E+05 | 7.23E+04 | 4.28E+04 | 1.23E+04 | 1.72E+04 |
| **1455** | A0A140VK56 | Testicular secretory protein Li 56 | | 1.72E+06 | 0.00E+00 | 8.73E+05 | 8.51E+05 | 7.18E+04 | 0.00E+00 | 3.64E+04 | 3.54E+04 |
| **1456** | F1SBH3 | Uncharacterized protein | RBBP9 | 1.00E+06 | 1.00E+06 | 0.00E+00 | 0.00E+00 | 7.18E+04 | 7.18E+04 | 0.00E+00 | 0.00E+00 |
| **1457** | O19062 | C-reactive protein | CRP | 7.89E+05 | 4.19E+05 | 3.70E+05 | 0.00E+00 | 7.17E+04 | 3.81E+04 | 3.36E+04 | 0.00E+00 |
| **1458** | F1SBS4 | Uncharacterized protein | | 5.42E+06 | 2.27E+06 | 1.99E+06 | 1.16E+06 | 7.13E+04 | 2.98E+04 | 2.62E+04 | 1.53E+04 |
| **1459** | Q496C9 | D-tyrosyl-tRNA(Tyr) deacylase | DTD1 | 5.70E+05 | 5.70E+05 | 0.00E+00 | 0.00E+00 | 7.13E+04 | 7.13E+04 | 0.00E+00 | 0.00E+00 |
| **1460** | P54289 | Voltage-dependent calcium channel subunit alpha-2/delta-1 | CACNA2D1 | 4.19E+06 | 4.02E+06 | 1.62E+05 | 0.00E+00 | 7.09E+04 | 6.82E+04 | 2.74E+03 | 0.00E+00 |
| **1461** | A0A140VKC0 | Testis tissue sperm-binding protein Li 37a | | 2.41E+06 | 3.53E+05 | 1.64E+06 | 4.16E+05 | 7.09E+04 | 1.04E+04 | 4.82E+04 | 1.22E+04 |
| **1462** | I3LV69 | Pyridoxal kinase | PDXK | 1.27E+06 | 1.27E+06 | 0.00E+00 | 0.00E+00 | 7.08E+04 | 7.08E+04 | 0.00E+00 | 0.00E+00 |
| **1463** | A0A0S2Z4P7 | CCHC-type zinc finger nucleic acid binding protein isoform 2 | CNBP | 4.95E+05 | 4.95E+05 | 0.00E+00 | 0.00E+00 | 7.08E+04 | 7.08E+04 | 0.00E+00 | 0.00E+00 |
| **1464** | Q75NH0 | Troponin T fast skeletal muscle type | tnnt3 | 9.88E+05 | 2.75E+05 | 0.00E+00 | 7.13E+05 | 7.06E+04 | 1.97E+04 | 0.00E+00 | 5.09E+04 |
| **1465** | E7EBY5 | MACRO domain containing protein 1 | MACROD1 | 1.34E+06 | 9.27E+05 | 4.14E+05 | 0.00E+00 | 7.06E+04 | 4.88E+04 | 2.18E+04 | 0.00E+00 |
| **1466** | B3KQI1 | cDNA FLJ90452 fis, clone NT2RP3001475, highly similar to Splicing factor 3B subunit 3 | | 2.88E+06 | 1.44E+06 | 3.65E+05 | 1.07E+06 | 7.02E+04 | 3.52E+04 | 8.90E+03 | 2.62E+04 |
| **1467** | F1SA96 | Uncharacterized protein | IAH1 | 1.19E+06 | 7.14E+05 | 4.73E+05 | 0.00E+00 | 6.98E+04 | 4.20E+04 | 2.78E+04 | 0.00E+00 |
| **1468** | P11055 | Myosin-3 | MYH3 | 8.85E+06 | 6.85E+06 | 1.79E+06 | 2.11E+05 | 6.97E+04 | 5.39E+04 | 1.41E+04 | 1.66E+03 |
| **1469** | F1RI15 | Uncharacterized protein | HSPA4 | 3.97E+06 | 1.55E+06 | 4.98E+05 | 1.92E+06 | 6.96E+04 | 2.72E+04 | 8.73E+03 | 3.37E+04 |
| **1470** | K7GNQ2 | Eosinophil peroxidase | EPX | 3.46E+06 | 2.95E+06 | 5.07E+05 | 0.00E+00 | 6.92E+04 | 5.91E+04 | 1.01E+04 | 0.00E+00 |
| **1471** | I3LJT1 | Uncharacterized protein | | 1.38E+06 | 1.38E+06 | 0.00E+00 | 0.00E+00 | 6.92E+04 | 6.92E+04 | 0.00E+00 | 0.00E+00 |
| **1472** | A2A2M0 | Regulation of nuclear pre-mRNA domain-containing protein 1B (Fragment) | RPRD1B | 6.15E+05 | 6.15E+05 | 0.00E+00 | 0.00E+00 | 6.84E+04 | 6.84E+04 | 0.00E+00 | 0.00E+00 |
| **1473** | F1RQL0 | Uncharacterized protein | SH3BGRL2 | 4.77E+05 | 0.00E+00 | 0.00E+00 | 4.77E+05 | 6.81E+04 | 0.00E+00 | 0.00E+00 | 6.81E+04 |
| **1474** | F1SHG3 | Uncharacterized protein | COMMD9 | 8.78E+05 | 2.59E+05 | 0.00E+00 | 6.20E+05 | 6.75E+04 | 1.99E+04 | 0.00E+00 | 4.77E+04 |
| **1475** | Q06AT8 | RHOB | RHOB | 7.43E+05 | 7.43E+05 | 0.00E+00 | 0.00E+00 | 6.75E+04 | 6.75E+04 | 0.00E+00 | 0.00E+00 |
| **1476** | A0A087WTA8 | Collagen alpha-2(I) chain | COL1A2 | 5.40E+06 | 1.63E+06 | 1.28E+06 | 2.49E+06 | 6.75E+04 | 2.03E+04 | 1.60E+04 | 3.12E+04 |

**Supplementary Table S1: (continued)**

| | | | | | | | | | | | |
|---|---|---|---|---|---|---|---|---|---|---|---|
| **1477** | F1SIJ9 | Uncharacterized protein | PSAT1 | 1.89E+06 | 1.43E+06 | 4.61E+05 | 0.00E+00 | 6.75E+04 | 5.10E+04 | 1.65E+04 | 0.00E+00 |
| **1478** | I3LTI5 | Deoxyribonuclease-1-like 1 | DNASE1L1 | 1.14E+06 | 5.14E+05 | 6.25E+05 | 0.00E+00 | 6.70E+04 | 3.02E+04 | 3.68E+04 | 0.00E+00 |
| **1479** | F1S4Y9 | Uncharacterized protein | RALY | 1.34E+06 | 4.69E+05 | 8.67E+05 | 0.00E+00 | 6.68E+04 | 2.34E+04 | 4.34E+04 | 0.00E+00 |
| **1480** | F1RYY7 | Uncharacterized protein | PDDC1 | 8.01E+05 | 2.98E+05 | 5.03E+05 | 0.00E+00 | 6.67E+04 | 2.48E+04 | 4.19E+04 | 0.00E+00 |
| **1481** | I3L725 | Uncharacterized protein | RHOG | 3.34E+05 | 3.34E+05 | 0.00E+00 | 0.00E+00 | 6.67E+04 | 6.67E+04 | 0.00E+00 | 0.00E+00 |
| **1482** | B5BU72 | Phosphatidylinositol-binding clathrin assembly protein isoform 2 | PICALM | 1.73E+06 | 6.83E+05 | 5.80E+05 | 4.65E+05 | 6.64E+04 | 2.63E+04 | 2.23E+04 | 1.79E+04 |
| **1483** | F1SK12 | Uncharacterized protein | MAP1B | 7.70E+06 | 6.56E+05 | 4.58E+06 | 2.47E+06 | 6.64E+04 | 5.66E+03 | 3.94E+04 | 2.13E+04 |
| **1484** | E9PNR9 | Protein arginine N-methyltransferase 1 (Fragment) | PRMT1 | 5.91E+05 | 5.91E+05 | 0.00E+00 | 0.00E+00 | 6.56E+04 | 6.56E+04 | 0.00E+00 | 0.00E+00 |
| **1485** | C9JKG1 | Biglycan (Fragment) | BGN | 7.19E+05 | 0.00E+00 | 7.19E+05 | 0.00E+00 | 6.54E+04 | 0.00E+00 | 6.54E+04 | 0.00E+00 |
| **1486** | Q49AG2 | TMED5 protein | TMED5 | 6.53E+05 | 3.46E+05 | 3.07E+05 | 0.00E+00 | 6.53E+04 | 3.46E+04 | 3.07E+04 | 0.00E+00 |
| **1487** | P23229 | Integrin alpha-6 | ITGA6 | 4.48E+06 | 1.46E+06 | 1.57E+06 | 1.45E+06 | 6.49E+04 | 2.11E+04 | 2.28E+04 | 2.10E+04 |
| **1488** | F1RUV5 | Uncharacterized protein | PC | 2.43E+06 | 1.00E+06 | 6.77E+05 | 7.51E+05 | 6.39E+04 | 2.63E+04 | 1.78E+04 | 1.98E+04 |
| **1489** | I3LQ57 | Uncharacterized protein | PTER | 1.40E+06 | 1.31E+05 | 4.56E+05 | 8.09E+05 | 6.35E+04 | 5.96E+03 | 2.07E+04 | 3.68E+04 |
| **1490** | P81693 | Low molecular weight phosphotyrosine protein phosphatase | ACP1 | 5.70E+05 | 5.70E+05 | 0.00E+00 | 0.00E+00 | 6.33E+04 | 6.33E+04 | 0.00E+00 | 0.00E+00 |
| **1491** | F1RF28 | Uncharacterized protein | | 2.71E+06 | 2.07E+06 | 4.50E+05 | 1.92E+05 | 6.30E+04 | 4.80E+04 | 1.05E+04 | 4.47E+03 |
| **1492** | F1SRV4 | Glutathione S-transferase kappa 1 | GSTK1 | 1.06E+06 | 8.63E+05 | 0.00E+00 | 1.98E+05 | 6.24E+04 | 5.08E+04 | 0.00E+00 | 1.17E+04 |
| **1493** | B3KY79 | cDNA FLJ46620 fis, clone TLUNG2000654, highly similar to Keratin, type II cytoskeletal 7 | | 2.00E+06 | 1.68E+06 | 3.19E+05 | 0.00E+00 | 6.24E+04 | 5.24E+04 | 9.97E+03 | 0.00E+00 |
| **1494** | I3LI38 | Uncharacterized protein | ATF7IP2 | 2.55E+06 | 4.30E+05 | 0.00E+00 | 2.12E+06 | 6.23E+04 | 1.05E+04 | 0.00E+00 | 5.18E+04 |
| **1495** | F1RZC6 | Uncharacterized protein | CCDC141 | 5.42E+06 | 0.00E+00 | 0.00E+00 | 5.42E+06 | 6.16E+04 | 0.00E+00 | 0.00E+00 | 6.16E+04 |
| **1496** | Q9H6N6 | Putative uncharacterized protein MYH16 | MYH16 | 5.04E+06 | 3.47E+06 | 1.29E+06 | 2.73E+05 | 6.14E+04 | 4.24E+04 | 1.57E+04 | 3.33E+03 |
| **1497** | H7C400 | 3-hydroxyisobutyryl-CoA hydrolase, mitochondrial (Fragment) | HIBCH | 2.45E+05 | 2.45E+05 | 0.00E+00 | 0.00E+00 | 6.12E+04 | 6.12E+04 | 0.00E+00 | 0.00E+00 |
| **1498** | A0A024R972 | Laminin, gamma 1 (Formerly LAMB2), isoform CRA | LAMC1 | 5.08E+06 | 2.49E+06 | 0.00E+00 | 2.58E+06 | 6.12E+04 | 3.00E+04 | 0.00E+00 | 3.11E+04 |
| **1499** | F1SII4 | Uncharacterized protein | GARS | 3.16E+06 | 1.85E+06 | 7.52E+05 | 5.64E+05 | 6.08E+04 | 3.55E+04 | 1.45E+04 | 1.08E+04 |
| **1500** | Q8TBT6 | Putative uncharacterized protein (Fragment) | | 1.03E+06 | 1.03E+06 | 0.00E+00 | 0.00E+00 | 6.05E+04 | 6.05E+04 | 0.00E+00 | 0.00E+00 |
| **1501** | F2Z530 | Ubiquitin-fold modifier-conjugating enzyme 1 | UFC1 | 7.24E+05 | 7.24E+05 | 0.00E+00 | 0.00E+00 | 6.04E+04 | 6.04E+04 | 0.00E+00 | 0.00E+00 |
| **1502** | Q5U091 | Neuroblastoma RAS viral (V-ras) oncogene homolog | NRAS | 6.64E+05 | 6.64E+05 | 0.00E+00 | 0.00E+00 | 6.04E+04 | 6.04E+04 | 0.00E+00 | 0.00E+00 |
| **1503** | A2ACR1 | Proteasome subunit beta type | PSMB9 | 6.00E+05 | 1.42E+05 | 2.67E+05 | 1.90E+05 | 6.00E+04 | 1.42E+04 | 2.67E+04 | 1.90E+04 |
| **1504** | F1SJ86 | Uncharacterized protein | CSPG4 | 5.86E+06 | 1.96E+05 | 2.37E+06 | 3.30E+06 | 5.98E+04 | 2.00E+03 | 2.42E+04 | 3.37E+04 |
| **1505** | I3LBG8 | Uncharacterized protein | PON3 | 8.25E+05 | 3.58E+05 | 4.68E+05 | 0.00E+00 | 5.90E+04 | 2.56E+04 | 3.34E+04 | 0.00E+00 |
| **1506** | F2Z5K5 | Uncharacterized protein | TUBB4A | 1.30E+06 | 1.30E+06 | 0.00E+00 | 0.00E+00 | 5.89E+04 | 5.89E+04 | 0.00E+00 | 0.00E+00 |
| **1507** | A0A0U5BLD0 | Inactive dehydrogenase/reductase isoform | spDHRS11 | 9.42E+05 | 9.42E+05 | 0.00E+00 | 0.00E+00 | 5.89E+04 | 5.89E+04 | 0.00E+00 | 0.00E+00 |
| **1508** | Q5TFK1 | Eukaryotic translation initiation factor 3 subunit I (Fragment) | EIF3I | 6.48E+05 | 6.48E+05 | 0.00E+00 | 0.00E+00 | 5.89E+04 | 5.89E+04 | 0.00E+00 | 0.00E+00 |
| **1509** | A0A024QZE7 | Transforming growth factor beta 1 induced transcript 1, isoform CRA | TGFB1I1 | 1.47E+06 | 1.25E+06 | 2.22E+05 | 0.00E+00 | 5.88E+04 | 4.99E+04 | 8.89E+03 | 0.00E+00 |
| **1510** | Q5JR08 | Rho-related GTP-binding protein RhoC (Fragment) | RHOC | 6.46E+05 | 0.00E+00 | 0.00E+00 | 6.46E+05 | 5.87E+04 | 0.00E+00 | 0.00E+00 | 5.87E+04 |
| **1511** | F2Z5R6 | Uncharacterized protein | CAND1 | 3.88E+06 | 2.08E+06 | 1.06E+06 | 7.46E+05 | 5.79E+04 | 3.10E+04 | 1.58E+04 | 1.11E+04 |
| **1512** | F1RU12 | Uncharacterized protein | LACTB2 | 9.27E+05 | 5.33E+05 | 3.93E+05 | 0.00E+00 | 5.79E+04 | 3.33E+04 | 2.46E+04 | 0.00E+00 |
| **1513** | K9J4P3 | UDP-glucose glycoprotein glucosyltransferase 1 | UGGT1 | 4.95E+06 | 9.20E+05 | 2.87E+06 | 1.17E+06 | 5.76E+04 | 1.07E+04 | 3.33E+04 | 1.36E+04 |
| **1514** | I3LBF1 | Inhibitor of carbonic anhydrase | ICA | 2.22E+06 | 5.26E+05 | 3.86E+05 | 1.31E+06 | 5.69E+04 | 1.35E+04 | 9.90E+03 | 3.35E+04 |
| **1515** | B7ZA74 | cDNA, FLJ79088, highly similar to UV excision repair protein RAD23 homolog B | | 6.82E+05 | 0.00E+00 | 6.82E+05 | 0.00E+00 | 5.68E+04 | 0.00E+00 | 5.68E+04 | 0.00E+00 |
| **1516** | F1S7K2 | Uncharacterized protein | LRG1 | 1.25E+06 | 2.27E+05 | 1.02E+06 | 0.00E+00 | 5.67E+04 | 1.03E+04 | 4.64E+04 | 0.00E+00 |
| **1517** | F1SI88 | Beta-hexosaminidase | HEXA | 1.47E+06 | 7.26E+05 | 4.41E+05 | 3.00E+05 | 5.64E+04 | 2.79E+04 | 1.69E+04 | 1.15E+04 |
| **1518** | F1SK02 | Uncharacterized protein | ARPIN | 7.90E+05 | 7.90E+05 | 0.00E+00 | 0.00E+00 | 5.64E+04 | 5.64E+04 | 0.00E+00 | 0.00E+00 |
| **1519** | K9IVQ6 | Plectin (Fragment) | PLEC | 1.50E+07 | 8.07E+06 | 2.67E+06 | 4.30E+06 | 5.59E+04 | 3.00E+04 | 9.92E+03 | 1.60E+04 |
| **1520** | P14543 | Nidogen-1 | NID1 | 3.02E+06 | 2.05E+06 | 4.78E+05 | 4.88E+05 | 5.49E+04 | 3.73E+04 | 8.69E+03 | 8.88E+03 |
| **1521** | F1RP05 | Uncharacterized protein | DNAJA2 | 1.37E+06 | 1.37E+06 | 0.00E+00 | 0.00E+00 | 5.48E+04 | 5.48E+04 | 0.00E+00 | 0.00E+00 |
| **1522** | F1SP54 | Uncharacterized protein | NIPSNAP3B | 9.31E+05 | 9.31E+05 | 0.00E+00 | 0.00E+00 | 5.48E+04 | 5.48E+04 | 0.00E+00 | 0.00E+00 |
| **1523** | F5XVC2 | von Willebrand factor | VWF | 7.57E+06 | 2.71E+06 | 2.27E+06 | 2.59E+06 | 5.45E+04 | 1.95E+04 | 1.64E+04 | 1.86E+04 |
| **1524** | Q5JQ44 | Putative uncharacterized protein DKFZp547A0616 (Fragment) | DKFZp547A0616 | 5.96E+05 | 3.54E+05 | 2.42E+05 | 0.00E+00 | 5.42E+04 | 3.22E+04 | 2.20E+04 | 0.00E+00 |
| **1525** | H0YFU8 | Tubby-related protein 3 (Fragment) | TULP3 | 4.33E+05 | 0.00E+00 | 0.00E+00 | 4.33E+05 | 5.41E+04 | 0.00E+00 | 0.00E+00 | 5.41E+04 |
| **1526** | I3LLX8 | Sulfotransferase | | 1.24E+06 | 1.24E+06 | 0.00E+00 | 0.00E+00 | 5.39E+04 | 5.39E+04 | 0.00E+00 | 0.00E+00 |
| **1527** | B7Z1M6 | cDNA FLJ57716, highly similar to 40S ribosomal protein S4, X isoform | Rps4 | 5.90E+05 | 5.90E+05 | 0.00E+00 | 0.00E+00 | 5.36E+04 | 5.36E+04 | 0.00E+00 | 0.00E+00 |
| **1528** | V9HW42 | Epididymis secretory protein Li 105 | HEL-S-105 | 1.98E+06 | 6.03E+05 | 0.00E+00 | 1.37E+06 | 5.34E+04 | 1.63E+04 | 0.00E+00 | 3.71E+04 |
| **1529** | I3LRF8 | Uncharacterized protein | PCBP3 | 9.08E+05 | 9.08E+05 | 0.00E+00 | 0.00E+00 | 5.34E+04 | 5.34E+04 | 0.00E+00 | 0.00E+00 |
| **1530** | F1RL80 | Branched-chain-amino-acid aminotransferase | BCAT2 | 1.27E+06 | 1.08E+06 | 1.96E+05 | 0.00E+00 | 5.30E+04 | 4.49E+04 | 8.19E+03 | 0.00E+00 |
| **1531** | Q8HX66 | MHC class I antigen (Fragment) | SLA-1 | 1.11E+06 | 1.11E+06 | 0.00E+00 | 0.00E+00 | 5.29E+04 | 5.29E+04 | 0.00E+00 | 0.00E+00 |
| **1532** | D6RGE0 | 40S ribosomal protein S3a (Fragment) | RPS3A | 2.10E+05 | 0.00E+00 | 2.10E+05 | 0.00E+00 | 5.26E+04 | 0.00E+00 | 5.26E+04 | 0.00E+00 |
| **1533** | F1S3P5 | Uncharacterized protein | ENPP1 | 2.41E+06 | 4.77E+05 | 4.54E+05 | 1.48E+06 | 5.13E+04 | 1.02E+04 | 9.65E+03 | 3.15E+04 |
| **1534** | F1SL03 | Uncharacterized protein | | 2.82E+06 | 1.41E+06 | 1.41E+06 | 0.00E+00 | 5.12E+04 | 2.56E+04 | 2.56E+04 | 0.00E+00 |
| **1535** | B4DGN8 | cDNA FLJ53377, highly similar to Procollagen-lysine, 2-oxoglutarate 5-dioxygenase 1 (EC 1.14.11.4) | | 2.35E+06 | 0.00E+00 | 5.43E+05 | 1.81E+06 | 5.11E+04 | 0.00E+00 | 1.18E+04 | 3.93E+04 |
| **1536** | I3LC92 | Uncharacterized protein | ENOSF1 | 1.37E+06 | 0.00E+00 | 4.62E+05 | 9.09E+05 | 5.08E+04 | 0.00E+00 | 1.71E+04 | 3.37E+04 |
| **1537** | F1RNT9 | Uncharacterized protein | LNPEP | 2.87E+06 | 1.15E+06 | 6.47E+05 | 1.07E+06 | 5.03E+04 | 2.02E+04 | 1.14E+04 | 1.88E+04 |
| **1538** | F1SFI4 | Uncharacterized protein | KNG1 | 1.43E+06 | 0.00E+00 | 1.43E+06 | 0.00E+00 | 4.92E+04 | 0.00E+00 | 4.92E+04 | 0.00E+00 |
| **1539** | K7GN08 | Uncharacterized protein | L1CAM | 3.14E+06 | 2.63E+06 | 1.70E+05 | 3.38E+05 | 4.90E+04 | 4.11E+04 | 2.65E+03 | 5.28E+03 |
| **1540** | E9PMR5 | Myelin basic protein | MBP | 3.92E+05 | 0.00E+00 | 3.92E+05 | 0.00E+00 | 4.90E+04 | 0.00E+00 | 4.90E+04 | 0.00E+00 |
| **1541** | Q0VG54 | TNS1 protein | TNS1 | 4.31E+06 | 2.03E+06 | 4.51E+05 | 1.82E+06 | 4.90E+04 | 2.31E+04 | 5.12E+03 | 2.07E+04 |
| **1542** | E7EPB3 | 60S ribosomal protein L14 | RPL14 | 4.87E+05 | 0.00E+00 | 4.87E+05 | 0.00E+00 | 4.87E+04 | 0.00E+00 | 4.87E+04 | 0.00E+00 |
| **1543** | F1SN05 | Eukaryotic translation initiation factor 3 subunit J | EIF3J | 7.70E+05 | 6.49E+05 | 1.21E+05 | 0.00E+00 | 4.81E+04 | 4.06E+04 | 7.57E+03 | 0.00E+00 |
| **1544** | K7GQL2 | Coagulation factor XIII, A1 polypeptide | F13A1 | 2.20E+06 | 7.20E+05 | 1.09E+06 | 3.97E+05 | 4.79E+04 | 1.57E+04 | 2.36E+04 | 8.63E+03 |
| **1545** | P09497 | Clathrin light chain B | CLTB | 4.74E+05 | 0.00E+00 | 4.74E+05 | 0.00E+00 | 4.74E+04 | 0.00E+00 | 4.74E+04 | 0.00E+00 |
| **1546** | I3L7Z6 | Protein S100 | S100A6 | 2.84E+05 | 0.00E+00 | 0.00E+00 | 2.84E+05 | 4.73E+04 | 0.00E+00 | 0.00E+00 | 4.73E+04 |
| **1547** | F1S2E3 | Peptidyl-prolyl cis-trans isomerase | PPIF | 7.92E+05 | 7.92E+05 | 0.00E+00 | 0.00E+00 | 4.66E+04 | 4.66E+04 | 0.00E+00 | 0.00E+00 |
| **1548** | F1SAR5 | Uncharacterized protein | GLRX5 | 4.19E+05 | 0.00E+00 | 4.19E+05 | 0.00E+00 | 4.66E+04 | 0.00E+00 | 4.66E+04 | 0.00E+00 |
| **1549** | I3LFQ5 | 6-phosphofructokinase | PFKL | 2.00E+06 | 1.06E+06 | 4.24E+05 | 5.18E+05 | 4.65E+04 | 2.46E+04 | 9.87E+03 | 1.21E+04 |
| **1550** | F1SKG6 | Sodium/potassium-transporting ATPase subunit beta | ATP1B3 | 6.48E+05 | 0.00E+00 | 0.00E+00 | 6.48E+05 | 4.63E+04 | 0.00E+00 | 0.00E+00 | 4.63E+04 |
| **1551** | F2Z5W2 | Cell division control protein 42 homolog | CDC42 | 4.14E+05 | 4.14E+05 | 0.00E+00 | 0.00E+00 | 4.60E+04 | 4.60E+04 | 0.00E+00 | 0.00E+00 |
| **1552** | Q9TUQ3 | Complement component C7 | C7 | 2.33E+06 | 6.26E+05 | 5.02E+05 | 1.21E+06 | 4.57E+04 | 1.23E+04 | 9.85E+03 | 2.36E+04 |
| **1553** | I3LVG4 | GrpE protein homolog | GRPEL1 | 8.11E+05 | 2.97E+05 | 2.45E+05 | 2.68E+05 | 4.50E+04 | 1.65E+04 | 1.36E+04 | 1.49E+04 |
| **1554** | Q9HBM8 | cDNA, FLJ78849, highly similar to 1-acyl-sn-glycerol-3-phosphate acyltransferase alpha (EC 2.3.1.51) | | 5.35E+05 | 5.35E+05 | 0.00E+00 | 0.00E+00 | 4.46E+04 | 4.46E+04 | 0.00E+00 | 0.00E+00 |
| **1555** | B4E3I0 | cDNA FLJ55017, highly similar to Caldesmon | CALD1 | 9.76E+05 | 9.76E+05 | 0.00E+00 | 0.00E+00 | 4.44E+04 | 4.44E+04 | 0.00E+00 | 0.00E+00 |
| **1556** | B3KSM1 | cDNA FLJ36593 fis, clone TRACH2014077, highly similar to Homo sapiens influenza virus NS1A binding protein (IVNS1ABP), transcript variant 1, mRNA | | 6.20E+05 | 0.00E+00 | 6.20E+05 | 0.00E+00 | 4.43E+04 | 0.00E+00 | 4.43E+04 | 0.00E+00 |
| **1557** | K7GQB8 | Uncharacterized protein | CP | 2.51E+06 | 6.62E+05 | 4.49E+05 | 1.40E+06 | 4.41E+04 | 1.16E+04 | 7.87E+03 | 2.46E+04 |
| **1558** | B4DUT1 | cDNA FLJ52688, highly similar to SET and MYND domain-containing protein 5 | SMYD5 | 5.27E+05 | 5.27E+05 | 0.00E+00 | 0.00E+00 | 4.39E+04 | 4.39E+04 | 0.00E+00 | 0.00E+00 |
| **1559** | I3L677 | Glucose-6-phosphate 1-dehydrogenase | G6PD | 1.60E+06 | 4.85E+05 | 1.12E+05 | 1.01E+06 | 4.34E+04 | 1.31E+04 | 3.02E+03 | 2.72E+04 |
| **1560** | F1SJR5 | Uncharacterized protein | PACSIN2 | 1.17E+06 | 0.00E+00 | 1.17E+06 | 0.00E+00 | 4.32E+04 | 0.00E+00 | 4.32E+04 | 0.00E+00 |
| **1561** | H0YF44 | N-acetyl-D-glucosamine kinase (Fragment) | NAGK | 3.44E+05 | 3.44E+05 | 0.00E+00 | 0.00E+00 | 4.30E+04 | 4.30E+04 | 0.00E+00 | 0.00E+00 |
| **1562** | F1SGI2 | Uncharacterized protein | KRT84 | 1.23E+06 | 0.00E+00 | 0.00E+00 | 1.23E+06 | 4.25E+04 | 0.00E+00 | 0.00E+00 | 4.25E+04 |
| **1563** | F1RPD7 | Uncharacterized protein | SMTN | 2.23E+06 | 1.14E+06 | 3.45E+05 | 7.47E+05 | 4.13E+04 | 2.11E+04 | 6.39E+03 | 1.38E+04 |
| **1564** | Q8HZQ4 | Thioredoxin reductase (Fragment) | TrxR1 | 1.07E+06 | 0.00E+00 | 1.07E+06 | 0.00E+00 | 4.12E+04 | 0.00E+00 | 4.12E+04 | 0.00E+00 |
| **1565** | P04040 | Catalase | CAT | 1.32E+06 | 0.00E+00 | 0.00E+00 | 1.32E+06 | 4.12E+04 | 0.00E+00 | 0.00E+00 | 4.12E+04 |
| **1566** | A1L0S9 | CROCC protein (Fragment) | CROCC | 3.54E+06 | 5.46E+05 | 2.31E+06 | 6.85E+05 | 4.11E+04 | 6.34E+03 | 2.68E+04 | 7.97E+03 |
| **1567** | F1SCF0 | Alpha-1-antitrypsin | SERPINA1 | 9.86E+05 | 0.00E+00 | 0.00E+00 | 9.86E+05 | 4.11E+04 | 0.00E+00 | 0.00E+00 | 4.11E+04 |
| **1568** | F1SQP4 | Uncharacterized protein | NDUFA12 | 4.10E+05 | 4.10E+05 | 0.00E+00 | 0.00E+00 | 4.10E+04 | 4.10E+04 | 0.00E+00 | 0.00E+00 |
| **1569** | F1SN58 | Uncharacterized protein | LOC100152170 | 1.47E+06 | 1.47E+06 | 0.00E+00 | 0.00E+00 | 4.07E+04 | 4.07E+04 | 0.00E+00 | 0.00E+00 |
| **1570** | I3LCU8 | Uncharacterized protein | LSM1 | 4.46E+05 | 0.00E+00 | 0.00E+00 | 4.46E+05 | 4.06E+04 | 0.00E+00 | 0.00E+00 | 4.06E+04 |
| **1571** | A0A024R2Q5 | Myosin, light polypeptide 3, alkali ventricular, skeletal, slow, isoform CRA | MYL3 | 5.27E+05 | 5.27E+05 | 0.00E+00 | 0.00E+00 | 4.05E+04 | 4.05E+04 | 0.00E+00 | 0.00E+00 |
| **1572** | F1SD78 | Methylthioribose-1-phosphate isomerase | MRI1 | 9.54E+05 | 4.26E+05 | 5.28E+05 | 0.00E+00 | 3.98E+04 | 1.78E+04 | 2.20E+04 | 0.00E+00 |
| **1573** | H7C5W5 | Peripherin (Fragment) | PRPH | 6.27E+05 | 0.00E+00 | 2.28E+05 | 4.00E+05 | 3.92E+04 | 0.00E+00 | 1.42E+04 | 2.50E+04 |
| **1574** | F1RUX1 | Coronin | CORO1B | 1.05E+06 | 0.00E+00 | 0.00E+00 | 1.05E+06 | 3.88E+04 | 0.00E+00 | 0.00E+00 | 3.88E+04 |
| **1575** | F1S8R5 | Signal recognition particle 9 kDa protein | SRP9 | 2.71E+05 | 0.00E+00 | 0.00E+00 | 2.71E+05 | 3.88E+04 | 0.00E+00 | 0.00E+00 | 3.88E+04 |
| **1576** | M3UZ72 | Lipase maturation factor | LMF2 | 1.04E+06 | 1.04E+06 | 0.00E+00 | 0.00E+00 | 3.86E+04 | 3.86E+04 | 0.00E+00 | 0.00E+00 |
| **1577** | F1RQJ9 | Uncharacterized protein | IMPG1 | 1.56E+06 | 2.16E+05 | 6.26E+05 | 7.21E+05 | 3.72E+04 | 5.15E+03 | 1.49E+04 | 1.72E+04 |
| **1578** | Q96HI1 | Similar to plastin 3 (T isoform) (Fragment) | PLS3 | 1.00E+06 | 1.00E+06 | 0.00E+00 | 0.00E+00 | 3.71E+04 | 3.71E+04 | 0.00E+00 | 0.00E+00 |
| **1579** | C9IZL7 | Non-POU domain-containing octamer-binding protein (Fragment) | NONO | 4.08E+05 | 0.00E+00 | 2.00E+05 | 2.08E+05 | 3.71E+04 | 0.00E+00 | 1.82E+04 | 1.90E+04 |
| **1580** | D0G6Y3 | Estradiol 17-beta-dehydrogenase 8 | HSD17B8 | 4.45E+05 | 4.45E+05 | 0.00E+00 | 0.00E+00 | 3.70E+04 | 3.70E+04 | 0.00E+00 | 0.00E+00 |
| **1581** | F1SLX2 | Uncharacterized protein | A2M | 3.14E+06 | 1.73E+06 | 4.20E+05 | 9.96E+05 | 3.70E+04 | 2.03E+04 | 4.94E+03 | 1.17E+04 |
| **1582** | P07737 | Profilin-1 | PFN1 | 3.33E+05 | 0.00E+00 | 0.00E+00 | 3.33E+05 | 3.70E+04 | 0.00E+00 | 0.00E+00 | 3.70E+04 |
| **1583** | D6RHZ7 | Rap1 GTPase-GDP dissociation stimulator 1 (Fragment) | RAP1GDS1 | 2.58E+05 | 0.00E+00 | 2.58E+05 | 0.00E+00 | 3.69E+04 | 0.00E+00 | 3.69E+04 | 0.00E+00 |
| **1584** | F1STD5 | Uncharacterized protein | ACTR1B | 8.38E+05 | 0.00E+00 | 0.00E+00 | 8.38E+05 | 3.64E+04 | 0.00E+00 | 0.00E+00 | 3.64E+04 |
| **1585** | C3RZA0 | SET and MYND domain-containing 1 (Fragment) | SMYD1 | 3.25E+05 | 3.25E+05 | 0.00E+00 | 0.00E+00 | 3.61E+04 | 3.61E+04 | 0.00E+00 | 0.00E+00 |
| **1586** | Q63HR1 | Putative uncharacterized protein DKFZp686P17171 | DKFZp686P17171 | 7.16E+05 | 4.07E+05 | 0.00E+00 | 3.09E+05 | 3.58E+04 | 2.04E+04 | 0.00E+00 | 1.54E+04 |
| **1587** | A6NLG9 | cDNA FLJ36740 fis, clone UTERU2013322, highly similar to Biglycan | | 6.79E+05 | 1.87E+05 | 2.10E+05 | 2.82E+05 | 3.57E+04 | 9.84E+03 | 1.11E+04 | 1.49E+04 |
| **1588** | K7GT00 | Uncharacterized protein (Fragment) | COL14A1 | 3.04E+06 | 3.09E+05 | 1.34E+06 | 1.39E+06 | 3.57E+04 | 3.64E+03 | 1.58E+04 | 1.63E+04 |
| **1589** | A0A0D9SGF6 | Spectrin alpha chain, non-erythrocytic 1 | SPTAN1 | 6.89E+06 | 5.14E+06 | 1.08E+06 | 6.73E+05 | 3.57E+04 | 2.66E+04 | 5.61E+03 | 3.49E+03 |
| **1590** | F1S790 | Uncharacterized protein | C8B | 1.29E+06 | 7.92E+05 | 0.00E+00 | 4.94E+05 | 3.57E+04 | 2.20E+04 | 0.00E+00 | 1.37E+04 |
| **1591** | I3LU92 | Uncharacterized protein | TATDN1 | 4.64E+05 | 4.64E+05 | 0.00E+00 | 0.00E+00 | 3.57E+04 | 3.57E+04 | 0.00E+00 | 0.00E+00 |
| **1592** | F1RW31 | Uncharacterized protein | NUDT9 | 7.76E+05 | 7.76E+05 | 0.00E+00 | 0.00E+00 | 3.53E+04 | 3.53E+04 | 0.00E+00 | 0.00E+00 |
| **1593** | F1SQS2 | Uncharacterized protein | TMPO | 4.23E+05 | 0.00E+00 | 0.00E+00 | 4.23E+05 | 3.53E+04 | 0.00E+00 | 0.00E+00 | 3.53E+04 |
| **1594** | B4E1T4 | cDNA FLJ53955, highly similar to Secreted frizzled-related protein 4 | | 5.98E+05 | 0.00E+00 | 5.98E+05 | 0.00E+00 | 3.51E+04 | 0.00E+00 | 3.51E+04 | 0.00E+00 |
| **1595** | A0A024R1A3 | Testicular secretory protein Li 63 | UBE1 | 2.06E+06 | 0.00E+00 | 7.17E+05 | 1.34E+06 | 3.49E+04 | 0.00E+00 | 1.22E+04 | 2.28E+04 |
| **1596** | P02540 | Desmin | DES | 1.26E+06 | 1.26E+06 | 0.00E+00 | 0.00E+00 | 3.49E+04 | 3.49E+04 | 0.00E+00 | 0.00E+00 |
| **1597** | Q53FA3 | Heat shock 70 kDa protein 1-like (Fragment) | HSPA1L | 1.39E+06 | 1.39E+06 | 0.00E+00 | 0.00E+00 | 3.47E+04 | 3.47E+04 | 0.00E+00 | 0.00E+00 |
| **1598** | F1RMF9 | Uncharacterized protein | FAM111B | 1.65E+06 | 0.00E+00 | 0.00E+00 | 1.65E+06 | 3.45E+04 | 0.00E+00 | 0.00E+00 | 3.45E+04 |
| **1599** | A0A024R321 | Filamin B, beta (Actin binding protein 278), isoform CRA | FLNB | 5.43E+06 | 6.70E+05 | 2.20E+06 | 2.56E+06 | 3.41E+04 | 4.22E+03 | 1.38E+04 | 1.61E+04 |
| **1600** | Q3LIE0 | Putative uncharacterized protein Nbla00315 (Fragment) | Nbla00315 | 3.73E+05 | 1.42E+05 | 0.00E+00 | 2.31E+05 | 3.39E+04 | 1.30E+04 | 0.00E+00 | 2.10E+04 |
| **1601** | F1RX66 | Uncharacterized protein | GSR | 8.28E+05 | 8.28E+05 | 0.00E+00 | 0.00E+00 | 3.31E+04 | 3.31E+04 | 0.00E+00 | 0.00E+00 |
| **1602** | B7Z4V2 | cDNA FLJ51907, highly similar to Stress-70 protein, mitochondrial | | 1.34E+06 | 5.31E+05 | 8.07E+05 | 0.00E+00 | 3.27E+04 | 1.30E+04 | 1.97E+04 | 0.00E+00 |
| **1603** | B4DLP6 | Serine/threonine-protein phosphatase | PPP5C | 9.78E+05 | 0.00E+00 | 0.00E+00 | 9.78E+05 | 3.26E+04 | 0.00E+00 | 0.00E+00 | 3.26E+04 |
| **1604** | I3LAQ4 | Uncharacterized protein | LOC100736917 | 6.91E+05 | 9.89E+04 | 2.26E+05 | 3.66E+05 | 3.14E+04 | 4.50E+03 | 1.03E+04 | 1.66E+04 |
| **1605** | F1SJ93 | Uncharacterized protein | RCN2 | 4.06E+05 | 4.06E+05 | 0.00E+00 | 0.00E+00 | 3.13E+04 | 3.13E+04 | 0.00E+00 | 0.00E+00 |
| **1606** | P79384 | Propionyl-CoA carboxylase beta chain, mitochondrial | PCCB | 1.03E+06 | 0.00E+00 | 6.39E+05 | 3.88E+05 | 3.11E+04 | 0.00E+00 | 1.94E+04 | 1.18E+04 |
| **1607** | V5L1C3 | POSTN | POSTN | 1.27E+06 | 0.00E+00 | 2.95E+05 | 9.75E+05 | 3.10E+04 | 0.00E+00 | 7.19E+03 | 2.38E+04 |
| **1608** | B4DZQ7 | cDNA FLJ58652, highly similar to Probable ATP-dependent RNA helicase DDX17 (EC 3.6.1.-) | | 8.67E+05 | 8.67E+05 | 0.00E+00 | 0.00E+00 | 3.10E+04 | 3.10E+04 | 0.00E+00 | 0.00E+00 |
| **1609** | F1S4K0 | Fructose-1,6-bisphosphatase 1 | FBP1 | 5.54E+05 | 5.54E+05 | 0.00E+00 | 0.00E+00 | 3.08E+04 | 3.08E+04 | 0.00E+00 | 0.00E+00 |
| **1610** | E9PL24 | Myomegalin | PDE4DIP | 2.15E+06 | 5.51E+05 | 6.74E+05 | 9.22E+05 | 3.07E+04 | 7.87E+03 | 9.64E+03 | 1.32E+04 |
| **1611** | F1SGN7 | Uncharacterized protein | MDP1 | 3.05E+05 | 2.07E+05 | 9.82E+04 | 0.00E+00 | 3.05E+04 | 2.07E+04 | 9.82E+03 | 0.00E+00 |
| **1612** | I3LJ82 | Uncharacterized protein | PACSIN1 | 7.90E+05 | 7.90E+05 | 0.00E+00 | 0.00E+00 | 3.04E+04 | 3.04E+04 | 0.00E+00 | 0.00E+00 |
| **1613** | K7GPE7 | Uncharacterized protein | ADHFE1 | 5.43E+05 | 5.43E+05 | 0.00E+00 | 0.00E+00 | 3.02E+04 | 3.02E+04 | 0.00E+00 | 0.00E+00 |
| **1614** | P01030 | similar to Complement C4-A precursor | | 3.10E+06 | 6.03E+05 | 3.49E+05 | 2.14E+06 | 3.01E+04 | 5.86E+03 | 3.39E+03 | 2.08E+04 |
| **1615** | Q9NRW1 | Ras-related protein Rab-6B | RAB6B | 5.10E+05 | 1.71E+05 | 3.38E+05 | 0.00E+00 | 3.00E+04 | 1.01E+04 | 1.99E+04 | 0.00E+00 |
| **1616** | B7Z938 | cDNA FLJ61727, highly similar to Laminin alpha-4 chain (Fragment) | LAMA4 | 2.51E+06 | 1.47E+06 | 2.35E+05 | 8.01E+05 | 2.98E+04 | 1.75E+04 | 2.80E+03 | 9.54E+03 |
| **1617** | F1RP44 | Uncharacterized protein | IPO5 | 1.69E+06 | 4.98E+05 | 0.00E+00 | 1.19E+06 | 2.97E+04 | 8.74E+03 | 0.00E+00 | 2.09E+04 |
| **1618** | F1RQU4 | Uncharacterized protein | SLC29A1 | 4.75E+05 | 4.75E+05 | 0.00E+00 | 0.00E+00 | 2.97E+04 | 2.97E+04 | 0.00E+00 | 0.00E+00 |

**Supplementary Table S1: (continued)**

| | | | | | | | | | | | |
|---|---|---|---|---|---|---|---|---|---|---|---|
| **1619** | F1SL07 | Uncharacterized protein | NDUFA9 | 7.99E+05 | 0.00E+00 | 7.99E+05 | 0.00E+00 | 2.96E+04 | 0.00E+00 | 2.96E+04 | 0.00E+00 |
| **1620** | Q6EEI7 | Mannose receptor C1 (Fragment) | MRC1 | 2.28E+06 | 9.73E+05 | 4.49E+05 | 8.62E+05 | 2.93E+04 | 1.25E+04 | 5.75E+03 | 1.11E+04 |
| **1621** | F1SHR2 | Uncharacterized protein | BCL2L13 | 6.13E+05 | 3.16E+05 | 2.96E+05 | 0.00E+00 | 2.92E+04 | 1.51E+04 | 1.41E+04 | 0.00E+00 |
| **1622** | I3LLT4 | Uncharacterized protein | CYP20A1 | 2.61E+05 | 2.61E+05 | 0.00E+00 | 0.00E+00 | 2.90E+04 | 2.90E+04 | 0.00E+00 | 0.00E+00 |
| **1623** | F1S1A8 | Uncharacterized protein | NDUFS2 | 8.59E+05 | 0.00E+00 | 8.59E+05 | 0.00E+00 | 2.86E+04 | 0.00E+00 | 2.86E+04 | 0.00E+00 |
| **1624** | Q8IXJ3 | Small nuclear ribonucleoprotein component | SNRP116 | 1.50E+06 | 7.87E+05 | 7.11E+05 | 0.00E+00 | 2.83E+04 | 1.48E+04 | 1.34E+04 | 0.00E+00 |
| **1625** | P11607 | Sarcoplasmic/endoplasmic reticulum calcium ATPase 2 | ATP2A2 | 1.49E+06 | 1.49E+06 | 0.00E+00 | 0.00E+00 | 2.81E+04 | 2.81E+04 | 0.00E+00 | 0.00E+00 |
| **1626** | B4DEX5 | Prion protein interacting protein, isoform CRA | PRNPIP | 2.81E+05 | 2.81E+05 | 0.00E+00 | 0.00E+00 | 2.81E+04 | 2.81E+04 | 0.00E+00 | 0.00E+00 |
| **1627** | A0A024R1I3 | Pyridoxal (Pyridoxine, vitamin B6) phosphatase, isoform CRA | PDXP | 5.05E+05 | 0.00E+00 | 0.00E+00 | 5.05E+05 | 2.80E+04 | 0.00E+00 | 0.00E+00 | 2.80E+04 |
| **1628** | I3LLF2 | Uncharacterized protein | RPN2 | 7.25E+05 | 0.00E+00 | 7.25E+05 | 0.00E+00 | 2.79E+04 | 0.00E+00 | 2.79E+04 | 0.00E+00 |
| **1629** | A0A087X0P0 | Centromere-associated protein E | CENPE | 5.60E+06 | 1.70E+06 | 0.00E+00 | 3.90E+06 | 2.79E+04 | 8.45E+03 | 0.00E+00 | 1.94E+04 |
| **1630** | F1RKW8 | Uncharacterized protein | PSMD11 | 8.03E+05 | 8.03E+05 | 0.00E+00 | 0.00E+00 | 2.77E+04 | 2.77E+04 | 0.00E+00 | 0.00E+00 |
| **1631** | F1S091 | Uncharacterized protein | RAB8B | 4.07E+05 | 1.37E+05 | 2.70E+05 | 0.00E+00 | 2.71E+04 | 9.16E+03 | 1.80E+04 | 0.00E+00 |
| **1632** | Q8VED5 | Keratin 79 | Krt79 Keratin 79 | 8.69E+05 | 0.00E+00 | 0.00E+00 | 8.69E+05 | 2.71E+04 | 0.00E+00 | 0.00E+00 | 2.71E+04 |
| **1633** | Q8SPR7 | 1-phosphatidylinositol 4,5-bisphosphate phosphodiesterase delta-4 | PLCD4 | 8.03E+05 | 0.00E+00 | 2.50E+05 | 5.52E+05 | 2.68E+04 | 0.00E+00 | 8.35E+03 | 1.84E+04 |
| **1634** | E5RGJ2 | ER membrane protein complex subunit 2 (Fragment) | EMC2 | 1.87E+05 | 1.87E+05 | 0.00E+00 | 0.00E+00 | 2.66E+04 | 2.66E+04 | 0.00E+00 | 0.00E+00 |
| **1635** | Q9GLP2 | Vitamin K-dependent protein C | PROC | 7.63E+05 | 2.87E+05 | 4.75E+05 | 0.00E+00 | 2.54E+04 | 9.58E+03 | 1.58E+04 | 0.00E+00 |
| **1636** | P02666 | Beta-casein | | 2.03E+05 | 0.00E+00 | 0.00E+00 | 2.03E+05 | 2.53E+04 | 0.00E+00 | 0.00E+00 | 2.53E+04 |
| **1637** | F1SIX7 | Uncharacterized protein | LOC106504190 | 5.52E+05 | 5.52E+05 | 0.00E+00 | 0.00E+00 | 2.51E+04 | 2.51E+04 | 0.00E+00 | 0.00E+00 |
| **1638** | Q05BX4 | PSMD1 protein (Fragment) | PSMD1 | 1.08E+06 | 0.00E+00 | 1.19E+05 | 9.57E+05 | 2.50E+04 | 0.00E+00 | 2.76E+03 | 2.23E+04 |
| **1639** | B4DJ25 | cDNA FLJ52302, highly similar to Nuclear receptor corepressor 1 | | 1.54E+06 | 0.00E+00 | 0.00E+00 | 1.54E+06 | 2.49E+04 | 0.00E+00 | 0.00E+00 | 2.49E+04 |
| **1640** | A0A024R319 | Laminin, beta 2 (Laminin S), isoform CRA | LAMB2 | 2.64E+06 | 0.00E+00 | 1.17E+06 | 1.47E+06 | 2.42E+04 | 0.00E+00 | 1.07E+04 | 1.35E+04 |
| **1641** | F1SDX6 | Uncharacterized protein | TGM2 | 1.03E+06 | 5.98E+05 | 2.46E+05 | 1.86E+05 | 2.39E+04 | 1.39E+04 | 5.72E+03 | 4.32E+03 |
| **1642** | B4DIL5 | cDNA FLJ54516, highly similar to Adapter-related protein complex 2 beta-1 subunit | | 4.77E+05 | 0.00E+00 | 0.00E+00 | 4.77E+05 | 2.38E+04 | 0.00E+00 | 0.00E+00 | 2.38E+04 |
| **1643** | K7GP98 | Uncharacterized protein | TRIM23 | 7.39E+05 | 4.49E+05 | 1.77E+05 | 1.13E+05 | 2.38E+04 | 1.45E+04 | 5.70E+03 | 3.66E+03 |
| **1644** | F1RU52 | Uncharacterized protein | LOC100626654 | 1.05E+06 | 2.08E+05 | 5.55E+05 | 2.86E+05 | 2.38E+04 | 4.73E+03 | 1.26E+04 | 6.49E+03 |
| **1645** | Q4G1A8 | CAMK2D protein (Fragment) | CAMK2D | 4.52E+05 | 4.52E+05 | 0.00E+00 | 0.00E+00 | 2.38E+04 | 2.38E+04 | 0.00E+00 | 0.00E+00 |
| **1646** | P22612 | cAMP-dependent protein kinase catalytic subunit gamma | PRKACG | 4.74E+05 | 0.00E+00 | 4.74E+05 | 0.00E+00 | 2.37E+04 | 0.00E+00 | 2.37E+04 | 0.00E+00 |
| **1647** | F1SIG7 | Uncharacterized protein | SCRN1 | 4.47E+05 | 0.00E+00 | 4.47E+05 | 0.00E+00 | 2.35E+04 | 0.00E+00 | 2.35E+04 | 0.00E+00 |
| **1648** | F1RLR8 | Uncharacterized protein | GGT5 | 6.81E+05 | 5.14E+05 | 0.00E+00 | 1.67E+05 | 2.35E+04 | 1.77E+04 | 0.00E+00 | 5.77E+03 |
| **1649** | O14980 | Exportin-1 | XPO1 | 1.36E+06 | 1.02E+06 | 0.00E+00 | 3.39E+05 | 2.34E+04 | 1.76E+04 | 0.00E+00 | 5.84E+03 |
| **1650** | J3KNV4 | Integrin alpha-7 | ITGA7 | 1.26E+06 | 1.26E+06 | 0.00E+00 | 0.00E+00 | 2.34E+04 | 2.34E+04 | 0.00E+00 | 0.00E+00 |
| **1651** | I3LGD9 | Uncharacterized protein | AGRN | 2.47E+06 | 7.60E+05 | 1.71E+06 | 0.00E+00 | 2.33E+04 | 7.17E+03 | 1.62E+04 | 0.00E+00 |
| **1652** | A0A0S2Z3A5 | Acyl-CoA dehydrogenase C-4 to C-12 straight chain isoform 4 (Fragment) | ACADM | 3.29E+05 | 3.29E+05 | 0.00E+00 | 0.00E+00 | 2.19E+04 | 2.19E+04 | 0.00E+00 | 0.00E+00 |
| **1653** | F1SUN0 | Uncharacterized protein | ARRB1 | 4.94E+05 | 2.93E+05 | 2.01E+05 | 0.00E+00 | 2.15E+04 | 1.27E+04 | 8.74E+03 | 0.00E+00 |
| **1654** | I3L3J8 | Sodium/potassium-transporting ATPase subunit beta (Fragment) | ATP1B2 | 1.93E+05 | 0.00E+00 | 1.93E+05 | 0.00E+00 | 2.14E+04 | 0.00E+00 | 2.14E+04 | 0.00E+00 |
| **1655** | F1SSR4 | Uncharacterized protein | IVD | 4.85E+05 | 0.00E+00 | 0.00E+00 | 4.85E+05 | 2.11E+04 | 0.00E+00 | 0.00E+00 | 2.11E+04 |
| **1656** | Q13884 | Beta-1-syntrophin | SNTB1 | 7.38E+05 | 0.00E+00 | 7.38E+05 | 0.00E+00 | 2.11E+04 | 0.00E+00 | 2.11E+04 | 0.00E+00 |
| **1657** | Q9UKX3 | Myosin-13 | MYH13 | 2.60E+06 | 2.60E+06 | 0.00E+00 | 0.00E+00 | 2.08E+04 | 2.08E+04 | 0.00E+00 | 0.00E+00 |
| **1658** | J9JIL9 | 4-aminobutyrate aminotransferase, mitochondrial | ABAT | 6.74E+05 | 0.00E+00 | 4.29E+05 | 2.45E+05 | 2.04E+04 | 0.00E+00 | 1.30E+04 | 7.41E+03 |
| **1659** | F1RVH7 | Uncharacterized protein | IGFBP7 | 3.24E+05 | 3.24E+05 | 0.00E+00 | 0.00E+00 | 2.02E+04 | 2.02E+04 | 0.00E+00 | 0.00E+00 |
| **1660** | D6RD63 | COP9 signalosome complex subunit 4 (Fragment) | COPS4 | 3.96E+05 | 0.00E+00 | 3.96E+05 | 0.00E+00 | 1.98E+04 | 0.00E+00 | 1.98E+04 | 0.00E+00 |
| **1661** | Q5T1S7 | Serine/threonine-protein phosphatase (Fragment) | PPP6C | 1.18E+05 | 1.18E+05 | 0.00E+00 | 0.00E+00 | 1.97E+04 | 1.97E+04 | 0.00E+00 | 0.00E+00 |
| **1662** | I3LKD2 | Uncharacterized protein | FAM162A | 2.16E+05 | 2.16E+05 | 0.00E+00 | 0.00E+00 | 1.96E+04 | 1.96E+04 | 0.00E+00 | 0.00E+00 |
| **1663** | Q4W4Y1 | Dopamine receptor interacting protein 4 | DRIP4 | 1.09E+06 | 0.00E+00 | 4.69E+05 | 6.21E+05 | 1.95E+04 | 0.00E+00 | 8.38E+03 | 1.11E+04 |
| **1664** | B2RD36 | cDNA, FLJ96437 | | 3.28E+05 | 0.00E+00 | 3.28E+05 | 0.00E+00 | 1.93E+04 | 0.00E+00 | 1.93E+04 | 0.00E+00 |
| **1665** | F1S3Q8 | Uncharacterized protein | VNN3 | 4.38E+05 | 0.00E+00 | 2.09E+05 | 2.29E+05 | 1.90E+04 | 0.00E+00 | 9.07E+03 | 9.96E+03 |
| **1666** | B0I1S4 | DNHD1 variant protein (Fragment) | DNHD1 | 3.73E+06 | 3.64E+06 | 0.00E+00 | 9.09E+04 | 1.89E+04 | 1.85E+04 | 0.00E+00 | 4.62E+02 |
| **1667** | F1RQQ7 | Alpha-1,4 glucan phosphorylase | PYGB | 9.01E+05 | 0.00E+00 | 3.77E+05 | 5.23E+05 | 1.88E+04 | 0.00E+00 | 7.86E+03 | 1.09E+04 |
| **1668** | F1RWZ4 | Uncharacterized protein | ECI2 | 4.68E+05 | 4.68E+05 | 0.00E+00 | 0.00E+00 | 1.87E+04 | 1.87E+04 | 0.00E+00 | 0.00E+00 |
| **1669** | F1S8G5 | Thimet oligopeptidase | THOP1 | 7.56E+05 | 7.56E+05 | 0.00E+00 | 0.00E+00 | 1.85E+04 | 1.85E+04 | 0.00E+00 | 0.00E+00 |
| **1670** | F1S5S1 | Uncharacterized protein | IBA57 | 3.63E+05 | 2.27E+05 | 1.36E+05 | 0.00E+00 | 1.82E+04 | 1.14E+04 | 6.81E+03 | 0.00E+00 |
| **1671** | K7GQZ6 | Uncharacterized protein | ATP6AP2 | 3.27E+05 | 3.27E+05 | 0.00E+00 | 0.00E+00 | 1.81E+04 | 1.81E+04 | 0.00E+00 | 0.00E+00 |
| **1672** | A0A0U4BW16 | Non-muscle myosin heavy chain 9 | MYH9 | 2.20E+06 | 1.98E+06 | 2.23E+05 | 0.00E+00 | 1.76E+04 | 1.58E+04 | 1.78E+03 | 0.00E+00 |
| **1673** | F1SFM2 | Uncharacterized protein | CHL1 | 9.15E+05 | 9.15E+05 | 0.00E+00 | 0.00E+00 | 1.76E+04 | 1.76E+04 | 0.00E+00 | 0.00E+00 |
| **1674** | Q9UG85 | Putative uncharacterized protein DKFZp564O1822 (Fragment) | DKFZp564O1822 | 4.22E+05 | 0.00E+00 | 0.00E+00 | 4.22E+05 | 1.76E+04 | 0.00E+00 | 0.00E+00 | 1.76E+04 |
| **1675** | K9IVC5 | Protein-arginine deiminase type-2 | PADI2 | 6.50E+05 | 1.93E+05 | 0.00E+00 | 4.57E+05 | 1.76E+04 | 5.23E+03 | 0.00E+00 | 1.23E+04 |
| **1676** | F1SH60 | Uncharacterized protein | CPSF6 | 1.91E+05 | 0.00E+00 | 1.91E+05 | 0.00E+00 | 1.74E+04 | 0.00E+00 | 1.74E+04 | 0.00E+00 |
| **1677** | Q1T7A9 | Type VI collagen alpha-1 chain (Fragment) | COL6A1 | 1.21E+05 | 0.00E+00 | 0.00E+00 | 1.21E+05 | 1.72E+04 | 0.00E+00 | 0.00E+00 | 1.72E+04 |
| **1678** | V9HWA9 | Epididymis secretory sperm binding protein Li 62p | HEL-S-62p | 1.85E+06 | 0.00E+00 | 0.00E+00 | 1.85E+06 | 1.72E+04 | 0.00E+00 | 0.00E+00 | 1.72E+04 |
| **1679** | I3LMP2 | Uncharacterized protein | ABHD10 | 3.59E+05 | 3.59E+05 | 0.00E+00 | 0.00E+00 | 1.71E+04 | 1.71E+04 | 0.00E+00 | 0.00E+00 |
| **1680** | M3TYW5 | Glutamyl-prolyl-tRNA synthetase | QARS | 7.56E+05 | 2.57E+05 | 2.02E+05 | 2.97E+05 | 1.68E+04 | 5.72E+03 | 4.49E+03 | 6.59E+03 |
| **1681** | F1RZR9 | Chloride intracellular channel protein | CLIC2 | 2.64E+05 | 0.00E+00 | 2.64E+05 | 0.00E+00 | 1.65E+04 | 0.00E+00 | 1.65E+04 | 0.00E+00 |
| **1682** | F1S4X9 | Glutathione synthetase | GSS | 4.85E+05 | 4.85E+05 | 0.00E+00 | 0.00E+00 | 1.62E+04 | 1.62E+04 | 0.00E+00 | 0.00E+00 |
| **1683** | A0A087X0A8 | Collagen alpha-1(XII) chain | COL12A1 | 1.71E+06 | 1.41E+06 | 0.00E+00 | 3.01E+05 | 1.62E+04 | 1.33E+04 | 0.00E+00 | 2.84E+03 |
| **1684** | Q3YJM5 | cGMP-dependent protein kinase | Prkg1 | 6.56E+05 | 4.26E+05 | 9.21E+04 | 1.38E+05 | 1.60E+04 | 1.04E+04 | 2.25E+03 | 3.37E+03 |
| **1685** | O46409 | Apolipoprotein A-IV | APOA4 | 4.67E+05 | 0.00E+00 | 4.67E+05 | 0.00E+00 | 1.56E+04 | 0.00E+00 | 1.56E+04 | 0.00E+00 |
| **1686** | F1RRY6 | Uncharacterized protein | PTGES2 | 3.75E+05 | 3.75E+05 | 0.00E+00 | 0.00E+00 | 1.44E+04 | 1.44E+04 | 0.00E+00 | 0.00E+00 |
| **1687** | F1SAY0 | Uncharacterized protein (Fragment) | PTGFRN | 7.50E+05 | 0.00E+00 | 0.00E+00 | 7.50E+05 | 1.44E+04 | 0.00E+00 | 0.00E+00 | 1.44E+04 |
| **1688** | Q14204 | Cytoplasmic dynein 1 heavy chain 1 | DYNC1H1 | 4.04E+06 | 1.55E+06 | 2.49E+06 | 0.00E+00 | 1.42E+04 | 5.42E+03 | 8.74E+03 | 0.00E+00 |
| **1689** | D9YZV5 | Tropomyosin 1 (Alpha) isoform 4 | TPM1 | 3.17E+05 | 3.17E+05 | 0.00E+00 | 0.00E+00 | 1.38E+04 | 1.38E+04 | 0.00E+00 | 0.00E+00 |
| **1690** | F1SJ68 | Alpha-mannosidase | MAN2C1 | 7.01E+05 | 4.53E+05 | 0.00E+00 | 2.48E+05 | 1.37E+04 | 8.88E+03 | 0.00E+00 | 4.87E+03 |
| **1691** | F1SKJ1 | Uncharacterized protein | MYH9 | 1.69E+06 | 1.41E+06 | 0.00E+00 | 2.79E+05 | 1.37E+04 | 1.14E+04 | 0.00E+00 | 2.25E+03 |
| **1692** | X6RJS7 | Lys-63-specific deubiquitinase BRCC36 (Fragment) | BRCC3 | 1.62E+05 | 0.00E+00 | 0.00E+00 | 1.62E+05 | 1.35E+04 | 0.00E+00 | 0.00E+00 | 1.35E+04 |
| **1693** | I3LLW7 | Uncharacterized protein | | 6.00E+05 | 2.88E+05 | 0.00E+00 | 3.12E+05 | 1.30E+04 | 6.25E+03 | 0.00E+00 | 6.78E+03 |
| **1694** | F1RYU9 | Uncharacterized protein | USO1 | 6.64E+05 | 6.64E+05 | 0.00E+00 | 0.00E+00 | 1.30E+04 | 1.30E+04 | 0.00E+00 | 0.00E+00 |
| **1695** | B3KMZ6 | cDNA FLJ13058 fis, clone NT2RP3001587, highly similar to Ubiquitin-like 1-activating enzyme E1B | | 4.56E+05 | 0.00E+00 | 4.56E+05 | 0.00E+00 | 1.30E+04 | 0.00E+00 | 1.30E+04 | 0.00E+00 |
| **1696** | E7ENL6 | Collagen alpha-3(VI) chain | COL6A3 | 2.16E+06 | 2.85E+05 | 6.55E+05 | 1.22E+06 | 1.30E+04 | 1.71E+03 | 3.94E+03 | 7.34E+03 |
| **1697** | Q860V2 | MHC class I antigen | SLA-1 | 2.33E+05 | 0.00E+00 | 2.33E+05 | 0.00E+00 | 1.29E+04 | 0.00E+00 | 1.29E+04 | 0.00E+00 |
| **1698** | I3LRH6 | Uncharacterized protein | TENM3 | 1.56E+06 | 0.00E+00 | 0.00E+00 | 1.56E+06 | 1.25E+04 | 0.00E+00 | 0.00E+00 | 1.25E+04 |
| **1699** | J9T9K7 | CD163 protein | CD163 | 7.63E+05 | 6.09E+05 | 1.54E+05 | 0.00E+00 | 1.25E+04 | 9.99E+03 | 2.52E+03 | 0.00E+00 |
| **1700** | F1SG42 | 10-formyltetrahydrofolate dehydrogenase | ALDH1L2 | 8.36E+05 | 6.48E+05 | 0.00E+00 | 1.88E+05 | 1.25E+04 | 9.67E+03 | 0.00E+00 | 2.81E+03 |
| **1701** | Q6YSS6 | FYVE and coiled-coil domain containing 1 (Fragment) | FYCO1 | 6.44E+05 | 0.00E+00 | 0.00E+00 | 6.44E+05 | 1.24E+04 | 0.00E+00 | 0.00E+00 | 1.24E+04 |
| **1702** | I3LDA5 | Uncharacterized protein | EHD4 | 2.82E+05 | 0.00E+00 | 2.82E+05 | 0.00E+00 | 1.23E+04 | 0.00E+00 | 1.23E+04 | 0.00E+00 |
| **1703** | B3KPA6 | Acyl-Coenzyme A dehydrogenase, very long chain, isoform CRA | ACADVL | 4.36E+05 | 0.00E+00 | 1.89E+05 | 2.47E+05 | 1.18E+04 | 0.00E+00 | 5.10E+03 | 6.68E+03 |
| **1704** | B7ZA00 | cDNA, FLJ79014, highly similar to cAMP-dependent protein kinase, beta-catalytic subunit (EC 2.7.11.11) | | 2.00E+05 | 0.00E+00 | 2.00E+05 | 0.00E+00 | 1.18E+04 | 0.00E+00 | 1.18E+04 | 0.00E+00 |
| **1705** | F1RNS9 | Uncharacterized protein | PTCD1 | 4.86E+05 | 0.00E+00 | 0.00E+00 | 4.86E+05 | 1.16E+04 | 0.00E+00 | 0.00E+00 | 1.16E+04 |
| **1706** | H7C0E5 | Zinc finger protein ZPR1 (Fragment) | ZPR1 | 2.50E+05 | 0.00E+00 | 0.00E+00 | 2.50E+05 | 1.09E+04 | 0.00E+00 | 0.00E+00 | 1.09E+04 |
| **1707** | Q8TCR7 | Putative uncharacterized protein DKFZp761K0922 (Fragment) | DKFZp761K0922 | 2.81E+05 | 2.81E+05 | 0.00E+00 | 0.00E+00 | 1.00E+04 | 1.00E+04 | 0.00E+00 | 0.00E+00 |
| **1708** | F6SDV2 | Tubulointerstitial nephritis antigen-like | TINAGL1 | 1.90E+05 | 1.90E+05 | 0.00E+00 | 0.00E+00 | 1.00E+04 | 1.00E+04 | 0.00E+00 | 0.00E+00 |
| **1709** | B7Z8B3 | Peroxisomal biogenesis factor 19 | PEX19 | 1.19E+05 | 1.19E+05 | 0.00E+00 | 0.00E+00 | 9.93E+03 | 9.93E+03 | 0.00E+00 | 0.00E+00 |
| **1710** | F2Z5J8 | Eukaryotic translation initiation factor 2 subunit 1 | EIF2S1 | 2.14E+05 | 2.14E+05 | 0.00E+00 | 0.00E+00 | 9.74E+03 | 9.74E+03 | 0.00E+00 | 0.00E+00 |
| **1711** | F1RQW6 | Complement factor B | CFB | 3.94E+05 | 0.00E+00 | 0.00E+00 | 3.94E+05 | 8.96E+03 | 0.00E+00 | 0.00E+00 | 8.96E+03 |
| **1712** | Q8NDE0 | Putative uncharacterized protein DKFZp547P162 (Fragment) | DKFZp547P162 | 1.51E+05 | 0.00E+00 | 0.00E+00 | 1.51E+05 | 8.90E+03 | 0.00E+00 | 0.00E+00 | 8.90E+03 |
| **1713** | F1RRK9 | Uncharacterized protein | XPO5 | 4.88E+05 | 0.00E+00 | 2.80E+05 | 2.08E+05 | 8.87E+03 | 0.00E+00 | 5.09E+03 | 3.78E+03 |
| **1714** | Q01813 | ATP-dependent 6-phosphofructokinase, platelet type | PFKP | 3.21E+05 | 0.00E+00 | 3.21E+05 | 0.00E+00 | 8.69E+03 | 0.00E+00 | 8.69E+03 | 0.00E+00 |
| **1715** | Q3T052 | Inter-alpha (Globulin) inhibitor H4 | | 4.18E+05 | 0.00E+00 | 0.00E+00 | 4.18E+05 | 8.53E+03 | 0.00E+00 | 0.00E+00 | 8.53E+03 |
| **1716** | I3LCX2 | Uncharacterized protein | | 2.55E+05 | 2.55E+05 | 0.00E+00 | 0.00E+00 | 8.51E+03 | 8.51E+03 | 0.00E+00 | 0.00E+00 |
| **1717** | F1S682 | Sulfhydryl oxidase | QSOX1 | 4.32E+05 | 0.00E+00 | 4.32E+05 | 0.00E+00 | 8.47E+03 | 0.00E+00 | 8.47E+03 | 0.00E+00 |
| **1718** | F1SRK6 | Endoplasmin | HSP90B1 | 4.07E+05 | 4.07E+05 | 0.00E+00 | 0.00E+00 | 8.47E+03 | 8.47E+03 | 0.00E+00 | 0.00E+00 |
| **1719** | P35908 | Keratin, type II cytoskeletal 2 epidermal | KRT2 Keratin, type II | 3.54E+05 | 0.00E+00 | 0.00E+00 | 3.54E+05 | 8.43E+03 | 0.00E+00 | 0.00E+00 | 8.43E+03 |
| **1720** | I3LUE8 | Uncharacterized protein | | 2.30E+05 | 1.60E+05 | 0.00E+00 | 7.04E+04 | 7.95E+03 | 5.52E+03 | 0.00E+00 | 2.43E+03 |
| **1721** | F6Q4L6 | Uncharacterized protein | SLC25A24 | 2.03E+05 | 0.00E+00 | 0.00E+00 | 2.03E+05 | 7.80E+03 | 0.00E+00 | 0.00E+00 | 7.80E+03 |
| **1722** | F1RRW5 | Uncharacterized protein | ACE | 5.40E+05 | 1.05E+05 | 3.48E+05 | 8.70E+04 | 7.72E+03 | 1.50E+03 | 4.98E+03 | 1.24E+03 |
| **1723** | F1SHN7 | Uncharacterized protein | LRRK2 | 1.12E+06 | 0.00E+00 | 0.00E+00 | 1.12E+06 | 7.35E+03 | 0.00E+00 | 0.00E+00 | 7.35E+03 |
| **1724** | K9J4W0 | Inter-alpha-trypsin inhibitor heavy chain H5 | ITIH5 | 3.67E+05 | 0.00E+00 | 3.67E+05 | 0.00E+00 | 6.93E+03 | 0.00E+00 | 6.93E+03 | 0.00E+00 |
| **1725** | I3L8Q0 | Uncharacterized protein | | 8.00E+05 | 8.00E+05 | 0.00E+00 | 0.00E+00 | 6.78E+03 | 6.78E+03 | 0.00E+00 | 0.00E+00 |
| **1726** | F1SGT3 | Uncharacterized protein | PDHX | 2.02E+05 | 2.02E+05 | 0.00E+00 | 0.00E+00 | 6.73E+03 | 6.73E+03 | 0.00E+00 | 0.00E+00 |
| **1727** | Q9TV61 | Myosin-1 | MYH1 | 8.41E+05 | 0.00E+00 | 8.41E+05 | 0.00E+00 | 6.67E+03 | 0.00E+00 | 6.67E+03 | 0.00E+00 |
| **1728** | B4DSW9 | cDNA FLJ59415, highly similar to Beta-catenin | | 2.78E+05 | 0.00E+00 | 2.78E+05 | 0.00E+00 | 6.61E+03 | 0.00E+00 | 6.61E+03 | 0.00E+00 |
| **1729** | F1SC82 | Uncharacterized protein | | 8.69E+05 | 0.00E+00 | 6.56E+05 | 2.12E+05 | 6.58E+03 | 0.00E+00 | 4.97E+03 | 1.61E+03 |
| **1730** | F1RL04 | Uncharacterized protein | PPM1F | 1.36E+05 | 0.00E+00 | 0.00E+00 | 1.36E+05 | 6.48E+03 | 0.00E+00 | 0.00E+00 | 6.48E+03 |
| **1731** | F2Z5Q5 | Uncharacterized protein | RRAGA | 8.92E+04 | 8.92E+04 | 0.00E+00 | 0.00E+00 | 5.25E+03 | 5.25E+03 | 0.00E+00 | 0.00E+00 |
| **1732** | P04258 | Similar to Collagen alpha 1(III) chain | | 3.69E+05 | 0.00E+00 | 3.69E+05 | 0.00E+00 | 4.55E+03 | 0.00E+00 | 4.55E+03 | 0.00E+00 |
| **1733** | Q15149 | Plectin | PLEC | 1.53E+06 | 1.17E+06 | 0.00E+00 | 3.55E+05 | 4.46E+03 | 3.43E+03 | 0.00E+00 | 1.04E+03 |
| **1734** | I3LGC2 | Uncharacterized protein | RRBP1 | 4.98E+05 | 0.00E+00 | 4.98E+05 | 0.00E+00 | 4.26E+03 | 0.00E+00 | 4.26E+03 | 0.00E+00 |
| **1735** | F1S138 | Glutamyl aminopeptidase | ENPEP | 1.84E+05 | 1.84E+05 | 0.00E+00 | 0.00E+00 | 3.69E+03 | 3.69E+03 | 0.00E+00 | 0.00E+00 |
| **1736** | D3DPG0 | Titin, isoform CRA | TTN | 6.99E+06 | 2.85E+06 | 1.87E+06 | 2.28E+06 | 2.98E+03 | 1.22E+03 | 7.96E+02 | 9.71E+02 |
| **1737** | F1SME1 | Complement C5a anaphylatoxin | C5 | 2.51E+05 | 0.00E+00 | 0.00E+00 | 2.51E+05 | 2.75E+03 | 0.00E+00 | 0.00E+00 | 2.75E+03 |
| **1738** | A0A087WV90 | Dystrophin | DMD | 5.51E+05 | 1.06E+05 | 2.79E+05 | 1.66E+05 | 2.38E+03 | 4.56E+02 | 1.20E+03 | 7.16E+02 |
| **1739** | A5A8V9 | Valyl-tRNA synthetase 2 | VARS2 | 1.33E+05 | 0.00E+00 | 0.00E+00 | 1.33E+05 | 1.95E+03 | 0.00E+00 | 0.00E+00 | 1.95E+03 |
| **1740** | Q29021 | Apolipoprotein B (Fragment) | APOB | 3.09E+05 | 0.00E+00 | 1.42E+05 | 1.67E+05 | 1.94E+03 | 0.00E+00 | 8.95E+02 | 1.05E+03 |
| **1741** | I3LC73 | Uncharacterized protein | | 1.31E+05 | 0.00E+00 | 0.00E+00 | 1.31E+05 | 1.87E+03 | 0.00E+00 | 0.00E+00 | 1.87E+03 |
| **1742** | E7ENN3 | Nesprin-1 | SYNE1 | 8.43E+05 | 0.00E+00 | 1.46E+05 | 6.97E+05 | 1.57E+03 | 0.00E+00 | 2.71E+02 | 1.30E+03 |

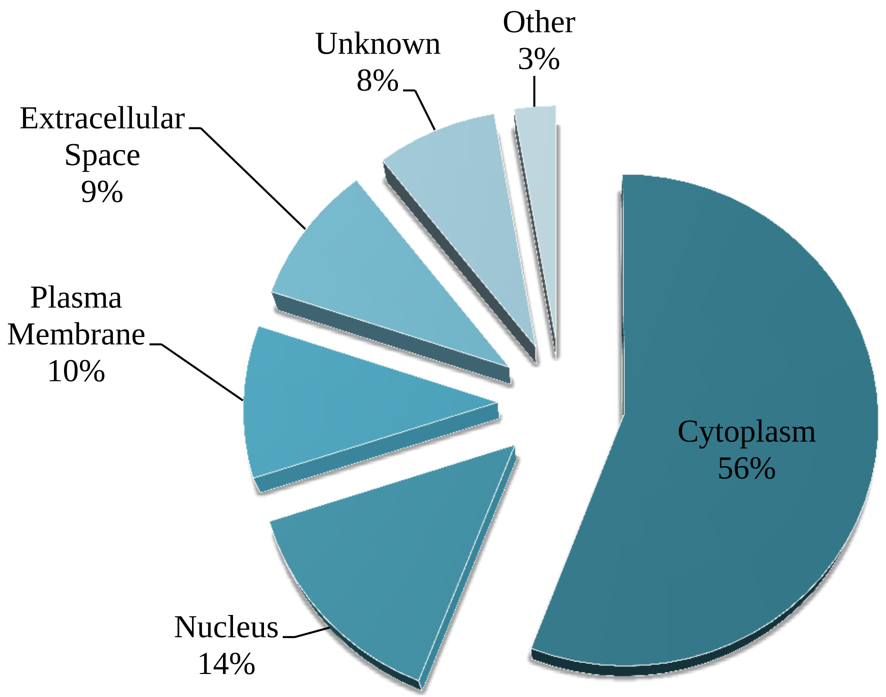


**Supplementary Figure S1**

**The major Gene Ontology Cellular Component terms (GOCCs) of the proteins identified from the porcine sPCA.** A large majority of the identified proteins are from the cytoplasm, while 14 % and 10 % are nuclear and plasma membrane proteins. Only a small percentage of the sPCA proteins are localized in the extracellular space, while the remaining 11 % corresponded to other unknown cellular compartments.

**Supplementary Table S2:**
**Complete list of the GOBP of proteins identified from the porcine sPCA analysed employing DAVID functional analysis tool.**

| No. | Term | Count | % | P-Value | List Total | Pop Hits | Pop Total | Fold Enrichment | Bonferroni | Benjamini | FDR |
|---|---|---|---|---|---|---|---|---|---|---|---|
| 1 | GO:0006091~generation of precursor metabolites and energy | 112 | 0.4105722 | 1.43E-40 | 1185 | 313 | 13528 | 4.0849705 | 5.19E-37 | 5.19E-37 | 2.63E-37 |
| 2 | GO:0015980~energy derivation by oxidation of organic compounds | 58 | 0.2126178 | 4.55E-24 | 1185 | 144 | 13528 | 4.5981247 | 1.65E-20 | 8.25E-21 | 8.36E-21 |
| 3 | GO:0045333~cellular respiration | 46 | 0.1686279 | 1.15E-22 | 1185 | 97 | 13528 | 5.4137892 | 4.18E-19 | 1.39E-19 | 2.12E-19 |
| 4 | GO:0055114~oxidation reduction | 133 | 0.4875545 | 8.33E-22 | 1185 | 639 | 13528 | 2.3761072 | 3.03E-18 | 7.56E-19 | 1.53E-18 |
| 5 | GO:0006414~translational elongation | 44 | 0.1612962 | 6.24E-20 | 1185 | 101 | 13528 | 4.9733216 | 2.26E-16 | 4.53E-17 | 1.15E-16 |
| 6 | GO:0006007~glucose catabolic process | 30 | 0.1099747 | 3.43E-16 | 1185 | 58 | 13528 | 5.904845 | 1.21E-12 | 2.02E-13 | 6.11E-13 |
| 7 | GO:0006119~oxidative phosphorylation | 38 | 0.1393013 | 2.98E-15 | 1185 | 98 | 13528 | 4.4266253 | 1.09E-11 | 1.55E-12 | 5.52E-12 |
| 8 | GO:0022900~electron transport chain | 41 | 0.1502988 | 4.12E-15 | 1185 | 114 | 13528 | 4.1057665 | 1.49E-11 | 1.86E-12 | 7.55E-12 |
| 9 | GO:0046365~monosaccharide catabolic process | 32 | 0.1173064 | 4.52E-15 | 1185 | 71 | 13528 | 5.1452547 | 1.65E-11 | 1.84E-12 | 8.37E-12 |
| 10 | GO:0044275~cellular carbohydrate catabolic process | 35 | 0.1283038 | 5.18E-15 | 1185 | 85 | 13528 | 4.7007198 | 1.89E-11 | 1.89E-12 | 9.59E-12 |
| 11 | GO:0006006~glucose metabolic process | 48 | 0.1759595 | 5.90E-15 | 1185 | 153 | 13528 | 3.5815008 | 2.14E-11 | 1.94E-12 | 1.08E-11 |
| 12 | GO:0046164~alcohol catabolic process | 34 | 0.124638 | 6.92E-15 | 1185 | 81 | 13528 | 4.7919154 | 2.50E-11 | 2.08E-12 | 1.27E-11 |
| 13 | GO:0006096~glycolysis | 25 | 0.0916456 | 7.35E-14 | 1185 | 47 | 13528 | 6.0723584 | 2.67E-10 | 2.05E-11 | 1.35E-10 |
| 14 | GO:0022904~respiratory electron transport chain | 29 | 0.1063089 | 8.96E-14 | 1185 | 64 | 13528 | 5.1728903 | 3.25E-10 | 2.32E-11 | 1.65E-10 |
| 15 | GO:0019320~hexose catabolic process | 30 | 0.1099747 | 1.15E-13 | 1185 | 69 | 13528 | 4.9634929 | 4.18E-10 | 2.79E-11 | 2.12E-10 |
| 16 | GO:0005996~monosaccharide metabolic process | 57 | 0.2089519 | 1.85E-13 | 1185 | 222 | 13528 | 2.9311438 | 6.72E-10 | 4.20E-11 | 3.41E-10 |
| 17 | GO:0007010~cytoskeleton organization | 87 | 0.3189266 | 3.47E-13 | 1185 | 436 | 13528 | 2.27797 | 1.26E-09 | 7.41E-11 | 6.38E-10 |
| 18 | GO:0006457~protein folding | 49 | 0.1796254 | 5.70E-13 | 1185 | 177 | 13528 | 3.1603709 | 2.07E-09 | 1.15E-10 | 1.05E-09 |
| 19 | GO:0030029~actin filament-based process | 59 | 0.2162836 | 5.95E-13 | 1185 | 241 | 13528 | 2.7947966 | 2.16E-09 | 1.14E-10 | 1.09E-09 |
| 20 | GO:0016052~carbohydrate catabolic process | 37 | 0.1356355 | 7.99E-13 | 1185 | 109 | 13528 | 3.8751674 | 2.90E-09 | 1.45E-10 | 1.47E-09 |
| 21 | GO:0042775~mitochondrial ATP synthesis coupled electron transport | 26 | 0.0953114 | 1.10E-12 | 1185 | 56 | 13528 | 5.3003014 | 3.98E-09 | 1.89E-10 | 2.01E-09 |
| 22 | GO:0042773~ATP synthesis coupled electron transport | 26 | 0.0953114 | 1.10E-12 | 1185 | 56 | 13528 | 5.3003014 | 3.98E-09 | 1.89E-10 | 2.01E-09 |
| 23 | GO:0031400~negative regulation of protein modification process | 38 | 0.1393013 | 3.06E-12 | 1185 | 119 | 13528 | 3.6454562 | 1.11E-08 | 5.05E-10 | 5.63E-09 |
| 24 | GO:0019318~hexose metabolic process | 50 | 0.1832912 | 3.84E-12 | 1185 | 192 | 13528 | 2.9729255 | 1.39E-08 | 6.06E-10 | 7.06E-09 |
| 25 | GO:0006120~mitochondrial electron transport, NADH to ubiquinone | 22 | 0.0806481 | 4.61E-12 | 1185 | 42 | 13528 | 5.9798272 | 1.67E-08 | 6.97E-10 | 8.47E-09 |
| 26 | GO:0030036~actin cytoskeleton organization | 55 | 0.2016203 | 5.12E-12 | 1185 | 226 | 13528 | 2.7782383 | 1.86E-08 | 7.44E-10 | 9.42E-09 |
| 27 | GO:0065003~macromolecular complex assembly | 113 | 0.4142381 | 5.37E-12 | 1185 | 665 | 13528 | 1.9398674 | 1.95E-08 | 7.50E-10 | 9.88E-09 |
| 28 | GO:0051187~cofactor catabolic process | 19 | 0.0696506 | 5.50E-12 | 1185 | 31 | 13528 | 6.9969239 | 1.99E-08 | 7.39E-10 | 1.01E-08 |
| 29 | GO:0009060~aerobic respiration | 20 | 0.0733165 | 7.21E-12 | 1185 | 35 | 13528 | 6.5234479 | 2.62E-08 | 9.35E-10 | 1.33E-08 |
| 30 | GO:0051443~positive regulation of ubiquitin-protein ligase activity | 28 | 0.1026431 | 9.34E-12 | 1185 | 70 | 13528 | 4.5664135 | 3.39E-08 | 1.17E-09 | 1.72E-08 |
| 31 | GO:0006732~coenzyme metabolic process | 43 | 0.1576304 | 1.05E-11 | 1185 | 153 | 13528 | 3.2084278 | 3.80E-08 | 1.27E-09 | 1.93E-08 |
| 32 | GO:0009109~coenzyme catabolic process | 17 | 0.062319 | 2.46E-11 | 1185 | 26 | 13528 | 7.4643298 | 8.92E-08 | 2.88E-09 | 4.52E-08 |
| 33 | GO:0051186~cofactor metabolic process | 49 | 0.1796254 | 2.56E-11 | 1185 | 195 | 13528 | 2.8686444 | 9.31E-08 | 2.91E-09 | 4.72E-08 |
| 34 | GO:0051438~regulation of ubiquitin-protein ligase activity | 29 | 0.1063089 | 2.93E-11 | 1185 | 78 | 13528 | 4.2444228 | 1.06E-07 | 3.22E-09 | 5.39E-08 |
| 35 | GO:0051351~positive regulation of ligase activity | 28 | 0.1026431 | 2.96E-11 | 1185 | 73 | 13528 | 4.3787527 | 1.07E-07 | 3.16E-09 | 5.44E-08 |
| 36 | GO:0046356~acetyl-CoA catabolic process | 16 | 0.0586532 | 3.11E-11 | 1185 | 23 | 13528 | 7.9415887 | 1.13E-07 | 3.23E-09 | 5.73E-08 |
| 37 | GO:0006099~tricarboxylic acid cycle | 16 | 0.0586532 | 3.11E-11 | 1185 | 23 | 13528 | 7.9415887 | 1.13E-07 | 3.23E-09 | 5.73E-08 |
| 38 | GO:0043933~macromolecular complex subunit organization | 116 | 0.4252355 | 3.58E-11 | 1185 | 710 | 13528 | 1.8651548 | 1.30E-07 | 3.61E-09 | 6.58E-08 |
| 39 | GO:0031398~positive regulation of protein ubiquitination | 30 | 0.1099747 | 3.93E-11 | 1185 | 84 | 13528 | 4.0771549 | 1.43E-07 | 3.86E-09 | 7.23E-08 |
| 40 | GO:0010035~response to inorganic substance | 50 | 0.1832912 | 4.93E-11 | 1185 | 205 | 13528 | 2.7843985 | 1.79E-07 | 4.71E-09 | 9.06E-08 |
| 41 | GO:0031145~anaphase-promoting complex-dependent proteasomal ubiquitin-dependent protein catabolic process | 26 | 0.0953114 | 5.91E-11 | 1185 | 65 | 13528 | 4.5664135 | 2.15E-07 | 5.50E-09 | 1.09E-07 |
| 42 | GO:0051436~negative regulation of ubiquitin-protein ligase activity during mitotic cell cycle | 26 | 0.0953114 | 5.91E-11 | 1185 | 65 | 13528 | 4.5664135 | 2.15E-07 | 5.50E-09 | 1.09E-07 |
| 43 | GO:0032269~negative regulation of cellular protein metabolic process | 46 | 0.1686279 | 6.17E-11 | 1185 | 180 | 13528 | 2.9174308 | 2.24E-07 | 5.60E-09 | 1.13E-07 |
| 44 | GO:0051248~negative regulation of protein metabolic process | 47 | 0.1722937 | 6.84E-11 | 1185 | 187 | 13528 | 2.8692705 | 2.48E-07 | 6.05E-09 | 1.26E-07 |
| 45 | GO:0006084~acetyl-CoA metabolic process | 18 | 0.0659848 | 7.54E-11 | 1185 | 31 | 13528 | 6.6286648 | 2.74E-07 | 6.51E-09 | 1.39E-07 |
| 46 | GO:0051340~regulation of ligase activity | 29 | 0.1063089 | 8.24E-11 | 1185 | 81 | 13528 | 4.087222 | 2.99E-07 | 6.96E-09 | 1.52E-07 |
| 47 | GO:0051439~regulation of ubiquitin-protein ligase activity during mitotic cell cycle | 27 | 0.0989772 | 8.90E-11 | 1185 | 71 | 13528 | 4.3413086 | 3.23E-07 | 7.35E-09 | 1.64E-07 |
| 48 | GO:0051444~negative regulation of ubiquitin-protein ligase activity | 26 | 0.0953114 | 1.28E-10 | 1185 | 67 | 13528 | 4.4301027 | 4.64E-07 | 1.03E-08 | 2.35E-07 |
| 49 | GO:0051352~negative regulation of ligase activity | 26 | 0.0953114 | 1.28E-10 | 1185 | 67 | 13528 | 4.4301027 | 4.64E-07 | 1.03E-08 | 2.35E-07 |
| 50 | GO:0051437~positive regulation of ubiquitin-protein ligase activity during mitotic cell cycle | 26 | 0.0953114 | 1.86E-10 | 1185 | 68 | 13528 | 4.3649541 | 6.74E-07 | 1.46E-08 | 3.41E-07 |
| 51 | GO:0031396~regulation of protein ubiquitination | 32 | 0.1173064 | 1.92E-10 | 1185 | 100 | 13528 | 3.6531308 | 6.99E-07 | 1.49E-08 | 3.54E-07 |
| 52 | GO:0009259~ribonucleotide metabolic process | 39 | 0.1429671 | 6.78E-10 | 1185 | 147 | 13528 | 3.0287436 | 2.46E-06 | 5.13E-08 | 1.25E-06 |
| 53 | GO:0006412~translation | 65 | 0.2382785 | 9.38E-10 | 1185 | 331 | 13528 | 2.2418193 | 3.40E-06 | 6.95E-08 | 1.72E-06 |
| 54 | GO:0009150~purine ribonucleotide metabolic process | 37 | 0.1356355 | 1.45E-09 | 1185 | 138 | 13528 | 3.0608206 | 5.28E-06 | 1.06E-07 | 2.68E-06 |
| 55 | GO:0031397~negative regulation of protein ubiquitination | 26 | 0.0953114 | 1.47E-09 | 1185 | 74 | 13528 | 4.0110389 | 5.33E-06 | 1.05E-07 | 2.70E-06 |
| 56 | GO:0010498~proteasomal protein catabolic process | 31 | 0.1136405 | 1.56E-09 | 1185 | 102 | 13528 | 3.4695789 | 5.65E-06 | 1.09E-07 | 2.86E-06 |
| 57 | GO:0043161~proteasomal ubiquitin-dependent protein catabolic process | 31 | 0.1136405 | 1.56E-09 | 1185 | 102 | 13528 | 3.4695789 | 5.65E-06 | 1.09E-07 | 2.86E-06 |
| 58 | GO:0006936~muscle contraction | 38 | 0.1393013 | 8.20E-09 | 1185 | 153 | 13528 | 2.8353548 | 2.98E-05 | 5.62E-07 | 1.51E-05 |
| 59 | GO:0002526~acute inflammatory response | 29 | 0.1063089 | 1.11E-08 | 1185 | 98 | 13528 | 3.3782141 | 4.03E-05 | 7.46E-07 | 2.04E-05 |
| 60 | GO:0003012~muscle system process | 40 | 0.1466329 | 1.11E-08 | 1185 | 168 | 13528 | 2.7181033 | 4.05E-05 | 7.36E-07 | 2.05E-05 |
| 61 | GO:0010033~response to organic substance | 109 | 0.3995748 | 1.35E-08 | 1185 | 721 | 13528 | 1.7258636 | 4.92E-05 | 8.78E-07 | 2.49E-05 |
| 62 | GO:0070271~protein complex biogenesis | 83 | 0.3042634 | 2.43E-08 | 1185 | 505 | 13528 | 1.8762986 | 8.82E-05 | 1.55E-06 | 4.47E-05 |
| 63 | GO:0006461~protein complex assembly | 83 | 0.3042634 | 2.43E-08 | 1185 | 505 | 13528 | 1.8762986 | 8.82E-05 | 1.55E-06 | 4.47E-05 |
| 64 | GO:0032268~regulation of cellular protein metabolic process | 79 | 0.2896001 | 3.02E-08 | 1185 | 474 | 13528 | 1.9026723 | 1.10E-04 | 1.89E-06 | 5.56E-05 |
| 65 | GO:0051259~protein oligomerization | 40 | 0.1466329 | 3.12E-08 | 1185 | 174 | 13528 | 2.6243756 | 1.13E-04 | 1.92E-06 | 5.74E-05 |
| 66 | GO:0045454~cell redox homeostasis | 22 | 0.0806481 | 3.94E-08 | 1185 | 63 | 13528 | 3.9865515 | 1.43E-04 | 2.38E-06 | 7.24E-05 |
| 67 | GO:0009152~purine ribonucleotide biosynthetic process | 31 | 0.1136405 | 5.14E-08 | 1185 | 117 | 13528 | 3.0247611 | 1.86E-04 | 3.06E-06 | 9.45E-05 |
| 68 | GO:0034622~cellular macromolecular complex assembly | 59 | 0.2162836 | 5.16E-08 | 1185 | 318 | 13528 | 2.1180692 | 1.87E-04 | 3.02E-06 | 9.49E-05 |
| 69 | GO:0009260~ribonucleotide biosynthetic process | 32 | 0.1173064 | 5.82E-08 | 1185 | 124 | 13528 | 2.9460732 | 2.11E-04 | 3.35E-06 | 1.07E-04 |
| 70 | GO:0051260~protein homooligomerization | 27 | 0.0989772 | 9.64E-08 | 1185 | 95 | 13528 | 3.244557 | 3.50E-04 | 5.47E-06 | 1.77E-04 |
| 71 | GO:0044271~nitrogen compound biosynthetic process | 59 | 0.2162836 | 1.13E-07 | 1185 | 325 | 13528 | 2.0724492 | 4.11E-04 | 6.32E-06 | 2.08E-04 |
| 72 | GO:0009144~purine nucleoside triphosphate metabolic process | 31 | 0.1136405 | 1.41E-07 | 1185 | 122 | 13528 | 2.9007955 | 5.11E-04 | 7.75E-06 | 2.59E-04 |
| 73 | GO:0051493~regulation of cytoskeleton organization | 33 | 0.1209722 | 1.64E-07 | 1185 | 136 | 13528 | 2.770067 | 5.95E-04 | 8.88E-06 | 3.01E-04 |
| 74 | GO:0007264~small GTPase mediated signal transduction | 56 | 0.2052861 | 1.69E-07 | 1185 | 305 | 13528 | 2.0960587 | 6.14E-04 | 9.03E-06 | 3.11E-04 |
| 75 | GO:0006081~cellular aldehyde metabolic process | 14 | 0.0513215 | 1.78E-07 | 1185 | 28 | 13528 | 5.7080169 | 6.45E-04 | 9.35E-06 | 3.27E-04 |
| 76 | GO:0006979~response to oxidative stress | 37 | 0.1356355 | 1.83E-07 | 1185 | 164 | 13528 | 2.5755686 | 6.65E-04 | 9.50E-06 | 3.37E-04 |
| 77 | GO:0009205~purine ribonucleoside triphosphate metabolic process | 30 | 0.1099747 | 1.89E-07 | 1185 | 117 | 13528 | 2.9271881 | 6.85E-04 | 9.66E-06 | 3.47E-04 |
| 78 | GO:0006163~purine nucleotide metabolic process | 40 | 0.1466329 | 2.06E-07 | 1185 | 186 | 13528 | 2.455061 | 7.48E-04 | 1.04E-05 | 3.79E-04 |
| 79 | GO:0009141~nucleoside triphosphate metabolic process | 32 | 0.1173064 | 2.22E-07 | 1185 | 131 | 13528 | 2.7886495 | 8.07E-04 | 1.11E-05 | 4.09E-04 |
| 80 | GO:0046034~ATP metabolic process | 28 | 0.1026431 | 2.23E-07 | 1185 | 105 | 13528 | 3.0442757 | 8.08E-04 | 1.09E-05 | 4.09E-04 |
| 81 | GO:0009199~ribonucleoside triphosphate metabolic process | 30 | 0.1099747 | 2.30E-07 | 1185 | 118 | 13528 | 2.9023815 | 8.34E-04 | 1.11E-05 | 4.23E-04 |
| 82 | GO:0006575~cellular amino acid derivative metabolic process | 37 | 0.1356355 | 2.52E-07 | 1185 | 166 | 13528 | 2.5445376 | 9.13E-04 | 1.20E-05 | 4.63E-04 |
| 83 | GO:0034621~cellular macromolecular complex subunit organization | 62 | 0.2272811 | 2.62E-07 | 1185 | 357 | 13528 | 1.9826165 | 9.52E-04 | 1.24E-05 | 4.83E-04 |
| 84 | GO:0043648~dicarboxylic acid metabolic process | 15 | 0.0549874 | 3.74E-07 | 1185 | 34 | 13528 | 5.0364855 | 1.36E-03 | 1.74E-05 | 6.89E-04 |
| 85 | GO:0019321~pentose metabolic process | 9 | 0.0329924 | 4.37E-07 | 1185 | 11 | 13528 | 9.3403913 | 1.58E-03 | 2.01E-05 | 8.03E-04 |
| 86 | GO:0016044~membrane organization | 64 | 0.2346127 | 5.52E-07 | 1185 | 381 | 13528 | 1.917654 | 2.00E-03 | 2.51E-05 | 1.02E-03 |
| 87 | GO:0019725~cellular homeostasis | 74 | 0.2712709 | 6.05E-07 | 1185 | 466 | 13528 | 1.8128466 | 2.20E-03 | 2.71E-05 | 1.11E-03 |
| 88 | GO:0030198~extracellular matrix organization | 27 | 0.0989772 | 6.72E-07 | 1185 | 104 | 13528 | 2.963778 | 2.44E-03 | 2.98E-05 | 1.24E-03 |
| 89 | GO:0042542~response to hydrogen peroxide | 19 | 0.0696506 | 6.78E-07 | 1185 | 56 | 13528 | 3.8732972 | 2.46E-03 | 2.97E-05 | 1.25E-03 |
| 90 | GO:0031589~cell-substrate adhesion | 26 | 0.0953114 | 7.30E-07 | 1185 | 98 | 13528 | 3.0287436 | 2.65E-03 | 3.15E-05 | 1.34E-03 |
| 91 | GO:0009206~purine ribonucleoside triphosphate biosynthetic process | 26 | 0.0953114 | 7.30E-07 | 1185 | 98 | 13528 | 3.0287436 | 2.65E-03 | 3.15E-05 | 1.34E-03 |
| 92 | GO:0009201~ribonucleoside triphosphate biosynthetic process | 26 | 0.0953114 | 8.96E-07 | 1185 | 99 | 13528 | 2.9981503 | 3.25E-03 | 3.83E-05 | 1.65E-03 |
| 93 | GO:0009145~purine nucleoside triphosphate biosynthetic process | 26 | 0.0953114 | 8.96E-07 | 1185 | 99 | 13528 | 2.9981503 | 3.25E-03 | 3.83E-05 | 1.65E-03 |
| 94 | GO:0030832~regulation of actin filament length | 20 | 0.0733165 | 1.01E-06 | 1185 | 63 | 13528 | 3.6241377 | 3.65E-03 | 4.25E-05 | 1.85E-03 |
| 95 | GO:0000302~response to reactive oxygen species | 22 | 0.0806481 | 1.08E-06 | 1185 | 75 | 13528 | 3.3487032 | 3.91E-03 | 4.50E-05 | 1.99E-03 |
| 96 | GO:0006164~purine nucleotide biosynthetic process | 33 | 0.1209722 | 1.24E-06 | 1185 | 148 | 13528 | 2.545467 | 4.48E-03 | 5.11E-05 | 2.28E-03 |
| 97 | GO:0006090~pyruvate metabolic process | 16 | 0.0586532 | 1.27E-06 | 1185 | 42 | 13528 | 4.3489652 | 4.59E-03 | 5.17E-05 | 2.33E-03 |
| 98 | GO:0006956~complement activation | 16 | 0.0586532 | 1.27E-06 | 1185 | 42 | 13528 | 4.3489652 | 4.59E-03 | 5.17E-05 | 2.33E-03 |
| 99 | GO:0006518~peptide metabolic process | 18 | 0.0659848 | 1.43E-06 | 1185 | 53 | 13528 | 3.8771435 | 5.17E-03 | 5.76E-05 | 2.63E-03 |
| 100 | GO:0051129~negative regulation of cellular component organization | 32 | 0.1173064 | 1.46E-06 | 1185 | 142 | 13528 | 2.5726273 | 5.27E-03 | 5.80E-05 | 2.68E-03 |
| 101 | GO:0006754~ATP biosynthetic process | 24 | 0.0879798 | 1.58E-06 | 1185 | 89 | 13528 | 3.078481 | 5.71E-03 | 6.22E-05 | 2.90E-03 |
| 102 | GO:0009142~nucleoside triphosphate biosynthetic process | 26 | 0.0953114 | 1.63E-06 | 1185 | 102 | 13528 | 2.9099694 | 5.89E-03 | 6.36E-05 | 2.99E-03 |
| 103 | GO:0002541~activation of plasma proteins involved in acute inflammatory response | 16 | 0.0586532 | 1.79E-06 | 1185 | 43 | 13528 | 4.2478265 | 6.48E-03 | 6.91E-05 | 3.29E-03 |
| 104 | GO:0043254~regulation of protein complex assembly | 24 | 0.0879798 | 1.94E-06 | 1185 | 90 | 13528 | 3.0442757 | 7.04E-03 | 7.43E-05 | 3.58E-03 |
| 105 | GO:0016054~organic acid catabolic process | 27 | 0.0989772 | 2.54E-06 | 1185 | 111 | 13528 | 2.7768731 | 9.18E-03 | 9.61E-05 | 4.67E-03 |
| 106 | GO:0046395~carboxylic acid catabolic process | 27 | 0.0989772 | 2.54E-06 | 1185 | 111 | 13528 | 2.7768731 | 9.18E-03 | 9.61E-05 | 4.67E-03 |
| 107 | GO:0008064~regulation of actin polymerization or depolymerization | 19 | 0.0696506 | 2.75E-06 | 1185 | 61 | 13528 | 3.5558138 | 9.93E-03 | 1.03E-04 | 5.05E-03 |
| 108 | GO:0044270~nitrogen compound catabolic process | 20 | 0.0733165 | 2.83E-06 | 1185 | 67 | 13528 | 3.4077713 | 1.02E-02 | 1.05E-04 | 5.20E-03 |
| 109 | GO:0042743~hydrogen peroxide metabolic process | 12 | 0.0439899 | 3.10E-06 | 1185 | 25 | 13528 | 5.4796962 | 1.12E-02 | 1.14E-04 | 5.71E-03 |
| 110 | GO:0043086~negative regulation of catalytic activity | 49 | 0.1796254 | 3.27E-06 | 1185 | 277 | 13528 | 2.0194428 | 1.18E-02 | 1.19E-04 | 6.02E-03 |
| 111 | GO:0043623~cellular protein complex assembly | 34 | 0.124638 | 3.42E-06 | 1185 | 162 | 13528 | 2.3959577 | 1.23E-02 | 1.23E-04 | 6.29E-03 |
| 112 | GO:0016192~vesicle-mediated transport | 84 | 0.3079292 | 3.45E-06 | 1185 | 576 | 13528 | 1.6648383 | 1.24E-02 | 1.23E-04 | 6.34E-03 |
| 113 | GO:0032271~regulation of protein polymerization | 20 | 0.0733165 | 3.61E-06 | 1185 | 68 | 13528 | 3.357657 | 1.30E-02 | 1.27E-04 | 6.64E-03 |
| 114 | GO:0034614~cellular response to reactive oxygen species | 13 | 0.0476557 | 3.72E-06 | 1185 | 30 | 13528 | 4.946948 | 1.34E-02 | 1.30E-04 | 6.85E-03 |
| 115 | GO:0046496~nicotinamide nucleotide metabolic process | 15 | 0.0549874 | 3.78E-06 | 1185 | 40 | 13528 | 4.2810127 | 1.36E-02 | 1.31E-04 | 6.95E-03 |
| 116 | GO:0006769~nicotinamide metabolic process | 15 | 0.0549874 | 3.78E-06 | 1185 | 40 | 13528 | 4.2810127 | 1.36E-02 | 1.31E-04 | 6.95E-03 |
| 117 | GO:0031399~regulation of protein modification process | 51 | 0.186957 | 3.98E-06 | 1185 | 295 | 13528 | 1.9736194 | 1.43E-02 | 1.36E-04 | 7.31E-03 |
| 118 | GO:0044087~regulation of cellular component biogenesis | 31 | 0.1136405 | 4.35E-06 | 1185 | 142 | 13528 | 2.4922327 | 1.57E-02 | 1.48E-04 | 8.01E-03 |
| 119 | GO:0006941~striated muscle contraction | 16 | 0.0586532 | 4.70E-06 | 1185 | 46 | 13528 | 3.9707943 | 1.69E-02 | 1.58E-04 | 8.65E-03 |
| 120 | GO:0032270~positive regulation of cellular protein metabolic process | 43 | 0.1576304 | 4.82E-06 | 1185 | 233 | 13528 | 2.1068217 | 1.74E-02 | 1.61E-04 | 8.87E-03 |
| 121 | GO:0031401~positive regulation of protein modification process | 37 | 0.1356355 | 4.93E-06 | 1185 | 187 | 13528 | 2.2587874 | 1.77E-02 | 1.63E-04 | 9.06E-03 |
| 122 | GO:0051289~protein homotetramerization | 12 | 0.0439899 | 4.96E-06 | 1185 | 26 | 13528 | 5.2689387 | 1.78E-02 | 1.62E-04 | 9.11E-03 |
| 123 | GO:0050819~negative regulation of coagulation | 12 | 0.0439899 | 4.96E-06 | 1185 | 26 | 13528 | 5.2689387 | 1.78E-02 | 1.62E-04 | 9.11E-03 |
| 124 | GO:0009820~alkaloid metabolic process | 15 | 0.0549874 | 5.28E-06 | 1185 | 41 | 13528 | 4.1765977 | 1.90E-02 | 1.71E-04 | 9.72E-03 |
| 125 | GO:0050818~regulation of coagulation | 15 | 0.0549874 | 5.28E-06 | 1185 | 41 | 13528 | 4.1765977 | 1.90E-02 | 1.71E-04 | 9.72E-03 |
| 126 | GO:0007160~cell-matrix adhesion | 23 | 0.0843139 | 5.84E-06 | 1185 | 89 | 13528 | 2.950211 | 2.10E-02 | 1.87E-04 | 1.07E-02 |
| 127 | GO:0032956~regulation of actin cytoskeleton organization | 23 | 0.0843139 | 5.84E-06 | 1185 | 89 | 13528 | 2.950211 | 2.10E-02 | 1.87E-04 | 1.07E-02 |
| 128 | GO:0044093~positive regulation of molecular function | 84 | 0.3079292 | 6.76E-06 | 1185 | 586 | 13528 | 1.636428 | 2.42E-02 | 2.15E-04 | 1.24E-02 |
| 129 | GO:0044092~negative regulation of molecular function | 55 | 0.2016203 | 7.01E-06 | 1185 | 334 | 13528 | 1.8798858 | 2.51E-02 | 2.21E-04 | 1.29E-02 |
| 130 | GO:0019362~pyridine nucleotide metabolic process | 15 | 0.0549874 | 7.29E-06 | 1185 | 42 | 13528 | 4.0771549 | 2.61E-02 | 2.28E-04 | 1.34E-02 |
| 131 | GO:0007015~actin filament organization | 20 | 0.0733165 | 9.04E-06 | 1185 | 72 | 13528 | 3.1711205 | 3.23E-02 | 2.80E-04 | 1.66E-02 |
| 132 | GO:0034654~nucleobase, nucleoside, nucleotide and nucleic acid biosynthetic process | 37 | 0.1356355 | 1.03E-05 | 1185 | 193 | 13528 | 2.1885661 | 3.68E-02 | 3.18E-04 | 1.90E-02 |
| 133 | GO:0034404~nucleobase, nucleoside and nucleotide biosynthetic process | 37 | 0.1356355 | 1.03E-05 | 1185 | 193 | 13528 | 2.1885661 | 3.68E-02 | 3.18E-04 | 1.90E-02 |
| 134 | GO:0032970~regulation of actin filament-based process | 23 | 0.0843139 | 1.04E-05 | 1185 | 92 | 13528 | 2.8540084 | 3.70E-02 | 3.16E-04 | 1.91E-02 |
| 135 | GO:0009611~response to wounding | 77 | 0.2822684 | 1.05E-05 | 1185 | 530 | 13528 | 1.6585558 | 3.73E-02 | 3.17E-04 | 1.92E-02 |
| 136 | GO:0009165~nucleotide biosynthetic process | 36 | 0.1319696 | 1.12E-05 | 1185 | 186 | 13528 | 2.2095549 | 3.99E-02 | 3.36E-04 | 2.06E-02 |
| 137 | GO:0006749~glutathione metabolic process | 12 | 0.0439899 | 1.17E-05 | 1185 | 28 | 13528 | 4.8925859 | 4.15E-02 | 3.48E-04 | 2.15E-02 |
| 138 | GO:0006957~complement activation, alternative pathway | 9 | 0.0329924 | 1.24E-05 | 1185 | 15 | 13528 | 6.8496203 | 4.39E-02 | 3.65E-04 | 2.27E-02 |
| 139 | GO:0006511~ubiquitin-dependent protein catabolic process | 43 | 0.1576304 | 1.27E-05 | 1185 | 242 | 13528 | 2.0284688 | 4.52E-02 | 3.73E-04 | 2.34E-02 |
| 140 | GO:0051247~positive regulation of protein metabolic process | 43 | 0.1576304 | 1.41E-05 | 1185 | 243 | 13528 | 2.0201212 | 5.00E-02 | 4.10E-04 | 2.60E-02 |
| 141 | GO:0007599~hemostasis | 25 | 0.0916456 | 1.59E-05 | 1185 | 108 | 13528 | 2.6426004 | 5.61E-02 | 4.58E-04 | 2.92E-02 |
| 142 | GO:0006958~complement activation, classical pathway | 12 | 0.0439899 | 1.73E-05 | 1185 | 29 | 13528 | 4.723876 | 6.10E-02 | 4.96E-04 | 3.19E-02 |
| 143 | GO:0043085~positive regulation of catalytic activity | 75 | 0.2749368 | 1.81E-05 | 1185 | 520 | 13528 | 1.6465433 | 6.36E-02 | 5.13E-04 | 3.33E-02 |
| 144 | GO:0046907~intracellular transport | 90 | 0.3299241 | 1.87E-05 | 1185 | 657 | 13528 | 1.5638402 | 6.58E-02 | 5.27E-04 | 3.44E-02 |
| 145 | GO:0006739~NADP metabolic process | 9 | 0.0329924 | 2.28E-05 | 1185 | 16 | 13528 | 6.421519 | 7.95E-02 | 6.37E-04 | 4.20E-02 |
| 146 | GO:0006733~oxidoreduction coenzyme metabolic process | 16 | 0.0586532 | 2.49E-05 | 1185 | 52 | 13528 | 3.5126258 | 8.66E-02 | 6.91E-04 | 4.59E-02 |
| 147 | GO:0033043~regulation of organelle organization | 39 | 0.1429671 | 2.65E-05 | 1185 | 217 | 13528 | 2.0517296 | 9.16E-02 | 7.28E-04 | 4.87E-02 |
| 148 | GO:0006986~response to unfolded protein | 19 | 0.0696506 | 2.80E-05 | 1185 | 71 | 13528 | 3.0549949 | 9.67E-02 | 7.64E-04 | 5.15E-02 |
| 149 | GO:0030193~regulation of blood coagulation | 13 | 0.0476557 | 3.28E-05 | 1185 | 36 | 13528 | 4.1224566 | 1.12E-01 | 8.89E-04 | 6.04E-02 |
| 150 | GO:0002455~humoral immune response mediated by circulating immunoglobulin | 12 | 0.0439899 | 3.60E-05 | 1185 | 31 | 13528 | 4.4191098 | 1.23E-01 | 9.69E-04 | 6.62E-02 |
| 151 | GO:0019748~secondary metabolic process | 20 | 0.0733165 | 3.76E-05 | 1185 | 79 | 13528 | 2.8901351 | 1.28E-01 | 1.00E-03 | 6.91E-02 |
| 152 | GO:0042744~hydrogen peroxide catabolic process | 9 | 0.0329924 | 3.98E-05 | 1185 | 17 | 13528 | 6.0437826 | 1.35E-01 | 1.05E-03 | 7.32E-02 |
| 153 | GO:0030833~regulation of actin filament polymerization | 16 | 0.0586532 | 4.08E-05 | 1185 | 54 | 13528 | 3.3825285 | 1.38E-01 | 1.07E-03 | 7.49E-02 |

**Supplementary Table S2: (continued)**

| | | | | | | | | | | | |
|---|---|---|---|---|---|---|---|---|---|---|---|
| **154** | GO:0030100~regulation of endocytosis | 17 | 0.062319 | 4.93E-05 | 1185 | 61 | 13528 | 3.1815176 | 1.64E-01 | 1.29E-03 | 9.07E-02 |
| **155** | GO:0034599~cellular response to oxidative stress | 14 | 0.0513215 | 5.00E-05 | 1185 | 43 | 13528 | 3.7168482 | 1.66E-01 | 1.30E-03 | 9.19E-02 |
| **156** | GO:0007596~blood coagulation | 23 | 0.0843139 | 5.75E-05 | 1185 | 102 | 13528 | 2.5742037 | 1.88E-01 | 1.48E-03 | 1.06E-01 |
| **157** | GO:0050817~coagulation | 23 | 0.0843139 | 5.75E-05 | 1185 | 102 | 13528 | 2.5742037 | 1.88E-01 | 1.48E-03 | 1.06E-01 |
| **158** | GO:0043603~cellular amide metabolic process | 16 | 0.0586532 | 6.48E-05 | 1185 | 56 | 13528 | 3.2617239 | 2.10E-01 | 1.66E-03 | 1.19E-01 |
| **159** | GO:0070301~cellular response to hydrogen peroxide | 9 | 0.0329924 | 6.62E-05 | 1185 | 18 | 13528 | 5.7080169 | 2.14E-01 | 1.68E-03 | 1.22E-01 |
| **160** | GO:0060627~regulation of vesicle-mediated transport | 22 | 0.0806481 | 6.77E-05 | 1185 | 96 | 13528 | 2.6161744 | 2.18E-01 | 1.70E-03 | 1.24E-01 |
| **161** | GO:0043062~extracellular structure organization | 31 | 0.1136405 | 7.12E-05 | 1185 | 163 | 13528 | 2.1711475 | 2.28E-01 | 1.78E-03 | 1.31E-01 |
| **162** | GO:0010941~regulation of cell death | 104 | 0.3812456 | 7.51E-05 | 1185 | 815 | 13528 | 1.45677 | 2.39E-01 | 1.86E-03 | 1.38E-01 |
| **163** | GO:0030195~negative regulation of blood coagulation | 10 | 0.0366582 | 7.71E-05 | 1185 | 23 | 13528 | 4.9634929 | 2.44E-01 | 1.90E-03 | 1.42E-01 |
| **164** | GO:0051262~protein tetramerization | 13 | 0.0476557 | 8.01E-05 | 1185 | 39 | 13528 | 3.8053446 | 2.52E-01 | 1.96E-03 | 1.47E-01 |
| **165** | GO:0034655~nucleobase, nucleoside, nucleotide and nucleic acid catabolic process | 16 | 0.0586532 | 1.00E-04 | 1185 | 58 | 13528 | 3.1492507 | 3.05E-01 | 2.44E-03 | 1.85E-01 |
| **166** | GO:0034656~nucleobase, nucleoside and nucleotide catabolic process | 16 | 0.0586532 | 1.00E-04 | 1185 | 58 | 13528 | 3.1492507 | 3.05E-01 | 2.44E-03 | 1.85E-01 |
| **167** | GO:0015985~energy coupled proton transport, down electrochemical gradient | 13 | 0.0476557 | 1.05E-04 | 1185 | 40 | 13528 | 3.710211 | 3.18E-01 | 2.55E-03 | 1.94E-01 |
| **168** | GO:0015986~ATP synthesis coupled proton transport | 13 | 0.0476557 | 1.05E-04 | 1185 | 40 | 13528 | 3.710211 | 3.18E-01 | 2.55E-03 | 1.94E-01 |
| **169** | GO:0060548~negative regulation of cell death | 54 | 0.1979545 | 1.16E-04 | 1185 | 360 | 13528 | 1.7124051 | 3.44E-01 | 2.79E-03 | 2.13E-01 |
| **170** | GO:0051789~response to protein stimulus | 23 | 0.0843139 | 1.22E-04 | 1185 | 107 | 13528 | 2.4539138 | 3.58E-01 | 2.91E-03 | 2.24E-01 |
| **171** | GO:0006959~humoral immune response | 19 | 0.0696506 | 1.27E-04 | 1185 | 79 | 13528 | 2.7456284 | 3.69E-01 | 3.00E-03 | 2.33E-01 |
| **172** | GO:0042274~ribosomal small subunit biogenesis | 7 | 0.0256608 | 1.39E-04 | 1185 | 11 | 13528 | 7.2647488 | 3.97E-01 | 3.28E-03 | 2.56E-01 |
| **173** | GO:0043066~negative regulation of apoptosis | 53 | 0.1942886 | 1.40E-04 | 1185 | 354 | 13528 | 1.7091802 | 3.99E-01 | 3.28E-03 | 2.58E-01 |
| **174** | GO:0009261~ribonucleotide catabolic process | 10 | 0.0366582 | 1.64E-04 | 1185 | 25 | 13528 | 4.5664135 | 4.49E-01 | 3.81E-03 | 3.01E-01 |
| **175** | GO:0043067~regulation of programmed cell death | 102 | 0.373914 | 1.64E-04 | 1185 | 812 | 13528 | 1.4340338 | 4.49E-01 | 3.79E-03 | 3.01E-01 |
| **176** | GO:0006800~oxygen and reactive oxygen species metabolic process | 17 | 0.062319 | 1.66E-04 | 1185 | 67 | 13528 | 2.8966056 | 4.52E-01 | 3.80E-03 | 3.04E-01 |
| **177** | GO:0042981~regulation of apoptosis | 101 | 0.3702482 | 1.76E-04 | 1185 | 804 | 13528 | 1.4341037 | 4.71E-01 | 4.00E-03 | 3.22E-01 |
| **178** | GO:0032535~regulation of cellular component size | 43 | 0.1576304 | 1.87E-04 | 1185 | 271 | 13528 | 1.8114002 | 4.92E-01 | 4.23E-03 | 3.43E-01 |
| **179** | GO:0043069~negative regulation of programmed cell death | 53 | 0.1942886 | 2.02E-04 | 1185 | 359 | 13528 | 1.6853755 | 5.19E-01 | 4.54E-03 | 3.70E-01 |
| **180** | GO:0050878~regulation of body fluid levels | 27 | 0.0989772 | 2.04E-04 | 1185 | 141 | 13528 | 2.186049 | 5.24E-01 | 4.57E-03 | 3.75E-01 |
| **181** | GO:0034220~ion transmembrane transport | 14 | 0.0513215 | 2.19E-04 | 1185 | 49 | 13528 | 3.2617239 | 5.48E-01 | 4.86E-03 | 4.01E-01 |
| **182** | GO:0046364~monosaccharide biosynthetic process | 12 | 0.0439899 | 2.23E-04 | 1185 | 37 | 13528 | 3.7024974 | 5.55E-01 | 4.92E-03 | 4.09E-01 |
| **183** | GO:0042592~homeostatic process | 95 | 0.3482532 | 2.25E-04 | 1185 | 751 | 13528 | 1.4441055 | 5.58E-01 | 4.94E-03 | 4.13E-01 |
| **184** | GO:0007265~Ras protein signal transduction | 22 | 0.0806481 | 2.58E-04 | 1185 | 105 | 13528 | 2.3919309 | 6.09E-01 | 5.63E-03 | 4.74E-01 |
| **185** | GO:0006014~D-ribose metabolic process | 5 | 0.0183291 | 2.72E-04 | 1185 | 5 | 13528 | 11.416034 | 6.27E-01 | 5.89E-03 | 4.98E-01 |
| **186** | GO:0006818~hydrogen transport | 16 | 0.0586532 | 2.72E-04 | 1185 | 63 | 13528 | 2.8993102 | 6.28E-01 | 5.86E-03 | 4.99E-01 |
| **187** | GO:0002449~lymphocyte mediated immunity | 17 | 0.062319 | 2.85E-04 | 1185 | 70 | 13528 | 2.7724653 | 6.44E-01 | 6.09E-03 | 5.22E-01 |
| **188** | GO:0046165~alcohol biosynthetic process | 13 | 0.0476557 | 2.87E-04 | 1185 | 44 | 13528 | 3.3729191 | 6.47E-01 | 6.11E-03 | 5.26E-01 |
| **189** | GO:0046700~heterocycle catabolic process | 18 | 0.0659848 | 2.89E-04 | 1185 | 77 | 13528 | 2.6686832 | 6.50E-01 | 6.12E-03 | 5.31E-01 |
| **190** | GO:0010038~response to metal ion | 25 | 0.0916456 | 3.08E-04 | 1185 | 129 | 13528 | 2.2124096 | 6.73E-01 | 6.47E-03 | 5.64E-01 |
| **191** | GO:0030155~regulation of cell adhesion | 26 | 0.0953114 | 3.17E-04 | 1185 | 137 | 13528 | 2.1665466 | 6.84E-01 | 6.63E-03 | 5.81E-01 |
| **192** | GO:0030834~regulation of actin filament depolymerization | 10 | 0.0366582 | 3.21E-04 | 1185 | 27 | 13528 | 4.2281607 | 6.88E-01 | 6.67E-03 | 5.88E-01 |
| **193** | GO:0051235~maintenance of location | 16 | 0.0586532 | 3.27E-04 | 1185 | 64 | 13528 | 2.8540084 | 6.95E-01 | 6.76E-03 | 6.00E-01 |
| **194** | GO:0000398~nuclear mRNA splicing, via spliceosome | 28 | 0.1026431 | 3.27E-04 | 1185 | 153 | 13528 | 2.0892088 | 6.95E-01 | 6.72E-03 | 6.00E-01 |
| **195** | GO:0000375~RNA splicing, via transesterification reactions | 28 | 0.1026431 | 3.27E-04 | 1185 | 153 | 13528 | 2.0892088 | 6.95E-01 | 6.72E-03 | 6.00E-01 |
| **196** | GO:0000377~RNA splicing, via transesterification reactions with bulged adenosine as nucleophile | 28 | 0.1026431 | 3.27E-04 | 1185 | 153 | 13528 | 2.0892088 | 6.95E-01 | 6.72E-03 | 6.00E-01 |
| **197** | GO:0051604~protein maturation | 24 | 0.0879798 | 3.34E-04 | 1185 | 122 | 13528 | 2.2457771 | 7.02E-01 | 6.82E-03 | 6.12E-01 |
| **198** | GO:0043244~regulation of protein complex disassembly | 14 | 0.0513215 | 3.36E-04 | 1185 | 51 | 13528 | 3.1338132 | 7.05E-01 | 6.84E-03 | 6.17E-01 |
| **199** | GO:0009310~amine catabolic process | 18 | 0.0659848 | 3.40E-04 | 1185 | 78 | 13528 | 2.6344693 | 7.09E-01 | 6.88E-03 | 6.24E-01 |
| **200** | GO:0007517~muscle organ development | 35 | 0.1283038 | 3.58E-04 | 1185 | 211 | 13528 | 1.8936549 | 7.27E-01 | 7.19E-03 | 6.56E-01 |
| **201** | GO:0002443~leukocyte mediated immunity | 19 | 0.0696506 | 3.90E-04 | 1185 | 86 | 13528 | 2.522147 | 7.58E-01 | 7.80E-03 | 7.15E-01 |
| **202** | GO:0051605~protein maturation by peptide bond cleavage | 19 | 0.0696506 | 3.90E-04 | 1185 | 86 | 13528 | 2.522147 | 7.58E-01 | 7.80E-03 | 7.15E-01 |
| **203** | GO:0006937~regulation of muscle contraction | 17 | 0.062319 | 4.00E-04 | 1185 | 72 | 13528 | 2.6954524 | 7.66E-01 | 7.94E-03 | 7.33E-01 |
| **204** | GO:0008104~protein localization | 107 | 0.3922431 | 4.15E-04 | 1185 | 882 | 13528 | 1.3849383 | 7.78E-01 | 8.20E-03 | 7.60E-01 |
| **205** | GO:0002253~activation of immune response | 20 | 0.0733165 | 4.34E-04 | 1185 | 94 | 13528 | 2.4289434 | 7.93E-01 | 8.53E-03 | 7.95E-01 |
| **206** | GO:0031032~actomyosin structure organization | 10 | 0.0366582 | 4.36E-04 | 1185 | 28 | 13528 | 4.0771549 | 7.95E-01 | 8.52E-03 | 7.99E-01 |
| **207** | GO:0051241~negative regulation of multicellular organismal process | 29 | 0.1063089 | 4.48E-04 | 1185 | 164 | 13528 | 2.0186889 | 8.03E-01 | 8.70E-03 | 8.20E-01 |
| **208** | GO:0031333~negative regulation of protein complex assembly | 11 | 0.0403241 | 4.72E-04 | 1185 | 34 | 13528 | 3.6934227 | 8.20E-01 | 9.12E-03 | 8.64E-01 |
| **209** | GO:0006098~pentose-phosphate shunt | 6 | 0.0219949 | 4.75E-04 | 1185 | 9 | 13528 | 7.6106892 | 8.22E-01 | 9.14E-03 | 8.71E-01 |
| **210** | GO:0009154~purine ribonucleotide catabolic process | 9 | 0.0329924 | 4.99E-04 | 1185 | 23 | 13528 | 4.4671436 | 8.36E-01 | 9.53E-03 | 9.13E-01 |
| **211** | GO:0007044~cell-substrate junction assembly | 9 | 0.0329924 | 4.99E-04 | 1185 | 23 | 13528 | 4.4671436 | 8.36E-01 | 9.53E-03 | 9.13E-01 |
| **212** | GO:0030199~collagen fibril organization | 10 | 0.0366582 | 5.84E-04 | 1185 | 29 | 13528 | 3.9365634 | 8.80E-01 | 1.11E-02 | 1.07E+00 |
| **213** | GO:0015992~proton transport | 15 | 0.0549874 | 6.42E-04 | 1185 | 61 | 13528 | 2.8072214 | 9.03E-01 | 1.21E-02 | 1.17E+00 |
| **214** | GO:0009063~cellular amino acid catabolic process | 16 | 0.0586532 | 6.53E-04 | 1185 | 68 | 13528 | 2.6861256 | 9.07E-01 | 1.23E-02 | 1.19E+00 |
| **215** | GO:0009719~response to endogenous stimulus | 56 | 0.2052861 | 6.68E-04 | 1185 | 405 | 13528 | 1.5785133 | 9.12E-01 | 1.25E-02 | 1.22E+00 |
| **216** | GO:0051651~maintenance of location in cell | 13 | 0.0476557 | 6.83E-04 | 1185 | 48 | 13528 | 3.0918425 | 9.16E-01 | 1.27E-02 | 1.25E+00 |
| **217** | GO:0009143~nucleoside triphosphate catabolic process | 9 | 0.0329924 | 6.91E-04 | 1185 | 24 | 13528 | 4.2810127 | 9.19E-01 | 1.28E-02 | 1.26E+00 |
| **218** | GO:0010608~posttranscriptional regulation of gene expression | 34 | 0.124638 | 7.45E-04 | 1185 | 211 | 13528 | 1.8395505 | 9.33E-01 | 1.37E-02 | 1.36E+00 |
| **219** | GO:0034637~cellular carbohydrate biosynthetic process | 16 | 0.0586532 | 7.68E-04 | 1185 | 69 | 13528 | 2.6471962 | 9.38E-01 | 1.41E-02 | 1.40E+00 |
| **220** | GO:0010927~cellular component assembly involved in morphogenesis | 11 | 0.0403241 | 7.79E-04 | 1185 | 36 | 13528 | 3.4882325 | 9.41E-01 | 1.42E-02 | 1.42E+00 |
| **221** | GO:0055072~iron ion homeostasis | 11 | 0.0403241 | 7.79E-04 | 1185 | 36 | 13528 | 3.4882325 | 9.41E-01 | 1.42E-02 | 1.42E+00 |
| **222** | GO:0045184~establishment of protein localization | 94 | 0.3445874 | 7.92E-04 | 1185 | 769 | 13528 | 1.395458 | 9.44E-01 | 1.43E-02 | 1.45E+00 |
| **223** | GO:0051258~protein polymerization | 13 | 0.0476557 | 8.33E-04 | 1185 | 49 | 13528 | 3.0287436 | 9.52E-01 | 1.50E-02 | 1.52E+00 |
| **224** | GO:0006107~oxaloacetate metabolic process | 6 | 0.0219949 | 8.82E-04 | 1185 | 10 | 13528 | 6.8496203 | 9.59E-01 | 1.58E-02 | 1.61E+00 |
| **225** | GO:0009185~ribonucleoside diphosphate metabolic process | 6 | 0.0219949 | 8.82E-04 | 1185 | 10 | 13528 | 6.8496203 | 9.59E-01 | 1.58E-02 | 1.61E+00 |
| **226** | GO:0030194~positive regulation of blood coagulation | 6 | 0.0219949 | 8.82E-04 | 1185 | 10 | 13528 | 6.8496203 | 9.59E-01 | 1.58E-02 | 1.61E+00 |
| **227** | GO:0006916~anti-apoptosis | 33 | 0.1209722 | 9.99E-04 | 1185 | 206 | 13528 | 1.8287821 | 9.73E-01 | 1.78E-02 | 1.82E+00 |
| **228** | GO:0006879~cellular iron ion homeostasis | 10 | 0.0366582 | 1.00E-03 | 1185 | 31 | 13528 | 3.6825915 | 9.74E-01 | 1.78E-02 | 1.83E+00 |
| **229** | GO:0009065~glutamine family amino acid catabolic process | 8 | 0.0293266 | 1.08E-03 | 1185 | 20 | 13528 | 4.5664135 | 9.80E-01 | 1.91E-02 | 1.97E+00 |
| **230** | GO:0009725~response to hormone stimulus | 51 | 0.186957 | 1.10E-03 | 1185 | 367 | 13528 | 1.5864243 | 9.81E-01 | 1.93E-02 | 2.00E+00 |
| **231** | GO:0042060~wound healing | 31 | 0.1136405 | 1.18E-03 | 1185 | 191 | 13528 | 1.8528641 | 9.86E-01 | 2.06E-02 | 2.15E+00 |
| **232** | GO:0009166~nucleotide catabolic process | 13 | 0.0476557 | 1.22E-03 | 1185 | 51 | 13528 | 2.9099694 | 9.88E-01 | 2.11E-02 | 2.22E+00 |
| **233** | GO:0007266~Rho protein signal transduction | 11 | 0.0403241 | 1.23E-03 | 1185 | 38 | 13528 | 3.3046414 | 9.89E-01 | 2.13E-02 | 2.24E+00 |
| **234** | GO:0007568~aging | 21 | 0.0769823 | 1.27E-03 | 1185 | 110 | 13528 | 2.1794246 | 9.90E-01 | 2.18E-02 | 2.31E+00 |
| **235** | GO:0015031~protein transport | 92 | 0.3372558 | 1.29E-03 | 1185 | 762 | 13528 | 1.3783138 | 9.91E-01 | 2.21E-02 | 2.35E+00 |
| **236** | GO:0002252~immune effector process | 24 | 0.0879798 | 1.29E-03 | 1185 | 134 | 13528 | 2.0446628 | 9.91E-01 | 2.20E-02 | 2.35E+00 |
| **237** | GO:0032101~regulation of response to external stimulus | 27 | 0.0989772 | 1.35E-03 | 1185 | 159 | 13528 | 1.9385718 | 9.93E-01 | 2.29E-02 | 2.46E+00 |
| **238** | GO:0042440~pigment metabolic process | 12 | 0.0439899 | 1.38E-03 | 1185 | 45 | 13528 | 3.0442757 | 9.93E-01 | 2.33E-02 | 2.51E+00 |
| **239** | GO:0051051~negative regulation of transport | 24 | 0.0879798 | 1.43E-03 | 1185 | 135 | 13528 | 2.0295171 | 9.95E-01 | 2.41E-02 | 2.61E+00 |
| **240** | GO:0009146~purine nucleoside triphosphate catabolic process | 8 | 0.0293266 | 1.50E-03 | 1185 | 21 | 13528 | 4.3489652 | 9.96E-01 | 2.50E-02 | 2.72E+00 |
| **241** | GO:0030239~myofibril assembly | 8 | 0.0293266 | 1.50E-03 | 1185 | 21 | 13528 | 4.3489652 | 9.96E-01 | 2.50E-02 | 2.72E+00 |
| **242** | GO:0006195~purine nucleotide catabolic process | 10 | 0.0366582 | 1.63E-03 | 1185 | 33 | 13528 | 3.4594042 | 9.97E-01 | 2.71E-02 | 2.96E+00 |
| **243** | GO:0006002~fructose 6-phosphate metabolic process | 5 | 0.0183291 | 1.65E-03 | 1185 | 7 | 13528 | 8.1543098 | 9.97E-01 | 2.72E-02 | 2.98E+00 |
| **244** | GO:0010873~positive regulation of cholesterol esterification | 5 | 0.0183291 | 1.65E-03 | 1185 | 7 | 13528 | 8.1543098 | 9.97E-01 | 2.72E-02 | 2.98E+00 |
| **245** | GO:0006200~ATP catabolic process | 7 | 0.0256608 | 1.65E-03 | 1185 | 16 | 13528 | 4.9945148 | 9.97E-01 | 2.71E-02 | 2.99E+00 |
| **246** | GO:0034381~lipoprotein particle clearance | 7 | 0.0256608 | 1.65E-03 | 1185 | 16 | 13528 | 4.9945148 | 9.97E-01 | 2.71E-02 | 2.99E+00 |
| **247** | GO:0008380~RNA splicing | 41 | 0.1502988 | 1.78E-03 | 1185 | 284 | 13528 | 1.6480894 | 9.98E-01 | 2.91E-02 | 3.22E+00 |
| **248** | GO:0006508~proteolysis | 120 | 0.4398988 | 1.99E-03 | 1185 | 1054 | 13528 | 1.2997382 | 9.99E-01 | 3.23E-02 | 3.59E+00 |
| **249** | GO:0016053~organic acid biosynthetic process | 26 | 0.0953114 | 2.02E-03 | 1185 | 155 | 13528 | 1.9149476 | 9.99E-01 | 3.26E-02 | 3.64E+00 |
| **250** | GO:0046394~carboxylic acid biosynthetic process | 26 | 0.0953114 | 2.02E-03 | 1185 | 155 | 13528 | 1.9149476 | 9.99E-01 | 3.26E-02 | 3.64E+00 |
| **251** | GO:0051693~actin filament capping | 8 | 0.0293266 | 2.03E-03 | 1185 | 22 | 13528 | 4.151285 | 9.99E-01 | 3.28E-02 | 3.68E+00 |
| **252** | GO:0016064~immunoglobulin mediated immune response | 13 | 0.0476557 | 2.06E-03 | 1185 | 54 | 13528 | 2.7483044 | 9.99E-01 | 3.30E-02 | 3.72E+00 |
| **253** | GO:0065004~protein-DNA complex assembly | 18 | 0.0659848 | 2.11E-03 | 1185 | 91 | 13528 | 2.2581166 | 1.00E+00 | 3.37E-02 | 3.81E+00 |
| **254** | GO:0006635~fatty acid beta-oxidation | 9 | 0.0329924 | 2.13E-03 | 1185 | 28 | 13528 | 3.6694394 | 1.00E+00 | 3.38E-02 | 3.85E+00 |
| **255** | GO:0051130~positive regulation of cellular component organization | 29 | 0.1063089 | 2.13E-03 | 1185 | 181 | 13528 | 1.8290883 | 1.00E+00 | 3.37E-02 | 3.85E+00 |
| **256** | GO:0048193~Golgi vesicle transport | 23 | 0.0843139 | 2.17E-03 | 1185 | 131 | 13528 | 2.0043418 | 1.00E+00 | 3.42E-02 | 3.92E+00 |
| **257** | GO:0000278~mitotic cell cycle | 50 | 0.1832912 | 2.23E-03 | 1185 | 370 | 13528 | 1.5427073 | 1.00E+00 | 3.50E-02 | 4.03E+00 |
| **258** | GO:0006334~nucleosome assembly | 17 | 0.062319 | 2.29E-03 | 1185 | 84 | 13528 | 2.3103878 | 1.00E+00 | 3.57E-02 | 4.13E+00 |
| **259** | GO:0034329~cell junction assembly | 11 | 0.0403241 | 2.30E-03 | 1185 | 41 | 13528 | 3.0628383 | 1.00E+00 | 3.57E-02 | 4.14E+00 |
| **260** | GO:0009132~nucleoside diphosphate metabolic process | 7 | 0.0256608 | 2.36E-03 | 1185 | 17 | 13528 | 4.7007198 | 1.00E+00 | 3.64E-02 | 4.25E+00 |
| **261** | GO:0006000~fructose metabolic process | 7 | 0.0256608 | 2.36E-03 | 1185 | 17 | 13528 | 4.7007198 | 1.00E+00 | 3.64E-02 | 4.25E+00 |
| **262** | GO:0065005~protein-lipid complex assembly | 6 | 0.0219949 | 2.39E-03 | 1185 | 12 | 13528 | 5.7080169 | 1.00E+00 | 3.67E-02 | 4.30E+00 |
| **263** | GO:0034377~plasma lipoprotein particle assembly | 6 | 0.0219949 | 2.39E-03 | 1185 | 12 | 13528 | 5.7080169 | 1.00E+00 | 3.67E-02 | 4.30E+00 |
| **264** | GO:0042730~fibrinolysis | 6 | 0.0219949 | 2.39E-03 | 1185 | 12 | 13528 | 5.7080169 | 1.00E+00 | 3.67E-02 | 4.30E+00 |
| **265** | GO:0051592~response to calcium ion | 13 | 0.0476557 | 2.43E-03 | 1185 | 55 | 13528 | 2.6983353 | 1.00E+00 | 3.72E-02 | 4.38E+00 |
| **266** | GO:0070482~response to oxygen levels | 24 | 0.0879798 | 2.57E-03 | 1185 | 141 | 13528 | 1.9431547 | 1.00E+00 | 3.92E-02 | 4.63E+00 |
| **267** | GO:0032272~negative regulation of protein polymerization | 9 | 0.0329924 | 2.72E-03 | 1185 | 29 | 13528 | 3.542907 | 1.00E+00 | 4.12E-02 | 4.89E+00 |
| **268** | GO:0051271~negative regulation of cell motion | 14 | 0.0513215 | 2.76E-03 | 1185 | 63 | 13528 | 2.5368964 | 1.00E+00 | 4.17E-02 | 4.96E+00 |
| **269** | GO:0019724~B cell mediated immunity | 13 | 0.0476557 | 2.85E-03 | 1185 | 56 | 13528 | 2.6501507 | 1.00E+00 | 4.28E-02 | 5.12E+00 |
| **270** | GO:0010872~regulation of cholesterol esterification | 5 | 0.0183291 | 3.07E-03 | 1185 | 8 | 13528 | 7.1350211 | 1.00E+00 | 4.57E-02 | 5.49E+00 |
| **271** | GO:0043094~cellular metabolic compound salvage | 5 | 0.0183291 | 3.07E-03 | 1185 | 8 | 13528 | 7.1350211 | 1.00E+00 | 4.57E-02 | 5.49E+00 |
| **272** | GO:0009179~purine ribonucleoside diphosphate metabolic process | 5 | 0.0183291 | 3.07E-03 | 1185 | 8 | 13528 | 7.1350211 | 1.00E+00 | 4.57E-02 | 5.49E+00 |
| **273** | GO:0006108~malate metabolic process | 5 | 0.0183291 | 3.07E-03 | 1185 | 8 | 13528 | 7.1350211 | 1.00E+00 | 4.57E-02 | 5.49E+00 |
| **274** | GO:0009135~purine nucleoside diphosphate metabolic process | 5 | 0.0183291 | 3.07E-03 | 1185 | 8 | 13528 | 7.1350211 | 1.00E+00 | 4.57E-02 | 5.49E+00 |
| **275** | GO:0001937~negative regulation of endothelial cell proliferation | 7 | 0.0256608 | 3.28E-03 | 1185 | 18 | 13528 | 4.4395687 | 1.00E+00 | 4.87E-02 | 5.86E+00 |
| **276** | GO:0031497~chromatin assembly | 17 | 0.062319 | 3.32E-03 | 1185 | 87 | 13528 | 2.2307192 | 1.00E+00 | 4.90E-02 | 5.93E+00 |
| **277** | GO:0034330~cell junction organization | 13 | 0.0476557 | 3.33E-03 | 1185 | 57 | 13528 | 2.6036568 | 1.00E+00 | 4.90E-02 | 5.95E+00 |
| **278** | GO:0032368~regulation of lipid transport | 9 | 0.0329924 | 3.43E-03 | 1185 | 30 | 13528 | 3.4248101 | 1.00E+00 | 5.02E-02 | 6.12E+00 |
| **279** | GO:0009066~aspartate family amino acid metabolic process | 8 | 0.0293266 | 3.54E-03 | 1185 | 24 | 13528 | 3.8053446 | 1.00E+00 | 5.15E-02 | 6.31E+00 |
| **280** | GO:0030835~negative regulation of actin filament depolymerization | 8 | 0.0293266 | 3.54E-03 | 1185 | 24 | 13528 | 3.8053446 | 1.00E+00 | 5.15E-02 | 6.31E+00 |
| **281** | GO:0050820~positive regulation of coagulation | 6 | 0.0219949 | 3.60E-03 | 1185 | 13 | 13528 | 5.2689387 | 1.00E+00 | 5.23E-02 | 6.43E+00 |
| **282** | GO:0050778~positive regulation of immune response | 24 | 0.0879798 | 3.70E-03 | 1185 | 145 | 13528 | 1.8895504 | 1.00E+00 | 5.34E-02 | 6.59E+00 |
| **283** | GO:0016485~protein processing | 20 | 0.0733165 | 3.75E-03 | 1185 | 112 | 13528 | 2.0385775 | 1.00E+00 | 5.40E-02 | 6.68E+00 |
| **284** | GO:0008652~cellular amino acid biosynthetic process | 12 | 0.0439899 | 3.99E-03 | 1185 | 51 | 13528 | 2.6861256 | 1.00E+00 | 5.71E-02 | 7.09E+00 |
| **285** | GO:0042692~muscle cell differentiation | 21 | 0.0769823 | 4.07E-03 | 1185 | 121 | 13528 | 1.9812951 | 1.00E+00 | 5.79E-02 | 7.22E+00 |
| **286** | GO:0006954~inflammatory response | 44 | 0.1612962 | 4.09E-03 | 1185 | 325 | 13528 | 1.5455553 | 1.00E+00 | 5.80E-02 | 7.26E+00 |
| **287** | GO:0045087~innate immune response | 23 | 0.0843139 | 4.20E-03 | 1185 | 138 | 13528 | 1.9026723 | 1.00E+00 | 5.93E-02 | 7.44E+00 |
| **288** | GO:0009203~ribonucleoside triphosphate catabolic process | 7 | 0.0256608 | 4.44E-03 | 1185 | 19 | 13528 | 4.2059072 | 1.00E+00 | 6.24E-02 | 7.86E+00 |
| **289** | GO:0009207~purine ribonucleoside triphosphate catabolic process | 7 | 0.0256608 | 4.44E-03 | 1185 | 19 | 13528 | 4.2059072 | 1.00E+00 | 6.24E-02 | 7.86E+00 |
| **290** | GO:0007017~microtubule-based process | 36 | 0.1319696 | 4.45E-03 | 1185 | 253 | 13528 | 1.6244159 | 1.00E+00 | 6.23E-02 | 7.88E+00 |
| **291** | GO:0006094~gluconeogenesis | 8 | 0.0293266 | 4.55E-03 | 1185 | 25 | 13528 | 3.6531308 | 1.00E+00 | 6.33E-02 | 8.04E+00 |
| **292** | GO:0006873~cellular ion homeostasis | 49 | 0.1796254 | 4.57E-03 | 1185 | 374 | 13528 | 1.4956836 | 1.00E+00 | 6.33E-02 | 8.07E+00 |
| **293** | GO:0009755~hormone-mediated signaling | 12 | 0.0439899 | 4.67E-03 | 1185 | 52 | 13528 | 2.6344693 | 1.00E+00 | 6.45E-02 | 8.25E+00 |
| **294** | GO:0051495~positive regulation of cytoskeleton organization | 11 | 0.0403241 | 4.75E-03 | 1185 | 45 | 13528 | 2.790586 | 1.00E+00 | 6.53E-02 | 8.38E+00 |
| **295** | GO:0006413~translational initiation | 11 | 0.0403241 | 4.75E-03 | 1185 | 45 | 13528 | 2.790586 | 1.00E+00 | 6.53E-02 | 8.38E+00 |
| **296** | GO:0006790~sulfur metabolic process | 20 | 0.0733165 | 5.05E-03 | 1185 | 115 | 13528 | 1.9853972 | 1.00E+00 | 6.91E-02 | 8.90E+00 |
| **297** | GO:0009113~purine base biosynthetic process | 5 | 0.0183291 | 5.14E-03 | 1185 | 9 | 13528 | 6.342241 | 1.00E+00 | 6.99E-02 | 9.04E+00 |
| **298** | GO:0051917~regulation of fibrinolysis | 5 | 0.0183291 | 5.14E-03 | 1185 | 9 | 13528 | 6.342241 | 1.00E+00 | 6.99E-02 | 9.04E+00 |
| **299** | GO:0045785~positive regulation of cell adhesion | 13 | 0.0476557 | 5.17E-03 | 1185 | 60 | 13528 | 2.473474 | 1.00E+00 | 7.01E-02 | 9.09E+00 |
| **300** | GO:0007155~cell adhesion | 82 | 0.3005975 | 5.41E-03 | 1185 | 700 | 13528 | 1.3373068 | 1.00E+00 | 7.30E-02 | 9.50E+00 |
| **301** | GO:0050880~regulation of blood vessel size | 12 | 0.0439899 | 5.44E-03 | 1185 | 53 | 13528 | 2.5847624 | 1.00E+00 | 7.31E-02 | 9.55E+00 |
| **302** | GO:0035150~regulation of tube size | 12 | 0.0439899 | 5.44E-03 | 1185 | 53 | 13528 | 2.5847624 | 1.00E+00 | 7.31E-02 | 9.55E+00 |
| **303** | GO:0022610~biological adhesion | 82 | 0.3005975 | 5.68E-03 | 1185 | 701 | 13528 | 1.3353991 | 1.00E+00 | 7.59E-02 | 9.94E+00 |
| **304** | GO:0044242~cellular lipid catabolic process | 15 | 0.0549874 | 5.72E-03 | 1185 | 76 | 13528 | 2.2531646 | 1.00E+00 | 7.62E-02 | 1.00E+01 |
| **305** | GO:0030388~fructose 1,6-bisphosphate metabolic process | 4 | 0.0146633 | 5.84E-03 | 1185 | 5 | 13528 | 9.132827 | 1.00E+00 | 7.74E-02 | 1.02E+01 |
| **306** | GO:0042026~protein refolding | 4 | 0.0146633 | 5.84E-03 | 1185 | 5 | 13528 | 9.132827 | 1.00E+00 | 7.74E-02 | 1.02E+01 |
| **307** | GO:0009167~purine ribonucleoside monophosphate metabolic process | 7 | 0.0256608 | 5.88E-03 | 1185 | 20 | 13528 | 3.9956118 | 1.00E+00 | 7.77E-02 | 1.03E+01 |

**Supplementary Table S2: (continued)**

| | | | | | | | | | | | |
|---|---|---|---|---|---|---|---|---|---|---|---|
| **308** | GO:0009126~purine nucleoside monophosphate metabolic process | 7 | 0.0256608 | 5.88E-03 | 1185 | 20 | 13528 | 3.9956118 | 1.00E+00 | 7.77E-02 | 1.03E+01 |
| **309** | GO:0016071~mRNA metabolic process | 48 | 0.1759595 | 6.05E-03 | 1185 | 370 | 13528 | 1.480999 | 1.00E+00 | 7.94E-02 | 1.06E+01 |
| **310** | GO:0055082~cellular chemical homeostasis | 49 | 0.1796254 | 6.15E-03 | 1185 | 380 | 13528 | 1.4720675 | 1.00E+00 | 8.04E-02 | 1.07E+01 |
| **311** | GO:0008015~blood circulation | 28 | 0.1026431 | 6.15E-03 | 1185 | 186 | 13528 | 1.7185427 | 1.00E+00 | 8.02E-02 | 1.07E+01 |
| **312** | GO:0003013~circulatory system process | 28 | 0.1026431 | 6.15E-03 | 1185 | 186 | 13528 | 1.7185427 | 1.00E+00 | 8.02E-02 | 1.07E+01 |
| **313** | GO:0001666~response to hypoxia | 22 | 0.0806481 | 6.21E-03 | 1185 | 134 | 13528 | 1.8742742 | 1.00E+00 | 8.06E-02 | 1.08E+01 |
| **314** | GO:0034728~nucleosome organization | 17 | 0.062319 | 6.50E-03 | 1185 | 93 | 13528 | 2.0868019 | 1.00E+00 | 8.40E-02 | 1.13E+01 |
| **315** | GO:0006953~acute-phase response | 10 | 0.0366582 | 6.65E-03 | 1185 | 40 | 13528 | 2.8540084 | 1.00E+00 | 8.55E-02 | 1.15E+01 |
| **316** | GO:0009067~aspartate family amino acid biosynthetic process | 6 | 0.0219949 | 7.26E-03 | 1185 | 15 | 13528 | 4.5664135 | 1.00E+00 | 9.26E-02 | 1.25E+01 |
| **317** | GO:0051017~actin filament bundle formation | 7 | 0.0256608 | 7.64E-03 | 1185 | 21 | 13528 | 3.8053446 | 1.00E+00 | 9.69E-02 | 1.32E+01 |
| **318** | GO:0043242~negative regulation of protein complex disassembly | 10 | 0.0366582 | 7.87E-03 | 1185 | 41 | 13528 | 2.7843985 | 1.00E+00 | 9.94E-02 | 1.35E+01 |
| **319** | GO:0044057~regulation of system process | 41 | 0.1502988 | 7.92E-03 | 1185 | 309 | 13528 | 1.5147488 | 1.00E+00 | 9.96E-02 | 1.36E+01 |
| **320** | GO:0006103~2-oxoglutarate metabolic process | 5 | 0.0183291 | 7.97E-03 | 1185 | 10 | 13528 | 5.7080169 | 1.00E+00 | 9.99E-02 | 1.37E+01 |
| **321** | GO:0042273~ribosomal large subunit biogenesis | 5 | 0.0183291 | 7.97E-03 | 1185 | 10 | 13528 | 5.7080169 | 1.00E+00 | 9.99E-02 | 1.37E+01 |
| **322** | GO:0033700~phospholipid efflux | 5 | 0.0183291 | 7.97E-03 | 1185 | 10 | 13528 | 5.7080169 | 1.00E+00 | 9.99E-02 | 1.37E+01 |
| **323** | GO:0006417~regulation of translation | 22 | 0.0806481 | 7.99E-03 | 1185 | 137 | 13528 | 1.8332317 | 1.00E+00 | 9.98E-02 | 1.37E+01 |
| **324** | GO:0009408~response to heat | 12 | 0.0439899 | 8.36E-03 | 1185 | 56 | 13528 | 2.4462929 | 1.00E+00 | 1.04E-01 | 1.43E+01 |
| **325** | GO:0016042~lipid catabolic process | 26 | 0.0953114 | 8.65E-03 | 1185 | 173 | 13528 | 1.7157045 | 1.00E+00 | 1.07E-01 | 1.48E+01 |
| **326** | GO:0030837~negative regulation of actin filament polymerization | 8 | 0.0293266 | 8.89E-03 | 1185 | 28 | 13528 | 3.2617239 | 1.00E+00 | 1.09E-01 | 1.51E+01 |
| **327** | GO:0000226~microtubule cytoskeleton organization | 23 | 0.0843139 | 8.90E-03 | 1185 | 147 | 13528 | 1.7861822 | 1.00E+00 | 1.09E-01 | 1.52E+01 |
| **328** | GO:0070507~regulation of microtubule cytoskeleton organization | 10 | 0.0366582 | 9.26E-03 | 1185 | 42 | 13528 | 2.7181033 | 1.00E+00 | 1.13E-01 | 1.57E+01 |
| **329** | GO:0048738~cardiac muscle tissue development | 12 | 0.0439899 | 9.56E-03 | 1185 | 57 | 13528 | 2.4033755 | 1.00E+00 | 1.16E-01 | 1.62E+01 |
| **330** | GO:0006897~endocytosis | 31 | 0.1136405 | 9.80E-03 | 1185 | 220 | 13528 | 1.6086229 | 1.00E+00 | 1.18E-01 | 1.66E+01 |
| **331** | GO:0010324~membrane invagination | 31 | 0.1136405 | 9.80E-03 | 1185 | 220 | 13528 | 1.6086229 | 1.00E+00 | 1.18E-01 | 1.66E+01 |
| **332** | GO:0001959~regulation of cytokine-mediated signaling pathway | 6 | 0.0219949 | 9.81E-03 | 1185 | 16 | 13528 | 4.2810127 | 1.00E+00 | 1.18E-01 | 1.66E+01 |
| **333** | GO:0043691~reverse cholesterol transport | 6 | 0.0219949 | 9.81E-03 | 1185 | 16 | 13528 | 4.2810127 | 1.00E+00 | 1.18E-01 | 1.66E+01 |
| **334** | GO:0022604~regulation of cell morphogenesis | 21 | 0.0769823 | 9.91E-03 | 1185 | 131 | 13528 | 1.8300512 | 1.00E+00 | 1.19E-01 | 1.67E+01 |
| **335** | GO:0003018~vascular process in circulatory system | 12 | 0.0439899 | 1.09E-02 | 1185 | 58 | 13528 | 2.361938 | 1.00E+00 | 1.29E-01 | 1.82E+01 |
| **336** | GO:0042159~lipoprotein catabolic process | 4 | 0.0146633 | 1.09E-02 | 1185 | 6 | 13528 | 7.6106892 | 1.00E+00 | 1.29E-01 | 1.83E+01 |
| **337** | GO:0046031~ADP metabolic process | 4 | 0.0146633 | 1.09E-02 | 1185 | 6 | 13528 | 7.6106892 | 1.00E+00 | 1.29E-01 | 1.83E+01 |
| **338** | GO:0014866~skeletal myofibril assembly | 4 | 0.0146633 | 1.09E-02 | 1185 | 6 | 13528 | 7.6106892 | 1.00E+00 | 1.29E-01 | 1.83E+01 |
| **339** | GO:0070613~regulation of protein processing | 4 | 0.0146633 | 1.09E-02 | 1185 | 6 | 13528 | 7.6106892 | 1.00E+00 | 1.29E-01 | 1.83E+01 |
| **340** | GO:0010953~regulation of protein maturation by peptide bond cleavage | 4 | 0.0146633 | 1.09E-02 | 1185 | 6 | 13528 | 7.6106892 | 1.00E+00 | 1.29E-01 | 1.83E+01 |
| **341** | GO:0043277~apoptotic cell clearance | 4 | 0.0146633 | 1.09E-02 | 1185 | 6 | 13528 | 7.6106892 | 1.00E+00 | 1.29E-01 | 1.83E+01 |
| **342** | GO:0009068~aspartate family amino acid catabolic process | 4 | 0.0146633 | 1.09E-02 | 1185 | 6 | 13528 | 7.6106892 | 1.00E+00 | 1.29E-01 | 1.83E+01 |
| **343** | GO:0006729~tetrahydrobiopterin biosynthetic process | 4 | 0.0146633 | 1.09E-02 | 1185 | 6 | 13528 | 7.6106892 | 1.00E+00 | 1.29E-01 | 1.83E+01 |
| **344** | GO:0051918~negative regulation of fibrinolysis | 4 | 0.0146633 | 1.09E-02 | 1185 | 6 | 13528 | 7.6106892 | 1.00E+00 | 1.29E-01 | 1.83E+01 |
| **345** | GO:0006085~acetyl-CoA biosynthetic process | 4 | 0.0146633 | 1.09E-02 | 1185 | 6 | 13528 | 7.6106892 | 1.00E+00 | 1.29E-01 | 1.83E+01 |
| **346** | GO:0009062~fatty acid catabolic process | 9 | 0.0329924 | 1.11E-02 | 1185 | 36 | 13528 | 2.8540084 | 1.00E+00 | 1.31E-01 | 1.86E+01 |
| **347** | GO:0019218~regulation of steroid metabolic process | 9 | 0.0329924 | 1.11E-02 | 1185 | 36 | 13528 | 2.8540084 | 1.00E+00 | 1.31E-01 | 1.86E+01 |
| **348** | GO:0032870~cellular response to hormone stimulus | 21 | 0.0769823 | 1.16E-02 | 1185 | 133 | 13528 | 1.8025316 | 1.00E+00 | 1.36E-01 | 1.94E+01 |
| **349** | GO:0043524~negative regulation of neuron apoptosis | 11 | 0.0403241 | 1.18E-02 | 1185 | 51 | 13528 | 2.4622818 | 1.00E+00 | 1.38E-01 | 1.96E+01 |
| **350** | GO:0009112~nucleobase metabolic process | 7 | 0.0256608 | 1.22E-02 | 1185 | 23 | 13528 | 3.4744451 | 1.00E+00 | 1.42E-01 | 2.02E+01 |
| **351** | GO:0003015~heart process | 7 | 0.0256608 | 1.22E-02 | 1185 | 23 | 13528 | 3.4744451 | 1.00E+00 | 1.42E-01 | 2.02E+01 |
| **352** | GO:0030048~actin filament-based movement | 7 | 0.0256608 | 1.22E-02 | 1185 | 23 | 13528 | 3.4744451 | 1.00E+00 | 1.42E-01 | 2.02E+01 |
| **353** | GO:0042364~water-soluble vitamin biosynthetic process | 7 | 0.0256608 | 1.22E-02 | 1185 | 23 | 13528 | 3.4744451 | 1.00E+00 | 1.42E-01 | 2.02E+01 |
| **354** | GO:0060047~heart contraction | 7 | 0.0256608 | 1.22E-02 | 1185 | 23 | 13528 | 3.4744451 | 1.00E+00 | 1.42E-01 | 2.02E+01 |
| **355** | GO:0009266~response to temperature stimulus | 15 | 0.0549874 | 1.25E-02 | 1185 | 83 | 13528 | 2.0631386 | 1.00E+00 | 1.44E-01 | 2.06E+01 |
| **356** | GO:0010638~positive regulation of organelle organization | 15 | 0.0549874 | 1.25E-02 | 1185 | 83 | 13528 | 2.0631386 | 1.00E+00 | 1.44E-01 | 2.06E+01 |
| **357** | GO:0006323~DNA packaging | 19 | 0.0696506 | 1.29E-02 | 1185 | 117 | 13528 | 1.8538858 | 1.00E+00 | 1.48E-01 | 2.13E+01 |
| **358** | GO:0030301~cholesterol transport | 9 | 0.0329924 | 1.31E-02 | 1185 | 37 | 13528 | 2.7768731 | 1.00E+00 | 1.50E-01 | 2.15E+01 |
| **359** | GO:0015918~sterol transport | 9 | 0.0329924 | 1.31E-02 | 1185 | 37 | 13528 | 2.7768731 | 1.00E+00 | 1.50E-01 | 2.15E+01 |
| **360** | GO:0042493~response to drug | 30 | 0.1099747 | 1.34E-02 | 1185 | 216 | 13528 | 1.5855602 | 1.00E+00 | 1.53E-01 | 2.20E+01 |
| **361** | GO:0016050~vesicle organization | 11 | 0.0403241 | 1.35E-02 | 1185 | 52 | 13528 | 2.4149302 | 1.00E+00 | 1.53E-01 | 2.21E+01 |
| **362** | GO:0051291~protein heterooligomerization | 11 | 0.0403241 | 1.35E-02 | 1185 | 52 | 13528 | 2.4149302 | 1.00E+00 | 1.53E-01 | 2.21E+01 |
| **363** | GO:0030705~cytoskeleton-dependent intracellular transport | 11 | 0.0403241 | 1.35E-02 | 1185 | 52 | 13528 | 2.4149302 | 1.00E+00 | 1.53E-01 | 2.21E+01 |
| **364** | GO:0050727~regulation of inflammatory response | 14 | 0.0513215 | 1.40E-02 | 1185 | 76 | 13528 | 2.1029536 | 1.00E+00 | 1.58E-01 | 2.29E+01 |
| **365** | GO:0001568~blood vessel development | 33 | 0.1209722 | 1.43E-02 | 1185 | 245 | 13528 | 1.5376699 | 1.00E+00 | 1.60E-01 | 2.33E+01 |
| **366** | GO:0006397~mRNA processing | 41 | 0.1502988 | 1.45E-02 | 1185 | 321 | 13528 | 1.4581227 | 1.00E+00 | 1.62E-01 | 2.35E+01 |
| **367** | GO:0050873~brown fat cell differentiation | 7 | 0.0256608 | 1.51E-02 | 1185 | 24 | 13528 | 3.3296765 | 1.00E+00 | 1.68E-01 | 2.44E+01 |
| **368** | GO:0001906~cell killing | 7 | 0.0256608 | 1.51E-02 | 1185 | 24 | 13528 | 3.3296765 | 1.00E+00 | 1.68E-01 | 2.44E+01 |
| **369** | GO:0014706~striated muscle tissue development | 19 | 0.0696506 | 1.52E-02 | 1185 | 119 | 13528 | 1.8227281 | 1.00E+00 | 1.69E-01 | 2.46E+01 |
| **370** | GO:0002460~adaptive immune response based on somatic recombination of immune receptors built from immunoglobulin superfamily domains | 14 | 0.0513215 | 1.56E-02 | 1185 | 77 | 13528 | 2.0756425 | 1.00E+00 | 1.71E-01 | 2.51E+01 |
| **371** | GO:0002250~adaptive immune response | 14 | 0.0513215 | 1.56E-02 | 1185 | 77 | 13528 | 2.0756425 | 1.00E+00 | 1.71E-01 | 2.51E+01 |
| **372** | GO:0019319~hexose biosynthetic process | 8 | 0.0293266 | 1.57E-02 | 1185 | 31 | 13528 | 2.9460732 | 1.00E+00 | 1.72E-01 | 2.52E+01 |
| **373** | GO:0014823~response to activity | 5 | 0.0183291 | 1.63E-02 | 1185 | 12 | 13528 | 4.7566807 | 1.00E+00 | 1.78E-01 | 2.61E+01 |
| **374** | GO:0019216~regulation of lipid metabolic process | 18 | 0.0659848 | 1.75E-02 | 1185 | 112 | 13528 | 1.8347197 | 1.00E+00 | 1.89E-01 | 2.77E+01 |
| **375** | GO:0014070~response to organic cyclic substance | 19 | 0.0696506 | 1.79E-02 | 1185 | 121 | 13528 | 1.7926003 | 1.00E+00 | 1.92E-01 | 2.82E+01 |
| **376** | GO:0046148~pigment biosynthetic process | 9 | 0.0329924 | 1.79E-02 | 1185 | 39 | 13528 | 2.6344693 | 1.00E+00 | 1.92E-01 | 2.82E+01 |
| **377** | GO:0034440~lipid oxidation | 9 | 0.0329924 | 1.79E-02 | 1185 | 39 | 13528 | 2.6344693 | 1.00E+00 | 1.92E-01 | 2.82E+01 |
| **378** | GO:0019395~fatty acid oxidation | 9 | 0.0329924 | 1.79E-02 | 1185 | 39 | 13528 | 2.6344693 | 1.00E+00 | 1.92E-01 | 2.82E+01 |
| **379** | GO:0033627~cell adhesion mediated by integrin | 4 | 0.0146633 | 1.79E-02 | 1185 | 7 | 13528 | 6.5234479 | 1.00E+00 | 1.91E-01 | 2.82E+01 |
| **380** | GO:0045599~negative regulation of fat cell differentiation | 4 | 0.0146633 | 1.79E-02 | 1185 | 7 | 13528 | 6.5234479 | 1.00E+00 | 1.91E-01 | 2.82E+01 |
| **381** | GO:0051131~chaperone-mediated protein complex assembly | 4 | 0.0146633 | 1.79E-02 | 1185 | 7 | 13528 | 6.5234479 | 1.00E+00 | 1.91E-01 | 2.82E+01 |
| **382** | GO:0043206~fibril organization | 4 | 0.0146633 | 1.79E-02 | 1185 | 7 | 13528 | 6.5234479 | 1.00E+00 | 1.91E-01 | 2.82E+01 |
| **383** | GO:0046033~AMP metabolic process | 4 | 0.0146633 | 1.79E-02 | 1185 | 7 | 13528 | 6.5234479 | 1.00E+00 | 1.91E-01 | 2.82E+01 |
| **384** | GO:0019430~removal of superoxide radicals | 4 | 0.0146633 | 1.79E-02 | 1185 | 7 | 13528 | 6.5234479 | 1.00E+00 | 1.91E-01 | 2.82E+01 |
| **385** | GO:0051238~sequestering of metal ion | 4 | 0.0146633 | 1.79E-02 | 1185 | 7 | 13528 | 6.5234479 | 1.00E+00 | 1.91E-01 | 2.82E+01 |
| **386** | GO:0046146~tetrahydrobiopterin metabolic process | 4 | 0.0146633 | 1.79E-02 | 1185 | 7 | 13528 | 6.5234479 | 1.00E+00 | 1.91E-01 | 2.82E+01 |
| **387** | GO:0009161~ribonucleoside monophosphate metabolic process | 7 | 0.0256608 | 1.85E-02 | 1185 | 25 | 13528 | 3.1964895 | 1.00E+00 | 1.96E-01 | 2.90E+01 |
| **388** | GO:0001936~regulation of endothelial cell proliferation | 8 | 0.0293266 | 1.86E-02 | 1185 | 32 | 13528 | 2.8540084 | 1.00E+00 | 1.97E-01 | 2.92E+01 |
| **389** | GO:0022402~cell cycle process | 65 | 0.2382785 | 1.92E-02 | 1185 | 565 | 13528 | 1.313349 | 1.00E+00 | 2.02E-01 | 3.00E+01 |
| **390** | GO:0051494~negative regulation of cytoskeleton organization | 11 | 0.0403241 | 1.97E-02 | 1185 | 55 | 13528 | 2.2832068 | 1.00E+00 | 2.06E-01 | 3.06E+01 |
| **391** | GO:0001944~vasculature development | 33 | 0.1209722 | 1.97E-02 | 1185 | 251 | 13528 | 1.5009128 | 1.00E+00 | 2.06E-01 | 3.07E+01 |
| **392** | GO:0051146~striated muscle cell differentiation | 15 | 0.0549874 | 2.02E-02 | 1185 | 88 | 13528 | 1.9459148 | 1.00E+00 | 2.09E-01 | 3.13E+01 |
| **393** | GO:0032989~cellular component morphogenesis | 48 | 0.1759595 | 2.04E-02 | 1185 | 397 | 13528 | 1.3802761 | 1.00E+00 | 2.11E-01 | 3.16E+01 |
| **394** | GO:0006767~water-soluble vitamin metabolic process | 9 | 0.0329924 | 2.07E-02 | 1185 | 40 | 13528 | 2.5686076 | 1.00E+00 | 2.13E-01 | 3.19E+01 |
| **395** | GO:0033344~cholesterol efflux | 6 | 0.0219949 | 2.10E-02 | 1185 | 19 | 13528 | 3.6050633 | 1.00E+00 | 2.15E-01 | 3.23E+01 |
| **396** | GO:0019322~pentose biosynthetic process | 3 | 0.0109975 | 2.16E-02 | 1185 | 3 | 13528 | 11.416034 | 1.00E+00 | 2.20E-01 | 3.31E+01 |
| **397** | GO:0046100~hypoxanthine metabolic process | 3 | 0.0109975 | 2.16E-02 | 1185 | 3 | 13528 | 11.416034 | 1.00E+00 | 2.20E-01 | 3.31E+01 |
| **398** | GO:0035305~negative regulation of dephosphorylation | 3 | 0.0109975 | 2.16E-02 | 1185 | 3 | 13528 | 11.416034 | 1.00E+00 | 2.20E-01 | 3.31E+01 |
| **399** | GO:0019693~ribose phosphate metabolic process | 3 | 0.0109975 | 2.16E-02 | 1185 | 3 | 13528 | 11.416034 | 1.00E+00 | 2.20E-01 | 3.31E+01 |
| **400** | GO:0006086~acetyl-CoA biosynthetic process from pyruvate | 3 | 0.0109975 | 2.16E-02 | 1185 | 3 | 13528 | 11.416034 | 1.00E+00 | 2.20E-01 | 3.31E+01 |
| **401** | GO:0042819~vitamin B6 biosynthetic process | 3 | 0.0109975 | 2.16E-02 | 1185 | 3 | 13528 | 11.416034 | 1.00E+00 | 2.20E-01 | 3.31E+01 |
| **402** | GO:0006106~fumarate metabolic process | 3 | 0.0109975 | 2.16E-02 | 1185 | 3 | 13528 | 11.416034 | 1.00E+00 | 2.20E-01 | 3.31E+01 |
| **403** | GO:0008614~pyridoxine metabolic process | 3 | 0.0109975 | 2.16E-02 | 1185 | 3 | 13528 | 11.416034 | 1.00E+00 | 2.20E-01 | 3.31E+01 |
| **404** | GO:0010903~negative regulation of very-low-density lipoprotein particle remodeling | 3 | 0.0109975 | 2.16E-02 | 1185 | 3 | 13528 | 11.416034 | 1.00E+00 | 2.20E-01 | 3.31E+01 |
| **405** | GO:0033240~positive regulation of cellular amine metabolic process | 3 | 0.0109975 | 2.16E-02 | 1185 | 3 | 13528 | 11.416034 | 1.00E+00 | 2.20E-01 | 3.31E+01 |
| **406** | GO:0035308~negative regulation of protein amino acid dephosphorylation | 3 | 0.0109975 | 2.16E-02 | 1185 | 3 | 13528 | 11.416034 | 1.00E+00 | 2.20E-01 | 3.31E+01 |
| **407** | GO:0008615~pyridoxine biosynthetic process | 3 | 0.0109975 | 2.16E-02 | 1185 | 3 | 13528 | 11.416034 | 1.00E+00 | 2.20E-01 | 3.31E+01 |
| **408** | GO:0042816~vitamin B6 metabolic process | 3 | 0.0109975 | 2.16E-02 | 1185 | 3 | 13528 | 11.416034 | 1.00E+00 | 2.20E-01 | 3.31E+01 |
| **409** | GO:0010563~negative regulation of phosphorus metabolic process | 10 | 0.0366582 | 2.18E-02 | 1185 | 48 | 13528 | 2.3783404 | 1.00E+00 | 2.21E-01 | 3.33E+01 |
| **410** | GO:0045936~negative regulation of phosphate metabolic process | 10 | 0.0366582 | 2.18E-02 | 1185 | 48 | 13528 | 2.3783404 | 1.00E+00 | 2.21E-01 | 3.33E+01 |
| **411** | GO:0051346~negative regulation of hydrolase activity | 10 | 0.0366582 | 2.18E-02 | 1185 | 48 | 13528 | 2.3783404 | 1.00E+00 | 2.21E-01 | 3.33E+01 |
| **412** | GO:0006939~smooth muscle contraction | 8 | 0.0293266 | 2.19E-02 | 1185 | 33 | 13528 | 2.7675233 | 1.00E+00 | 2.21E-01 | 3.34E+01 |
| **413** | GO:0009116~nucleoside metabolic process | 12 | 0.0439899 | 2.20E-02 | 1185 | 64 | 13528 | 2.1405063 | 1.00E+00 | 2.22E-01 | 3.36E+01 |
| **414** | GO:0031344~regulation of cell projection organization | 15 | 0.0549874 | 2.21E-02 | 1185 | 89 | 13528 | 1.9240506 | 1.00E+00 | 2.22E-01 | 3.37E+01 |
| **415** | GO:0048771~tissue remodeling | 11 | 0.0403241 | 2.21E-02 | 1185 | 56 | 13528 | 2.2424352 | 1.00E+00 | 2.22E-01 | 3.38E+01 |
| **416** | GO:0050801~ion homeostasis | 49 | 0.1796254 | 2.23E-02 | 1185 | 409 | 13528 | 1.3676911 | 1.00E+00 | 2.22E-01 | 3.39E+01 |
| **417** | GO:0018149~peptide cross-linking | 7 | 0.0256608 | 2.23E-02 | 1185 | 26 | 13528 | 3.0735475 | 1.00E+00 | 2.22E-01 | 3.39E+01 |
| **418** | GO:0006695~cholesterol biosynthetic process | 7 | 0.0256608 | 2.23E-02 | 1185 | 26 | 13528 | 3.0735475 | 1.00E+00 | 2.22E-01 | 3.39E+01 |
| **419** | GO:0016051~carbohydrate biosynthetic process | 17 | 0.062319 | 2.35E-02 | 1185 | 107 | 13528 | 1.8137624 | 1.00E+00 | 2.32E-01 | 3.55E+01 |
| **420** | GO:0060537~muscle tissue development | 19 | 0.0696506 | 2.42E-02 | 1185 | 125 | 13528 | 1.7352371 | 1.00E+00 | 2.37E-01 | 3.62E+01 |
| **421** | GO:0032886~regulation of microtubule-based process | 10 | 0.0366582 | 2.47E-02 | 1185 | 49 | 13528 | 2.3298028 | 1.00E+00 | 2.41E-01 | 3.68E+01 |
| **422** | GO:0030336~negative regulation of cell migration | 11 | 0.0403241 | 2.48E-02 | 1185 | 57 | 13528 | 2.2030942 | 1.00E+00 | 2.42E-01 | 3.70E+01 |
| **423** | GO:0010639~negative regulation of organelle organization | 14 | 0.0513215 | 2.53E-02 | 1185 | 82 | 13528 | 1.9490789 | 1.00E+00 | 2.45E-01 | 3.76E+01 |
| **424** | GO:0034368~protein-lipid complex remodeling | 6 | 0.0219949 | 2.60E-02 | 1185 | 20 | 13528 | 3.4248101 | 1.00E+00 | 2.50E-01 | 3.84E+01 |
| **425** | GO:0034369~plasma lipoprotein particle remodeling | 6 | 0.0219949 | 2.60E-02 | 1185 | 20 | 13528 | 3.4248101 | 1.00E+00 | 2.50E-01 | 3.84E+01 |
| **426** | GO:0032371~regulation of sterol transport | 6 | 0.0219949 | 2.60E-02 | 1185 | 20 | 13528 | 3.4248101 | 1.00E+00 | 2.50E-01 | 3.84E+01 |
| **427** | GO:0048678~response to axon injury | 6 | 0.0219949 | 2.60E-02 | 1185 | 20 | 13528 | 3.4248101 | 1.00E+00 | 2.50E-01 | 3.84E+01 |
| **428** | GO:0034367~macromolecular complex remodeling | 6 | 0.0219949 | 2.60E-02 | 1185 | 20 | 13528 | 3.4248101 | 1.00E+00 | 2.50E-01 | 3.84E+01 |
| **429** | GO:0032374~regulation of cholesterol transport | 6 | 0.0219949 | 2.60E-02 | 1185 | 20 | 13528 | 3.4248101 | 1.00E+00 | 2.50E-01 | 3.84E+01 |
| **430** | GO:0006886~intracellular protein transport | 45 | 0.1649621 | 2.67E-02 | 1185 | 374 | 13528 | 1.3735869 | 1.00E+00 | 2.55E-01 | 3.92E+01 |
| **431** | GO:0007016~cytoskeletal anchoring at plasma membrane | 4 | 0.0146633 | 2.68E-02 | 1185 | 8 | 13528 | 5.7080169 | 1.00E+00 | 2.56E-01 | 3.93E+01 |
| **432** | GO:0006734~NADH metabolic process | 4 | 0.0146633 | 2.68E-02 | 1185 | 8 | 13528 | 5.7080169 | 1.00E+00 | 2.56E-01 | 3.93E+01 |
| **433** | GO:0009163~nucleoside biosynthetic process | 4 | 0.0146633 | 2.68E-02 | 1185 | 8 | 13528 | 5.7080169 | 1.00E+00 | 2.56E-01 | 3.93E+01 |
| **434** | GO:0046129~purine ribonucleoside biosynthetic process | 4 | 0.0146633 | 2.68E-02 | 1185 | 8 | 13528 | 5.7080169 | 1.00E+00 | 2.56E-01 | 3.93E+01 |
| **435** | GO:0042451~purine nucleoside biosynthetic process | 4 | 0.0146633 | 2.68E-02 | 1185 | 8 | 13528 | 5.7080169 | 1.00E+00 | 2.56E-01 | 3.93E+01 |
| **436** | GO:0060343~trabecula formation | 4 | 0.0146633 | 2.68E-02 | 1185 | 8 | 13528 | 5.7080169 | 1.00E+00 | 2.56E-01 | 3.93E+01 |
| **437** | GO:0042455~ribonucleoside biosynthetic process | 4 | 0.0146633 | 2.68E-02 | 1185 | 8 | 13528 | 5.7080169 | 1.00E+00 | 2.56E-01 | 3.93E+01 |
| **438** | GO:0008217~regulation of blood pressure | 16 | 0.0586532 | 2.70E-02 | 1185 | 100 | 13528 | 1.8265654 | 1.00E+00 | 2.57E-01 | 3.96E+01 |
| **439** | GO:0006892~post-Golgi vesicle-mediated transport | 11 | 0.0403241 | 2.77E-02 | 1185 | 58 | 13528 | 2.1651099 | 1.00E+00 | 2.62E-01 | 4.04E+01 |
| **440** | GO:0006928~cell motion | 55 | 0.2016203 | 2.78E-02 | 1185 | 475 | 13528 | 1.3218565 | 1.00E+00 | 2.62E-01 | 4.05E+01 |
| **441** | GO:0006333~chromatin assembly or disassembly | 19 | 0.0696506 | 2.79E-02 | 1185 | 127 | 13528 | 1.7079106 | 1.00E+00 | 2.62E-01 | 4.05E+01 |
| **442** | GO:0009991~response to extracellular stimulus | 29 | 0.1063089 | 2.83E-02 | 1185 | 220 | 13528 | 1.5048408 | 1.00E+00 | 2.65E-01 | 4.10E+01 |
| **443** | GO:0046716~muscle maintenance | 5 | 0.0183291 | 2.87E-02 | 1185 | 14 | 13528 | 4.0771549 | 1.00E+00 | 2.67E-01 | 4.14E+01 |
| **444** | GO:0045940~positive regulation of steroid metabolic process | 5 | 0.0183291 | 2.87E-02 | 1185 | 14 | 13528 | 4.0771549 | 1.00E+00 | 2.67E-01 | 4.14E+01 |
| **445** | GO:0007263~nitric oxide mediated signal transduction | 5 | 0.0183291 | 2.87E-02 | 1185 | 14 | 13528 | 4.0771549 | 1.00E+00 | 2.67E-01 | 4.14E+01 |
| **446** | GO:0046112~nucleobase biosynthetic process | 5 | 0.0183291 | 2.87E-02 | 1185 | 14 | 13528 | 4.0771549 | 1.00E+00 | 2.67E-01 | 4.14E+01 |
| **447** | GO:0030865~cortical cytoskeleton organization | 5 | 0.0183291 | 2.87E-02 | 1185 | 14 | 13528 | 4.0771549 | 1.00E+00 | 2.67E-01 | 4.14E+01 |
| **448** | GO:0006144~purine base metabolic process | 5 | 0.0183291 | 2.87E-02 | 1185 | 14 | 13528 | 4.0771549 | 1.00E+00 | 2.67E-01 | 4.14E+01 |
| **449** | GO:0005977~glycogen metabolic process | 8 | 0.0293266 | 2.96E-02 | 1185 | 35 | 13528 | 2.6093791 | 1.00E+00 | 2.74E-01 | 4.24E+01 |
| **450** | GO:0001505~regulation of neurotransmitter levels | 12 | 0.0439899 | 3.00E-02 | 1185 | 67 | 13528 | 2.0446628 | 1.00E+00 | 2.76E-01 | 4.29E+01 |
| **451** | GO:0018130~heterocycle biosynthetic process | 11 | 0.0403241 | 3.09E-02 | 1185 | 59 | 13528 | 2.1284131 | 1.00E+00 | 2.82E-01 | 4.38E+01 |
| **452** | GO:0007162~negative regulation of cell adhesion | 9 | 0.0329924 | 3.10E-02 | 1185 | 43 | 13528 | 2.3894024 | 1.00E+00 | 2.82E-01 | 4.39E+01 |
| **453** | GO:0006112~energy reserve metabolic process | 9 | 0.0329924 | 3.10E-02 | 1185 | 43 | 13528 | 2.3894024 | 1.00E+00 | 2.82E-01 | 4.39E+01 |
| **454** | GO:0010811~positive regulation of cell-substrate adhesion | 7 | 0.0256608 | 3.14E-02 | 1185 | 28 | 13528 | 2.8540084 | 1.00E+00 | 2.85E-01 | 4.44E+01 |
| **455** | GO:0009110~vitamin biosynthetic process | 7 | 0.0256608 | 3.14E-02 | 1185 | 28 | 13528 | 2.8540084 | 1.00E+00 | 2.85E-01 | 4.44E+01 |
| **456** | GO:0006890~retrograde vesicle-mediated transport, Golgi to ER | 6 | 0.0219949 | 3.17E-02 | 1185 | 21 | 13528 | 3.2617239 | 1.00E+00 | 2.87E-01 | 4.47E+01 |
| **457** | GO:0019835~cytolysis | 6 | 0.0219949 | 3.17E-02 | 1185 | 21 | 13528 | 3.2617239 | 1.00E+00 | 2.87E-01 | 4.47E+01 |
| **458** | GO:0007005~mitochondrion organization | 20 | 0.0733165 | 3.18E-02 | 1185 | 138 | 13528 | 1.6544976 | 1.00E+00 | 2.87E-01 | 4.48E+01 |
| **459** | GO:0006073~cellular glucan metabolic process | 8 | 0.0293266 | 3.41E-02 | 1185 | 36 | 13528 | 2.5368964 | 1.00E+00 | 3.03E-01 | 4.71E+01 |
| **460** | GO:0031348~negative regulation of defense response | 8 | 0.0293266 | 3.41E-02 | 1185 | 36 | 13528 | 2.5368964 | 1.00E+00 | 3.03E-01 | 4.71E+01 |
| **461** | GO:0044042~glucan metabolic process | 8 | 0.0293266 | 3.41E-02 | 1185 | 36 | 13528 | 2.5368964 | 1.00E+00 | 3.03E-01 | 4.71E+01 |

**Supplementary Table S2: (continued)**

| | | | | | | | | | | | |
|---|---|---|---|---|---|---|---|---|---|---|---|
| **462** | GO:0009743~response to carbohydrate stimulus | 11 | 0.0403241 | 3.42E-02 | 1185 | 60 | 13528 | 2.0929395 | 1.00E+00 | 3.04E-01 | 4.73E+01 |
| **463** | GO:0010769~regulation of cell morphogenesis involved in differentiation | 13 | 0.0476557 | 3.47E-02 | 1185 | 77 | 13528 | 1.9273823 | 1.00E+00 | 3.07E-01 | 4.78E+01 |
| **464** | GO:0009057~macromolecule catabolic process | 84 | 0.3079292 | 3.47E-02 | 1185 | 781 | 13528 | 1.2278449 | 1.00E+00 | 3.06E-01 | 4.78E+01 |
| **465** | GO:0048878~chemical homeostasis | 58 | 0.2126178 | 3.48E-02 | 1185 | 512 | 13528 | 1.2932226 | 1.00E+00 | 3.06E-01 | 4.79E+01 |
| **466** | GO:0055002~striated muscle cell development | 10 | 0.0366582 | 3.50E-02 | 1185 | 52 | 13528 | 2.1953911 | 1.00E+00 | 3.07E-01 | 4.80E+01 |
| **467** | GO:0009064~glutamine family amino acid metabolic process | 10 | 0.0366582 | 3.50E-02 | 1185 | 52 | 13528 | 2.1953911 | 1.00E+00 | 3.07E-01 | 4.80E+01 |
| **468** | GO:0034613~cellular protein localization | 48 | 0.1759595 | 3.51E-02 | 1185 | 411 | 13528 | 1.3332594 | 1.00E+00 | 3.06E-01 | 4.81E+01 |
| **469** | GO:0052547~regulation of peptidase activity | 14 | 0.0513215 | 3.58E-02 | 1185 | 86 | 13528 | 1.8584241 | 1.00E+00 | 3.11E-01 | 4.88E+01 |
| **470** | GO:0007584~response to nutrient | 20 | 0.0733165 | 3.62E-02 | 1185 | 140 | 13528 | 1.630862 | 1.00E+00 | 3.13E-01 | 4.92E+01 |
| **471** | GO:0006901~vesicle coating | 5 | 0.0183291 | 3.64E-02 | 1185 | 15 | 13528 | 3.8053446 | 1.00E+00 | 3.14E-01 | 4.95E+01 |
| **472** | GO:0043489~RNA stabilization | 5 | 0.0183291 | 3.64E-02 | 1185 | 15 | 13528 | 3.8053446 | 1.00E+00 | 3.14E-01 | 4.95E+01 |
| **473** | GO:0045806~negative regulation of endocytosis | 5 | 0.0183291 | 3.64E-02 | 1185 | 15 | 13528 | 3.8053446 | 1.00E+00 | 3.14E-01 | 4.95E+01 |
| **474** | GO:0048255~mRNA stabilization | 5 | 0.0183291 | 3.64E-02 | 1185 | 15 | 13528 | 3.8053446 | 1.00E+00 | 3.14E-01 | 4.95E+01 |
| **475** | GO:0032369~negative regulation of lipid transport | 5 | 0.0183291 | 3.64E-02 | 1185 | 15 | 13528 | 3.8053446 | 1.00E+00 | 3.14E-01 | 4.95E+01 |
| **476** | GO:0030003~cellular cation homeostasis | 32 | 0.1173064 | 3.65E-02 | 1185 | 254 | 13528 | 1.4382405 | 1.00E+00 | 3.14E-01 | 4.95E+01 |
| **477** | GO:0042278~purine nucleoside metabolic process | 7 | 0.0256608 | 3.67E-02 | 1185 | 29 | 13528 | 2.7555944 | 1.00E+00 | 3.15E-01 | 4.98E+01 |
| **478** | GO:0046128~purine ribonucleoside metabolic process | 7 | 0.0256608 | 3.67E-02 | 1185 | 29 | 13528 | 2.7555944 | 1.00E+00 | 3.15E-01 | 4.98E+01 |
| **479** | GO:0045807~positive regulation of endocytosis | 7 | 0.0256608 | 3.67E-02 | 1185 | 29 | 13528 | 2.7555944 | 1.00E+00 | 3.15E-01 | 4.98E+01 |
| **480** | GO:0042254~ribosome biogenesis | 18 | 0.0659848 | 3.68E-02 | 1185 | 122 | 13528 | 1.6843328 | 1.00E+00 | 3.15E-01 | 4.99E+01 |
| **481** | GO:0006599~phosphagen metabolic process | 4 | 0.0146633 | 3.76E-02 | 1185 | 9 | 13528 | 5.0737928 | 1.00E+00 | 3.20E-01 | 5.06E+01 |
| **482** | GO:0006600~creatine metabolic process | 4 | 0.0146633 | 3.76E-02 | 1185 | 9 | 13528 | 5.0737928 | 1.00E+00 | 3.20E-01 | 5.06E+01 |
| **483** | GO:0033275~actin-myosin filament sliding | 4 | 0.0146633 | 3.76E-02 | 1185 | 9 | 13528 | 5.0737928 | 1.00E+00 | 3.20E-01 | 5.06E+01 |
| **484** | GO:0030049~muscle filament sliding | 4 | 0.0146633 | 3.76E-02 | 1185 | 9 | 13528 | 5.0737928 | 1.00E+00 | 3.20E-01 | 5.06E+01 |
| **485** | GO:0070252~actin-mediated cell contraction | 4 | 0.0146633 | 3.76E-02 | 1185 | 9 | 13528 | 5.0737928 | 1.00E+00 | 3.20E-01 | 5.06E+01 |
| **486** | GO:0030212~hyaluronan metabolic process | 4 | 0.0146633 | 3.76E-02 | 1185 | 9 | 13528 | 5.0737928 | 1.00E+00 | 3.20E-01 | 5.06E+01 |
| **487** | GO:0040013~negative regulation of locomotion | 11 | 0.0403241 | 3.79E-02 | 1185 | 61 | 13528 | 2.058629 | 1.00E+00 | 3.21E-01 | 5.08E+01 |
| **488** | GO:0006942~regulation of striated muscle contraction | 6 | 0.0219949 | 3.82E-02 | 1185 | 22 | 13528 | 3.1134638 | 1.00E+00 | 3.22E-01 | 5.11E+01 |
| **489** | GO:0006282~regulation of DNA repair | 6 | 0.0219949 | 3.82E-02 | 1185 | 22 | 13528 | 3.1134638 | 1.00E+00 | 3.22E-01 | 5.11E+01 |
| **490** | GO:0045103~intermediate filament-based process | 6 | 0.0219949 | 3.82E-02 | 1185 | 22 | 13528 | 3.1134638 | 1.00E+00 | 3.22E-01 | 5.11E+01 |
| **491** | GO:0043488~regulation of mRNA stability | 6 | 0.0219949 | 3.82E-02 | 1185 | 22 | 13528 | 3.1134638 | 1.00E+00 | 3.22E-01 | 5.11E+01 |
| **492** | GO:0048584~positive regulation of response to stimulus | 30 | 0.1099747 | 3.89E-02 | 1185 | 236 | 13528 | 1.4511907 | 1.00E+00 | 3.27E-01 | 5.18E+01 |
| **493** | GO:0070727~cellular macromolecule localization | 48 | 0.1759595 | 3.90E-02 | 1185 | 414 | 13528 | 1.3235981 | 1.00E+00 | 3.27E-01 | 5.19E+01 |
| **494** | GO:0006631~fatty acid metabolic process | 26 | 0.0953114 | 3.98E-02 | 1185 | 198 | 13528 | 1.4990751 | 1.00E+00 | 3.32E-01 | 5.26E+01 |
| **495** | GO:0010975~regulation of neuron projection development | 12 | 0.0439899 | 3.99E-02 | 1185 | 70 | 13528 | 1.9570344 | 1.00E+00 | 3.31E-01 | 5.27E+01 |
| **496** | GO:0007229~integrin-mediated signaling pathway | 12 | 0.0439899 | 3.99E-02 | 1185 | 70 | 13528 | 1.9570344 | 1.00E+00 | 3.31E-01 | 5.27E+01 |
| **497** | GO:0006533~aspartate catabolic process | 3 | 0.0109975 | 4.07E-02 | 1185 | 4 | 13528 | 8.5620253 | 1.00E+00 | 3.37E-01 | 5.35E+01 |
| **498** | GO:0033631~cell-cell adhesion mediated by integrin | 3 | 0.0109975 | 4.07E-02 | 1185 | 4 | 13528 | 8.5620253 | 1.00E+00 | 3.37E-01 | 5.35E+01 |
| **499** | GO:0060334~regulation of interferon-gamma-mediated signaling pathway | 3 | 0.0109975 | 4.07E-02 | 1185 | 4 | 13528 | 8.5620253 | 1.00E+00 | 3.37E-01 | 5.35E+01 |
| **500** | GO:0032488~Cdc42 protein signal transduction | 3 | 0.0109975 | 4.07E-02 | 1185 | 4 | 13528 | 8.5620253 | 1.00E+00 | 3.37E-01 | 5.35E+01 |
| **501** | GO:0006531~aspartate metabolic process | 3 | 0.0109975 | 4.07E-02 | 1185 | 4 | 13528 | 8.5620253 | 1.00E+00 | 3.37E-01 | 5.35E+01 |
| **502** | GO:0006152~purine nucleoside catabolic process | 3 | 0.0109975 | 4.07E-02 | 1185 | 4 | 13528 | 8.5620253 | 1.00E+00 | 3.37E-01 | 5.35E+01 |
| **503** | GO:0006104~succinyl-CoA metabolic process | 3 | 0.0109975 | 4.07E-02 | 1185 | 4 | 13528 | 8.5620253 | 1.00E+00 | 3.37E-01 | 5.35E+01 |
| **504** | GO:0006907~pinocytosis | 3 | 0.0109975 | 4.07E-02 | 1185 | 4 | 13528 | 8.5620253 | 1.00E+00 | 3.37E-01 | 5.35E+01 |
| **505** | GO:0010955~negative regulation of protein maturation by peptide bond cleavage | 3 | 0.0109975 | 4.07E-02 | 1185 | 4 | 13528 | 8.5620253 | 1.00E+00 | 3.37E-01 | 5.35E+01 |
| **506** | GO:0046130~purine ribonucleoside catabolic process | 3 | 0.0109975 | 4.07E-02 | 1185 | 4 | 13528 | 8.5620253 | 1.00E+00 | 3.37E-01 | 5.35E+01 |
| **507** | GO:0022613~ribonucleoprotein complex biogenesis | 24 | 0.0879798 | 4.21E-02 | 1185 | 180 | 13528 | 1.5221378 | 1.00E+00 | 3.45E-01 | 5.46E+01 |
| **508** | GO:0045834~positive regulation of lipid metabolic process | 9 | 0.0329924 | 4.43E-02 | 1185 | 46 | 13528 | 2.2335718 | 1.00E+00 | 3.59E-01 | 5.65E+01 |
| **509** | GO:0009119~ribonucleoside metabolic process | 9 | 0.0329924 | 4.43E-02 | 1185 | 46 | 13528 | 2.2335718 | 1.00E+00 | 3.59E-01 | 5.65E+01 |
| **510** | GO:0006458~'de novo' protein folding | 5 | 0.0183291 | 4.53E-02 | 1185 | 16 | 13528 | 3.5675105 | 1.00E+00 | 3.65E-01 | 5.74E+01 |
| **511** | GO:0050764~regulation of phagocytosis | 5 | 0.0183291 | 4.53E-02 | 1185 | 16 | 13528 | 3.5675105 | 1.00E+00 | 3.65E-01 | 5.74E+01 |
| **512** | GO:0030032~lamellipodium assembly | 5 | 0.0183291 | 4.53E-02 | 1185 | 16 | 13528 | 3.5675105 | 1.00E+00 | 3.65E-01 | 5.74E+01 |
| **513** | GO:0006900~membrane budding | 5 | 0.0183291 | 4.53E-02 | 1185 | 16 | 13528 | 3.5675105 | 1.00E+00 | 3.65E-01 | 5.74E+01 |
| **514** | GO:0048545~response to steroid hormone stimulus | 25 | 0.0916456 | 4.78E-02 | 1185 | 192 | 13528 | 1.4864627 | 1.00E+00 | 3.80E-01 | 5.94E+01 |
| **515** | GO:0009309~amine biosynthetic process | 13 | 0.0476557 | 4.86E-02 | 1185 | 81 | 13528 | 1.8322029 | 1.00E+00 | 3.84E-01 | 6.00E+01 |
| **516** | GO:0031346~positive regulation of cell projection organization | 9 | 0.0329924 | 4.94E-02 | 1185 | 47 | 13528 | 2.186049 | 1.00E+00 | 3.89E-01 | 6.06E+01 |
| **517** | GO:0000303~response to superoxide | 4 | 0.0146633 | 5.03E-02 | 1185 | 10 | 13528 | 4.5664135 | 1.00E+00 | 3.93E-01 | 6.13E+01 |
| **518** | GO:0018279~protein amino acid N-linked glycosylation via asparagine | 4 | 0.0146633 | 5.03E-02 | 1185 | 10 | 13528 | 4.5664135 | 1.00E+00 | 3.93E-01 | 6.13E+01 |
| **519** | GO:0001909~leukocyte mediated cytotoxicity | 4 | 0.0146633 | 5.03E-02 | 1185 | 10 | 13528 | 4.5664135 | 1.00E+00 | 3.93E-01 | 6.13E+01 |
| **520** | GO:0018196~peptidyl-asparagine modification | 4 | 0.0146633 | 5.03E-02 | 1185 | 10 | 13528 | 4.5664135 | 1.00E+00 | 3.93E-01 | 6.13E+01 |
| **521** | GO:0048585~negative regulation of response to stimulus | 15 | 0.0549874 | 5.28E-02 | 1185 | 100 | 13528 | 1.7124051 | 1.00E+00 | 4.08E-01 | 6.31E+01 |
| **522** | GO:0055001~muscle cell development | 10 | 0.0366582 | 5.28E-02 | 1185 | 56 | 13528 | 2.0385775 | 1.00E+00 | 4.07E-01 | 6.32E+01 |
| **523** | GO:0043487~regulation of RNA stability | 6 | 0.0219949 | 5.33E-02 | 1185 | 24 | 13528 | 2.8540084 | 1.00E+00 | 4.09E-01 | 6.35E+01 |
| **524** | GO:0006396~RNA processing | 60 | 0.2199494 | 5.42E-02 | 1185 | 547 | 13528 | 1.2522158 | 1.00E+00 | 4.14E-01 | 6.41E+01 |
| **525** | GO:0051004~regulation of lipoprotein lipase activity | 5 | 0.0183291 | 5.53E-02 | 1185 | 17 | 13528 | 3.357657 | 1.00E+00 | 4.19E-01 | 6.49E+01 |
| **526** | GO:0042310~vasoconstriction | 5 | 0.0183291 | 5.53E-02 | 1185 | 17 | 13528 | 3.357657 | 1.00E+00 | 4.19E-01 | 6.49E+01 |
| **527** | GO:0034199~activation of protein kinase A activity | 5 | 0.0183291 | 5.53E-02 | 1185 | 17 | 13528 | 3.357657 | 1.00E+00 | 4.19E-01 | 6.49E+01 |
| **528** | GO:0044265~cellular macromolecule catabolic process | 77 | 0.2822684 | 5.53E-02 | 1185 | 725 | 13528 | 1.2124615 | 1.00E+00 | 4.19E-01 | 6.49E+01 |
| **529** | GO:0001933~negative regulation of protein amino acid phosphorylation | 7 | 0.0256608 | 5.62E-02 | 1185 | 32 | 13528 | 2.4972574 | 1.00E+00 | 4.23E-01 | 6.55E+01 |
| **530** | GO:0030168~platelet activation | 7 | 0.0256608 | 5.62E-02 | 1185 | 32 | 13528 | 2.4972574 | 1.00E+00 | 4.23E-01 | 6.55E+01 |
| **531** | GO:0010876~lipid localization | 21 | 0.0769823 | 5.66E-02 | 1185 | 157 | 13528 | 1.5269854 | 1.00E+00 | 4.24E-01 | 6.57E+01 |
| **532** | GO:0030031~cell projection assembly | 13 | 0.0476557 | 5.68E-02 | 1185 | 83 | 13528 | 1.7880535 | 1.00E+00 | 4.25E-01 | 6.59E+01 |
| **533** | GO:0030163~protein catabolic process | 67 | 0.2456102 | 5.77E-02 | 1185 | 622 | 13528 | 1.2297014 | 1.00E+00 | 4.29E-01 | 6.65E+01 |
| **534** | GO:0050770~regulation of axonogenesis | 10 | 0.0366582 | 5.81E-02 | 1185 | 57 | 13528 | 2.0028129 | 1.00E+00 | 4.30E-01 | 6.67E+01 |
| **535** | GO:0005976~polysaccharide metabolic process | 16 | 0.0586532 | 5.97E-02 | 1185 | 111 | 13528 | 1.6455544 | 1.00E+00 | 4.39E-01 | 6.78E+01 |
| **536** | GO:0006766~vitamin metabolic process | 12 | 0.0439899 | 6.10E-02 | 1185 | 75 | 13528 | 1.8265654 | 1.00E+00 | 4.45E-01 | 6.85E+01 |
| **537** | GO:0031667~response to nutrient levels | 25 | 0.0916456 | 6.10E-02 | 1185 | 197 | 13528 | 1.4487352 | 1.00E+00 | 4.44E-01 | 6.85E+01 |
| **538** | GO:0010743~regulation of foam cell differentiation | 6 | 0.0219949 | 6.21E-02 | 1185 | 25 | 13528 | 2.7398481 | 1.00E+00 | 4.49E-01 | 6.92E+01 |
| **539** | GO:0019674~NAD metabolic process | 6 | 0.0219949 | 6.21E-02 | 1185 | 25 | 13528 | 2.7398481 | 1.00E+00 | 4.49E-01 | 6.92E+01 |
| **540** | GO:0032273~positive regulation of protein polymerization | 6 | 0.0219949 | 6.21E-02 | 1185 | 25 | 13528 | 2.7398481 | 1.00E+00 | 4.49E-01 | 6.92E+01 |
| **541** | GO:0032507~maintenance of protein location in cell | 8 | 0.0293266 | 6.30E-02 | 1185 | 41 | 13528 | 2.2275188 | 1.00E+00 | 4.53E-01 | 6.98E+01 |
| **542** | GO:0042454~ribonucleoside catabolic process | 3 | 0.0109975 | 6.40E-02 | 1185 | 5 | 13528 | 6.8496203 | 1.00E+00 | 4.58E-01 | 7.04E+01 |
| **543** | GO:0032964~collagen biosynthetic process | 3 | 0.0109975 | 6.40E-02 | 1185 | 5 | 13528 | 6.8496203 | 1.00E+00 | 4.58E-01 | 7.04E+01 |
| **544** | GO:0006172~ADP biosynthetic process | 3 | 0.0109975 | 6.40E-02 | 1185 | 5 | 13528 | 6.8496203 | 1.00E+00 | 4.58E-01 | 7.04E+01 |
| **545** | GO:0009136~purine nucleoside diphosphate biosynthetic process | 3 | 0.0109975 | 6.40E-02 | 1185 | 5 | 13528 | 6.8496203 | 1.00E+00 | 4.58E-01 | 7.04E+01 |
| **546** | GO:0006102~isocitrate metabolic process | 3 | 0.0109975 | 6.40E-02 | 1185 | 5 | 13528 | 6.8496203 | 1.00E+00 | 4.58E-01 | 7.04E+01 |
| **547** | GO:0034380~high-density lipoprotein particle assembly | 3 | 0.0109975 | 6.40E-02 | 1185 | 5 | 13528 | 6.8496203 | 1.00E+00 | 4.58E-01 | 7.04E+01 |
| **548** | GO:0030240~muscle thin filament assembly | 3 | 0.0109975 | 6.40E-02 | 1185 | 5 | 13528 | 6.8496203 | 1.00E+00 | 4.58E-01 | 7.04E+01 |
| **549** | GO:0009188~ribonucleoside diphosphate biosynthetic process | 3 | 0.0109975 | 6.40E-02 | 1185 | 5 | 13528 | 6.8496203 | 1.00E+00 | 4.58E-01 | 7.04E+01 |
| **550** | GO:0043101~purine salvage | 3 | 0.0109975 | 6.40E-02 | 1185 | 5 | 13528 | 6.8496203 | 1.00E+00 | 4.58E-01 | 7.04E+01 |
| **551** | GO:0010901~regulation of very-low-density lipoprotein particle remodeling | 3 | 0.0109975 | 6.40E-02 | 1185 | 5 | 13528 | 6.8496203 | 1.00E+00 | 4.58E-01 | 7.04E+01 |
| **552** | GO:0009180~purine ribonucleoside diphosphate biosynthetic process | 3 | 0.0109975 | 6.40E-02 | 1185 | 5 | 13528 | 6.8496203 | 1.00E+00 | 4.58E-01 | 7.04E+01 |
| **553** | GO:0009191~ribonucleoside diphosphate catabolic process | 3 | 0.0109975 | 6.40E-02 | 1185 | 5 | 13528 | 6.8496203 | 1.00E+00 | 4.58E-01 | 7.04E+01 |
| **554** | GO:0046457~prostanoid biosynthetic process | 4 | 0.0146633 | 6.48E-02 | 1185 | 11 | 13528 | 4.151285 | 1.00E+00 | 4.61E-01 | 7.08E+01 |
| **555** | GO:0001516~prostaglandin biosynthetic process | 4 | 0.0146633 | 6.48E-02 | 1185 | 11 | 13528 | 4.151285 | 1.00E+00 | 4.61E-01 | 7.08E+01 |
| **556** | GO:0051881~regulation of mitochondrial membrane potential | 4 | 0.0146633 | 6.48E-02 | 1185 | 11 | 13528 | 4.151285 | 1.00E+00 | 4.61E-01 | 7.08E+01 |
| **557** | GO:0009083~branched chain family amino acid catabolic process | 4 | 0.0146633 | 6.48E-02 | 1185 | 11 | 13528 | 4.151285 | 1.00E+00 | 4.61E-01 | 7.08E+01 |
| **558** | GO:0000305~response to oxygen radical | 4 | 0.0146633 | 6.48E-02 | 1185 | 11 | 13528 | 4.151285 | 1.00E+00 | 4.61E-01 | 7.08E+01 |
| **559** | GO:0030030~cell projection organization | 42 | 0.1539646 | 6.49E-02 | 1185 | 368 | 13528 | 1.3029169 | 1.00E+00 | 4.61E-01 | 7.09E+01 |
| **560** | GO:0009628~response to abiotic stimulus | 42 | 0.1539646 | 6.49E-02 | 1185 | 368 | 13528 | 1.3029169 | 1.00E+00 | 4.61E-01 | 7.09E+01 |
| **561** | GO:0006611~protein export from nucleus | 5 | 0.0183291 | 6.64E-02 | 1185 | 18 | 13528 | 3.1711205 | 1.00E+00 | 4.68E-01 | 7.17E+01 |
| **562** | GO:0009127~purine nucleoside monophosphate biosynthetic process | 5 | 0.0183291 | 6.64E-02 | 1185 | 18 | 13528 | 3.1711205 | 1.00E+00 | 4.68E-01 | 7.17E+01 |
| **563** | GO:0032024~positive regulation of insulin secretion | 5 | 0.0183291 | 6.64E-02 | 1185 | 18 | 13528 | 3.1711205 | 1.00E+00 | 4.68E-01 | 7.17E+01 |
| **564** | GO:0007212~dopamine receptor signaling pathway | 5 | 0.0183291 | 6.64E-02 | 1185 | 18 | 13528 | 3.1711205 | 1.00E+00 | 4.68E-01 | 7.17E+01 |
| **565** | GO:0019915~lipid storage | 5 | 0.0183291 | 6.64E-02 | 1185 | 18 | 13528 | 3.1711205 | 1.00E+00 | 4.68E-01 | 7.17E+01 |
| **566** | GO:0009168~purine ribonucleoside monophosphate biosynthetic process | 5 | 0.0183291 | 6.64E-02 | 1185 | 18 | 13528 | 3.1711205 | 1.00E+00 | 4.68E-01 | 7.17E+01 |
| **567** | GO:0019438~aromatic compound biosynthetic process | 5 | 0.0183291 | 6.64E-02 | 1185 | 18 | 13528 | 3.1711205 | 1.00E+00 | 4.68E-01 | 7.17E+01 |
| **568** | GO:0007018~microtubule-based movement | 16 | 0.0586532 | 6.77E-02 | 1185 | 113 | 13528 | 1.6164296 | 1.00E+00 | 4.74E-01 | 7.25E+01 |
| **569** | GO:0042311~vasodilation | 6 | 0.0219949 | 7.15E-02 | 1185 | 26 | 13528 | 2.6344693 | 1.00E+00 | 4.93E-01 | 7.45E+01 |
| **570** | GO:0048514~blood vessel morphogenesis | 26 | 0.0953114 | 7.37E-02 | 1185 | 211 | 13528 | 1.4067151 | 1.00E+00 | 5.02E-01 | 7.55E+01 |
| **571** | GO:0006662~glycerol ether metabolic process | 9 | 0.0329924 | 7.38E-02 | 1185 | 51 | 13528 | 2.0145942 | 1.00E+00 | 5.02E-01 | 7.56E+01 |
| **572** | GO:0051603~proteolysis involved in cellular protein catabolic process | 64 | 0.2346127 | 7.42E-02 | 1185 | 600 | 13528 | 1.2177103 | 1.00E+00 | 5.03E-01 | 7.58E+01 |
| **573** | GO:0009108~coenzyme biosynthetic process | 11 | 0.0403241 | 7.66E-02 | 1185 | 69 | 13528 | 1.8199474 | 1.00E+00 | 5.14E-01 | 7.69E+01 |
| **574** | GO:0006839~mitochondrial transport | 11 | 0.0403241 | 7.66E-02 | 1185 | 69 | 13528 | 1.8199474 | 1.00E+00 | 5.14E-01 | 7.69E+01 |
| **575** | GO:0006641~triglyceride metabolic process | 8 | 0.0293266 | 7.77E-02 | 1185 | 43 | 13528 | 2.1239133 | 1.00E+00 | 5.18E-01 | 7.74E+01 |
| **576** | GO:0060048~cardiac muscle contraction | 5 | 0.0183291 | 7.85E-02 | 1185 | 19 | 13528 | 3.0042194 | 1.00E+00 | 5.21E-01 | 7.77E+01 |
| **577** | GO:0051270~regulation of cell motion | 24 | 0.0879798 | 7.94E-02 | 1185 | 193 | 13528 | 1.4196104 | 1.00E+00 | 5.25E-01 | 7.82E+01 |
| **578** | GO:0044257~cellular protein catabolic process | 64 | 0.2346127 | 7.96E-02 | 1185 | 603 | 13528 | 1.211652 | 1.00E+00 | 5.25E-01 | 7.82E+01 |
| **579** | GO:0006576~biogenic amine metabolic process | 14 | 0.0513215 | 7.99E-02 | 1185 | 97 | 13528 | 1.647675 | 1.00E+00 | 5.25E-01 | 7.84E+01 |
| **580** | GO:0051188~cofactor biosynthetic process | 14 | 0.0513215 | 7.99E-02 | 1185 | 97 | 13528 | 1.647675 | 1.00E+00 | 5.25E-01 | 7.84E+01 |
| **581** | GO:0016126~sterol biosynthetic process | 7 | 0.0256608 | 8.07E-02 | 1185 | 35 | 13528 | 2.2832068 | 1.00E+00 | 5.28E-01 | 7.87E+01 |
| **582** | GO:0048199~vesicle targeting, to, from or within Golgi | 4 | 0.0146633 | 8.10E-02 | 1185 | 12 | 13528 | 3.8053446 | 1.00E+00 | 5.28E-01 | 7.88E+01 |
| **583** | GO:0003009~skeletal muscle contraction | 4 | 0.0146633 | 8.10E-02 | 1185 | 12 | 13528 | 3.8053446 | 1.00E+00 | 5.28E-01 | 7.88E+01 |
| **584** | GO:0006906~vesicle fusion | 4 | 0.0146633 | 8.10E-02 | 1185 | 12 | 13528 | 3.8053446 | 1.00E+00 | 5.28E-01 | 7.88E+01 |
| **585** | GO:0045214~sarcomere organization | 4 | 0.0146633 | 8.10E-02 | 1185 | 12 | 13528 | 3.8053446 | 1.00E+00 | 5.28E-01 | 7.88E+01 |
| **586** | GO:0034375~high-density lipoprotein particle remodeling | 4 | 0.0146633 | 8.10E-02 | 1185 | 12 | 13528 | 3.8053446 | 1.00E+00 | 5.28E-01 | 7.88E+01 |
| **587** | GO:0050766~positive regulation of phagocytosis | 4 | 0.0146633 | 8.10E-02 | 1185 | 12 | 13528 | 3.8053446 | 1.00E+00 | 5.28E-01 | 7.88E+01 |
| **588** | GO:0055008~cardiac muscle tissue morphogenesis | 6 | 0.0219949 | 8.18E-02 | 1185 | 27 | 13528 | 2.5368964 | 1.00E+00 | 5.31E-01 | 7.92E+01 |
| **589** | GO:0060415~muscle tissue morphogenesis | 6 | 0.0219949 | 8.18E-02 | 1185 | 27 | 13528 | 2.5368964 | 1.00E+00 | 5.31E-01 | 7.92E+01 |
| **590** | GO:0045428~regulation of nitric oxide biosynthetic process | 6 | 0.0219949 | 8.18E-02 | 1185 | 27 | 13528 | 2.5368964 | 1.00E+00 | 5.31E-01 | 7.92E+01 |
| **591** | GO:0006869~lipid transport | 19 | 0.0696506 | 8.24E-02 | 1185 | 145 | 13528 | 1.4958941 | 1.00E+00 | 5.33E-01 | 7.94E+01 |
| **592** | GO:0043632~modification-dependent macromolecule catabolic process | 61 | 0.2236152 | 8.62E-02 | 1185 | 574 | 13528 | 1.2132022 | 1.00E+00 | 5.49E-01 | 8.09E+01 |
| **593** | GO:0019941~modification-dependent protein catabolic process | 61 | 0.2236152 | 8.62E-02 | 1185 | 574 | 13528 | 1.2132022 | 1.00E+00 | 5.49E-01 | 8.09E+01 |
| **594** | GO:0007049~cell cycle | 80 | 0.2932659 | 8.65E-02 | 1185 | 776 | 13528 | 1.1769107 | 1.00E+00 | 5.50E-01 | 8.11E+01 |
| **595** | GO:0006605~protein targeting | 26 | 0.0953114 | 8.72E-02 | 1185 | 215 | 13528 | 1.3805436 | 1.00E+00 | 5.52E-01 | 8.13E+01 |
| **596** | GO:0018904~organic ether metabolic process | 9 | 0.0329924 | 8.84E-02 | 1185 | 53 | 13528 | 1.9385718 | 1.00E+00 | 5.56E-01 | 8.18E+01 |
| **597** | GO:0055080~cation homeostasis | 33 | 0.1209722 | 8.89E-02 | 1185 | 286 | 13528 | 1.3172347 | 1.00E+00 | 5.57E-01 | 8.19E+01 |
| **598** | GO:0051336~regulation of hydrolase activity | 38 | 0.1393013 | 8.98E-02 | 1185 | 337 | 13528 | 1.2872679 | 1.00E+00 | 5.60E-01 | 8.23E+01 |
| **599** | GO:0010741~negative regulation of protein kinase cascade | 7 | 0.0256608 | 9.01E-02 | 1185 | 36 | 13528 | 2.2197843 | 1.00E+00 | 5.60E-01 | 8.24E+01 |
| **600** | GO:0006122~mitochondrial electron transport, ubiquinol to cytochrome c | 3 | 0.0109975 | 9.06E-02 | 1185 | 6 | 13528 | 5.7080169 | 1.00E+00 | 5.62E-01 | 8.25E+01 |
| **601** | GO:0045039~protein import into mitochondrial inner membrane | 3 | 0.0109975 | 9.06E-02 | 1185 | 6 | 13528 | 5.7080169 | 1.00E+00 | 5.62E-01 | 8.25E+01 |
| **602** | GO:0046487~glyoxylate metabolic process | 3 | 0.0109975 | 9.06E-02 | 1185 | 6 | 13528 | 5.7080169 | 1.00E+00 | 5.62E-01 | 8.25E+01 |
| **603** | GO:0035304~regulation of protein amino acid dephosphorylation | 3 | 0.0109975 | 9.06E-02 | 1185 | 6 | 13528 | 5.7080169 | 1.00E+00 | 5.62E-01 | 8.25E+01 |
| **604** | GO:0030300~regulation of intestinal cholesterol absorption | 3 | 0.0109975 | 9.06E-02 | 1185 | 6 | 13528 | 5.7080169 | 1.00E+00 | 5.62E-01 | 8.25E+01 |
| **605** | GO:0034384~high-density lipoprotein particle clearance | 3 | 0.0109975 | 9.06E-02 | 1185 | 6 | 13528 | 5.7080169 | 1.00E+00 | 5.62E-01 | 8.25E+01 |
| **606** | GO:0055003~cardiac myofibril assembly | 3 | 0.0109975 | 9.06E-02 | 1185 | 6 | 13528 | 5.7080169 | 1.00E+00 | 5.62E-01 | 8.25E+01 |
| **607** | GO:0005980~glycogen catabolic process | 3 | 0.0109975 | 9.06E-02 | 1185 | 6 | 13528 | 5.7080169 | 1.00E+00 | 5.62E-01 | 8.25E+01 |
| **608** | GO:0006538~glutamate catabolic process | 3 | 0.0109975 | 9.06E-02 | 1185 | 6 | 13528 | 5.7080169 | 1.00E+00 | 5.62E-01 | 8.25E+01 |
| **609** | GO:0006448~regulation of translational elongation | 3 | 0.0109975 | 9.06E-02 | 1185 | 6 | 13528 | 5.7080169 | 1.00E+00 | 5.62E-01 | 8.25E+01 |
| **610** | GO:0018198~peptidyl-cysteine modification | 3 | 0.0109975 | 9.06E-02 | 1185 | 6 | 13528 | 5.7080169 | 1.00E+00 | 5.62E-01 | 8.25E+01 |
| **611** | GO:0042532~negative regulation of tyrosine phosphorylation of STAT protein | 3 | 0.0109975 | 9.06E-02 | 1185 | 6 | 13528 | 5.7080169 | 1.00E+00 | 5.62E-01 | 8.25E+01 |
| **612** | GO:0009134~nucleoside diphosphate catabolic process | 3 | 0.0109975 | 9.06E-02 | 1185 | 6 | 13528 | 5.7080169 | 1.00E+00 | 5.62E-01 | 8.25E+01 |
| **613** | GO:0019439~aromatic compound catabolic process | 5 | 0.0183291 | 9.16E-02 | 1185 | 20 | 13528 | 2.8540084 | 1.00E+00 | 5.65E-01 | 8.29E+01 |
| **614** | GO:0045104~intermediate filament cytoskeleton organization | 5 | 0.0183291 | 9.16E-02 | 1185 | 20 | 13528 | 2.8540084 | 1.00E+00 | 5.65E-01 | 8.29E+01 |
| **615** | GO:0007159~leukocyte adhesion | 6 | 0.0219949 | 9.27E-02 | 1185 | 28 | 13528 | 2.4462929 | 1.00E+00 | 5.69E-01 | 8.33E+01 |
| **616** | GO:0015908~fatty acid transport | 6 | 0.0219949 | 9.27E-02 | 1185 | 28 | 13528 | 2.4462929 | 1.00E+00 | 5.69E-01 | 8.33E+01 |
| **617** | GO:0031345~negative regulation of cell projection organization | 6 | 0.0219949 | 9.27E-02 | 1185 | 28 | 13528 | 2.4462929 | 1.00E+00 | 5.69E-01 | 8.33E+01 |
| **618** | GO:0000387~spliceosomal snRNP biogenesis | 6 | 0.0219949 | 9.27E-02 | 1185 | 28 | 13528 | 2.4462929 | 1.00E+00 | 5.69E-01 | 8.33E+01 |
| **619** | GO:0007051~spindle organization | 8 | 0.0293266 | 9.44E-02 | 1185 | 45 | 13528 | 2.0295171 | 1.00E+00 | 5.75E-01 | 8.38E+01 |
| **620** | GO:0046890~regulation of lipid biosynthetic process | 8 | 0.0293266 | 9.44E-02 | 1185 | 45 | 13528 | 2.0295171 | 1.00E+00 | 5.75E-01 | 8.38E+01 |
| **621** | GO:0006919~activation of caspase activity | 9 | 0.0329924 | 9.62E-02 | 1185 | 54 | 13528 | 1.9026723 | 1.00E+00 | 5.81E-01 | 8.44E+01 |
| **622** | GO:0002684~positive regulation of immune system process | 28 | 0.1026431 | 9.79E-02 | 1185 | 238 | 13528 | 1.3430628 | 1.00E+00 | 5.87E-01 | 8.49E+01 |
| **623** | GO:0031109~microtubule polymerization or depolymerization | 4 | 0.0146633 | 9.87E-02 | 1185 | 13 | 13528 | 3.5126258 | 1.00E+00 | 5.89E-01 | 8.52E+01 |
| **624** | GO:0031113~regulation of microtubule polymerization | 4 | 0.0146633 | 9.87E-02 | 1185 | 13 | 13528 | 3.5126258 | 1.00E+00 | 5.89E-01 | 8.52E+01 |
| **625** | GO:0030866~cortical actin cytoskeleton organization | 4 | 0.0146633 | 9.87E-02 | 1185 | 13 | 13528 | 3.5126258 | 1.00E+00 | 5.89E-01 | 8.52E+01 |
| **626** | GO:0044236~multicellular organismal metabolic process | 7 | 0.0256608 | 1.00E-01 | 1185 | 37 | 13528 | 2.1597902 | 1.00E+00 | 5.93E-01 | 8.56E+01 |

**Supplementary Table S3:**
**Relationship types between proteins identified in the significant (p<0.05) vasoactive pathways in the porcine sPCA_IPA analysis**

| From Molecule(s) | Relationship Type | To Molecule(s) | Catalyst(s) |
|---|---|---|---|
| 4.1R | protein-protein interactions | FODRIN | |
| 4.1R | protein-protein interactions | ZO2 | |
| AFADIN | protein-protein interactions | F-ACTIN | |
| AFADIN | protein-protein interactions | JAM | |
| AFADIN | protein-protein interactions | NECTIN | |
| AKT | causation | Cell proliferation | |
| CASK | protein-protein interactions | JAM | |
| CDC42 | protein-protein interactions | PAR6 | |
| CDK4-ZONAB | causation | G1/S phase transition | |
| CDK4-ZONAB | membership | CDK4 | |
| CDK4-ZONAB | membership | ZONAB | |
| CEBP | protein-protein interactions | AP-1 | |
| CEBP-AP-1 | causation | Epithelial celldifferentiation | |
| CEBP-AP-1 | causation | Epithelial cellproliferation | |
| CEBP-AP-1 | membership | AP-1 | |
| CEBP-AP-1 | membership | CEBP | |
| CGN | inhibition | GEFH1 | |
| CGN | protein-protein interactions | F-ACTIN | |
| CGN | protein-protein interactions | GEFH1 | |
| CGN | protein-protein interactions | JAM | |
| CGN | protein-protein interactions | MYOSIN | |
| CGN | protein-protein interactions | ZO1 | |
| CGN | protein-protein interactions | ZO2 | |
| CGN | protein-protein interactions | ZO3 | |
| CLDN | protein-protein interactions | CLDN | |
| CRB3 | protein-protein interactions | PALS1 | |
| CRB3 | protein-protein interactions | PAR6 | |
| CRB3 | protein-protein interactions | PATJ | |
| CSTF-CPSF-SYMPK | causation | mRNApolyadenylation | |
| CSTF-CPSF-SYMPK | membership | CPSF | |
| CSTF-CPSF-SYMPK | membership | CSTF | |
| CSTF-CPSF-SYMPK | membership | SYMPK | |
| CTNNB1 | protein-protein interactions | MAGI-2 | |
| CTNNB1 | reaction | MAGI-2 | VCL |
| F-ACTIN | causation | Actinnucleation | |
| GEFH1 | activation | RHOA | |
| JAM | protein-protein interactions | JAM | |
| JAM | protein-protein interactions | ZO1 | |
| MAGI-1 | protein-protein interactions | JAM | |
| MAGI-2 | activation | PTEN | |
| MAGI-2 | protein-protein interactions | PTEN | |
| MLCK | causation | Tight junctionpermeability | |
| MUPP1 | protein-protein interactions | CLDN | |
| NECTIN | protein-protein interactions | AFADIN | |
| NECTIN | protein-protein interactions | CLDN | |
| NECTIN | protein-protein interactions | OCLN | |
| NF-κB | expression | MLCK | |
| OCLN | protein-protein interactions | CGN | |
| OCLN | protein-protein interactions | F-ACTIN | |
| OCLN | protein-protein interactions | OCLN | |
| OCLN | protein-protein interactions | ZO1 | |
| OCLN | protein-protein interactions | ZO2 | |

**Supplementary Table S3: (continued)**

| | | | |
|---|---|---|---|
| OCLN | protein-protein interactions | ZO3 | |
| PAR3 | protein-protein interactions | JAM | |
| PAR3 | protein-protein interactions | PAR6 | |
| PAR3 | protein-protein interactions | TIAM1 | |
| PAR6 | activation | SMURF1 | |
| PAR6 | protein-protein interactions | PALS1 | |
| PAR6 | protein-protein interactions | SMURF1 | |
| PAR6 | protein-protein interactions | aPKC | |
| PAR6 | protein-protein interactions | mLGL | |
| PATJ | protein-protein interactions | PALS1 | |
| PATJ | protein-protein interactions | ZO3 | |
| PKA | activation | VASP | |
| PKA | phosphorylation | VASP | |
| PP2A | inhibition | aPKC | |
| PTEN | inhibition | AKT | |
| RAB13 | inhibition | PKA | |
| RAB13 | protein-protein interactions | PKA | |
| RHOA | causation | Actinorganization | |
| RHOA | causation | Epithelial to mesenchymal transition(EMT) | |
| SMURF1 | inhibition | RHOA | |
| SNARE | causation | Vesicletrafficking | |
| STX4 | causation | Vesicletransport | |
| STX4 | protein-protein interactions | SNARE | |
| STX4 | protein-protein interactions | mLGL | |
| SYMPK | activation | HSF1 | |
| SYMPK | protein-protein interactions | CPSF | |
| SYMPK | protein-protein interactions | CSTF | |
| SYMPK | protein-protein interactions | HSF1 | |
| TGF-β | activation | TGFBR | |
| TGF-β | protein-protein interactions | TGFBR | |
| TGFBR | phosphorylation | PAR6 | |
| TIAM1 | activation | RAC1 | |
| TIAM1 | protein-protein interactions | PAR3 | |
| TNF-α | activation | TNFR | |
| TNF-α | protein-protein interactions | TNFR | |
| VAP33 | protein-protein interactions | OCLN | |
| VASP | protein-protein interactions | F-ACTIN | |
| VCL | protein-protein interactions | CTNNA1 | |
| ZO1 | protein-protein interactions | CASK | |
| ZO1 | protein-protein interactions | CGN | |
| ZO1 | protein-protein interactions | CLDN | |
| ZO1 | protein-protein interactions | CTNNA1 | |
| ZO1 | protein-protein interactions | F-ACTIN | |
| ZO1 | protein-protein interactions | FODRIN | |
| ZO1 | protein-protein interactions | RAB13 | |
| ZO1 | protein-protein interactions | ZO2 | |
| ZO1 | protein-protein interactions | ZO3 | |
| ZO1 | protein-protein interactions | ZONAB | |
| ZO2 | inhibition | CEBP-AP-1 | |
| ZO2 | protein-protein interactions | 4.1R | |
| ZO2 | protein-protein interactions | CLDN | |
| ZO2 | protein-protein interactions | F-ACTIN | |
| ZO2 | protein-protein interactions | SAFB | |
| ZO3 | protein-protein interactions | CLDN | |
| ZO3 | protein-protein interactions | F-ACTIN | |
| ZONAB | protein-protein interactions | CDK4 | |
| ZONAB | reaction | ZONAB | ZO1 |
| ZONAB | translocation | ZONAB | |
| aPKC | inhibition | MARK2 | |
| aPKC | inhibition | mLGL | |
| aPKC | phosphorylation | MARK2 | |
| aPKC | phosphorylation | mLGL | |
| aPKC | protein-protein interactions | PAR3 | |
| aPKC | protein-protein interactions | PAR6 | |

**Supplementary Table S4:**
**PANTHER analysis of Parkinson's and Huntington' disease pathways**

**Parkinson disease**

| Gene Name | Protein names |
|---|---|
| HSPA1B | Heat shock 70 kDa protein 1B |
| HSPA1L | Heat shock 70 kDa protein 1-like |
| HSPA2 | Heat shock-related 70 kDa protein 2 |
| HSPA5 | 78 kDa glucose-regulated protein |
| HSPA6 | Heat shock 70 kDa protein 6 |
| HSPA8 | Heat shock cognate 71 kDa protein |
| HSPA9 | Stress-70 protein, mitochondrial |
| MAPK1 | Mitogen-activated protein kinase 1 |
| MAPK3 | Mitogen-activated protein kinase 3 |
| NDUFV2 | NADH dehydrogenase [ubiquinone] flavoprotein 2, mitochondrial |
| PSMA1 | Proteasome subunit alpha type-1 |
| PSMA2 | Proteasome subunit alpha type-2 |
| PSMA3 | Proteasome subunit alpha type-3 |
| PSMA4 | Proteasome subunit alpha type-4 |
| PSMA5 | Proteasome subunit alpha type-5 |
| PSMA6 | Proteasome subunit alpha type-6 |
| PSMA7 | Proteasome subunit alpha type-7 |
| PSMB1 | Proteasome subunit beta type-1 |
| PSMB10 | Proteasome subunit beta type-10 |
| PSMB3 | Proteasome subunit beta type-3 |
| PSMB4 | Proteasome subunit beta type-4 |
| SEPT2 | Septin-2 |
| SFN | 14-3-3 protein sigma |
| SNCB | Beta-synuclein |
| SNCG | Gamma-synuclein |
| UBE2L3 | Ubiquitin-conjugating enzyme E2 L3 |
| YWHAB | 14-3-3 protein beta/alpha |
| YWHAE | 14-3-3 protein epsilon |
| YWHAG | 14-3-3 protein gamma |
| YWHAH | 14-3-3 protein eta |
| YWHAQ | 14-3-3 protein theta |
| YWHAZ | 14-3-3 protein zeta/delta |

**Huntington disease**

| Gene Name | Protein names |
|---|---|
| ACTR1A | Alpha-centractin |
| ACTR1B | Beta-centractin |
| ACTR2 | Actin-related protein 2 |
| ACTR3 | Actin-related protein 3 |
| AKT2 | RAC-beta serine/threonine-protein kinase |
| ARF1 | ADP-ribosylation factor 1 |
| ARF4 | ADP-ribosylation factor 4 |
| ARL8B | ADP-ribosylation factor-like protein 8B |
| ARPC1A | Actin-related protein 2/3 complex subunit 1A |
| ARPC1B | Actin-related protein 2/3 complex subunit 1B |
| ARPC5 | Actin-related protein 2/3 complex subunit 5 |
| ARPC5L | Actin-related protein 2/3 complex subunit 5-like protein |
| CAPN1 | Calpain-1 catalytic subunit |
| CAPN2 | Calpain-2 catalytic subunit |
| CAPNS1 | Calpain small subunit 1 |
| CDC42 | Cell division control protein 42 homolog |
| CLTA | Clathrin light chain A |
| CLTB | Clathrin light chain B |
| CRYZ | Quinone oxidoreductase |
| CYC1 | Cytochrome c1, heme protein, mitochondrial |
| CYFIP1 | Cytoplasmic FMR1-interacting protein 1 |
| DNAH12 | Dynein heavy chain 12, axonemal |
| DYNC1H1 | Cytoplasmic dynein 1 heavy chain 1 |
| NCOR1 | Nuclear receptor corepressor 1 |
| PACSIN1 | Protein kinase C and casein kinase substrate in neurons protein 1 |
| PACSIN2 | Protein kinase C and casein kinase substrate in neurons protein 2 |
| RAB11B | Ras-related protein Rab-11 |
| RAC1 | Ras-related C3 botulinum toxin substrate 1 |
| RHOG | Rho-related GTP-binding protein RhoG |
| TRIM23 | E3 ubiquitin-protein ligase TRIM23 |
| TUBB | Tubulin beta chain |
| TUBB2A | Tubulin beta-2A chain |
| TUBB2C | Tubulin beta-4B chain |
| TUBB3 | Tubulin beta-3 chain |
| VAT1 | Synaptic vesicle membrane protein VAT-1 homolog |
| VAT1L | Synaptic vesicle membrane protein VAT-1 homolog-like |
